# Supplementary material for: Anti-selective [3+2] (Hetero)annulation of non-conjugated alkenes via directed nucleopalladation
Source: Nat Commun. 2020 Dec 22;11:6432. doi: 10.1038/s41467-020-20182-4 (PMC7755910; doi:10.1038/s41467-020-20182-4)
Supplement: Supplementary file 1 — Supplementary Information [file 41467_2020_20182_MOESM1_ESM.pdf]

SUPPLEMENTARY INFORMATION

Anti-Selective [3+2] (Hetero)annulation of Non-Conjugated Alkenes via Directed Nucleopalladation

Hui-Qi Ni†, Ilia Kevlishvili‡, Pranali G. Bedekar†, Joyann S. Barber§, Shouliang Yang§, Michelle Tran-Dubé§, Andrew M. Romine†, Hou-Xiang Lu†, Indrawan J. McAlpine§*, Peng Liu‡*, and Keary M. Engle†*

†Department of Chemistry, Scripps Research, 10550 North Torrey Pines Road, La Jolla, California 92037, United States
‡Department of Chemistry, University of Pittsburgh, Pittsburgh, Pennsylvania 15260, United States
§Pfizer Oncology Medicinal Chemistry, 10770 Science Center Drive, San Diego, California 92121, United States

* indrawan.mcalpine@pfizer.com; pengliu@pitt.edu; keary@scripps.edu

Table of Contents

General Information……S-2
Experimental Procedures……S-2
 Alkene Substrate Synthesis……S-2
 Protected *ortho*-Iodoaniline Synthesis……S-5
 C-Nucleophile Synthesis……S-5
 Reaction Optimization Details……S-6
 Mechanistic Experiments……S-9
 Competition Experiments……S-14
 General Procedure A for [3+2] 2,3-Dihydrobenzofuran Synthesis……S-15
 General Procedure B for [3+2] Indoline Synthesis……S-22
 General Procedure C for [3+2] Indane Synthesis……S-33
 General Procedure D for [3+2] Reactions with NHPA Directing groups……S-34
 General Procedure E for Deprotection of AQ Directing Group……S-37
 Procedure F for 50 mmol-Scale Reaction……S-38
 Unsuccessful Examples……S-39
X-Ray Crystallography……S-40
Computational Details……S-77
 Additional Computational Results……S-77
NMR Spectra……S-82
References……S-142

GENERAL INFORMATION

Unless otherwise noted, all materials were used as received from commercial sources without further purification. All *ortho*-iodophenols and *ortho*-iodoanilines were purchased from Combi-Blocks or obtained from the internal Pfizer compound library. HFIP (99.5%) was purchased from Oakwood Products Inc. $Pd(OAc)_2$ was obtained from Johnson Matthey. Thin layer chromatography (TLC) was conducted with EMD gel 60 F254 pre-coated plates (0.25 mm) and visualized using a combination of UV light. Preparative thin layer chromatography (TLC) was conducted with Analtech silica gel GF UV254 pre-coated plates (1.0 mm) and visualized using UV light. Silicycle Siliaflash P60 (particle size 0.040–0.063 mm) was used for flash column chromatography. NMR spectra were recorded on Bruker AV-400, DRX500 and AV-600 instruments. Spectra were internally referenced to $SiMe_4$ or solvent signals. The following abbreviations (or combinations thereof) were used to explain multiplicities: s = singlet, d = doublet, t = triplet, q = quartet, p = pentet, m = multiplet. High-resolution mass spectra (HRMS) for new compounds were recorded on an Agilent LC/MSD TOF mass spectrometer.

EXPERIMENTAL PROCEDURES

Alkene Substrate Synthesis

Supplementary Table 1. Alkene substrates **1a**–**i**.

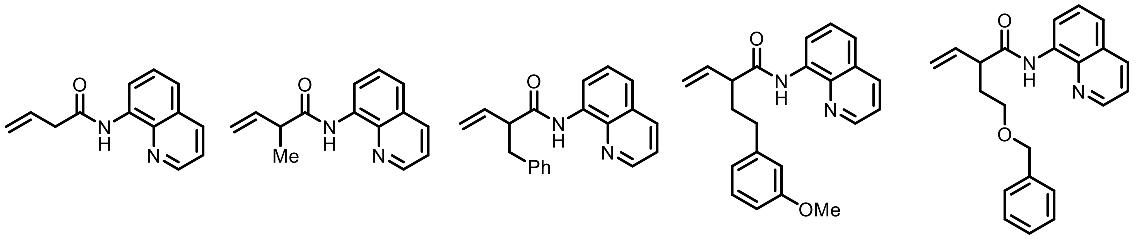

1a **1b** **1c** **1d** **1e**

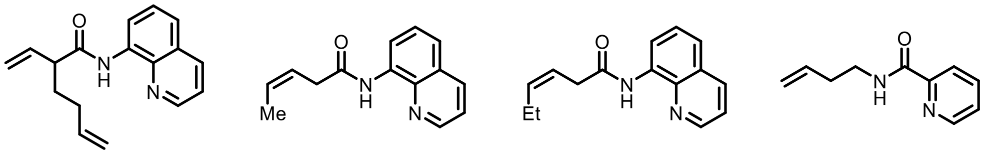

1f **1g** **1h** **1i**

Alkene substrates **1a**–**i** were prepared according to literature procedures.[1-9]

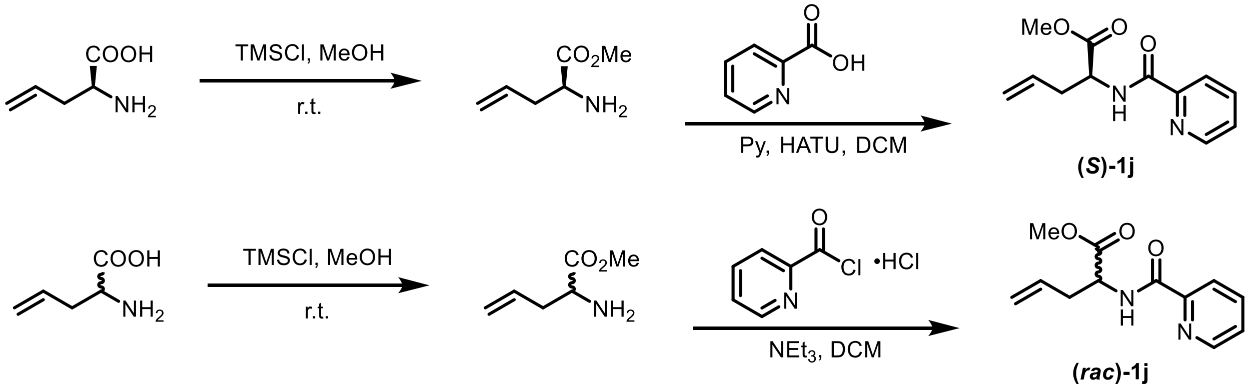

Supplementary Figure 1: Synthesis of methyl **(*S*)-1j** and **(*rac*)-1j**.

Methyl (*S*)-2-(picolinamido)pent-4-enoate ((*S*)-1j): Commercially available (*S*)-2-Aminopent-4-enoic acid (1.0 g, 8.69 mmol) was charged into a 25-mL round-bottom flask equipped with a Teflon-coated magnetic stir bar to which MeOH (10 mL, 0.87 M) was added. The solution was cooled to 0 °C, and TMSCl (3 mL, 23.64 mmol, 2.7 equiv) was added dropwise. The reaction was allowed to warm to room temperature and stir for 24 h, the reaction mixture was concentrated using a rotary evaporator. The crude product was carried forward to the next step without further purification. The crude product was charged into a 50-mL round-bottom flask containing DCM (15 mL, 0.58 M). Picolinic acid (615 mg, 5 mmol, 0.58 equiv), pyridine (0.9 mL, 12 mmol, 1.38 equiv), and HATU (2.5 g, 6.5 mmol, 0.75 equiv) were added sequentially, and the reaction mixture was stirred at room temperature for 16 h. The deep-brown solution was diluted with EtOAc (100 mL), washed with sat. $NaHCO_3$ (50 mL, ×2) and brine (50 mL, ×1), concentrated under reduced pressure, and purified by silica gel column chromatography (hexane:EtOAc = 10:1) to afford 9.2 g (79% yield from picolinic acid) of **(*S*)-1j** as a colorless oil. The purified sample was diluted with MeCN to approximately 0.1 mg/mL and run on 1D chiral-SFC to determine *ee*. **^{1}H NMR** (600 MHz, $CDCl_3$) δ 8.58 (ddd, *J* = 4.8, 1.7, 0.9 Hz, 1H), 8.51 (d, *J* = 8.4 Hz, 1H), 8.17 (dt, *J* = 7.8, 1.1 Hz, 1H), 7.84 (td, *J* = 7.7, 1.7 Hz, 1H), 7.43 (ddd, *J* = 7.6, 4.8, 1.2 Hz, 1H), 5.79 (ddt, *J* = 17.2, 10.1, 7.2 Hz, 1H), 5.26–5.10 (m, 2H), 4.87 (ddd, *J* = 8.3, 6.6, 5.4 Hz, 1H), 3.78 (s, 3H), 2.80–2.68 (m, 1H), 2.70–2.58 (m, 1H). **^{13}C NMR** (150 MHz, $CDCl_3$) δ 172.10, 164.20, 149.50, 148.44, 137.42, 132.42, 126.52, 122.44, 119.37, 52.55, 52.01, 36.82. **HRMS (ESI-TOF)** Calcd for $C_{12}H_{15}N_2O_3^+$ [M+H]$^+$ 235.1083, found 235.1085.

SAMPLE INFORMATION

Sample Name:	NH-371	Acquired By:	System
Sample Type:	Unknown	Sample Set Name	Samples_SFC
Vial:	1:A,2	Acq. Method Set:	G_ENG0301
Injection #:	1	Processing Method	ENG0301
Injection Volume:	2.00 ul	Channel Name:	214.0nm
Run Time:	3.5 Minutes	Proc. Chnl. Descr.:	PDA Spectrum PDA 214.0 nm
Date Acquired:	11/25/2019 4:19:54 PM PST		
Date Processed:	11/25/2019 4:23:27 PM PST		

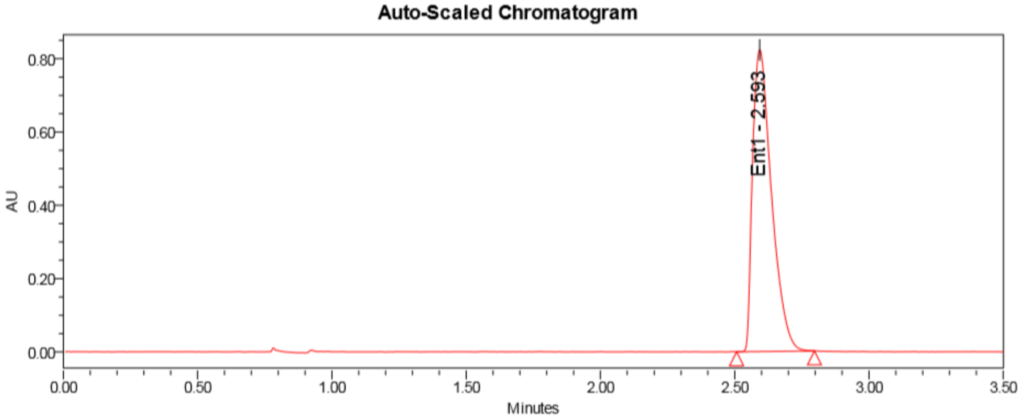

	SampleName	ent1	ent2	ee	Ent1	Ent2
1	NH-272	49.67	50.33	-0.66	1808203	1832228
2	NH-371	100.00		100.00	4036372	

Supplementary Figure 2: SFC result for **(*S*)-1j** (NH-371).

Methyl 2-(picolinamido)pent-4-enoate ((*rac*)-1j): 2-Aminopent-4-enoic acid (1.0 g, 8.69 mmol) was charged into a 25-mL round-bottom flask equipped with a Teflon-coated magnetic stir bar to which MeOH (10 mL, 0.87 M) was added. The solution was cooled to 0 °C, and then TMSCl (3

mL, 23.64 mmol, 2.7 equiv) was added dropwise. The reaction was allowed to warm to room temperature and stir for 24 h, the reaction mixture was concentrated using a rotary evaporator. The crude product was carried forward to the next step without further purification. The crude product was charged into a 50-mL round-bottom flask containing DCM (25 mL, 0.58 M), and picolinoyl chloride hydrochloride (1.1g, 6 mmol, 0.69 equiv) were added sequentially. The solution was cooled to 0 °C, and then Et_3N (2.2 mL, 11 mmol, 1.27 equiv) was added dropwise. The reaction mixture was stirred at room temperature for 16 h. The solution was quenched with water (100 mL) and extracted with DCM (100 mL ×2). The combined organic layers were washed with brine (10 mL, ×1), concentrated under reduced pressure, and purified by silica gel column chromatography (hexane:EtOAc = 10:1) to afford 8.1 g (58% yield from picolinoyl chloride hydrochloride) of **(*rac*)-1j**, as a colorless oil. The purified sample was diluted with MeCN to approximately 0.1 mg/mL and run on 1D chiral-SFC to determine *ee*. **^{1}H NMR** (600 MHz, $CDCl_3$) δ 8.57 (ddd, *J* = 4.8, 1.7, 0.9 Hz, 1H), 8.50 (d, *J* = 8.2 Hz, 1H), 8.16 (dt, *J* = 7.8, 1.1 Hz, 1H), 7.83 (td, *J* = 7.7, 1.7 Hz, 1H), 7.43 (ddd, *J* = 7.6, 4.8, 1.2 Hz, 1H), 5.78 (ddt, *J* = 17.2, 10.1, 7.2 Hz, 1H), 5.21–5.14 (m, 2H), 4.87 (ddd, *J* = 8.2, 6.6, 5.4 Hz, 1H), 3.77 (s, 3H), 2.72 (dddt, *J* = 13.9, 6.9, 5.4, 1.2 Hz, 1H), 2.64 (dtt, *J* = 14.1, 6.8, 1.2 Hz, 1H). **^{13}C NMR** (150 MHz, $CDCl_3$) δ 172.08, 164.18, 149.48, 148.43, 137.40, 132.40, 126.50, 122.41, 119.35, 52.52, 51.99, 36.80. **HRMS (ESI-TOF)** Calcd for $C_{12}H_{15}N_2O_3^+$ $[M+H]^+$ 235.1083, found 235.1084.

SAMPLE INFORMATION

Sample Name:	NH-272	Acquired By:	System
Sample Type:	Unknown	Sample Set Name	
Vial:	1:A,1	Acq. Method Set:	B_MeOH_03_SFC
Injection #:	6	Processing Method	ENG0301
Injection Volume:	2.00 ul	Channel Name:	214.0nm
Run Time:	10.0 Minutes	Proc. Chnl. Descr.:	PDA Spectrum PDA 214.0 nm
Date Acquired:	11/25/2019 4:12:45 PM PST		
Date Processed:	11/25/2019 4:19:41 PM PST		

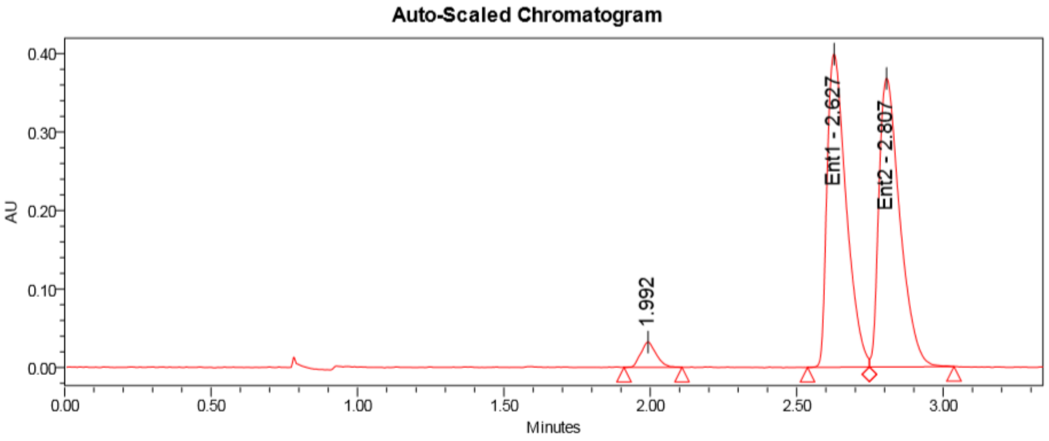

	SampleName	ent1	ent2	ee	Ent1	Ent2
1	NH-272	49.67	50.33	-0.66	1808203	1832228
2	NH-371	100.00		100.00	4036372	

Supplementary Figure 3: SFC results for **(*rac*)-1j (NH-272).**

Protected ortho-Iodoaniline Synthesis

Supplementary Table 2. *ortho*-Iodoanilines **2bv**, **2bt**, **2bx**, and **2by**.

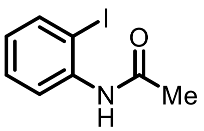

2bv

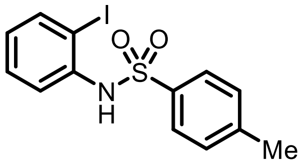

2bt

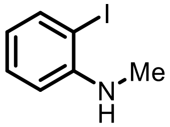

2bx

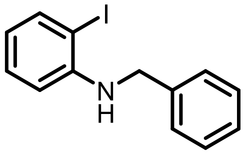

2by

ortho-Iodoanilines **2bv**[10], **2bt**[11], **2bx**[12], and **2by**[13] were prepared according to literature procedures.

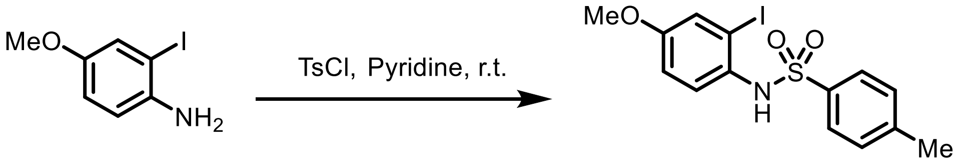

2bd **2bu**

Supplementary Figure 4: Synthesis of *N*-(2-iodo-4-methoxyphenyl)-4-methylbenzenesulfonamide (**2bu**).

***N*-(2-iodo-4-methoxyphenyl)-4-methylbenzenesulfonamide (2bu)**: 2-Iodo-4-methoxyaniline (0.57 g, 2.3 mmol) and TsCl (0.46 g, 2.4 mmol, 1.05 equiv) were charged into a 25-mL round-bottom flask containing pyridine (5 mL) that was equipped with a Teflon-coated magnetic stir bar. The reaction mixture was stirred at room temperature for 1.5 h. The solution was quenched with water (50 mL) and extracted DCM (50 mL, ×3). The combined organic layers were dried over $NaSO_4$, filtered, and concentrated under reduced pressure, and purified by silica gel column chromatography (hexane:EtOAc = 3:1) to afford **2bu** (0.2 g, 22% yield) as a brown solid. **^{1}H NMR** (600 MHz, $CDCl_3$) δ 7.60 – 7.53 (m, 3H), 7.23 – 7.18 (m, 2H), 7.16 (d, J = 2.8 Hz, 1H), 6.89 (dd, J = 8.9, 2.8 Hz, 1H), 6.46 (s, 1H), 3.75 (s, 3H), 2.39 (s, 3H). **^{13}C NMR** (150 MHz, $CDCl_3$) δ 157.96, 144.17, 136.09, 130.75, 129.70, 127.70, 125.50, 124.07, 115.28, 94.69, 55.80, 21.75. **HRMS** (ESI-TOF) Calcd for $C_{14}H_{15}INO_3S^+$ $[M+H]^+$ radical cation 402.9739, found 402.9725.

C-Nucleophile Synthesis

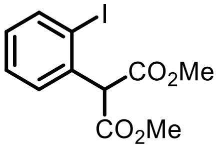

5a

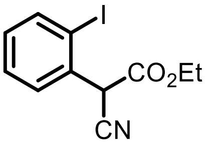

5b

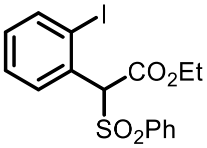

5c

C-Nucleophile **5a-c** were prepared according to literature procedures.[14, 24]

Reaction Optimization Details

Supplementary Table 3. Base optimization for *ortho*-iodophenol substrates.

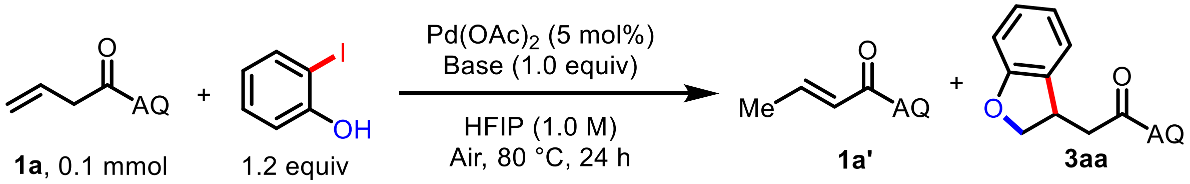

Entry	Base	**1a**[a]	**3aa**[a]
1	K_2CO_3	n.d.	95% (96%[b])
2	$KHCO_3$	n.d.	74%
3	Na_2CO_3	n.d.	64%
4	KF	55%	35%
5	K_3PO_4	n.d.	95%
6	KOH	12%	72%
7	KO^{t}Bu	n.d.	87%
8	KOAc	55%	22%
9	NEt_3	n.d. (54% **1a'**)	33%

[a] Yield determined by ^{1}H NMR analysis of the crude reaction mixture using CH_2Br_2 as an internal standard. [b] Isolated yield.

Supplementary Table 4. Solvent optimization for *ortho*-iodophenol substrates.

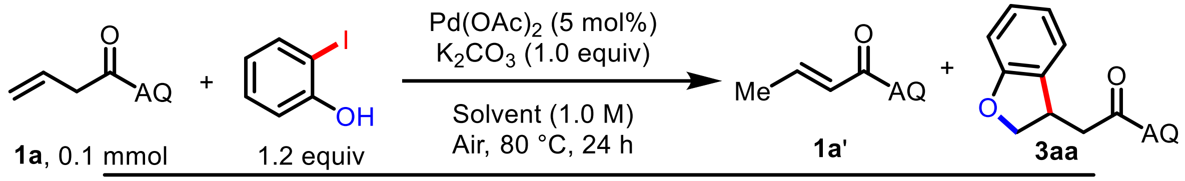

Entry	Solvent	**1a'**[a]	**3aa**[a]
1	HFIP	n.d.	95% (96%[b])
2	MeCN	83%	9%
3	t-AmylOH	60%	22%
1	^{i}PrOH	90%	9%
2	toluene	24%	71%
3	DMF	37%	trace
1	DCM	45%	50%
2	1,4-dioxane	76%	17%
3	THF	80%	4%

[a] Yield determined by ^{1}H NMR analysis of the crude reaction mixture using CH_2Br_2 as an internal standard. [b] Isolated yield.

Supplementary Table 5. Concentration Screening for *ortho*-iodophenol substrates.

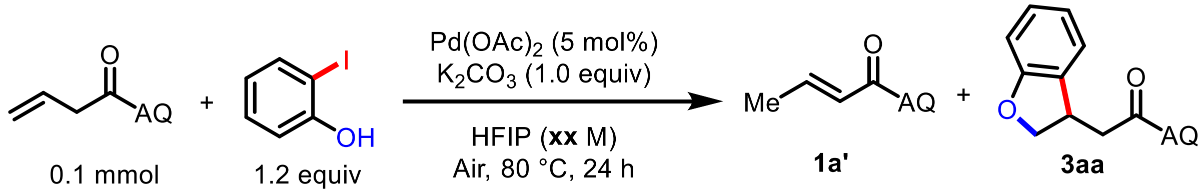

Entry	Concentration	**1a'**[a]	**3aa**[a]
1	HFIP (0.5 M)	n.d.	70%
2	HFIP (1.0 M)	n.d.	95% (96%[b])
3	Neat	54%	31%

[a] Yield determined by ^{1}H NMR analysis of the crude reaction mixture using CH_2Br_2 as an internal standard. [b] Isolated yield.

Supplementary Table 6. Concentration Screening for *ortho*-iodoaniline substrates.

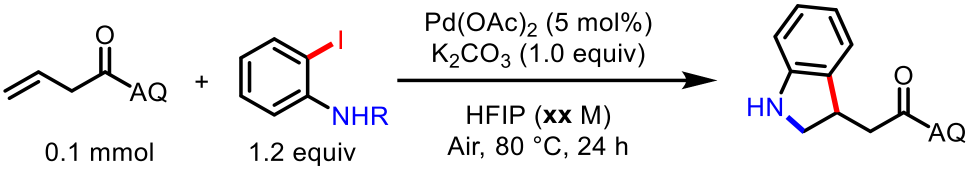

Entry	R	Concentration	Yield[a]
1	H	HFIP (0.5 M)	76% (75%[b])
2	H	HFIP (1.0 M)	69%
3	Me[c]	HFIP (0.5 M)	10%
4	Me[c]	HFIP (2.0 M)	47% (50%[b])
5	Bn[c]	HFIP (0.5 M)	10%
6	Bn[c]	HFIP (1.0 M)	33%
7	Bn[c]	HFIP (2.0 M)	55% (58%[b])

[a] Yield determined by ^{1}H NMR analysis of the crude reaction mixture using CH_2Br_2 as an internal standard. [b] Isolated yield. [c] $Pd(OAc)_2$ (10 mol%)

Supplementary Table 7. Temperature Screening for 2,3-dihydroindiene synthesis.

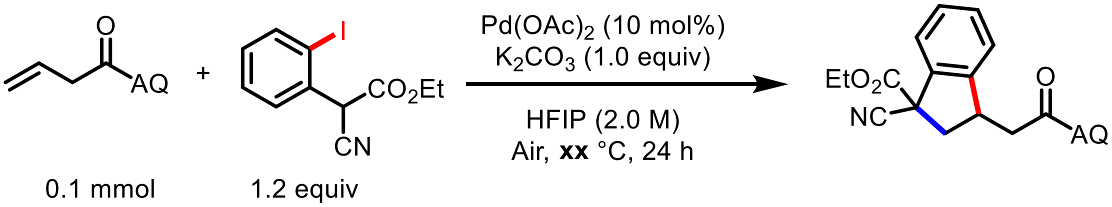

Entry	Temperature	Yield[a]
1	40 °C	90% (86%[b])
2	60 °C	79%
3	80 °C	65%

[a] Yield of mixture determined by ^{1}H NMR analysis of the crude reaction mixture using $Cl_2CHCHCl_2$ as an internal standard. [b] Isolated yield.

Mechanistic Studies

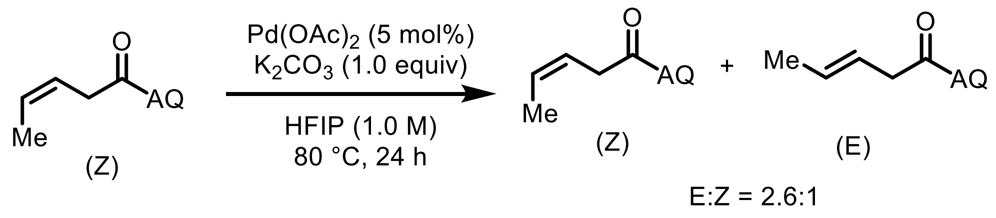

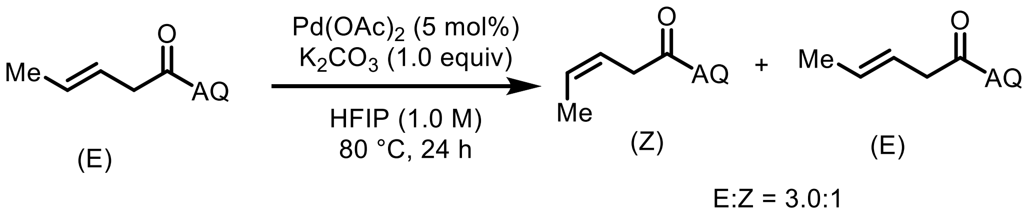

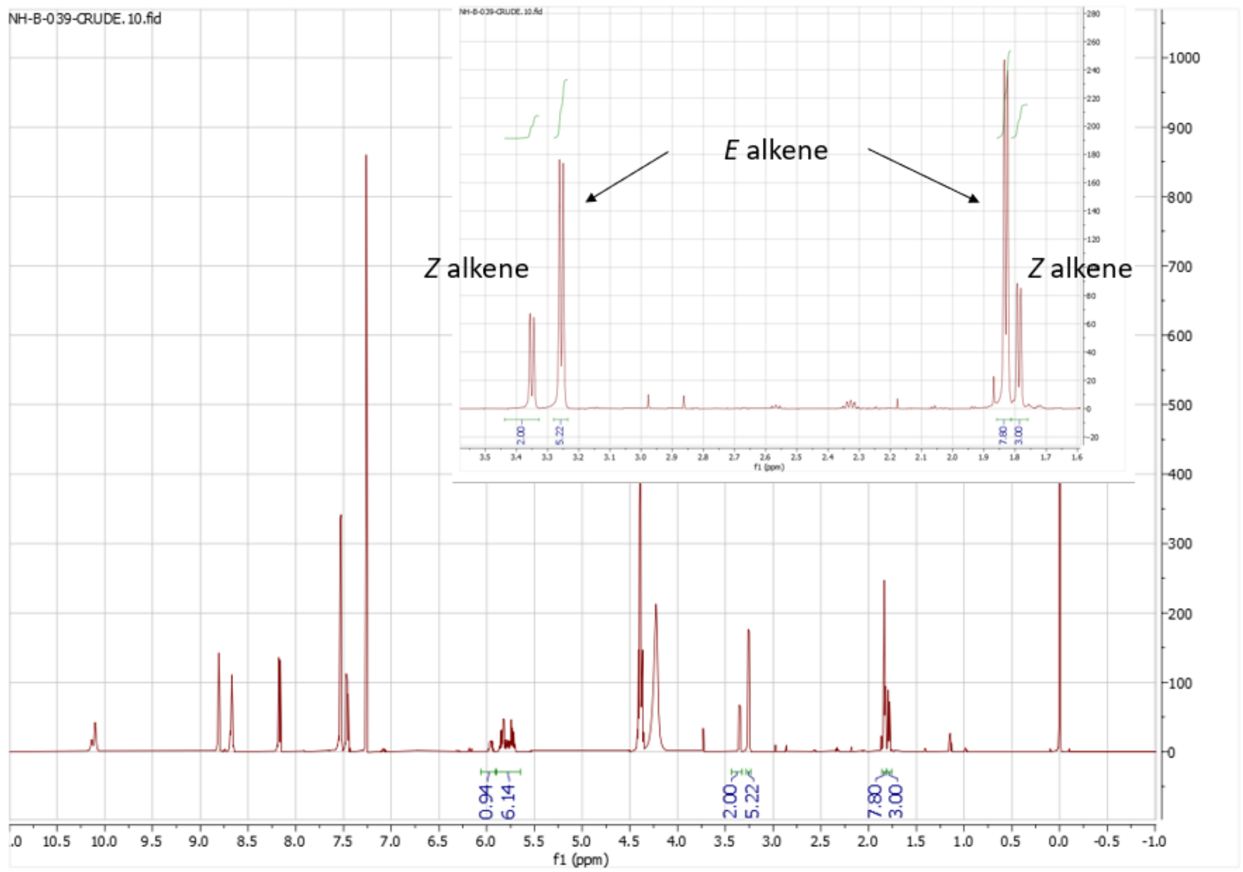

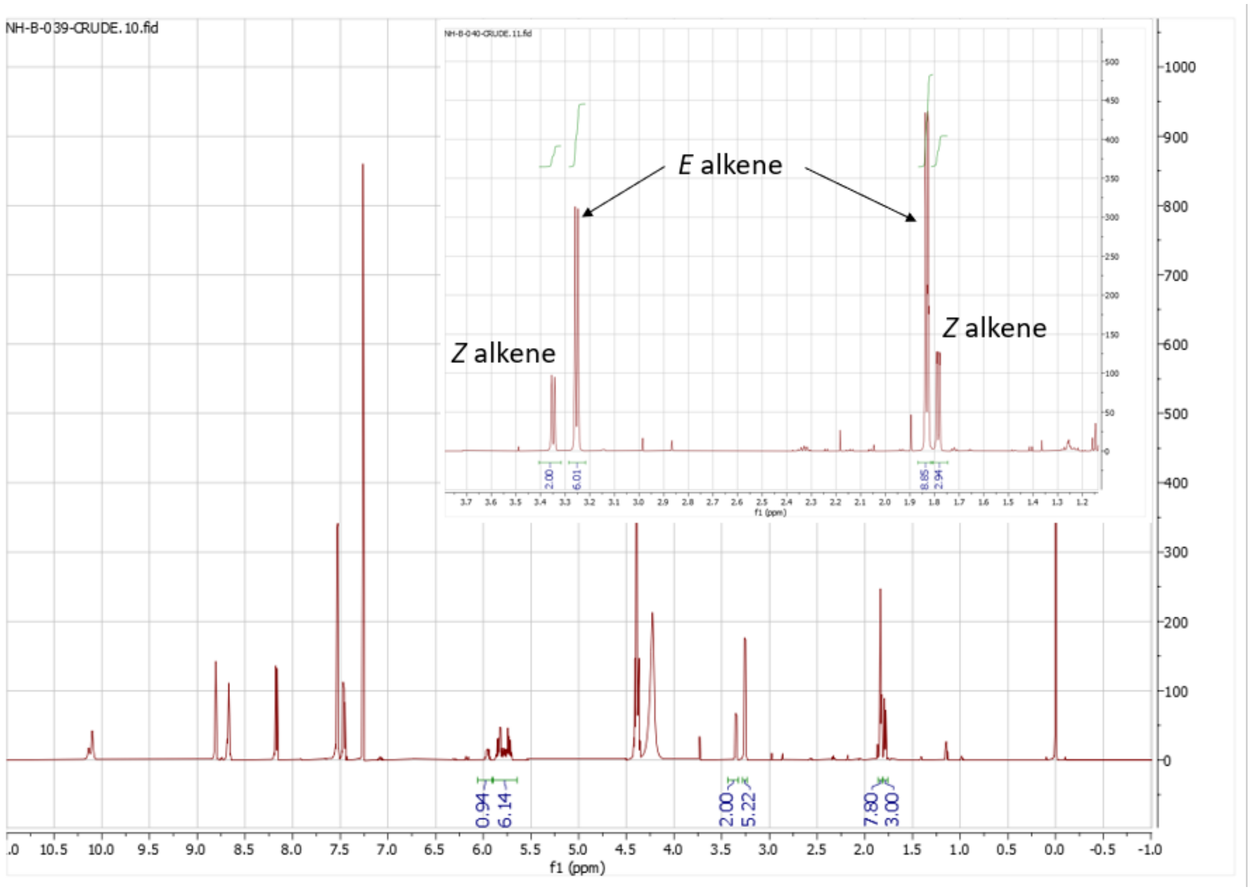

Supplementary Figure 5: Investigation of Alkene Substrate E/Z Isomerization.

To a 4-mL scintillation vial equipped with a Teflon-coated magnetic stir bar were added the $Pd(OAc)_2$ (1.1 mg, 0.005 mmol, 5 mol%), alkene substrate (24 mg, 0.1 mmol), K_2CO_3 (13.8 mg, 0.1 mmol, 1 equiv), and HFIP (0.1 mL). The vial was sealed with a screw-top septum cap and placed in a heating block that was pre-heated to 80 °C. The reaction was allowed to run for 24 h, and *E:Z* ratio was determined by ^{1}H NMR analysis of the crude reaction mixture.

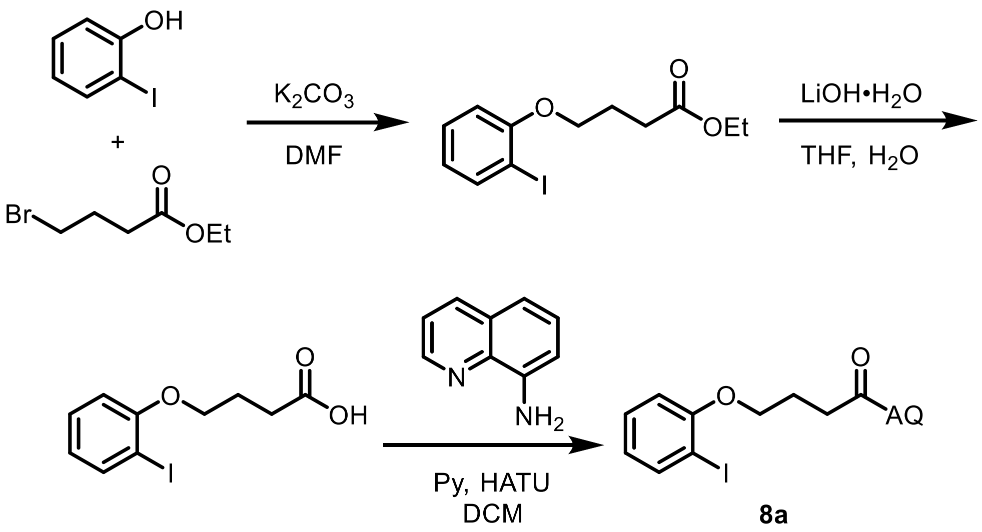

Supplementary Figure 6: Synthesis of 4-(2-iodophenoxy)-*N*-(quinolin-8-yl)butanamide (**8a**)

4-(2-iodophenoxy)-*N*-(quinolin-8-yl)butanamide (8a): The title compound was prepared using a procedure adapted from literature.[15] Ethyl 4-bromobutanoate (2.9 g, 15 mmol) and 2-iodophenol (2.2 g, 10 mmol) were charged into a 100-mL round-bottom flask. K_2CO_3 (6.9 g, 50 mmol, 5 equiv) and DMF (50 mL) were then added. The reaction was stirred at 60 °C for 12 h. The mixture was quenched with water, extracted with EtOAc (100 mL×1). The organic layers were dried over Na_2SO_4, filtered, and concentrated under reduced pressure. The crude product was carried forward to the next step without further purification. The crude mixture containing ethyl 4-(2-iodophenoxy)butanoate was charged into a 100-mL round-bottom flask containing THF (10 mL) and water (10 mL). Lithium hydroxide monohydrate (1.26 g, 30 mmol, 3 equiv) was added, and the reaction mixture was stirred at ambient temperature for 16 h. The organic layer was acidified with conc. HCl to pH=1 and extracted with EtOAc (50 mL, ×3). The combined organic layers were dried over Na_2SO_4 and concentrated under reduced pressure. The crude mixture containing 4-(2-iodophenoxy)butanoic acid was charged into a 100-mL round-botttom flask containing DCM (30 mL). 8-Aminoquinoline (1.44 g, 10 mmol, 1 equiv), pyridine (1.6 mL), and HATU (684 mg, 13 mmol, 1.3 equiv) were added sequentially, and the reaction mixture was stirred at ambient temperature for 16 h. The deep brown solution was diluted with EtOAc (200 mL), washed with sat. $NaHCO_3$ (200 mL ×1) and brine (200 mL ×1), dried over Na_2SO_4, filtered, concentrated under reduced pressure, and purified by column chromatography (hexane:EtOAc = 10:1) to afford **8a** (60% yield over 3 steps from 2-iodophenol) as a light yellow solid. **^{1}H NMR** (600 MHz, $CDCl_3$) δ 9.92 (s, 1H), 8.77 (dd, *J* = 7.5, 1.5 Hz, 1H), 8.72 (dd, *J* = 4.2, 1.7 Hz, 1H), 8.14 (dd, *J* = 8.3, 1.7 Hz, 1H), 7.77 (dd, *J* = 7.8, 1.6 Hz, 1H), 7.58–7.46 (m, 2H), 7.43 (dd, *J* = 8.3, 4.2 Hz, 1H), 7.29–7.23 (m, 1H), 6.82 (dd, *J* = 8.3, 1.3 Hz, 1H), 6.70 (td, *J* = 7.6, 1.4 Hz, 1H), 4.17 (t, *J* = 5.9 Hz, 2H), 2.91 (t, *J* = 7.2 Hz, 2H), 2.35 (tt, *J* = 7.2, 5.9 Hz, 2H). **^{13}C NMR** (150 MHz, $CDCl_3$) δ 171.21, 157.43, 148.26, 139.49, 138.47, 136.41, 134.65, 129.61, 128.06, 127.50, 122.70, 121.72, 121.59, 116.57, 112.36, 86.96, 67.95, 34.43, 25.19. **HRMS (ESI-TOF)** Calcd for $C_{19}H_{18}IN_2O_2^+$ $[M+H]^+$ 433.0413, found 433.0407.

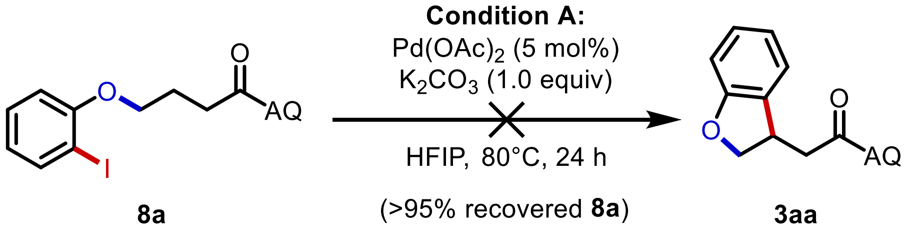

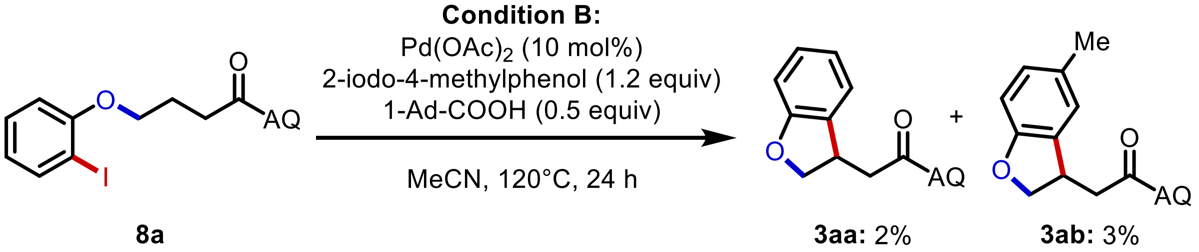

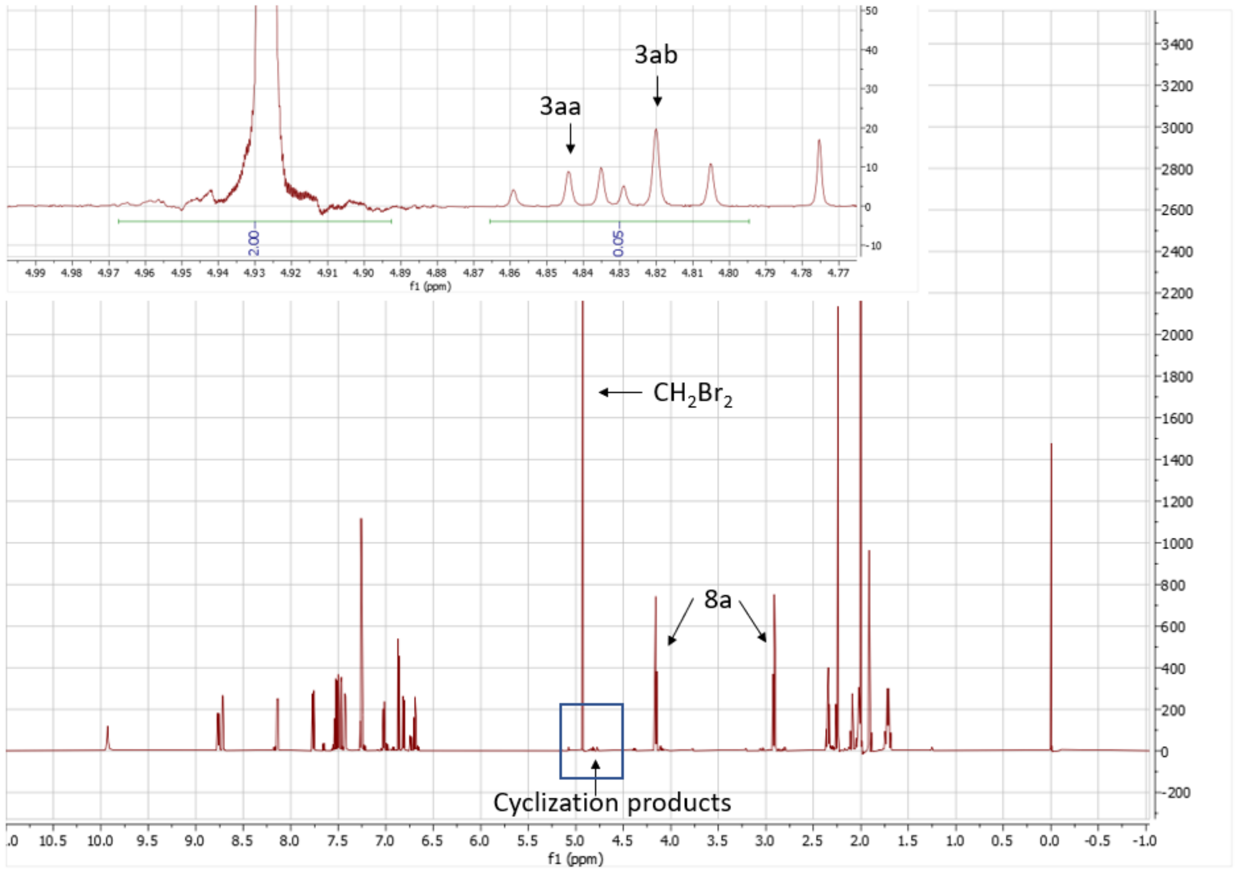

Supplementary Figure 7: Mechanistic Experiment A

Conditions A: **8a** (43.3 mg, 0.1 mmol), $Pd(OAc)_2$ (1.1 mg, 0.005 mmol, 5 mol%), K_2CO_3 (13.8 mg, 0.1 mmol, 1.0 equiv), and HFIP (0.1 mL) were added into a 4-mL scintillation vial equipped with a Teflon-coated magnetic stir bar. The vial was capped and placed in a heating block that was pre-heated to 80 °C. The reaction was allowed to run for 24 h, and yields were determined by ^1H NMR analysis of the crude reaction mixture using CH_2Br_2 as internal standard.

Conditions B: **8a** (43.3 mg, 0.1 mmol), $Pd(OAc)_2$ (2.2 mg, 0.010 mmol, 10 mol%), 2-iodo-4-methylphenol (28.1 mg, 0.12 mmol, 1.2 equiv), 1-Ad-COOH (9.0 mg, 0.05 mmol, 0.5 equiv), and MeCN (0.1 mL) were added into a 4-mL scintillation vial equipped with a Teflon-coated magnetic stir bar. The vial was capped and placed in a heating block that was pre-heated to 120 °C. The reaction was allowed to run for 24 h, and yields were determined by ^1H NMR analysis of the crude reaction mixture using CH_2Br_2 as internal standard.

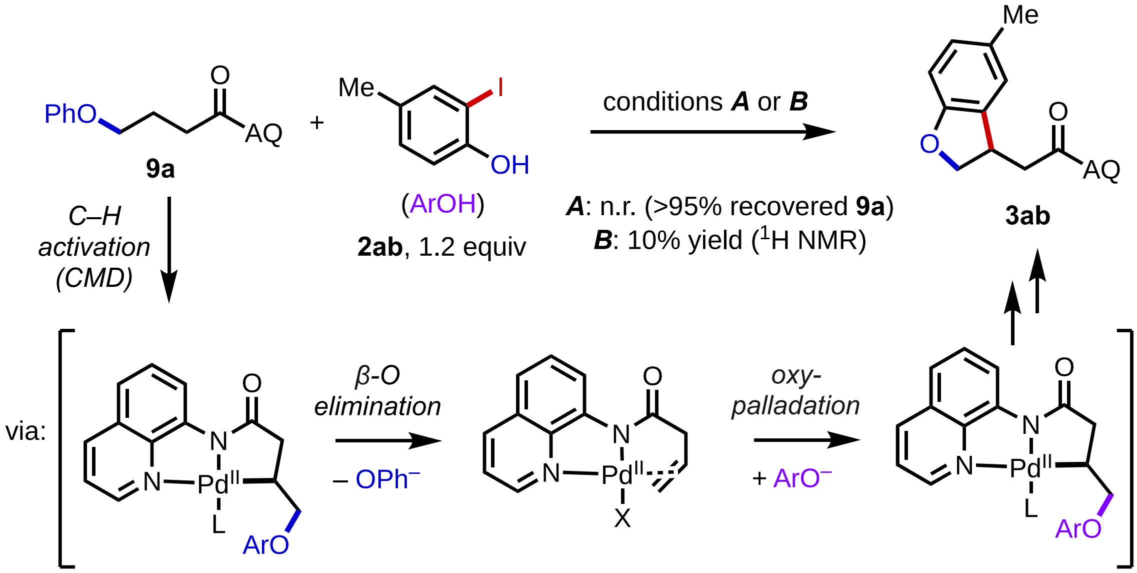

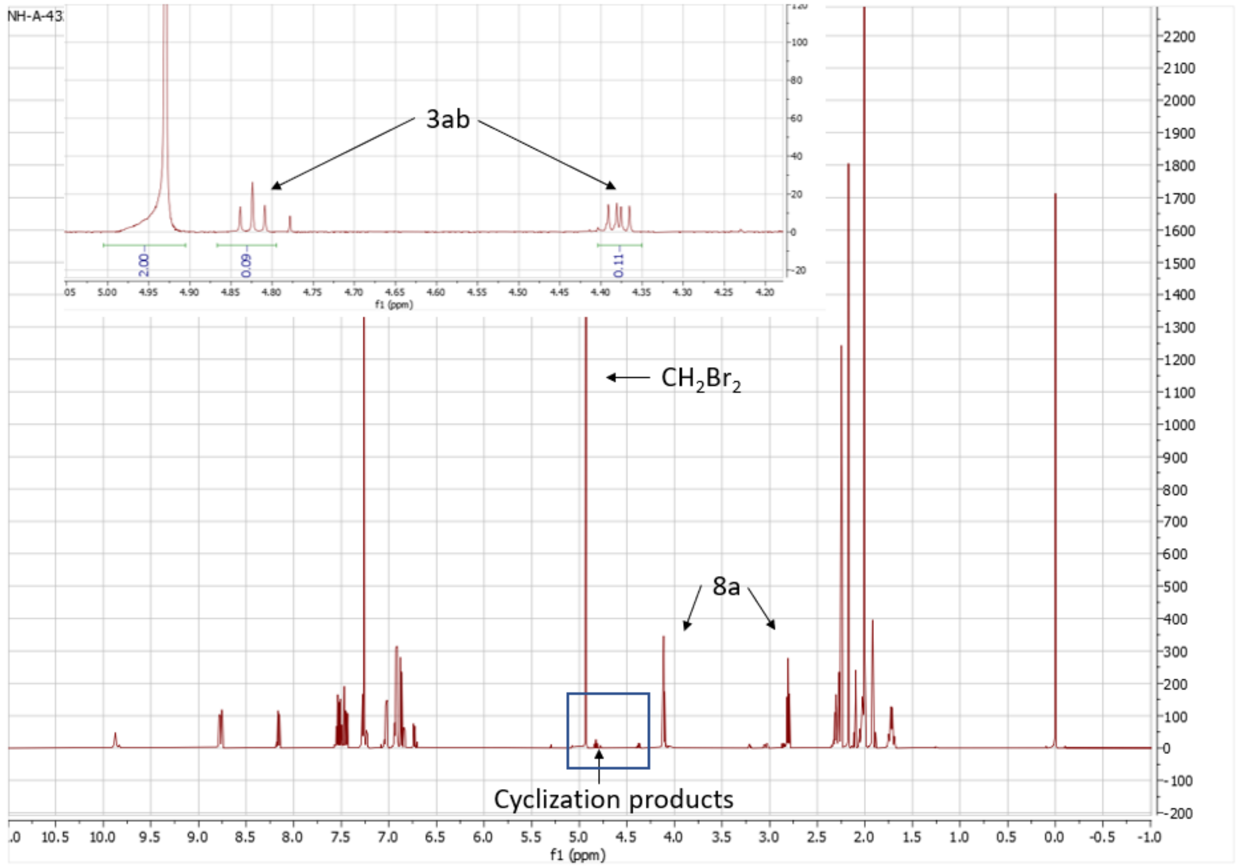

Supplementary Figure 8: Mechanistic Experiment B

Compound **9a** (30.6 mg, 0.1 mmol), $Pd(OAc)_2$ (4.5 mg, 20 mol% or 1.1 mg, 5 mol%, respectively), the appropriate iodophenol substrate (0.12 mmol), either 1-Ad-COOH (9 mg, 0.05 mmol, 0.5 equiv) or K_2CO_3 (13.8 mg, 0.1 mmol, 1.0 equiv), and solvent were added into a 4-mL scintillation vial equipped with a Teflon-coated magnetic stir bar. The vial was capped and placed in a heating block that was pre-heated to the appropriate temperature (120 °C or 80 °C, respectively). The reaction was allowed to run at elevated temperature for 24 h, and yields were determined by ^{1}H NMR analysis of the crude reaction mixture using CH_2Br_2 as internal standard.

Competition Study

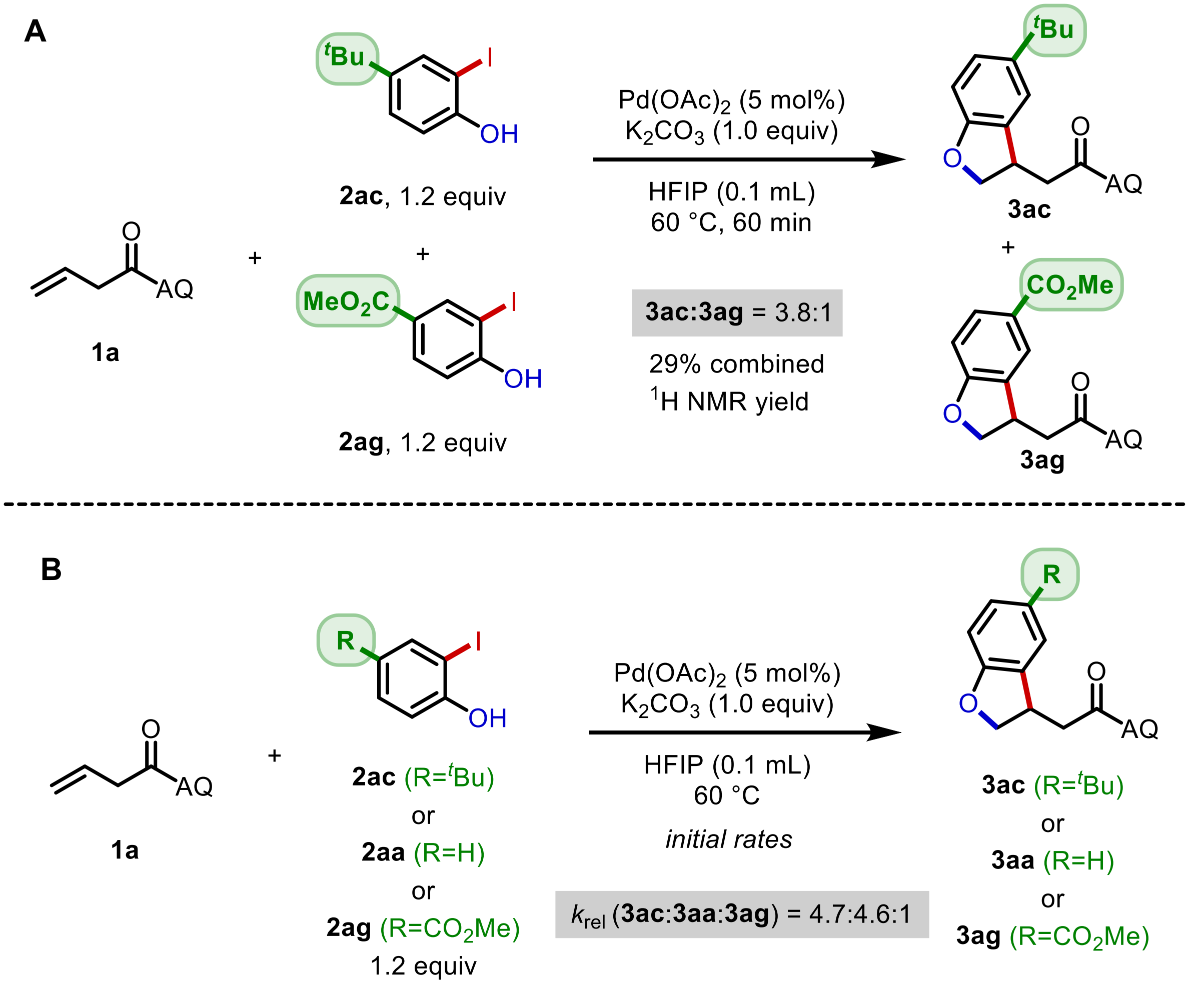

Supplementary Figure 9: (A) One-pot competition experiment; (B) Comparison of initial rates.

Study A: To a 4-mL scintillation vial equipped with a Teflon-coated magnetic stir bar were added the $Pd(OAc)_2$ (1.1 mg, 0.005 mmol, 5 mol%), alkene substrate (21.2 mg, 0.1 mmol), the appropriate *ortho*-iodophenol substrates (0.12 mmol), K_2CO_3 (13.8 mg, 0.1 mmol, 1 equiv), and HFIP (0.1 mL). The vial was sealed with a screw-top septum cap and placed in a heating block that was pre-heated to 60 °C. The reaction was allowed to run for 60 min, and yields were determined by ^1H NMR analysis of the crude reaction mixture using $Cl_2CHCHCl_2$ as internal standard.

Study B: To a 4-mL scintillation vial equipped with a Teflon-coated magnetic stir bar were added the $Pd(OAc)_2$ (1.1 mg, 0.005 mmol, 5 mol%), alkene substrate (21.2 mg, 0.1 mmol), the appropriate *ortho*-iodophenol substrate (0.12 mmol), K_2CO_3 (13.8 mg, 0.1 mmol, 1 equiv), and HFIP (0.1 mL). The vial was sealed with a screw-top septum cap and placed in a heating block that was pre-heated to 60 °C. The reaction was allowed to run for 30-120 min, and yields were determined by ^1H NMR analysis of the crude reaction mixture using $Cl_2CHCHCl_2$ as internal standard. (Multiple parallel reactions stopped at pre-determined time points.)

Supplementary Table 8: Initial rates for phenols **2aa**, **2ag**, and **2ac**

	30 min	60 min	90 min	120 min
**2aa**	14%	29%	40%	49%
**2ag**	3%	7%	8%	8%
**2ac**	18%	32%	45%	53%

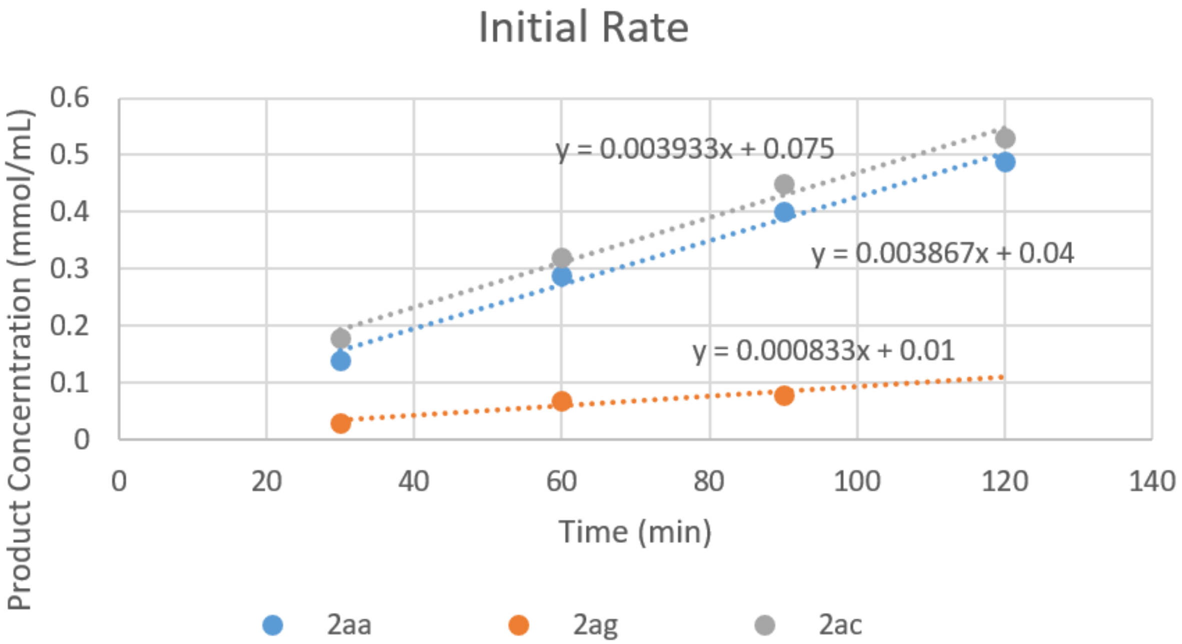

Supplementary Figure 10: Initial rates for **2aa**, **2ag**, and **2ac**.

General Procedure A for [3+2] 2,3-Dihydrobenzofuran Synthesis

To a 4-mL scintillation vial equipped with a Teflon-coated magnetic stir bar were added the $Pd(OAc)_2$ (1.1 mg, 0.005 mmol, 5 mol%), alkene substrate (21.2 mg, 0.1 mmol), the appropriate *ortho*-iodophenol (0.12 mmol, 1.2 equiv), K_2CO_3 (13.8 mg, 0.1 mmol, 1 equiv), and HFIP (0.1 mL). The vial was sealed with a screw-top septum cap and placed in a heating block that was pre-heated to 80 °C. The reaction was allowed to run for 24 h and then was cooled to room temperature. The reaction mixture was filtered through a short plug of Celite, which was washed with EtOAc. The filtrate was concentrated under reduced pressure to afford a brown residue, which, upon purification by preparative TLC, afforded the pure product.

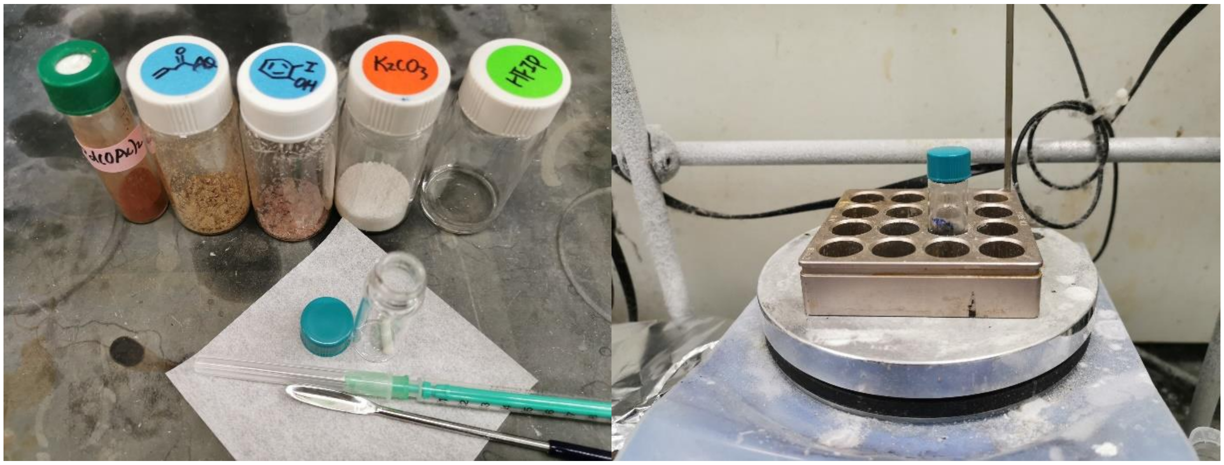

Supplementary Figure 11: Photographic depiction of the reaction procedure.

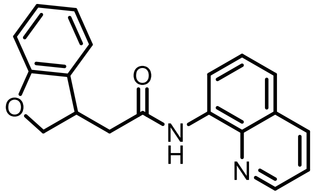

3aa

2-(2,3-dihydrobenzofuran-3-yl)-*N*-(quinolin-8-yl)acetamide (3aa): The title compound was prepared from **1a** (21.2 mg, 0.1 mmol) and 2-iodophenol (26.4 mg, 0.12 mmol) according to General Procedure A. Purification using preparative TLC with 3:1 hexanes:EtOAc as the eluent gave the product as a white solid (29.2 mg, 96% yield). **^{1}H NMR** (600 MHz, $CDCl_3$) δ 9.84 (s, 1H), 8.81–8.75 (m, 2H), 8.17 (dd, *J* = 8.2, 1.7 Hz, 1H), 7.58–7.54 (m, 1H), 7.53 (dd, *J* = 8.3, 1.6 Hz, 1H), 7.46 (dd, *J* = 8.2, 4.2 Hz, 1H), 7.26–7.23 (m, 1H), 7.17–7.12 (m, 1H), 6.86 (td, *J* = 7.4, 1.0 Hz, 1H), 6.83 (dd, *J* = 8.0, 0.9 Hz, 1H), 4.85 (t, *J* = 9.1 Hz, 1H), 4.40 (dd, *J* = 9.2, 6.1 Hz, 1H), 4.12 (tt, *J* = 9.1, 5.8 Hz, 1H), 3.05 (dd, *J* = 15.5, 5.6 Hz, 1H), 2.87 (dd, *J* = 15.5, 9.3 Hz, 1H). **^{13}C NMR** (150 MHz, $CDCl_3$) δ 169.59, 160.02, 148.37, 138.41, 136.54, 134.34, 129.61, 128.77, 128.10, 127.52, 124.55, 121.90, 121.85, 120.76, 116.68, 109.88, 77.00, 43.23, 38.78. **HRMS (ESI-TOF)** Calcd for $C_{19}H_{17}N_2O_2^+$ $[M+H]^+$ 305.1290, found 305.1287. **X-ray** (single-crystal) Colorless needle crystals of X-ray diffraction quality were obtained by vapor diffusion of hexane into a saturated solution of **3aa** in toluene (CCDC 2031197).[16]

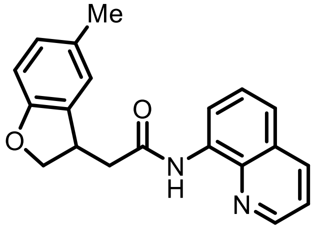

3ab

2-(5-methyl-2,3-dihydrobenzofuran-3-yl)-*N*-(quinolin-8-yl)acetamide (3ab): The title compound was prepared from **1a** (21.2 mg, 0.1 mmol) and 2-iodo-4-methylphenol (28.1 mg, 0.12 mmol) according to the General Procedure A. Purification using preparative TLC with 3:1 hexanes:EtOAc as the eluent gave the product as a white solid (27.0 mg, 85% yield). **^{1}H NMR** (600 MHz, $CDCl_3$) δ 9.83 (s, 1H), 8.81–8.74 (m, 2H), 8.17 (dd, *J* = 8.3, 1.7 Hz, 1H), 7.58–7.55 (m, 1H), 7.53 (dd, *J* = 8.3, 1.6 Hz, 1H), 7.46 (dd, *J* = 8.2, 4.2 Hz, 1H), 7.07–7.03 (m, 1H), 6.97–6.92 (m, 1H), 6.72 (d, *J* = 8.1 Hz, 1H), 4.83 (t, *J* = 9.0 Hz, 1H), 4.38 (dd, *J* = 9.2, 6.1 Hz, 1H), 4.06 (tt, *J* = 9.1, 5.7 Hz, 1H), 3.05 (dd, *J* = 15.4, 5.4 Hz, 1H), 2.85 (dd, *J* = 15.4, 9.3 Hz, 1H), 2.25(s, 3H). **^{13}C NMR** (150 MHz, $CDCl_3$) δ 169.67, 157.90, 148.35, 138.41, 136.53, 134.35, 130.04, 129.60, 129.12, 128.09, 127.52, 125.07, 121.87, 121.83, 116.67, 109.38, 77.11, 43.20, 38.88, 20.92. **HRMS (ESI-TOF)** Calcd for $C_{20}H_{19}N_2O_2^+$ $[M+H]^+$ 319.1447, found 319.1448.

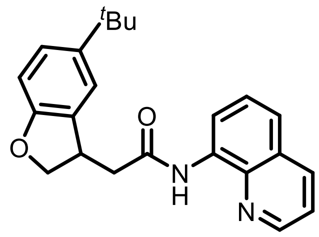

3ac

2-(5-(tert-butyl)-2,3-dihydrobenzofuran-3-yl)-*N*-(quinolin-8-yl)acetamide (3ac): The title compound was prepared from **1a** (21.2 mg, 0.1 mmol) and 4-(tert-butyl)-2-iodophenol (33.1 mg, 0.12 mmol) according to the General Procedure A. Purification using preparative TLC with 3:1 hexanes:EtOAc as the eluent gave the product as a white solid (35.6 mg, 99% yield). **^{1}H NMR** (600 MHz, $CDCl_3$) δ 9.84 (s, 1H), 8.80 (dd, *J* = 7.4, 1.5 Hz, 1H), 8.77 (dd, *J* = 4.2, 1.7 Hz, 1H), 8.17 (dd, *J* = 8.3, 1.7 Hz, 1H), 7.59–7.51 (m, 2H), 7.45 (dd, *J* = 8.3, 4.2 Hz, 1H), 7.27–7.25 (m, 1H), 7.17 (ddd, *J* = 8.4, 2.2, 0.7 Hz, 1H), 6.76 (d, *J* = 8.4 Hz, 1H), 4.85 (t, *J* = 9.0 Hz, 1H), 4.43–4.37 (m, 1H), 4.12–4.04 (m, 1H), 3.06 (dd, *J* = 15.3, 5.5 Hz, 1H), 2.87 (dd, *J* = 15.3, 9.4 Hz, 1H), 1.22 (s, 9H). **^{13}C NMR** (150 MHz, $CDCl_3$) δ 169.75, 157.76, 148.33, 143.81, 138.41, 136.52, 134.35, 129.11, 128.08, 127.52, 125.60, 121.87, 121.81, 121.38, 116.66, 109.04, 77.25, 43.37, 39.17, 34.42, 31.77. **HRMS (ESI-TOF)** Calcd for $C_{23}H_{25}N_2O_2^+$ $[M+H]^+$ 361.1916, found 361.1914.

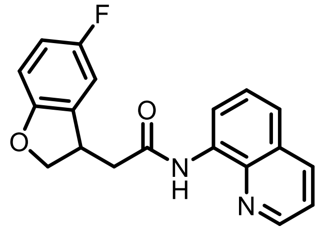

3ad

2-(5-fluoro-2,3-dihydrobenzofuran-3-yl)-*N*-(quinolin-8-yl)acetamide (3ad): The title compound was prepared from **1a** (21.2 mg, 0.1 mmol) and 4-fluoro-2-iodophenol (28.6 mg, 0.12 mmol) according to the General Procedure A. Purification using preparative TLC with 3:1 hexanes:EtOAc as the eluent gave the product as a white solid (28.7 mg, 89% yield). **^{1}H NMR** (600 MHz, $CDCl_3$) δ 9.84 (s, 1H), 8.80–8.75 (m, 2H), 8.17 (dd, *J* = 8.2, 1.7 Hz, 1H), 7.59–7.51 (m, 2H), 7.46 (dd, *J* = 8.2, 4.2 Hz, 1H), 6.99–6.94 (m, 1H), 6.86–6.79 (m, 1H), 6.74–6.69 (m, 1H), 4.86 (t, *J* = 9.1 Hz, 1H), 4.41 (dd, *J* = 9.2, 6.2 Hz, 1H), 4.14–4.06 (m, 1H), 3.02 (dd, *J* = 15.6, 5.8 Hz, 1H), 2.88 (dd, *J* = 15.5, 9.0 Hz, 1H). **^{13}C NMR** (150 MHz, $CDCl_3$) δ 169.17, 158.42 (d, *J* = 367.2 Hz), 148.39, 138.39, 136.57, 134.24, 131.08 (d, *J* = 8.3 Hz), 128.10, 127.51, 121.99, 121.87, 116.71, 114.92 (d, *J* = 24.3 Hz), 111.77 (d, *J* = 24.9 Hz), 109.99 (d, *J* = 8.3 Hz), 77.56, 42.85, 39.04 (d, *J* = 2.1 Hz). **^{19}F NMR** (376 MHz, $CDCl_3$) δ -124.30. **HRMS (ESI-TOF)** Calcd for $C_{19}H_{16}FN_2O_2^+$ [M+H]+ 323.1196, found 323.1198.

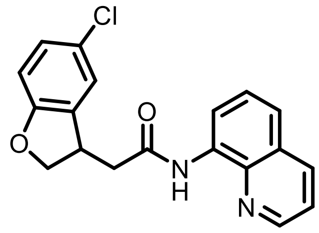

3ae

2-(5-chloro-2,3-dihydrobenzofuran-3-yl)-*N*-(quinolin-8-yl)acetamide (3ae): The title compound was prepared from **1a** (21.2 mg, 0.1 mmol) and 4-chloro-2-iodophenol (35.6 mg, 0.12 mmol) according to the General Procedure A. Purification using preparative TLC with 3:1 hexanes:EtOAc as the eluent gave the product as a white solid (25.4 mg, 75% yield). **^{1}H NMR** (600 MHz, $CDCl_3$) δ 9.84 (s, 1H), 8.80–8.74 (m, 2H), 8.17 (dd, J = 8.3, 1.7 Hz, 1H), 7.59–7.52 (m, 2H), 7.46 (dd, *J* = 8.3, 4.2 Hz, 1H), 7.22 (dd, *J* = 2.3, 1.0 Hz, 1H), 7.10 (ddd, *J* = 8.5, 2.3, 0.8 Hz, 1H), 6.73 (d, *J* = 8.5 Hz, 1H), 4.87 (t, *J* = 9.1 Hz, 1H), 4.42 (dd, *J* = 9.3, 6.2 Hz, 1H), 4.13–4.05 (m, 1H), 3.04 (dd, *J* = 15.7, 5.5 Hz, 1H), 2.87 (dd, *J* = 15.6, 9.3 Hz, 1H). **^{13}C NMR** (150 MHz, $CDCl_3$) δ 169.12, 158.77, 148.40, 138.39, 136.55, 134.23, 131.61, 128.66, 128.09, 127.50, 125.38, 124.77, 121.99, 121.87, 116.70, 110.81, 77.60, 42.87, 38.77. **HRMS (ESI-TOF)** Calcd for $C_{19}H_{16}ClN_2O_2^+$ $[M+H]^+$ 339.0900, found 339.0894.

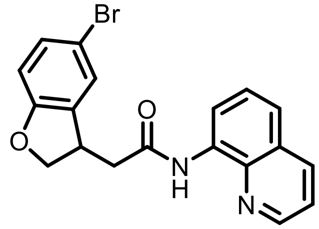

3af

2-(5-bromo-2,3-dihydrobenzofuran-3-yl)-*N*-(quinolin-8-yl)acetamide (3af): The title compound was prepared from **1a** (21.2 mg, 0.1 mmol) and 4-bromo-2-iodophenol (35.8 mg, 0.12 mmol) according to the General Procedure A. Purification using preparative TLC with 3:1 hexanes:EtOAc as the eluent as the eluent gave the product as a white solid (32.1 mg, 84% yield). **^{1}H NMR** (600 MHz, $CDCl_3$) δ 9.84 (s, 1H), 8.81–8.73 (m, 2H), 8.17 (dd, *J* = 8.2, 1.7 Hz, 1H), 7.59–7.52 (m, 2H), 7.46 (dd, *J* = 8.2, 4.2 Hz, 1H), 7.35 (dd, *J* = 2.1, 1.0 Hz, 1H), 7.24 (ddd, *J* = 8.5, 2.2, 0.7 Hz, 1H), 6.69 (d, *J* = 8.5 Hz, 1H), 4.87 (t, *J* = 9.1 Hz, 1H), 4.42 (dd, *J* = 9.3, 6.2 Hz, 1H), 4.13–4.05 (m, 1H), 3.04 (dd, *J* = 15.6, 5.4 Hz, 1H), 2.87 (dd, *J* = 15.6, 9.3 Hz, 1H). **^{13}C NMR** (150 MHz, $CDCl_3$) δ 169.11, 159.29, 148.41, 138.39, 136.55, 134.22, 132.16, 131.57, 128.10, 127.63, 127.50, 121.99, 121.87, 116.70, 112.44, 111.46, 77.58, 42.89, 38.72. **HRMS (ESI-TOF)** Calcd for $C_{19}H_{16}BrN_2O_2^+$ $[M+H]^+$ 383.0395, found 383.0385.

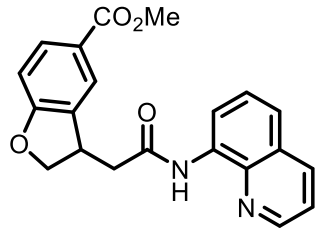

3ag

Methyl 3-(2-oxo-2-(quinolin-8-ylamino)ethyl)-2,3-dihydrobenzofuran-5-carboxylate (3ag): The title compound was prepared from **1a** (21.2 mg, 0.1 mmol) and methyl 4-hydroxy-3-iodobenzoate (33.4 mg, 0.12 mmol) according to the General Procedure A. Purification using preparative TLC with 3:1 hexanes:EtOAc as the eluent gave the product as a white solid (17.7 mg, 49% yield). **^{1}H NMR** (600 MHz, $CDCl_3$) δ 9.84 (s, 1H), 8.87 –8.68 (m, 2H), 8.16 (d, *J* = 8.2 Hz, 1H), 7.94–7.84 (m, 2H), 7.58–7.48 (m, 2H), 7.45 (dd, *J* = 8.3, 4.2 Hz, 1H), 6.82 (d, *J* = 8.4 Hz, 1H), 4.96 (t, *J* = 9.2 Hz, 1H), 4.51 (dd, *J* = 9.5, 6.3 Hz, 1H), 4.15–4.07 (m, 1H), 3.84 (s, 3H), 3.13 (dd, *J* = 15.7, 4.8 Hz, 1H), 2.88 (dd, *J* = 15.7, 10.0 Hz, 1H). **^{13}C NMR** (150 MHz, $CDCl_3$) δ 169.09, 166.83, 164.08, 148.25, 138.25, 136.40, 134.10, 131.68, 129.90, 127.95, 127.36, 126.26, 122.88, 121.84, 121.73, 116.57, 109.49, 78.11, 51.84, 42.85, 38.06. **HRMS (ESI-TOF)** Calcd for $C_{21}H_{19}N_2O_4^+$ $[M+H]^+$ 363.1345, found 363.1343.

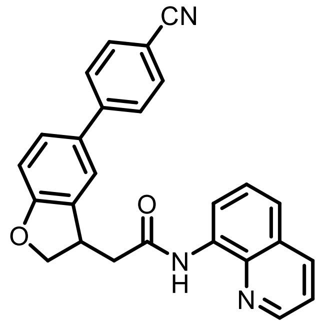

3ah

2-(5-(4-cyanophenyl)-2,3-dihydrobenzofuran-3-yl)-*N*-(quinolin-8-yl)acetamide (3ah): The title compound was prepared from **1a** (21.2 mg, 0.1 mmol) and 4'-hydroxy-3'-iodo-[1,1'-biphenyl]-4-carbonitrile (38.5 mg, 0.12 mmol) according to the General Procedure A. Purification using preparative TLC with 3:1 hexanes:EtOAc as the eluent as the eluent gave the product as a white solid (17.0 mg, 42% yield). **^{1}H NMR** (600 MHz, $CDCl_3$) δ 9.85 (s, 1H), 8.79 (dd, *J* = 7.2, 1.8 Hz, 1H), 8.72 (dd, *J* = 4.2, 1.7 Hz, 1H), 8.17 (dd, *J* = 8.3, 1.7 Hz, 1H), 7.60–7.54 (m, 4H), 7.52–7.48 (m, 2H), 7.48–7.46 (m, 1H), 7.45 (dd, *J* = 8.2, 4.2 Hz, 1H), 7.38 (ddd, *J* = 8.3, 2.1, 0.7 Hz, 1H), 6.91 (d, *J* = 8.3 Hz, 1H), 4.93 (t, *J* = 9.1 Hz, 1H), 4.48 (dd, J = 9.3, 6.0 Hz, 1H), 4.17 (tt, *J* = 8.8, 6.1 Hz, 1H), 3.07 (dd, *J* = 15.3, 6.2 Hz, 1H), 2.94 (dd, *J* = 15.3, 8.6 Hz, 1H). **^{13}C NMR** (150 MHz, $CDCl_3$) δ 169.29, 160.88, 148.38, 145.67, 138.36, 136.58, 134.20, 132.61, 132.12, 130.91, 128.23, 128.11, 127.53, 127.32, 123.68, 122.06, 121.89, 119.24, 116.75, 110.43, 110.07, 77.67, 43.28, 38.76. **HRMS (ESI-TOF)** Calcd for $C_{26}H_{20}N_3O_2^+$ $[M+H]^+$ 406.1556, found 406.1545.

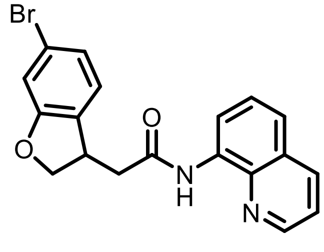

3ai

2-(6-bromo-2,3-dihydrobenzofuran-3-yl)-*N*-(quinolin-8-yl)acetamide (3ai): The title compound was prepared from **1a** (21.2 mg, 0.1 mmol) and 5-bromo-2-iodophenol (35.8 mg, 0.12 mmol) according to the General Procedure A. Purification using preparative TLC with 3:1 hexanes:EtOAc as the eluent as the eluent gave the product as a white solid (24.8 mg, 65% yield). **^{1}H NMR** (600 MHz, $CDCl_3$) δ 9.83 (s, 1H), 8.87–8.74 (m, 2H), 8.17 (dd, *J* = 8.2, 1.7 Hz, 1H), 7.59–7.49 (m, 2H), 7.46 (dd, *J* = 8.3, 4.2 Hz, 1H), 7.10 (dt, *J* = 7.4, 1.0 Hz, 1H), 7.00–6.95 (m, 2H), 4.86 (t, *J* = 9.1 Hz, 1H), 4.42 (dd, *J* = 9.3, 6.1 Hz, 1H), 4.06 (dddd, *J* = 9.0, 8.0, 6.2, 5.2 Hz, 1H), 3.01 (dd, *J* = 15.5, 5.9 Hz, 1H), 2.87 (dd, *J* = 15.5, 9.0 Hz, 1H). **^{13}C NMR** (150 MHz, $CDCl_3$) δ 169.20, 161.11, 148.40, 138.38, 136.57, 134.24, 129.00, 128.11, 127.50, 125.62, 123.79, 122.00, 121.89, 121.80, 116.70, 113.52, 77.77, 43.00, 38.32. **HRMS (ESI-TOF)** Calcd for $C_{19}H_{16}BrN_2O_2^+$ $[M+H]^+$ 383.0395, found 383.0385

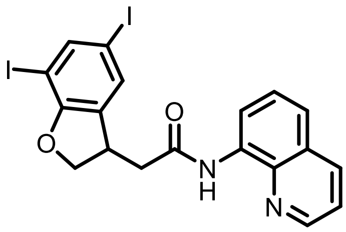

3aj

2-(5,7-diiodo-2,3-dihydrobenzofuran-3-yl)-N-(quinolin-8-yl)acetamide (3aj): The title compound was prepared from **1a** (21.2 mg, 0.1 mmol) and 2,4,6-triiodophenol (56.5 mg, 0.12 mmol) according to the General Procedure A. Purification using preparative TLC with 3:1 hexanes:EtOAc as the eluent as the eluent gave the product as a white solid (17 mg, 30% yield). **^{1}H NMR** (600 MHz, $CDCl_3$) δ 9.84 (s, 1H), 8.79 (dd, *J* = 4.2, 1.7 Hz, 1H), 8.74 (dd, *J* = 6.8, 2.2 Hz, 1H), 8.20–8.13 (m, 1H), 7.82–7.74 (m, 1H), 7.60–7.50 (m, 2H), 7.50–7.42 (m, 2H), 4.94 (t, *J* = 9.2 Hz, 1H), 4.49 (dd, *J* = 9.5, 6.3 Hz, 1H), 4.21 (tt, *J* = 9.3, 5.8 Hz, 1H), 3.03 (dd, *J* = 15.7, 5.4 Hz, 1H), 2.90 (dd, *J* = 15.7, 9.4 Hz, 1H). **^{13}C NMR** (151 MHz, $CDCl_3$) δ 168.75, 160.67, 148.46, 144.72, 138.38, 136.59, 134.12, 133.41, 132.1, 128.11, 127.5, 122.10, 121.92, 116.74, 82.51, 77.28, 75.5, 42.90, 39.8. **HRMS (ESI-TOF)** Calcd for $C_{19}H_{14}I_2N_2O_2^+$ $[M+H]^+$ 556.9223, found 556.9223.

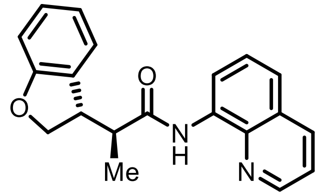

(±)-4ba
d.r. = 12:1

2-(2,3-dihydrobenzofuran-3-yl)-*N*-(quinolin-8-yl)acetamide ((±)-4ba): The title compound was prepared from **1b** (22.6 mg, 0.1 mmol) and 2-iodophenol (26.4 mg, 0.12 mmol) according to the General Procedure A. Purification using preparative TLC with with 3:1 hexanes:EtOAc as the eluent as the eluent gave the product as a colorless oil (29.2 mg, 96% yield). The reported *d.r.* was determined by ^{1}H NMR analysis of purified **(±)-4ba** and is consistent with that of the crude reaction mixture. The following analytical data correspond to the mixture. **^{1}H NMR** (500 MHz, $CDCl_3$) δ 9.89 (s, 1.08H), 8.86–8.79 (m, 1.08H), 8.77 (m, *J* = 4.2, 1.7 Hz, 1.08H), 8.19–8.14 (m, 1.08H), 7.60–7.49 (m, 2.16H), 7.45 (dd, *J* = 8.3, 4.2 Hz, 1.08H), 7.25–7.18 (m, 1.08H), 7.18–7.08 (m, 1.08H), 6.96–6.83 (m, 0.16H), 6.83–6.73 (m, 2H), 4.69 (t, *J* = 9.3 Hz, 1.08H), 4.62 (dd, *J* = 9.5, 5.7 Hz, 1H), 4.49 (dd, *J* = 9.4, 3.9 Hz, 0.08H), 4.01 (m, *J* = 9.1, 6.2 Hz, 1H), 3.90–3.78 (m, 0.08H), 2.94 (p, *J* = 7.0 Hz, 1H), 2.88–2.83 (p, 0.08H), 1.44 (d, *J* = 7.0 Hz, 0.24H), 1.32 (d, *J* = 7.0 Hz, 3H). **^{13}C NMR** (126 MHz, $CDCl_3$) δ 173.64, 160.37, 148.35, 138.55, 136.51, 134.37, 128.79, 128.46, 128.09, 127.49, 124.90, 121.89, 121.81, 120.69, 116.73, 109.73, 73.60, 46.45, 44.70, 14.47. **HRMS (ESI-TOF)** Calcd for $C_{20}H_{19}N_2O_2$+ [M+H]+ 319.1447, found 319.1448.

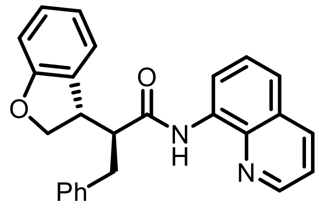

(±)-4ca
d.r. > 20:1

2-(2,3-dihydrobenzofuran-3-yl)-3-phenyl-*N*-(quinolin-8-yl)propanamide ((±)-4ca): The title compound was prepared from **1c** (30.2 mg, 0.1 mmol) and 2-iodophenol (26.4 mg, 0.12 mmol) according to the General Procedure A. Purification using preparative TLC with 3:1 hexanes:EtOAc as the eluent gave the product as a colorless oil (33.9 mg, 86% yield). **^{1}H NMR** (600 MHz, $CDCl_3$) δ 9.43 (s, 1H), 8.77 (dd, *J* = 7.5, 1.4 Hz, 1H), 8.60 (dd, *J* = 4.2, 1.6 Hz, 1H), 8.09 (dd, *J* = 8.2, 1.7 Hz, 1H), 7.52 (t, *J* = 7.9 Hz, 1H), 7.50–7.46 (m, 1H), 7.37 (dd, *J* = 8.2, 4.2 Hz, 1H), 7.26–7.23 (m, 1H), 7.20–7.16 (m, 2H), 7.16–7.13 (m, 2H), 7.13–7.08 (m, 1H), 7.06 (d, *J* = 7.2 Hz, 1H), 6.85–6.80 (m, 1H), 6.75 (td, *J* = 7.5, 1.0 Hz, 1H), 4.78 (dd, J = 9.5, 5.7 Hz, 1H), 4.72 (t, *J* = 9.2 Hz, 1H), 4.05 (dt, *J* = 9.0, 6.3 Hz, 1H), 3.17 (dd, *J* = 13.5, 10.9 Hz, 1H), 3.07–3.02 (m, 1H), 2.81 (dd, *J* = 13.6, 3.3 Hz, 1H). **^{13}C NMR** (150 MHz, $CDCl_3$) δ 172.12, 160.40, 148.06, 139.14, 138.37, 136.24, 134.10, 128.97, 128.95, 128.69, 128.10, 127.91, 127.38, 126.54, 125.04, 121.83, 121.64, 120.82, 116.65, 109.86, 73.98, 55.16, 44.82, 35.81. **HRMS (ESI-TOF)** Calcd for $C_{26}H_{23}N_2O_2^+$ $[M+H]^+$ 395.1760, found 395.1758.

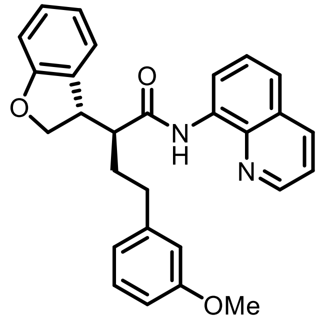

(±)-4da
d.r. > 20:1

2-(2,3-dihydrobenzofuran-3-yl)-4-(3-methoxyphenyl)-*N*-(quinolin-8-yl)butanamide ((±)-4da): The title compound was prepared from **1d** (34.6 mg, 0.1 mmol) and 2-iodophenol (26.4 mg, 0.12 mmol) according to the General Procedure A. Purification using preparative TLC with 3:1 hexanes:EtOAc as the eluent gave the product as a colorless oil (39.4 mg, 90% yield). **^{1}H NMR** (500 MHz, $CDCl_3$) δ 9.82 (s, 1H), 8.89 (dd, *J* = 7.3, 1.6 Hz, 1H), 8.80–8.75 (m, 1H), 8.18 (dd, *J* = 8.3, 1.7 Hz, 1H), 7.63–7.51 (m, 2H), 7.46 (dd, *J* = 8.2, 4.2 Hz, 1H), 7.17 (t, *J* = 7.8 Hz, 1H), 7.11–7.04 (m, 2H), 6.80–6.74 (m, 2H), 6.74–6.66 (m, 3H), 4.69–4.62 (m, 1H), 4.59 (dd, *J* = 9.3, 6.1 Hz, 1H), 3.93 (q, *J* = 7.6 Hz, 1H), 3.72 (s, 3H), 2.86–2.77 (m, 1H), 2.75–2.68 (m, 1H), 2.58 (dt, *J* = 13.9, 8.3 Hz, 1H), 2.33–2.24 (m, 1H), 1.84–1.75 (m, 1H). **^{13}C NMR** (126 MHz, $CDCl_3$) δ 172.78, 160.25, 159.88, 148.39, 142.70, 138.58, 136.46, 134.20, 129.62, 128.81, 128.11, 128.06, 127.52, 125.05, 122.06, 121.85, 121.04, 120.67, 116.86, 114.30, 111.72, 109.75, 74.04, 55.22, 51.76, 44.80, 33.67, 31.31. **HRMS (ESI-TOF)** Calcd for $C_{28}H_{27}N_2O_3^+$ $[M+H]^+$ 439.2022, found 439.2017.

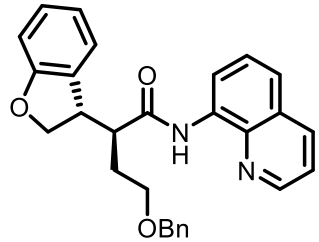

(±)-4ea
d.r. > 20:1

4-(benzyloxy)-2-(2,3-dihydrobenzofuran-3-yl)-*N*-(quinolin-8-yl)butanamide ((±)-4ea): The title compound was prepared from **1e** (34.6 mg, 0.1 mmol) and 2-iodophenol (26.4 mg, 0.12 mmol) according to the General Procedure A. Purification using preparative TLC with 3:1 hexanes:EtOAc as the eluent gave the product as a colorless oil (33.3 mg, 76% yield). **^{1}H NMR** (600 MHz, $CDCl_3$) δ 10.11 (s, 1H), 8.89 (dd, *J* = 7.4, 1.5 Hz, 1H), 8.69–8.58 (m, 1H), 8.18 (dd, *J* = 8.3, 1.7 Hz, 1H), 7.62–7.52 (m, 2H), 7.47–7.43 (m, 1H), 7.34–7.30 (m, 2H), 7.25–7.22 (m, 3H), 7.20 (dt, *J* = 7.6, 1.4 Hz, 1H), 7.13–7.09 (m, 1H), 6.82 (d, *J* = 8.0 Hz, 1H), 6.75 (td, *J* = 7.4, 1.0 Hz, 1H), 4.77–4.64 (m, 2H), 4.52 (d, *J* = 11.8 Hz, 1H), 4.42 (d, *J* = 11.9 Hz, 1H), 4.05–3.98 (m, 1H), 3.60–3.53 (m, 2H), 3.17–3.11 (m, 1H), 2.16–2.09 (m, 1H), 1.96–1.85 (m, 1H). **^{13}C NMR** (150 MHz, $CDCl_3$) δ 173.01, 160.36, 148.30, 138.63, 138.37, 136.37, 134.45, 128.79, 128.36, 128.32, 128.09, 127.71, 127.59, 127.50, 125.10, 121.90, 121.77, 120.69, 116.80, 109.75, 74.05, 73.20, 67.85, 49.12, 44.40, 29.87. **HRMS (ESI-TOF)** Calcd for $C_{28}H_{27}N_2O_3^+$ $[M+H]^+$ 439.2022, found 439.2014.

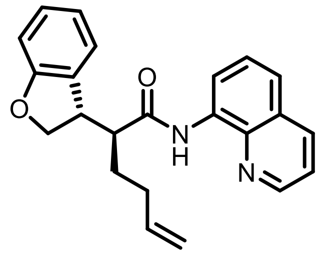

(±)-4fa
d.r. > 20:1

2-(2,3-dihydrobenzofuran-3-yl)-*N*-(quinolin-8-yl)hex-5-enamide ((±)-4fa): The title compound was prepared from **1f** (26.6 mg, 0.1 mmol) and 2-iodophenol (26.4 mg, 0.12 mmol) according to the General Procedure A. Purification using preparative TLC with 3:1 hexanes:EtOAc as the eluent gave the product as a colorless oil (16.5 mg, 46% yield). **^{1}H NMR** (600 MHz, $CDCl_3$) δ 9.84–9.81 (m, 1H), 8.87 (dd, *J* = 7.4, 1.5 Hz, 1H), 8.75 (dd, *J* = 4.1, 1.7 Hz, 1H), 8.17 (dd, *J* = 8.3, 1.7 Hz, 1H), 7.64–7.50 (m, 2H), 7.45 (dd, *J* = 8.2, 4.2 Hz, 1H), 7.22–7.15 (m, 1H), 7.08 (tdd, *J* = 8.1, 1.4, 0.7 Hz, 1H), 6.79 (ddt, *J* = 8.0, 1.0, 0.5 Hz, 1H), 6.71 (td, *J* = 7.4, 1.0 Hz, 1H), 5.80–5.70 (m, 1H), 5.12–4.95 (m, 2H), 4.71–4.60 (m, 2H), 3.93 (q, *J* = 7.6 Hz, 1H), 2.77 (ddd, *J* = 10.7, 7.8, 3.1 Hz, 1H), 2.28–2.19 (m, 1H), 2.15–2.08 (m, 1H), 2.08–2.01 (m, 1H), 1.60–1.55 (m, 1H). **^{13}C NMR** (150 MHz, $CDCl_3$) δ 172.21, 159.66, 147.75, 137.96, 136.96, 135.85, 133.61, 128.21, 127.65, 127.49, 126.92, 124.48, 121.39, 121.22, 120.11, 116.21, 115.57, 109.17, 73.50, 51.38, 44.19, 31.12, 28.49. **HRMS (ESI-TOF)** Calcd for $C_{23}H_{23}N_2O_2^+$ $[M+H]^+$ 359.1760, found 359.1767.

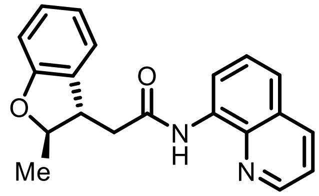

(±)-4ga
d.r. = 3:1

2-(2-methyl-2,3-dihydrobenzofuran-3-yl)-*N*-(quinolin-8-yl)acetamide ((±)-4ga): The title compound was prepared from **1g** (22.6 mg, 0.1 mmol) and 2-iodophenol (26.4 mg, 0.12 mmol) according to the General Procedure A. Purification using preparative TLC with 3:1 hexanes:EtOAc as the eluent gave the product as a white solid (29.6 mg, 93% yield). The reported *d.r.* was determined by ^{1}H NMR analysis of purified **(±)-4ga** and is consistent with that of the crude reaction mixture. The diastereomers were separated on a Waters Autopurification LC with a Waters BEH C18 column (5 μm, 19×160 mm) using a 0.1% aqueous formic acid:acetonitrile gradient (30 mL/min, main segment of gradient at 55–75% acetonitrile over 8 minutes) at ambient temperature. The following analytical data correspond to the major diastereomer. **^{1}H NMR** (600 MHz, $CDCl_3$) δ 9.84 (s, 1H), 8.81 (dd, *J* = 7.4, 1.6 Hz, 1H), 8.78–8.75 (m, 1H), 8.17 (dd, *J* = 8.2, 1.7 Hz, 1H), 7.59–7.55 (m, 1H), 7.53 (dd, *J* = 8.3, 1.6 Hz, 1H), 7.48–7.42 (m, 1H), 7.25–7.21 (m, 1H), 7.14 (dddd, *J* = 8.1, 7.5, 1.4, 0.7 Hz, 1H), 6.84 (td, J = 7.4, 1.0 Hz, 1H), 6.80 (ddt, *J* = 8.0, 1.0, 0.5 Hz, 1H), 4.70 (dd, *J* = 6.4, 5.6 Hz, 1H), 3.71 (dt, *J* = 8.4, 5.9 Hz, 1H), 2.97 (dd, *J* = 15.3, 6.2 Hz, 1H), 2.85 (dd, *J* = 15.3, 8.5 Hz, 1H), 1.51 (d, *J* = 6.3 Hz, 3H). **^{13}C NMR** (150 MHz, $CDCl_3$) δ 169.47, 159.13, 148.37, 138.43, 136.53, 134.36, 129.62, 128.78, 128.11, 127.54, 124.83, 121.90, 121.85, 120.62, 116.71, 109.89, 85.32, 45.81, 43.28, 21.36. **HRMS (ESI-TOF)** Calcd for $C_{20}H_{19}N_2O_2^+$ $[M+H]^+$ 319.1447, found 319.1448. **X-ray** (single-crystal) Colorless needles of X-ray diffraction quality were obtained by vapor diffusion of hexane into a saturated solution of **4ga** in toluene (CCDC 1999888).[16]

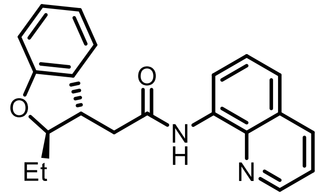

(±)-4ha
d.r. = 17:1

2-(2-ethyl-2,3-dihydrobenzofuran-3-yl)-*N*-(quinolin-8-yl)acetamide ((±)-4ha): The title compound was prepared from **1h** (24.0 mg, 0.1 mmol) and 2-iodophenol (26.4 mg, 0.12 mmol) according to the General Procedure A. Purification using preparative TLC with 3:1 hexanes:EtOAc as the eluent gave the product as a white solid (24.6 mg, 74% yield). The reported *d.r.* was determined by ^{1}H NMR analysis of purified **(±)-4ha** and is consistent with that of the crude reaction mixture. The following analytical data correspond to the major diastereomer. **^{1}H NMR** (600 MHz, $CDCl_3$) δ 9.83 (s, 1H), 8.81 (dd, *J* = 7.5, 1.5 Hz, 1H), 8.77 (dd, *J* = 4.2, 1.7 Hz, 1H), 8.17 (dd, *J* = 8.3, 1.7 Hz, 1H), 7.58–7.55 (m, 1H), 7.53 (dd, *J* = 8.3, 1.5 Hz,

1H), 7.45 (dd, J = 8.2, 4.2 Hz, 1H), 7.25–7.21 (m, 1H), 7.13 (dddd, J = 8.1, 7.5, 1.4, 0.7 Hz, 1H), 6.85–6.77 (m, 2H), 4.51 (dt, J = 7.0, 5.3 Hz, 1H), 3.78 (q, J = 6.6 Hz, 1H), 2.95 (dd, J = 15.1, 6.5 Hz, 1H), 2.85 (dd, J = 15.1, 8.1 Hz, 1H), 1.88–1.75 (m, 2H), 1.05 (t, J = 7.4 Hz, 3H). **^{13}C NMR** (150 MHz, $CDCl_3$) δ 168.87, 158.78, 147.63, 137.84, 135.91, 133.77, 129.18, 128.13, 127.50, 126.93, 124.24, 121.27, 121.24, 119.90, 116.11, 109.14, 89.53, 43.25, 43.13, 27.96, 9.13. **HRMS (ESI-TOF)** Calcd for $C_{21}H_{21}N_2O_2^+$ $[M+H]^+$ 333.1603, found 333.1607.

General Procedure B for [3+2] Indoline Synthesis

To a 4-mL scintillation vial equipped with a Teflon-coated magnetic stir bar were added the $Pd(OAc)_2$ (1.1 mg, 0.005 mmol, 5 mol%), alkene substrate (21.2 mg, 0.1 mmol), the appropriate *ortho*-iodoaniline substrate (0.12 mmol, 1.2 equiv), K_2CO_3 (13.8 mg, 0.1 mmol, 1 equiv), and HFIP (0.2 mL). The vial was sealed with a screw-top septum cap and placed in a heating block that was pre-heated to 80 °C. The reaction was allowed to run for 24 h and then was cooled to room temperature. The reaction mixture was then filtered through a short plug of Celite, and concentrated to afford a brown residue, which, upon purification by preparative TLC, afforded pure product.

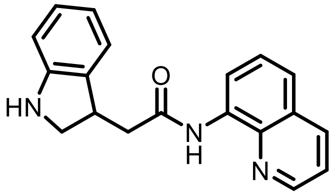

3ba

2-(indolin-3-yl)-*N*-(quinolin-8-yl)acetamide (3ba): The title compound was prepared from **1a** (21.2 mg, 0.1 mmol) and 2-iodoaniline (26.3 mg, 0.12 mmol) according to the General Procedure B. Purification using preparative TLC with 1:1 hexanes:EtOAc as the eluent gave the product as a yellow solid (22.7 mg, 75% yield). **^{1}H NMR** (600 MHz, $CDCl_3$) δ 9.84 (s, 1H), 8.81 (dd, J = 7.5, 1.5 Hz, 1H), 8.77 (dd, J = 4.2, 1.7 Hz, 1H), 8.16 (dd, J = 8.2, 1.7 Hz, 1H), 7.56 (dd, J = 8.3, 7.5 Hz, 1H), 7.52 (dd, J = 8.3, 1.5 Hz, 1H), 7.45 (dd, J = 8.2, 4.2 Hz, 1H), 7.19 (dtd, J = 7.3, 1.1, 0.6 Hz, 1H), 7.08–7.03 (m, 1H), 6.72 (td, J = 7.4, 1.0 Hz, 1H), 6.70–6.65 (m, 1H), 3.97 (tt, J = 8.8, 6.0 Hz, 1H), 3.88 (t, J = 8.9 Hz, 1H), 3.79 (s, 1H), 3.42 (dd, J = 9.1, 6.3 Hz, 1H), 3.01 (dd, J = 15.1, 5.7 Hz, 1H), 2.85 (dd, J = 15.1, 9.0 Hz, 1H). **^{13}C NMR** (150 MHz, $CDCl_3$) δ 170.27, 151.39, 148.34, 138.47, 136.50, 134.52, 131.67, 128.09, 127.55, 124.25, 121.78, 121.73, 119.01, 116.65, 109.83, 53.44, 42.86, 39.02. **HRMS (ESI-TOF)** Calcd for $C_{19}H_{18}N_3O^+$ $[M+H]^+$ 304.1450, found 304.1453.

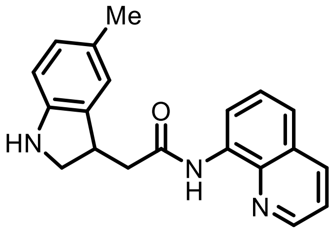

3bj

2-(5-methylindolin-3-yl)-*N*-(quinolin-8-yl)acetamide (3bj): The title compound was prepared from **1a** (21.2 mg, 0.1 mmol) and 2-iodo-4-methylaniline (28.0 mg, 0.12 mmol) according to the General Procedure B. Purification using preparative TLC with 1:1 hexanes:EtOAc as the eluent gave the product as a yellow solid (15.9 mg, 50% yield). **^{1}H NMR** (600 MHz, $CDCl_3$) δ 9.83 (s, 1H), 8.81 (dd, J = 7.5, 1.4 Hz, 1H), 8.77 (dd, J = 4.2, 1.7 Hz, 1H), 8.16 (dd, J = 8.2, 1.7 Hz, 1H), 7.57–7.54 (m, 1H), 7.52 (dd, J = 8.3, 1.5 Hz, 1H), 7.45 (dd, J = 8.2, 4.2 Hz, 1H), 7.02–7.00 (m, 1H), 6.89–6.84 (m, 1H), 6.59 (d, J = 7.8 Hz, 1H), 3.92 (tt, J = 8.8, 5.8 Hz, 1H), 3.86 (t, J = 8.9 Hz, 1H), 3,71 (br, 1H), 3.40 (dd, J = 9.1, 6.2 Hz, 1H), 3.00 (dd, J = 15.1, 5.5 Hz, 1H), 2.83 (dd, J = 15.1, 8.9 Hz, 1H), 2.22 (s, 3H). **^{13}C NMR** (150 MHz, $CDCl_3$) δ 170.34, 149.02, 148.31, 138.47, 136.48, 134.53, 132.04, 128.45, 128.43, 128.08,

127.55, 124.95, 116.64, 109.87, 53.70, 42.83, 39.17, 20.95. **HRMS (ESI-TOF)** Calcd for $C_{20}H_{20}N_3O^+$ $[M+H]^+$ 318.1606, found 318.1614.

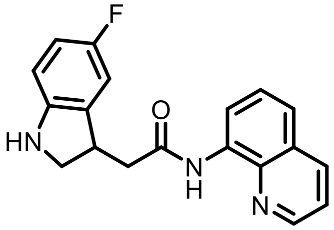

3bc

2-(5-fluoroindolin-3-yl)-*N*-(quinolin-8-yl)acetamide (3bc): The title compound was prepared from **1a** (21.2 mg, 0.1 mmol) and 4-fluoro-2-iodoaniline (28.4 mg, 0.12 mmol) according to the General Procedure B. Purification using preparative TLC with 1:1 hexanes:EtOAc as the eluent gave the product as a yellow solid (23.4 mg, 73% yield). **^{1}H NMR** (600 MHz, $CDCl_3$) δ 9.83 (s, 1H), 8.78 (dd, *J* = 12.6, 5.8 Hz, 2H), 8.16 (d, *J* = 8.2 Hz, 1H), 7.58–7.50 (m, 2H), 7.45 (dd, *J* = 8.3, 4.2 Hz, 1H), 6.92 (dd, *J* = 8.4, 2.7 Hz, 1H), 6.74 (td, J = 8.9, 2.7 Hz, 1H), 6.56 (dd, *J* = 8.5, 4.3 Hz, 1H), 4.00–3.91 (m, 1H), 3.88 (t, *J* = 8.9 Hz, 1H), 3.70 (br, 1H), 3.42 (dd, *J* = 9.1, 6.1 Hz, 1H), 2.97 (dd, *J* = 15.2, 5.8 Hz, 1H), 2.85 (dd, *J* = 15.1, 8.6 Hz, 1H). **^{13}C NMR** (150 MHz, $CDCl_3$) δ 169.83, 157.21 (d, *J* = 235.5 Hz), 148.33, 147.35, 138.42, 136.50, 134.40, 133.45 (d, *J* = 7.8 Hz), 128.08, 127.52, 121.82, 121.80, 116.67, 114.11 (d, *J* = 23.2 Hz), 111.72 (d, *J* = 23.9 Hz), 110.10 (d, *J* = 8.2 Hz), 53.98, 42.48, 39.27 (d, *J* = 2.1 Hz). **^{19}F NMR** (376 MHz, $CDCl_3$) δ -126.17. **HRMS (ESI-TOF)** Calcd for $C_{19}H_{17}FN_3O^+$ $[M+H]^+$ 322.1356, found 322.1362.

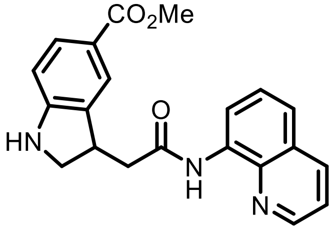

3bg

Methyl 3-(2-oxo-2-(quinolin-8-ylamino)ethyl)indoline-5-carboxylate (3bg): The title compound was prepared from **1a** (21.2 mg, 0.1 mmol) and methyl 4-amino-3-iodobenzoate (33.2 mg, 0.12 mmol) according to the General Procedure B. Purification using preparative TLC with 1:1 hexanes:EtOAc as the eluent gave the product as a yellow solid (30.0 mg, 83% yield). **^{1}H NMR** (600 MHz, $CDCl_3$) δ 9.83 (s, 1H), 8.80–8.74 (m, 2H), 8.16 (dd, *J* = 8.2, 1.7 Hz, 1H), 7.83 (s, 1H), 7.81–7.78 (m, 1H), 7.57–7.53 (m, 1H), 7.52 (dd, *J* = 8.3, 1.5 Hz, 1H), 7.45 (dd, *J* = 8.2, 4.2 Hz, 1H), 6.57 (d, *J* = 8.2 Hz, 1H), 4.16 (s, 1H), 4.03–3.92 (m, 2H), 3.81 (s, 3H), 3.55 (dd, *J* = 8.3, 5.2 Hz, 1H), 3.11–3.04 (dd, 1H), 2.88–2.80 (dd, 1H). **^{13}C NMR** (150 MHz, $CDCl_3$) δ 169.88, 167.41, 155.62, 148.34, 138.44, 136.48, 134.41, 131.50, 130.99, 128.07, 127.52, 125.83, 121.81, 121.79, 119.95, 116.65, 107.87, 53.51, 51.68, 42.80, 38.21. **HRMS (ESI-TOF)** Calcd for $C_{21}H_{20}N_3O_3^+$ $[M+H]^+$ 362.1499, found 362.1499.

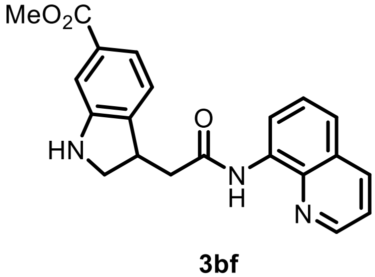

3bf

Methyl 3-(2-oxo-2-(quinolin-8-ylamino)ethyl)indoline-6-carboxylate (3bf): To an 8-mL scintillation vial equipped with a Teflon-coated magnetic stir bar were added the $Pd(OAc)_2$ (2.6 mg, 0.0118 mmol, 5 mol%), **1a** (50 mg, 0.24 mmol), methyl 3-amino-4-iodobenzoate (78 mg, 0.28 mmol), K_2CO_3 (32.6 mg, 0.236 mmol, 1 equiv), and HFIP (0.48 mL). The vial was sealed with a screw-top septum cap and placed in a heating block that was pre-heated to 80 °C. The reaction was allowed to run for 24 h and then was cooled to room temperature. The reaction mixture was then filtered through an autovial syringeless filter device, and concentrated to afford a brown residue, which, upon purification by ISCO automated silica gel column chromatography using an eluent system of 0–100% EtOAc/heptane, afforded the product as a yellow solid (79 mg, 93% yield). **^{1}H NMR** (400 MHz, $CDCl_3$) δ = 9.84 (br s, 1H), 8.81 (dd, *J* = 1.7, 7.2 Hz, 1H), 8.77 (dd, *J* = 1.7, 4.2 Hz, 1H), 8.18 (dd, *J* = 1.6, 8.3 Hz, 1H), 7.60–7.51 (m, 2H), 7.49–7.40 (m, 2H), 7.29 (d, *J* = 1.3 Hz, 1H), 7.23 (d, *J* = 7.7 Hz, 1H), 4.03–3.91 (m, 2H), 3.90 (br, 1H), 3.88 (s, 3H), 3.48 (dd, *J* = 6.0, 8.9 Hz, 1H), 3.06–2.97 (m, 1H),

2.92–2.81 (m, 1H) ^{13}**C NMR** (100 MHz, $CDCl_3$) δ 169.64, 167.40, 151.41, 148.17, 138.28, 136.91, 136.40, 134.27, 130.11, 127.97, 127.40, 123.81, 121.69, 121.65, 120.84, 116.58, 109.95, 53.39, 51.94, 42.35, 38.76. **HRMS (ESI-TOF)** Calcd for $C_{21}H_{20}N_3O_3^+$ $[M+H]^+$ 362.14992, found 362.15175.

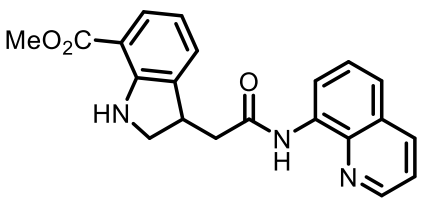

3be

Methyl 3-(2-oxo-2-(quinolin-8-ylamino)ethyl)indoline-7-carboxylate (3be): To an 8-mL scintillation vial equipped with a Teflon-coated magnetic stir bar were added the $Pd(OAc)_2$ (2.6 mg, 0.0118 mmol, 5 mol%), **1a** (50 mg, 0.24 mmol) and methyl 2-amino-3-iodobenzoate (78 mg, 0.28 mmol), K_2CO_3 (32.6 mg, 0.236 mmol, 1 equiv), and HFIP (0.48 mL). The vial was sealed with a screw-top septum cap and placed in a heating block that was pre-heated to 80 °C. The reaction was allowed to run for 24 h and then was cooled to room temperature. The reaction mixture was then filtered through an autovial syringeless filter device, and concentrated to afford a brown residue, which, upon purification by ISCO automated silica gel column chromatography using an eluent system of 0–100% EtOAc/heptane, afforded the product as a yellow solid (65 mg, 76% yield).^{1}**H NMR** (400 MHz, $CDCl_3$) δ = 9.84 (br s, 1H), 8.81 (dd, *J* = 1.6, 7.2 Hz, 1H), 8.78 (dd, *J* = 1.7, 4.2 Hz, 1H), 8.17 (dd, *J* = 1.6, 8.3 Hz, 1H), 7.61 (d, *J* = 8.1 Hz, 1H), 7.59–7.51 (m, 2H), 7.46 (dd, *J* = 4.2, 8.3 Hz, 1H), 7.27–7.25 (m, 1H), 6.58 (dd, *J* = 7.2, 8.0 Hz, 1H), 6.09 (s, 1H), 4.07–3.94 (m, 2H), 3.88 (s, 3H), 3.62–3.54 (m, 1H), 3.04–2.95 (m, 1H), 2.91 - 2.79 (m, 1H) ^{13}**C NMR** (100 MHz, $CDCl_3$) δ 169.72, 167.99, 153.94, 148.20, 138.32, 136.35, 134.30, 133.14, 128.62, 128.29, 127.96, 127.39, 121.66, 116.54, 116.35, 108.21, 52.98, 51.45, 43.01, 37.64. **HRMS (ESI-TOF)** Calcd for $C_{21}H_{20}N_3O_3^+$ $[M+H]^+$ 362.14992, found 362.14994.

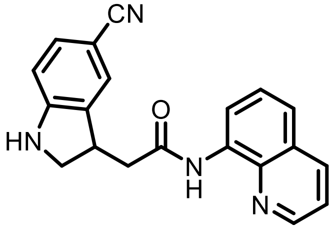

3bb

2-(5-cyanoindolin-3-yl)-*N*-(quinolin-8-yl)acetamide (3bb): The title compound was prepared from **1a** (21.2 mg, 0.1 mmol) and 4-amino-3-iodobenzonitrile (29.3 mg, 0.12 mmol) according to the General Procedure B. Purification using preparative TLC with 1:1 hexanes:EtOAc as the eluent gave the product as a yellow solid (32.5 mg, 99% yield).^{1}**H NMR** (600 MHz, $CDCl_3$) δ 9.83 (s, 1H), 8.81–8.74 (m, 2H), 8.17 (dd, *J* = 8.3, 1.7 Hz, 1H), 7.63–7.50 (m, 2H), 7.46 (dd, *J* = 8.2, 4.2 Hz, 1H), 7.39 (dd, *J* = 1.6, 0.8 Hz, 1H), 7.32 (dd, *J* = 8.1, 1.7 Hz, 1H), 6.56 (d, *J* = 8.2 Hz, 1H), 4.26 (s, 1H), 4.02–3.95 (m, 2H), 3.57–3.50 (m, 1H), 3.03–2.94 (m, 1H), 2.92–2.81 (m, 1H). ^{13}**C NMR** (150 MHz, $CDCl_3$) δ 168.75, 154.41, 147.80, 137.80, 135.97, 133.65, 133.22, 131.34, 127.51, 127.42, 126.92, 121.38, 121.28, 120.10, 116.11, 107.91, 99.44, 52.58, 42.03, 37.36. **HRMS (ESI-TOF)** Calcd for $C_{20}H_{17}N_4O^+$ $[M+H]^+$ 329.1402, found 329.1405.

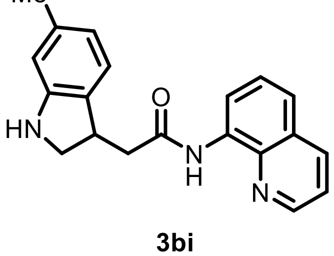

3bi

2-(6-methylindolin-3-yl)-*N*-(quinolin-8-yl)acetamide (3bi): The title compound was prepared from **1a** (21.2 mg, 0.1 mmol) and 2-iodo-5-methylaniline (28.0 mg, 0.12 mmol) according to the General Procedure B. Purification using preparative TLC with 1:1 hexanes:EtOAc as the eluent gave the product as a yellow solid (19.3 mg, 61% yield).^{1}**H NMR** (600 MHz, $CDCl_3$) δ 9.84 (s, 1H), 8.81 (dd, *J* = 7.5, 1.4 Hz, 1H), 8.77 (dd, *J* = 4.2, 1.7 Hz, 1H), 8.16 (dd, *J* = 8.3, 1.7 Hz, 1H), 7.58–7.53 (m, 1H), 7.52 (dd, *J* = 8.3, 1.5 Hz, 1H), 7.45

(dd, *J* = 8.2, 4.2 Hz, 1H), 7.07 (d, *J* = 7.5 Hz, 1H), 6.55–6.52 (m, 1H), 6.52–6.49 (m, 1H), 3.95–3.90 (m, 1H), 3.87 (t, *J* = 8.8 Hz, 1H), 3.40 (dd, J = 8.9, 6.0 Hz, 1H), 2.98 (dd, *J* = 15.0, 5.6 Hz, 1H), 2.82 (dd, *J* = 15.1, 8.8 Hz, 1H), 2.26 (s, 3H). **^{13}C NMR** (150 MHz, $CDCl_3$) δ 170.35, 151.62, 148.31, 138.46, 138.04, 136.48, 134.54, 128.85, 128.08, 127.55, 123.93, 121.77, 121.70, 119.74, 116.64, 110.70, 53.66, 43.00, 38.72, 21.63. **HRMS (ESI-TOF)** Calcd for $C_{20}H_{20}N_3O^+$ $[M+H]^+$ 318.1606, found 318.1610.

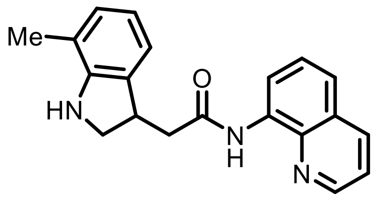

3bh

2-(7-methylindolin-3-yl)-*N*-(quinolin-8-yl)acetamide (3bh): To an 8-mL scintillation vial equipped with a Teflon-coated magnetic stir bar were added the $Pd(OAc)_2$ (2.6 mg, 0.0118 mmol, 5 mol%), **1a** (50 mg, 0.24 mmol) and 2-bromo-6-methylaniline (52.6 mg, 0.28 mmol), K_2CO_3 (32.6 mg, 0.236 mmol, 1 equiv), and HFIP (0.48 mL). The vial was sealed with a screw-top septum cap and placed in a heating block that was pre-heated to 80 °C. The reaction was allowed to run for 24 h and then was cooled to room temperature. The reaction mixture was then filtered through an autovial syringeless filter device, and concentrated to afford a brown residue, which, upon purification by ISCO automated silica gel column chromatography using an eluent system of 0–100% EtOAc/heptane, afforded the product as a yellow solid (49 mg, 66% yield).**^{1}H NMR** (600 MHz, $CDCl_3$) δ = 9.85 (br s, 1H), 8.82 (br d, J = 6.5 Hz, 1H), 8.78 (dd, J = 1.4, 4.1 Hz, 1H), 8.17 (dd, J = 1.3, 8.3 Hz, 1H), 7.617.49 (m, 2H), 7.46 (dd, J = 4.2, 8.2 Hz, 1H), 7.07 (d, J = 7.3 Hz, 1H), 6.92 (d, J = 7.3 Hz, 1H), 6.69 (t, J = 7.5 Hz, 1H), 4.07–3.97 (m, 1H), 3.96–3.86 (m, 1H), 3.60 (br, 1H), 3.46 (dd, J = 6.2, 9.0 Hz, 1H), 3.02 (dd, J = 5.5, 15.2 Hz, 1H), 2.85 (dd, J = 8.9, 15.2 Hz, 1H), 2.16 (s, 3H) **^{13}C NM**R (150 MHz, $CDCl_3$) δ 170.32, 149.91, 148.31, 138.50, 136.47, 134.57, 131.04, 129.02, 128.10, 127.55, 121.76, 121.70, 119.32, 119.21, 116.67, 53.34, 43.02, 39.41, 16.89. **HRMS (ESI-TOF)** Calcd for $C_{20}H_{20}N_3O_+$ $[M+H]_+$ 318.1606, found 318.1618.

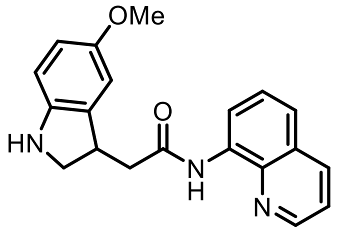

3bd

2-(5-methoxyindolin-3-yl)-*N*-(quinolin-8-yl)acetamide (3bd): To an 8-mL scintillation vial equipped with a Teflon-coated magnetic stir bar were added the $Pd(OAc)_2$ (2.6 mg, 0.0118 mmol, 5 mol%), alkene substrate **1a** (50 mg, 0.24 mmol), 2-iodo-4-methoxyaniline (70.4 mg, 0.28 mmol, 1.2 equiv), K_2CO_3 (32.6 mg, 0.236 mmol, 1 equiv), and HFIP (0.48 mL). The vial was sealed with a screw-top septum cap and placed in a heating block that was pre-heated to 80 °C. The reaction was allowed to run for 24 h and then was cooled to room temperature. The reaction mixture was then filtered through an autovial syringeless filter device, and concentrated to afford a brown residue, which, upon purification by ISCO automated silica gel column chromatography using an eluent system of 0–100% EtOAc/heptane, afforded pure product as a dark red solid (37.1 mg, 47% yield). **^{1}H NMR** (400 MHz, $CDCl_3$) δ = 9.95–9.77 (m, 1H), 8.87–8.72 (m, 2H), 8.19–8.14 (m, 1H), 7.59–7.50 (m, 2H), 7.49–7.42 (m, 1H), 6.87–6.81 (m, 1H), 6.83–6.76 (m, 1H), 6.71–6.65 (m, 1H), 4.04–3.88 (m, 2H), 3.75 (s, 3H), 3.53–3.47 (m, 1H), 3.06–2.81 (m, 2H) **^{13}C NMR** (100 MHz, $CDCl_3$) δ = 169.94, 154.04, 148.18, 144.28, 138.34, 136.32, 134.36, 133.52, 127.95, 127.38, 121.63, 121.61, 116.54, 113.36, 111.02, 110.75, 55.95, 53.69, 42.57, 39.49 **HRMS (ESI-TOF)** Calcd for $C_{20}H_{20}N_3O_2^+$ $[M+H]^+$ 334.15398, found 334.15500.

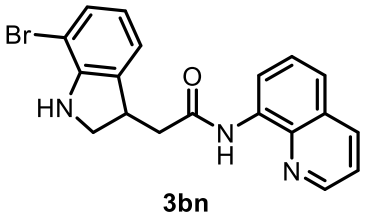

3bn

2-(7-bromoindolin-3-yl)-*N*-(quinolin-8-yl)acetamide (3bn): To an 8-mL scintillation vial equipped with a Teflon-coated magnetic stir bar were added the $Pd(OAc)_2$ (2.6 mg, 0.0118 mmol, 5 mol%), alkene substrate **1a** (50 mg, 0.24 mmol), 2-bromo-6-iodoaniline (84.2 mg, 0.28 mmol, 1.2 equiv), K_2CO_3 (32.6 mg, 0.236 mmol, 1 equiv), and HFIP (0.48 mL). The vial was sealed with a screw-top septum cap and placed in a heating block that was pre-heated to 80 °C. The reaction was allowed to run for 24 h and then was cooled to room temperature. The reaction mixture was then filtered through an autovial syringeless filter device, and concentrated to afford a brown residue, which, upon purification by ISCO automated silica gel column chromatography using an eluent system of 0–100% EtOAc/heptane, afforded pure product as a white solid (28.8 mg, 32% yield). **^{1}H NMR** (400 MHz, $CDCl_3$) δ = 9.86 (br s, 1H), 8.84 - 8.78 (m, 2H), 8.19 (dd, *J* = 1.6, 8.3 Hz, 1H), 7.60–7.53 (m, 2H), 7.48 (dd, *J* = 4.2, 8.3 Hz, 1H), 7.22 (d, *J* = 7.9 Hz, 1H), 7.12 (d, *J* = 7.2 Hz, 1H), 6.59 (t, *J* = 7.7 Hz, 1H), 4.14–4.01 (m, 2H), 4.01–3.93 (m, 1H), 3.51 (dd, *J* = 6.0, 9.2 Hz, 1H), 3.01 (dd, *J* = 5.9, 15.2 Hz, 1H), 2.94–2.86 (m, 1H). **^{13}C NMR** (100 MHz, $CDCl_3$) δ = 169.66, 149.73, 148.20, 138.32, 136.35, 134.30, 132.73, 130.49, 127.95, 127.38, 122.97, 121.66, 119.82, 116.54, 103.35, 52.72, 42.67, 39.93. **HRMS (ESI-TOF)** Calcd for $C_{19}H_{17}BrN_3O^+$ $[M+H]^+$ 382.05495, found 382.05658.

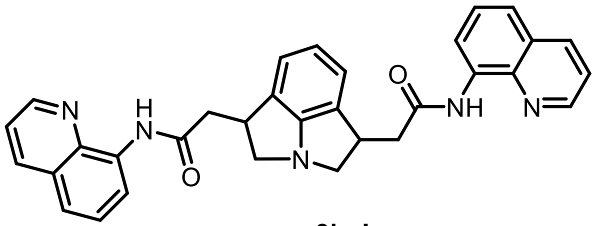

3bn'
d.r. =1:1

2,2'-(1,2,4,5-tetrahydropyrrolo[3,2,1-*hi*]indole-1,5-diyl)bis(*N*-(quinolin-8-yl)acetamide) (3bn'): To an 8-mL scintillation vial equipped with a Teflon-coated magnetic stir bar were added the $Pd(OAc)_2$ (2.6 mg, 0.0118 mmol, 5 mol%), alkene substrate **1a** (50 mg, 0.24 mmol), 2-bromo-6-iodoaniline (84.2 mg, 0.28 mmol, 1.2 equiv), K_2CO_3 (32.6 mg, 0.236 mmol, 1 equiv), and HFIP (0.48 mL). The vial was sealed with a screw-top septum cap and placed in a heating block that was pre-heated to 80 °C. The reaction was allowed to run for 24 h and then was cooled to room temperature. The reaction mixture was then filtered through an autovial syringeless filter device, and concentrated to afford a brown residue, which, upon purification by ISCO automated silica gel column chromatography using an eluent system of 0–100% EtOAc/heptane, afforded pure product as a yellow foam (36.5 mg, 59% yield). The reported *d.r.* was determined by ^{1}H NMR analysis of purified **3bn'** and is consistent with that of the crude reaction mixture. **^{1}H NMR** (400 MHz, $CDCl_3$) δ = 9.93–9.85 (m, 2H), 8.86–8.79 (m, 4H), 8.22–8.13 (m, 2H), 7.61–7.45 (m, 6H), 7.03 (d, *J* = 7.5 Hz, 2H), 6.70–6.60 (m, 1H), 4.40–4.23 (m, 2H), 3.79–3.68 (m, 1H), 3.62–3.51 (m, 1H), 3.20–3.04 (m, 3H), 3.04–2.85 (m, 3H) **^{13}C NMR** (150 MHz, DMSO-d_6) δ = 170.20, 163.48, 163.45, 148.77, 148.76, 138.16, 136.49, 136.47, 134.46, 127.81, 126.90, 126.88, 125.70, 125.65, 122.16, 122.03, 122.01, 121.87, 121.71, 119.66, 119.60, 116.94, 116.91, 64.30, 63.54, 44.41, 44.28, 40.54, 40.09, 40.04, 28.28, 14.01,13.87. **HRMS (ESI-TOF)** Calcd for $C_{32}H_{28}N_5O_2^+$ $[M+H]^+$ calc: 514.22375, found 514.22694. **X-ray** (single-crystal) Colorless needle crystals of X-ray diffraction quality were obtained by vapor diffusion of hexane into a saturated solution of **3bn** in DCM (CCDC 2033387).[16]

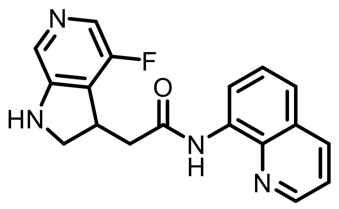

3br

2-(4-fluoro-2,3-dihydro-1H-pyrrolo[2,3-*c*]pyridin-3-yl)-*N*-(quinolin-8-yl)acetamide (3br): To an 8-mL scintillation vial equipped with a Teflon-coated magnetic stir bar were added the Pd(OAc)$_2$ (12.7 mg, 0.057 mmol, 20 mol%), alkene substrate **1a** (60 mg, 0.28 mmol), 5-fluoro-4-iodopyridin-3-amine (80.7 mg, 0.34 mmol, 1.2 equiv), K_2CO_3 (39.1 mg, 0.28 mmol, 1 equiv), and HFIP (0.57 mL). The vial was sealed with a screw-top septum cap and placed in a heating block that was pre-heated to 100 °C. The reaction was allowed to run for 24 h and then was cooled to room temperature. The reaction mixture was then filtered through an autovial syringeless filter device, and concentrated to afford a brown residue, which, upon purification by reverse phase HPLC, afforded pure product as a yellow film (45 mg, 49% yield). **^{1}H NMR** (400 MHz, DMSO-*d*$_6$) δ 10.21 (s, 1H), 8.92 (dd, *J* = 4.2, 1.7 Hz, 1H), 8.62 (dd, *J* = 7.7, 1.4 Hz, 1H), 8.40 (dd, *J* = 8.3, 1.7 Hz, 1H), 7.72–7.65 (m, 3H), 7.65–7.54 (m, 2H), 6.15–6.07 (m, 1H), 4.03–3.91 (m, 1H), 3.76 (td, *J* = 9.5, 1.5 Hz, 1H), 3.38 (ddd, *J* = 9.6, 6.5, 1.7 Hz, 1H), 3.09 (dd, *J* = 15.4, 4.9 Hz, 1H), 2.96 (dd, *J* = 15.4, 9.2 Hz, 1H). **^{13}C NMR** (100 MHz, DMSO) δ 169.57, 157.86, 155.34, 151.49 (d, *J* = 6.0 Hz), 148.81, 138.22, 136.50, 134.46, 127.82, 126.87, 126.64, 126.61, 126.24, 126.01, 124.26, 124.09, 122.05, 121.99, 117.09, 52.47, 36.27. **HRMS (ESI-TOF)** Calcd for $C_{18}H_{15}FN_4ONa^+$ $[M+Na]^+$ calc: 345.1122, found 345.1117.

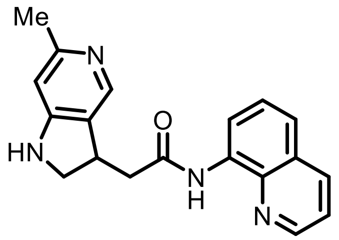

3bq

2-(6-methyl-2,3-dihydro-1H-pyrrolo[3,2-*c*]pyridin-3-yl)-*N*-(quinolin-8-yl)acetamide (3bq): To an 8-mL scintillation vial equipped with a Teflon-coated magnetic stir bar were added the Pd(OAc)$_2$ (21.2 mg, 0.094 mmol, 20 mol%), alkene substrate **1a** (100 mg, 0.47 mmol), 3-iodopyridin-4-amine (124 mg, 0.57 mmol, 1.2 equiv , K_2CO_3 (65.1 mg, 0.47 mmol, 1 equiv), and HFIP (0.94 mL). The vial was sealed with a screw-top septum cap and placed in a heating block that was pre-heated to 100 °C. The reaction was allowed to run for 24 h and then was cooled to room temperature. The reaction mixture was then filtered through an autovial syringeless filter device, and concentrated to afford a brown residue, which, upon purification by reverse phase HPLC, afforded pure product as a colorless oil (90 mg, 56% yield). **^{1}H NMR** (400 MHz, DMSO-*d*$_6$) δ 10.18 (s, 1H), 8.92 (dd, *J* = 4.2, 1.7 Hz, 1H), 8.64 (dd, *J* = 7.7, 1.4 Hz, 1H), 8.41 (dd, *J* = 8.3, 1.7 Hz, 1H), 7.91 (s, 1H), 7.71–7.55 (m, 3H), 6.81 (s, 1H), 6.32 (s, 1H), 3.85–3.70 (m, 2H), 3.43–3.35 (m, 1H), 3.11–3.01 (m, 1H), 2.87 (dd, *J* = 15.2, 7.9 Hz, 1H), 2.26 (s, 3H). **^{13}C NMR** (100 MHz, DMSO) δ 170.25, 158.54, 156.12, 148.81, 141.73, 138.18, 136.53, 134.46, 127.85, 126.92, 125.06, 122.07, 121.95, 116.97, 102.00, 52.31, 41.75, 35.57, 23.54. **HRMS (ESI-TOF)** Calcd for $C_{19}H_{19}N_4O^+$ $[M+H]^+$ calc: 319.1153, found 319.1564.

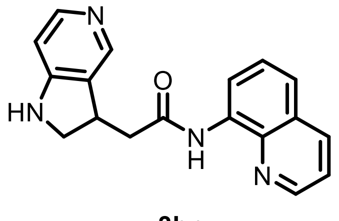

3bp

2-(2,3-dihydro-1*H*-pyrrolo[3,2-*c*]pyridin-3-yl)-*N*-(quinolin-8-yl)acetamide (3bp): To an 8-mL scintillation vial equipped with a Teflon-coated magnetic stir bar were added the Pd(OAc)$_2$ (10.6 mg, 0.047 mmol, 20 mol%), alkene substrate **1a** (50 mg, 0.24 mmol), 4-iodo-1-methyl-1H-pyrazol-3-amine (63 mg, 0.28 mmol, 1.2 equiv), K_2CO_3 (32.6 mg, 0.24 mmol, 1 equiv), and HFIP (0.47 mL). The vial was sealed with a screw-top septum cap and placed in a heating block that was pre-heated to 100 °C. The reaction was allowed to run for 24 h and then was cooled to room temperature. The reaction mixture was then filtered through an autovial syringeless filter

device, and concentrated to afford a brown residue, which, upon purification by reverse phase HPLC, afforded pure product as a colorless oil (46 mg, 63% yield). **^{1}H NMR** (400 MHz, DMSO-*d*$_6$) δ 10.19 (s, 1H), 8.91 (dd, *J* = 4.2, 1.7 Hz, 1H), 8.66 (dd, *J* = 7.6, 1.4 Hz, 1H), 8.40 (dd, *J* = 8.3, 1.7 Hz, 1H), 7.68 (dd, *J* = 8.3, 1.3 Hz, 1H), 7.65–7.56 (m, 2H), 6.52 (d, *J* = 29.5 Hz, 2H), 3.87–3.69 (m, 2H), 3.35 (q, *J* = 4.7 Hz, 1H), 3.10 (dd, *J* = 15.4, 5.5 Hz, 1H), 2.94–2.79 (m, 1H), 2.00–1.83 (m, 2H). **^{13}C NMR** (100 MHz, DMSO) δ 170.23, 157.62, 148.81, 148.16, 143.16, 138.19, 136.53, 134.46, 127.85, 126.92, 122.06, 121.96, 116.98, 52.11, 41.65, 36.04. **HRMS (ESI-TOF)** Calcd for $C_{18}H_{17}N_4O^+$ $[M+H]^+$ calc: 305.1397, found 305.1411.

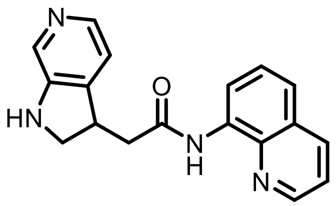

3bo

2-(2,3-dihydro-1*H*-pyrrolo[2,3-*c*]pyridin-3-yl)-*N*-(quinolin-8-yl)acetamide (3bo): To an 8-mL scintillation vial equipped with a Teflon-coated magnetic stir bar were added the Pd(OAc)$_2$ (10.6 mg, 0.047 mmol, 20 mol%), alkene substrate **1a** (50 mg, 0.24 mmol), 4-iodopyridin-3-amine (62.2 mg, 0.28 mmol, 1.2 equiv), K_2CO_3 (32.6 mg, 0.24 mmol, 1 equiv), and HFIP (0.47 mL). The vial was sealed with a screw-top septum cap and placed in a heating block that was pre-heated to 80 °C. The reaction was allowed to run for 24 h and then was cooled to room temperature. The reaction mixture was then filtered through an autovial syringeless filter device, and concentrated to afford a brown residue, which, upon purification by ISCO automated silica gel column chromatography using an eluent system of 0–60% EtOAc/heptane, afforded pure product as a colorless oil 61 mg, 85% yield). **^{1}H NMR** (400 MHz, Chloroform-*d*) δ 9.82 (s, 1H), 8.79–8.71 (m, 2H), 8.15 (dd, *J* = 8.3, 1.7 Hz, 1H), 8.04–7.95 (m, 2H), 7.57–7.47 (m, 2H), 7.44 (dd, *J* = 8.3, 4.2 Hz, 1H), 7.11 (dt, *J* = 4.8, 1.0 Hz, 1H), 4.03–3.93 (m, 1H), 3.89 (t, *J* = 9.1 Hz, 1H), 3.42 (dd, *J* = 9.1, 6.3 Hz, 1H), 2.98 (dd, *J* = 15.3, 6.0 Hz, 1H), 2.85 (dd, *J* = 15.3, 8.4 Hz, 1H). **^{13}C NMR** (100 MHz, CDCl$_3$) δ 169.26, 148.20, 147.92, 140.58, 140.12, 138.23, 136.35, 134.14, 131.38, 127.92, 127.31, 121.75, 121.66, 119.29, 116.53, 52.87, 41.78, 38.50. **HRMS (ESI-TOF)** Calcd for $C_{18}H_{17}N_4O^+$ $[M+H]^+$ calc: 305.1397, found 305.1406.

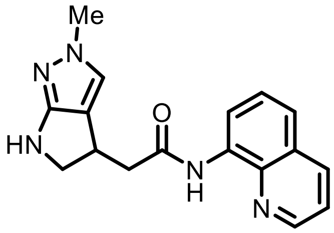

3bs

2-(2-methyl-2,4,5,6-tetrahydropyrrolo[2,3-*c*]pyrazol-4-yl)-*N*-(quinolin-8-yl)acetamide (3bs): To an 8-mL scintillation vial equipped with a Teflon-coated magnetic stir bar were added the Pd(OAc)$_2$ (10.6 mg, 0.047 mmol, 20 mol%), alkene substrate **1a** (50 mg, 0.24 mmol), 4-iodo-1-methyl-1H-pyrazol-3-amine (63 mg, 0.28 mmol, 1.2 equiv), K_2CO_3 (32.6 mg, 0.24 mmol, 1 equiv), and HFIP (0.47 mL). The vial was sealed with a screw-top septum cap and placed in a heating block that was pre-heated to 100 °C. The reaction was allowed to run for 24 h and then was cooled to room temperature. The reaction mixture was then filtered through an autovial syringeless filter device, and concentrated to afford a brown residue, which, upon purification by ISCO automated silica gel column chromatography using an eluent system of 0-10% 7N NH_3 in MeOH/DCM, afforded pure product as a colorless oil (60 mg, 83% yield). **^{1}H NMR** (400 MHz, Chloroform-*d*) δ 9.77 (s, 1H), 8.80–8.73 (m, 2H), 8.15 (dd, *J* = 8.3, 1.7 Hz, 1H), 7.58–7.47 (m, 2H), 7.44 (dd, *J* = 8.3, 4.2 Hz, 1H), 6.88 (d, *J* = 0.7 Hz, 1H), 4.08 (dd, *J* = 9.6, 8.4 Hz, 1H), 3.86–3.76 (m, 1H), 3.63 (s, 3H), 3.61–3.55 (m, 1H), 2.87–2.71 (m, 2H). **^{13}C NMR** (100 MHz, CDCl$_3$) δ 170.10, 165.51, 148.17, 138.31, 136.38, 134.38,127.97, 127.38, 125.62, 121.67, 121.61, 116.51, 113.01, 58.58, 43.46, 38.63, 33.94. **HRMS (ESI-TOF)** Calcd for $C_{17}H_{18}N_5O^+$ $[M+H]^+$ calc: 308.1506, found 308.1502

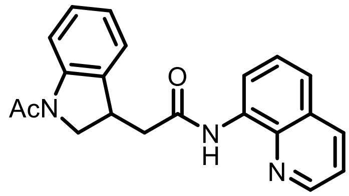

3bv

2-(1-acetylindolin-3-yl)-*N*-(quinolin-8-yl)acetamide (3bv): To a 4-mL scintillation vial equipped with a Teflon-coated magnetic stir bar were added the $Pd(OAc)_2$ (1.1 mg, 0.005 mmol, 5 mol%), alkene substrate **1a** (21.2 mg, 0.1 mmol), *N*-(2-iodophenyl)acetamide (31.3 mg, 0.12 mmol, 1.2 equiv), $KHCO_3$ (10.0 mg, 0.1 mmol, 1 equiv), and HFIP (0.05 mL). The vial was sealed with a screw-top septum cap and placed in a heating block that was pre-heated to 80°C. The reaction was allowed to run for 24 h and then was cooled to room temperature. The reaction mixture was then filtered through a short plug of Celite, and concentrated to afford a brown residue, which, upon purification by preparative TLC with 1:1 hexanes:EtOAc as the eluent, afforded pure product as a yellow solid (24.8 mg, 72% yield).**^{1}H NMR** (600 MHz, $CDCl_3$) δ 9.87 (s, 1H), 8.82–8.75 (m, 2H), 8.24 (d, *J* = 8.0 Hz, 1H), 8.18 (dd, *J* = 8.2, 1.7 Hz, 1H), 7.60–7.52 (m, 2H), 7.47 (dd, *J* = 8.3, 4.2 Hz, 1H), 7.25–7.21 (m, 2H), 7.06–7.02 (m, 1H), 4.42 (dd, *J* = 10.9, 9.4 Hz, 1H), 4.07 (tt, *J* = 9.9, 5.2 Hz, 1H), 3.91 (dd, *J* = 10.8, 5.8 Hz, 1H), 3.12 (dd, *J* = 15.6, 4.7 Hz, 1H), 2.85 (dd, *J* = 15.6, 10.2 Hz, 1H), 2.24 (s, 3H). **^{13}C NMR** (150 MHz, $CDCl_3$) δ 169.48, 169.00, 148.44, 142.85, 138.41, 136.58, 134.24, 133.58, 128.51, 128.12, 127.50, 123.93, 123.87, 122.05, 121.92, 117.30, 116.69, 55.42, 43.59, 37.06, 24.46. **HRMS (ESI-TOF)** Calcd for $C_{21}H_{20}N_3O_2^+$ $[M+H]^+$ 346.1556, found 346.1553.

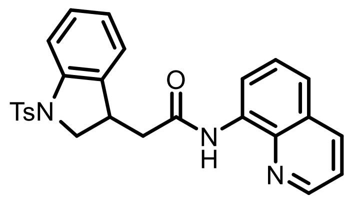

3bt

***N*-(quinolin-8-yl)-2-(1-tosylindolin-3-yl)acetamide (3bt)**: To a 4-mL scintillation vial equipped with a Teflon-coated magnetic stir bar were added the $Pd(OAc)_2$ (1.1 mg, 0.005 mmol, 5 mol%), alkene substrate **1a** (21.2 mg, 0.1 mmol), *N*-(2-iodophenyl)-4-methylbenzenesulfonamide (44.8 mg, 0.12 mmol, 1.2 equiv), K_2CO_3 (13.8 mg, 0.1 mmol, 1 equiv), and HFIP (0.1 mL). The vial was sealed with a screw-top septum cap and placed in a heating block that was pre-heated to 80 °C. The reaction was allowed to run for 24 h and then was cooled to room temperature. The reaction mixture was then filtered through a short plug of Celite, and concentrated to afford a brown residue, which, upon purification by preparative TLC, with 1:1 hexanes:EtOAc as the eluent, afforded pure product as a white solid (45.8 mg, >99% yield).**^{1}H NMR** (600 MHz, $CDCl_3$) δ 9.64 (s, 1H), 8.79 (dd, *J* = 4.2, 1.7 Hz, 1H), 8.77 (s, 1H), 8.18 (dd, J = 8.3, 1.7 Hz, 1H), 7.72–7.67 (m, 3H), 7.59–7.51 (m, 2H), 7.47 (dd, *J* = 8.3, 4.2 Hz, 1H), 7.26–7.21 (m, 3H), 7.16–7.13 (m, 1H), 6.99 (td, *J* = 7.5, 1.0 Hz, 1H), 4.21–4.14 (m, 1H), 3.84–3.75 (m, 2H), 2.72–2.65 (m, 1H), 2.38 (s, 3H), 2.37–2.33 (m, 1H). **^{13}C NMR** (150 MHz, $CDCl_3$) δ 169.05, 148.32, 144.34, 141.90, 138.36, 136.59, 134.46, 134.06, 129.87, 128.60, 128.10, 127.51, 124.72, 124.18, 121.99, 121.87, 116.72, 115.54, 55.84, 43.25, 36.88, 21.70. **HRMS (ESI-TOF)** Calcd for $C_{26}H_{24}N_3O_3S^+$ $[M+H]^+$ 458.1538, found 458.1537.

2-(5-methoxy-1-tosylindolin-3-yl)-*N*-(quinolin-8-yl)acetamide (3bu): To a 4-mL scintillation vial equipped with a Teflon-coated magnetic stir bar were added the $Pd(OAc)_2$ (1.1 mg, 0.005 mmol, 5 mol%), alkene substrate **1a** (21.2 mg, 0.1 mmol), *N*-(2-iodo-4-methoxyphenyl)-4-methylbenzenesulfonamide (48.3 mg, 0.12 mmol, 1.2 equiv), K_2CO_3 (13.8 mg, 0.1 mmol, 1 equiv), and HFIP (0.1 mL). The vial was sealed with a screw-top septum cap and placed in a heating block that was pre-heated to 80 °C. The reaction was allowed to run for 24 h and then was cooled to room temperature. The reaction mixture was then filtered through a short plug of Celite, and concentrated to afford a brown residue, which, upon purification by preparative TLC, with 1:1

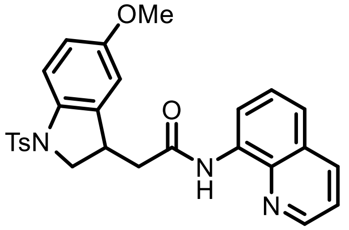

3bu

hexanes:EtOAc as the eluent, afforded pure product as a white solid (48.8 mg, >99% yield).**^{1}H NMR** (600 MHz, $CDCl_3$) δ 9.58 (s, 1H), 8.80 (dd, *J* = 4.2, 1.7 Hz, 1H), 8.74 (dd, *J* = 6.7, 2.3 Hz, 1H), 8.18 (dd, *J* = 8.3, 1.7 Hz, 1H), 7.67–7.64 (m, 2H), 7.62 (d, *J* = 8.8 Hz, 1H), 7.58–7.51 (m, 2H), 7.48 (dd, *J* = 8.3, 4.2 Hz, 1H), 7.25–7.24 (m, 2H), 6.78 (ddd, *J* = 8.8, 2.7, 0.7 Hz, 1H), 6.68 (dd, *J* = 2.6, 0.9 Hz, 1H), 4.18 (dd, *J* = 11.7, 8.9 Hz, 1H), 3.81–3.75 (m, 1H), 3.69 (m, 4H), 2.56 (dd, *J* = 15.4, 5.6 Hz, 1H), 2.40 (s, 3H), 2.21 (dd, *J* = 15.4, 9.2 Hz, 1H). **^{13}C NMR** (150 MHz, $CDCl_3$) δ 168.99, 157.15, 148.32, 144.24, 138.38, 136.62, 136.40, 135.30, 134.21, 133.96, 129.85, 128.12, 127.61, 127.53, 122.02, 121.89, 117.15, 116.75, 113.88, 110.35, 56.17, 55.72, 43.14, 37.28, 21.73. **HRMS (ESI-TOF)** Calcd for $C_{27}H_{26}N_3O_4S^+$ $[M+H]^+$ 488.1644, found 488.1638.

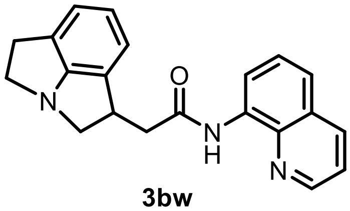

3bw

***N*-(quinolin-8-yl)-2-(1,2,4,5-tetrahydropyrrolo[3,2,1-hi]indol-1-yl)acetamide (3bw):** To an 4-mL scintillation vial equipped with a Teflon-coated magnetic stir bar were added the $Pd(OAc)_2$ (2.6 mg, 0.0118 mmol, 5 mol%), alkene substrate **1a** (50 mg, 0.24 mmol), 7-bromoindoline (56 mg, 0.28 mmol, 1.2 equiv), K_2CO_3 (32.6 mg, 0.236 mmol, 1 equiv), and HFIP (0.48 mL). The vial was sealed with a screw-top septum cap and placed in a heating block that was pre-heated to 80 °C. The reaction was allowed to run for 24 h and then was cooled to room temperature. The reaction mixture was then filtered through an autovial syringeless filter device, and concentrated to afford a brown residue, which, upon purification by ISCO automated silica gel column chromatography using an eluent system of 0–100% EtOAc/heptane, afforded pure product as a light brown film (70 mg, 90% yield). **^{1}H NMR** (400 MHz, $CDCl_3$) δ = 9.88 (br s, 1H), 8.83 (dd, *J* = 1.5, 7.3 Hz, *1*H), 8.78 (dd, *J* = 1.7, 4.2 Hz, 1H), 8.17 (dd, *J* = 1.7, 8.3 Hz, 1H), 7.60–7.50 (m, 2H), 7.45 (dd, *J* = 4.2, 8.3 Hz, 1H), 7.02–6.94 (m, 2H), 6.65 (t, *J* = 7.3 Hz, 1H), 4.30 (quin, *J* = 7.3 Hz, 1H), 3.61 (dd, *J* = 7.3, 8.7 Hz, 1H), 3.37–3.15 (m, 4H), 3.12–3.01 (m, 2H), 2.92 (dd, *J* = 8.1, 15.0 Hz, 1H) **^{13}C NMR** (100 MHz, $CDCl_3$) δ = 169.87, 163.97, 148.13, 138.32, 136.29, 134.38, 127.92, 127.37, 125.35, 123.48, 122.75, 121.67, 121.59, 121.54, 120.32, 116.51, 64.44, 58.68, 45.06, 41.77, 34.74. **HRMS (ESI-TOF)** Calcd for $C_{21}H_{20}N_3O^+$ $[M+H]^+$ 330.16009, found 330.16201.

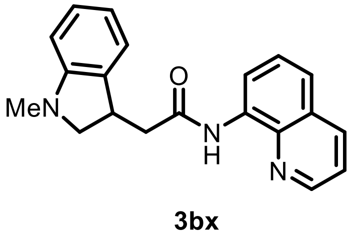

3bx

2-(1-methylindolin-3-yl)-*N*-(quinolin-8-yl)acetamide (3bx): To a 4-mL scintillation vial equipped with a Teflon-coated magnetic stir bar were added the $Pd(OAc)_2$ (2.2 mg, 0.01 equiv, 10 mol%), alkene substrate **1a** (21.2 mg, 0.1 mmol), 2-iodo-*N*-methylaniline (28.0 mg, 0.12 mmol, 1.2 equiv), K_2CO_3 (13.8 mg, 0.1 mmol, 1 equiv), and HFIP (0.1 mL). The vial was sealed with a screw-top septum cap and placed in a heating block that was pre-heated to 80°C. The reaction was allowed to run for 24 h and then was cooled to room temperature. The reaction mixture was then filtered through a short plug of Celite, and concentrated to afford a brown residue, which, upon purification by preparative TLC, with 1:1 hexanes:EtOAc as the eluent, afforded pure product as a yellow oil (15.9 mg, 50% yield).**^{1}H NMR** (600 MHz, $CDCl_3$) δ 9.85 (s, 1H), 8.85–8.79 (m, 1H), 8.79–8.73 (m, 1H), 8.19–8.14 (m, 1H), 7.60–7.54 (m, 1H), 7.53 (d, *J* = 1.5 Hz, 1H), 7.45 (dd, *J* = 8.2, 4.2 Hz, 1H), 7.23–7.09 (m, 2H), 6.68 (td, *J* = 7.4, 1.0 Hz, 1H), 6.52 (d, *J* = 7.8 Hz, 1H), 3.90–3.83 (m, 1H), 3.62 (dd, *J* = 9.0, 8.4 Hz, 1H), 3.19 (dd, *J* = 9.0, 6.3 Hz, 1H), 3.00 (dd, *J* = 15.1, 5.8 Hz, 1H), 2.83 (dd, *J* = 15.1, 9.1 Hz, 1H), 2.77 (s, 3H). **^{13}C NMR** (150 MHz,

$CDCl_3$) δ 170.32, 153.14, 148.32, 138.47, 136.49, 134.54, 132.59, 128.22, 128.09, 127.56, 123.86, 121.79, 121.72, 118.08, 116.65, 107.52, 62.06, 42.61, 37.75, 36.07. **HRMS (ESI-TOF)** Calcd for $C_{20}H_{20}N_3O^+$ $[M+H]^+$ 318.1606, found 318.1602.

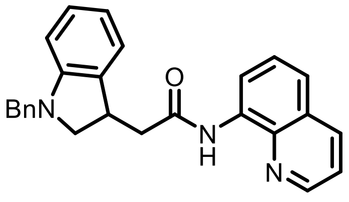

3by

2-(1-benzylindolin-3-yl)-*N*-(quinolin-8-yl)acetamide (3by): To a 4-mL scintillation vial equipped with a Teflon-coated magnetic stir bar were added the $Pd(OAc)_2$ (2.2 mg, 0.01 equiv, 10 mol%), alkene substrate **1a** (21.2 mg, 0.1 mmol), N-benzyl-2-iodoaniline (37.1 mg, 0.12 mmol, 1.2 equiv), K_2CO_3 (13.8 mg, 0.1 mmol, 1 equiv), and HFIP (0.1 mL). The vial was sealed with a screw-top septum cap and placed in a heating block that was pre-heated to 80 °C. The reaction was allowed to run for 24 h and then was cooled to room temperature. The reaction mixture was then filtered through a short plug of Celite, and concentrated to afford a brown residue, which, upon purification by preparative TLC, with 1:1 hexanes:EtOAc as the eluent, afforded pure product as a yellow oil (22.8 mg, 58% yield).**^{1}H NMR** (600 MHz, CDCl3) δ 9.82 (s, 1H), 8.82–8.75 (m, 2H), 8.16 (dd, *J* = 8.2, 1.7 Hz, 1H), 7.54 (s, 1H), 7.52 (d, *J* = 1.5 Hz, 1H), 7.45 (dd, *J* = 8.2, 4.2 Hz, 1H), 7.38–7.34 (m, 2H), 7.32–7.28 (m, 2H), 7.26–7.22 (m, 1H), 7.17 (dd, *J* = 7.4, 1.3 Hz, 1H), 7.12–7.07 (m, 1H), 6.68 (td, *J* = 7.4, 0.9 Hz, 1H), 6.54 (d, *J* = 7.9 Hz, 1H), 4.35–4.21 (m, 2H), 3.95–3.87 (m, 1H), 3.68 (t, *J* = 8.9 Hz, 1H), 3.19 (dd, *J* = 9.1, 6.5 Hz, 1H), 3.03 (dd, *J* = 15.2, 5.8 Hz, 1H), 2.82 (dd, *J* = 15.1, 8.9 Hz, 1H). **^{13}C NMR** (150 MHz, CDCl3) δ 170.22, 152.29, 148.30, 138.45, 138.36, 136.49, 134.50, 132.30, 128.65, 128.21, 128.08, 128.00, 127.55, 127.28, 124.07, 121.77, 121.71, 118.00, 116.63, 107.38, 59.71, 53.39, 42.87, 37.59. **HRMS (ESI-TOF)** Calcd for $C_{26}H_{24}N_3O^+$ $[M+H]^+$ 394.1919, found 394.1913.

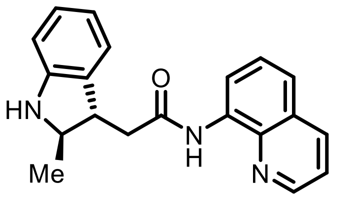

(±)-4gb
d.r. = 4:1

2-(2-methylindolin-3-yl)-*N*-(quinolin-8-yl)acetamide ((±)-4gb): The title compound was prepared from **1g** (22.6 mg, 0.1 mmol) and 2-iodoaniline (26.3 mg, 0.12 mmol) according to the General Procedure B. Purification using preparative TLC with 1:1 hexanes:EtOAc as the eluent gave the product as a yellow solid (23.5 mg, 74% yield).). The reported *d.r.* was determined by ^{1}H NMR analysis of purified **(±)-4gb** and is consistent with that of the crude reaction mixture. The following analytical data correspond to the major diastereomer. **^{1}H NMR** (600 MHz, $CDCl_3$) δ 9.85 (s, 1H), 8.83 (dd, *J* = 7.5, 1.5 Hz, 1H), 8.76 (dd, *J* = 4.2, 1.7 Hz, 1H), 8.16 (dd, *J* = 8.2, 1.7 Hz, 1H), 7.56 (t, *J* = 7.9 Hz, 1H), 7.52 (dd, *J* = 8.3, 1.4 Hz, 1H), 7.45 (dd, *J* = 8.2, 4.2 Hz, 1H), 7.16 (dd, *J* = 7.4, 1.3 Hz, 1H), 7.04 (tt, *J* = 7.7, 1.0 Hz, 1H), 6.69 (td, *J* = 7.4, 1.0 Hz, 1H), 6.63 (d, *J* = 7.8 Hz, 1H), 3.80 (br, 1H), 3.77 (p, *J* = 6.2 Hz, 1H), 3.55 (q, *J* = 6.7 Hz, 1H), 2.92 (dd, *J* = 15.0, 6.6 Hz, 1H), 2.83 (dd, *J* = 15.0, 7.8 Hz, 1H), 1.33 (d, *J* = 6.2 Hz, 3H). **^{13}C NMR** (150 MHz, $CDCl_3$) δ 170.18, 150.18, 148.32, 138.48, 136.48, 134.52, 131.12, 128.10, 127.56, 124.62, 121.78, 121.73, 118.93, 116.67, 109.59, 60.98, 46.40, 42.92, 21.99. **HRMS (ESI-TOF)** Calcd for $C_{20}H_{20}N_3O^+$ $[M+H]^+$ 318.1606, found 318.1611.

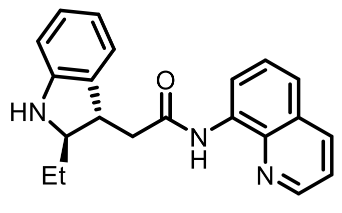

(±)-4hb
d.r. > 20:1

2-(2-Ethylindolin-3-yl)-*N*-(quinolin-8-yl)acetamide ((±)-4hb): The title compound was prepared from **1h** and 2-iodoaniline (26.3 mg, 0.12 mmol) according to the General Procedure B. Purification using preparative TLC with 1:1 hexanes:EtOAc as the eluent gave the product as a yellow solid (17.9 mg, 54% yield). **^{1}H NMR** (600 MHz, $CDCl_3$) δ 9.83 (s, 1H), 8.83 (dd, *J* = 7.6, 1.4 Hz, 1H), 8.76 (dd, *J* = 4.2, 1.7 Hz, 1H), 8.16 (dd, *J* = 8.2, 1.7 Hz, 1H), 7.56 (t, *J* = 7.9 Hz, 1H), 7.52 (dd, *J* = 8.3, 1.4 Hz, 1H), 7.44 (dd, *J* = 8.2, 4.2 Hz, 1H), 7.18–7.14 (m, 1H), 7.06–7.00 (m, 1H), 6.67 (td, *J* = 7.4, 1.0 Hz, 1H), 6.63 (d, *J* = 7.8 Hz, 1H), 3.91 (br, 1H), 3.63 (q, *J* = 6.6 Hz, 1H), 3.56 (dt, *J* = 7.7, 5.2 Hz, 1H), 2.90 (dd, J = 14.9, 6.7 Hz, 1H), 2.84 (dd, J = 14.9, 7.6 Hz, 1H), 1.75–1.65 (m, 1H), 1.64–1.53 (m, 1H), 0.98 (t, *J* = 7.4 Hz, 3H). **^{13}C NMR** (150 MHz, $CDCl_3$) δ 170.18, 150.29, 148.31, 138.49, 136.47, 134.55, 131.20, 128.07, 127.57, 124.65, 121.77, 121.70, 118.75, 116.67, 109.37, 66.77, 44.43, 43.53, 28.96, 10.58. **HRMS (ESI-TOF)** Calcd for $C_{21}H_{22}N_3O^+$ $[M+H]^+$ 332.1763, found 332.1766.

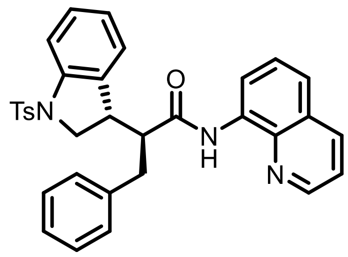

(±)-4cp
d.r. >20:1

3-Phenyl-*N*-(quinolin-8-yl)-2-(1-tosylindolin-3-yl)propanamide ((±)-4cp): To a 4-mL scintillation vial equipped with a Teflon-coated magnetic stir bar were added the $Pd(OAc)_2$ (1.1 mg, 0.005 mmol, 5 mol%), **1c** (30.2 mg, 0.1 mmol, 1 equiv), the appropriate *ortho*-iodoaniline substrate (mg, 0.12 mmol, 1.2 equiv), K_2CO_3 (13.8 mg, 0.1 mmol, 1 equiv), and HFIP (0.1 mL). The vial was sealed with a screw-top septum cap and placed in a heating block that was pre-heated to 80°C The reaction was allowed to run for 24 h and then was cooled to room temperature. The reaction mixture was then filtered through a short plug of Celite, and concentrated to afford a brown residue, which, upon purification by preparative TLC, with 1:1 hexanes:EtOAc as the eluent, afforded pure product as a white solid (46.5 mg, 85% yield). **^{1}H NMR** (600 MHz, $CDCl_3$) δ 9.15 (s, 1H), 8.71 (dd, *J* = 7.4, 1.5 Hz, 1H), 8.61 (dd, *J* = 4.2, 1.6 Hz, 1H), 8.10 (dd, *J* = 8.2, 1.7 Hz, 1H), 7.78–7.71 (m, 3H), 7.54–7.45 (m, 2H), 7.38 (dd, J = 8.2, 4.2 Hz, 1H), 7.28–7.27 (m, 2H), 7.20–7.08 (m, 4H), 7.07–7.01 (m, 3H), 6.79 (td, *J* = 7.5, 1.0 Hz, 1H), 4.17–4.05 (m, 2H), 3.69 (td, *J* = 7.9, 5.7 Hz, 1H), 2.89 (dd, *J* = 13.7, 11.1 Hz, 1H), 2.54 (dd, *J* = 13.7, 3.2 Hz, 1H), 2.45 (ddd, *J* = 10.9, 7.6, 3.1 Hz, 1H), 2.31 (s, 3H). **^{13}C NMR** (150 MHz, $CDCl_3$) δ 171.02, 147.36, 143.90, 141.73, 138.19, 137.73, 135.69, 133.53, 133.37, 132.05, 129.43, 128.26, 128.24, 127.99, 127.31, 126.87, 126.77, 125.93, 124.80, 123.43, 121.33, 121.06, 116.14, 114.74, 54.40, 52.33, 42.46, 35.05, 21.04. **HRMS (ESI-TOF)** Calcd for $C_{33}H_{30}N_3O_3S^+$ $[M+H]^+$ 548.2008, found 548.2014. **X-ray** (single-crystal) Colorless needle crystals of X-ray diffraction quality were obtained by vapor diffusion of hexane into a saturated solution of **4cp** in toluene (CCDC 1999889).[16]

Note: The diastereomeric ratios of products **4ca-4fa, 4hb** and **4cp** were determined directly based on ^{1}H NMR spectra of the crude reaction mixtures. In these cases, based on our analysis, only a single product diastereomer was detected. Trace impurities were sometimes detected in the ^{1}H NMR spectra of the crude reaction mixture, but the peaks of these minor byproducts did not appear to correspond to the other potential diastereomer. However, except for the major product, no other compounds were formed in sufficient quantity to be isolated and characterized. Based on this data (namely, that the other potential diastereomers were not observed), we have conservatively reported a lower bound of 20:1 for the diastereomeric ratio in these cases.

General Procedure C for [3+2] Indane Synthesis

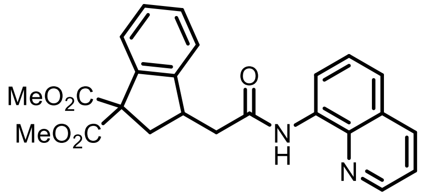

6a

Dimethyl 3-(2-oxo-2-(quinolin-8-ylamino)ethyl)-2,3-dihydro-1*H*-indene-1,1-dicarboxylate (6a): To a 4-mL scintillation vial equipped with a Teflon-coated magnetic stir bar were added the $Pd(OAc)_2$ (2.2 mg, 0.01 mmol, 10 mol%), alkene substrate **1a** (21.2 mg, 0.1 mmol), the carbon nucleophile (40 mg, 0.12 mmol, 1.2 equiv), K_2CO_3 (13.8 mg, 0.1 mmol, 1 equiv), and HFIP (0.05 mL). The vial was sealed with a screw-top septum cap and placed in a heating block that was pre-heated to 40 °C. The reaction was allowed to run for 24 h and then was cooled to room temperature. The reaction mixture was then filtered through a short plug of Celite, and concentrated to afford a brown residue, which, upon purification by preparative TLC, afforded pure product as a colorless oil (40.1 mg, 96% yield). **^{1}H NMR** (600 MHz, $CDCl_3$) δ 9.90 (s, 1H), 8.83 (dd, *J* = 7.5, 1.5 Hz, 1H), 8.78 (dd, *J* = 4.2, 1.7 Hz, 1H), 8.17 (dd, *J* = 8.2, 1.7 Hz, 1H), 7.66–7.48 (m, 3H), 7.45 (dd, *J* = 8.2, 4.2 Hz, 1H), 7.35–7.28 (m, 3H), 4.02 (tt, *J* = 8.4, 6.4 Hz, 1H), 3.80 (s, 3H), 3.73 (s, 3H), 3.13 (dd, *J* = 13.7, 8.1 Hz, 1H), 3.06 (dd, *J* = 14.9, 5.9 Hz, 1H), 2.79 (dd, *J* = 14.9, 9.0 Hz, 1H), 2.56 (dd, *J* = 13.7, 6.9 Hz, 1H). **^{13}C NMR** (150 MHz, $CDCl_3$) δ 171.44, 171.09, 170.13, 148.33, 146.46, 139.00, 138.49, 136.50, 134.54, 129.21, 128.09, 127.57, 127.55, 126.94, 124.04, 121.79, 121.77, 116.73, 64.83, 53.12 (d, *J* = 5.0 Hz), 44.00, 40.57, 39.93. **HRMS (ESI-TOF)** Calcd for $C_{24}H_{23}N_2O_5^+$ $[M+H]^+$ 419.1607, found 419.1607.

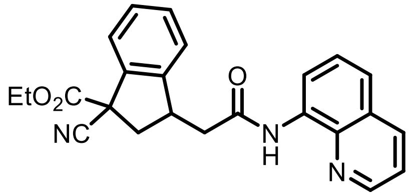

(±)-6b

Ethyl 1-cyano-3-(2-oxo-2-(quinolin-8-ylamino)ethyl)-2,3-dihydro-1*H*-indene-1-carboxylate ((±)-6b): To a 4-mL scintillation vial equipped with a Teflon-coated magnetic stir bar were added the $Pd(OAc)_2$ (2.2 mg, 0.01 mmol, 10 mol%), alkene substrate **1a** (21.2 mg, 0.1 mmol), the carbon nucleophile (37.8 mg, 0.12 mmol, 1.2 equiv), K_2CO_3 (13.8 mg, 0.1 mmol, 1 equiv), and HFIP (0.05 mL). The vial was sealed with a screw-top septum cap and placed in a heating block that was pre-heated to 40 °C. The reaction was allowed to run for 24 h and then was cooled to room temperature. The reaction mixture was then filtered through a short plug of Celite, and concentrated to afford a brown residue, which, upon purification by preparative TLC, afforded pure product as a colorless oil (34.3 mg, 86% yield). The reported *d.r.* was determined by ^{1}H NMR analysis of purified **(±)-6b** and is consistent with that of the crude reaction mixture. The following analytical data correspond to the mixture. **^{1}H NMR** δ 9.91 (s, 2.5H), 8.84–8.76 (m, 5H), 8.20–8.15 (m, 2.5H), 7.61–7.50 (m, 7.5H), 7.46 (ddd, *J* = 8.2, 4.2, 2.8 Hz, 2.5H), 7.40–7.36 (m, 5H), 7.36–7.30 (m, 2.5H), 4.39–4.31 (m, 2H), 4.30–4.22 (m, 3H), 4.22–4.11 (m, 2.5H), 3.36 (dd, *J* = 13.4, 7.7 Hz, 1.5H), 3.15 (dd, *J* = 15.2, 6.1 Hz, 1.5H), 3.12–3.02 (m, 2H), 2.96–2.91 (m, 1H), 2.89–2.76 (m, 2.5H), 2.51 (dd, *J* = 13.4, 7.7 Hz, 1.5H), 1.38 (t, *J* = 7.1 Hz, 3H), 1.30 (t, *J* = 7.1 Hz, 4.5H). **^{13}C NMR** (150 MHz, $CDCl_3$) δ 169.67, 169.36, 168.07, 167.47, 148.43, 148.36, 145.79, 145.60, 138.48, 138.44, 137.97, 137.80, 136.55, 136.54, 134.43, 134.36, 130.32, 130.23, 128.58, 128.48, 128.11, 128.10, 127.56, 127.52, 124.97, 124.81, 124.77, 124.74, 121.95, 121.91, 121.87, 121.83, 119.83, 119.37, 116.77, 116.73,, 63.62, 63.41, 52.22, 51.86, 43.67, 43.34, 42.99, 41.72, 40.37, 40.31, 14.18, 14.09. **HRMS (ESI-TOF)** Calcd for $C_{24}H_{22}N_3O_3^+$ $[M+H]^+$ 410.1661, found 410.1660.

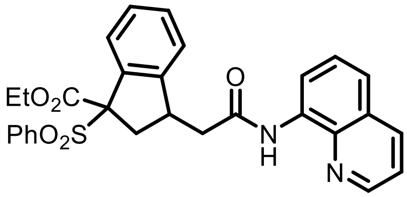

(±)-6c

Ethyl 3-(2-oxo-2-(quinolin-8-ylamino)ethyl)-1-(phenylsulfonyl)-2,3-dihydro-1H-indene-1-carboxylate ((±)-6c): To a 4-mL scintillation vial equipped with a Teflon-coated magnetic stir bar were added the $Pd(OAc)_2$ (2.2 mg, 0.01 mmol, 10 mol%), alkene substrate **1a** (21.2 mg, 0.1 mmol), the carbon nucleophile (51.6 mg, 0.12 mmol, 1.2 equiv), K_2CO_3 (13.8 mg, 0.1 mmol, 1 equiv), and HFIP (0.05 mL). The vial was sealed with a screw-top septum cap and placed in a heating block that was pre-heated to 60 °C. The reaction was allowed to run for 24 h and then was cooled to room temperature. The reaction mixture was then filtered through a short plug of Celite, and concentrated to afford a brown residue, which, upon purification by preparative TLC, afforded pure product as a colorless oil (31.9 mg, 62% yield). The reported *d.r.* was determined by ^{1}H NMR analysis of purified **(±)-6c** and is consistent with that of the crude reaction mixture. The following analytical data correspond to the mixture. **^{1}H NMR** (600 MHz, $CDCl_3$) δ 9.86 (s, 2.3H), 8.88–8.81 (m, 2H), 8.81–8.71 (m, 2.6H), 8.18 (ddd, *J* = 11.9, 8.3, 1.7 Hz, 2.3H), 7.77–7.72 (m, 2H), 7.69–7.62 (m, 2.3H), 7.62–7.56 (m, 6.9H), 7.56–7.51 (m, 2.6H), 7.50–7.45 (m, 2.3H), 7.45–7.40 (m, 4.6H), 7.38 (tdd, *J* = 7.5, 6.2, 1.2 Hz, 2.3H), 7.33–7.27 (m, 2.6H), 7.27–7.21 (m, 2H), 4.38–4.23 (m, 2.6H), 4.23–4.13 (m, 2H), 4.01 (dp, *J* = 9.7, 4.9 Hz, 1H), 3.70 (qd, *J* = 8.4, 5.6 Hz, 1.3H), 3.43–3.33 (m, 2.6H), 3.06 (dd, *J* = 15.2, 5.6 Hz, 1.3H), 2.94 (dd, *J* = 15.0, 5.6 Hz, 1H), 2.80–2.59 (m, 4.6H), 1.29 (t, *J* = 7.1 Hz, 3.9H), 1.24 (t, *J* = 7.1 Hz, 3H). **^{13}C NMR** (150 MHz, $CDCl_3$) δ 170.33, 169.52, 167.03, 166.95, 148.39, 148.34, 148.32, 138.52, 138.43, 136.55, 136.52, 136.23, 135.84, 134.71, 134.53, 134.46, 134.33, 134.24, 131.10, 130.84, 130.58, 130.55, 128.70, 128.61, 128.52, 128.36, 128.12, 128.09, 127.55, 127.54, 127.41, 127.32, 124.46, 124.07, 121.86, 121.84, 121.83, 121.81, 116.74, 83.58, 82.89, 77.37, 77.16, 76.95, 62.99, 62.95, 44.07, 43.76, 39.52, 39.14, 38.77, 36.72. **HRMS (ESI-TOF)** Calcd for $C_{29}H_{27}N_2O_5S^+$ $[M+H]^+$ 515.1641, found 515.1647.

General Procedure for [3+2] Reactions with NHPA Directing groups

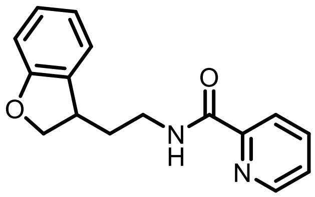

4ia

***N*-(2-(2,3-Dihydrobenzofuran-3-yl)ethyl)picolinamide (4ia)**: To a 4-mL scintillation vial equipped with a Teflon-coated magnetic stir bar were added the $Pd(OAc)_2$ (2.2 mg, 0.01 mmol, 10 mol%), N-(but-3-en-1-yl)picolinamide (17.6 mg, 0.1 mmol), the 2-iodophenol (26.4 mg, 0.12 mmol, 1.2 equiv), K_2CO_3 (13.8 mg, 0.1 mmol, 1 equiv), and HFIP (0.1 mL). The vial was sealed with a screw-top septum cap and placed in a heating block that was pre-heated to 80 °C. The reaction was allowed to run for 24 h and then was cooled to room temperature. The reaction mixture was then filtered through a short plug of Celite, and concentrated to afford a brown residue, which, upon purification by preparative TLC, with 3:1 hexanes:EtOAc as the eluent, afforded pure product as a yellow oil (17.7 mg, 66% yield). **^{1}H NMR** (600 MHz, $CDCl_3$) δ 8.55 (ddd, *J* = 4.7, 1.7, 0.9 Hz, 1H), 8.20 (dt, *J* = 7.9, 1.1 Hz, 1H), 8.13 (s, 1H), 7.86 (td, *J* = 7.7, 1.7 Hz, 1H), 7.43 (ddd, *J* = 7.6, 4.8, 1.3 Hz, 1H), 7.25–7.21 (m, 1H), 7.18–7.08 (m, 1H), 6.87 (td, *J* = 7.4, 1.0 Hz, 1H), 6.80 (dd, *J* = 8.0, 0.9 Hz, 1H), 4.69 (t, *J* = 8.8 Hz, 1H), 4.31 (dd, *J* = 8.9, 6.2 Hz, 1H), 3.68–3.49 (m, 3H), 2.16–2.06 (m, 1H), 1.97–1.88 (m, 1H). **^{13}C NMR** (150 MHz, $CDCl_3$) δ 164.61, 159.97, 149.92, 148.21, 137.46, 130.26, 128.52, 124.53, 122.29, 120.67, 109.81, 76.79, 39.83, 37.38, 35.05. **HRMS (ESI-TOF)** Calcd for $C_{16}H_{17}N_2O_2^+$ $[M+H]^+$ 269.1290, found 269.1295.

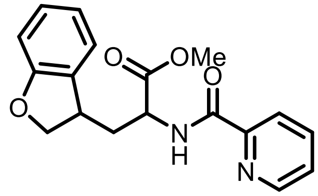

(±)-4ja'
d.r. = 1.06:1

Methyl 3-(2,3-dihydrobenzofuran-3-yl)-2-(picolinamido)propanoate ((±)-4ja'): To a 4-mL scintillation vial equipped with a Teflon-coated magnetic stir bar were added the $Pd(OAc)_2$ (4.4 mg, 0.02 equiv, 20 mol%), methyl-2-(picolinamido)pent-4-enoate (23.4 mg, 0.1 mmol), the 2-iodophenol (26.4 mg, 0.12 mmol, 1.2 equiv), K_2CO_3 (13.8 mg, 0.1 mmol, 1 equiv), and HFIP (0.1 mL). The vial was sealed with a screw-top septum cap and placed in a heating block that was pre-heated to 80 °C. The reaction was allowed to run for 24 h and then was cooled to room temperature. The reaction mixture was then filtered through a short plug of Celite, and concentrated to afford a brown residue, which, upon purification by preparative TLC, with 1:1 hexanes:EtOAc as the eluent, afforded pure product as a yellow oil (16.3 mg, 50% yield). The reported *d.r.* was determined by ^{1}H NMR analysis of purified **(±)-4ja'** and is consistent with that of the crude reaction mixture. The following analytical data correspond to the major diastereomer. The purified sample was diluted with MeCN to approximately 0.1 mg/mL and run on 1D chiral-SFC to determine *ee*. **^{1}H NMR** (600 MHz, $CDCl_3$) δ 8.67–8.51 (m, 4.12H), 8.23–8.15 (m, 2.06H), 7.87 (tdd, *J* = 7.7, 2.5, 1.7 Hz, 2.06H), 7.47 (ddt, *J* = 7.6, 4.8, 1.3 Hz, 2.06H), 7.35 (dd, *J* = 7.4, 1.3 Hz, 1H), 7.16 (dd, *J* = 7.4, 1.4 Hz, 1.06H), 7.15–7.10 (m, 2.06H), 6.88 (td, *J* = 7.4, 1.0 Hz, 1H), 6.85 (td, *J* = 7.5, 1.0 Hz, 1.06H), 6.79 (s, 1.06H), 6.78 (s, 1H), 5.00 (td, *J* = 8.9, 5.4 Hz, 1H), 4.93 (td, *J* = 8.7, 6.3 Hz, 1.06H), 4.73 (t, *J* = 8.9 Hz, 1.06H), 4.64 (t, *J* = 8.8 Hz, 1H), 4.39 (dd, *J* = 9.1, 6.3 Hz, 1.06H), 4.26 (dd, *J* = 8.9, 6.0 Hz, 1H), 3.78 (d, *J* = 3.5 Hz, 6.18H), 3.62–3.51 (m, 2.06H), 2.46 (ddd, *J* = 14.0, 6.5, 5.4 Hz, 1H), 2.28–2.18 (m, 2.12H), 2.07 (ddd, *J* = 14.0, 8.9, 8.1 Hz, 1H). **^{13}C NMR** (150 MHz, $CDCl_3$) δ 171.89, 171.82 163.93, 163.81, 159.37, 159.22, 148.69, 148.65, 147.90, 147.88, 136.98, 129.25, 129.10, 128.14, 128.09, 126.14, 126.13, 124.28, 123.64, 121.96, 120.23, 120.06, 109.26, 109.24, 75.97, 52.22, 52.15, 50.39, 50.21, 38.57, 38.42, 37.91, 37.80. **HRMS (ESI-TOF)** Calcd for $C_{18}H_{19}N_2O_4^+$ $[M+H]^+$ 327.1345, found 327.1342.

SAMPLE INFORMATION

Sample Name:	NH-444	Acquired By:	System
Sample Type:	Unknown	Sample Set Name	Samples_SFC
Vial:	1:F,1	Acq. Method Set:	G_ENG0308
Injection #:	1	Processing Method	ENG0308B
Injection Volume:	1.00 ul	Channel Name:	PDA Ch1 215nm@12.0nm
Run Time:	6.0 Minutes	Proc. Chnl. Descr.:	PDA Ch1 215nm@12.0nm
Date Acquired:	1/22/2020 9:22:51 AM PST		
Date Processed:	1/22/2020 9:43:50 AM PST		

Auto-Scaled Chromatogram

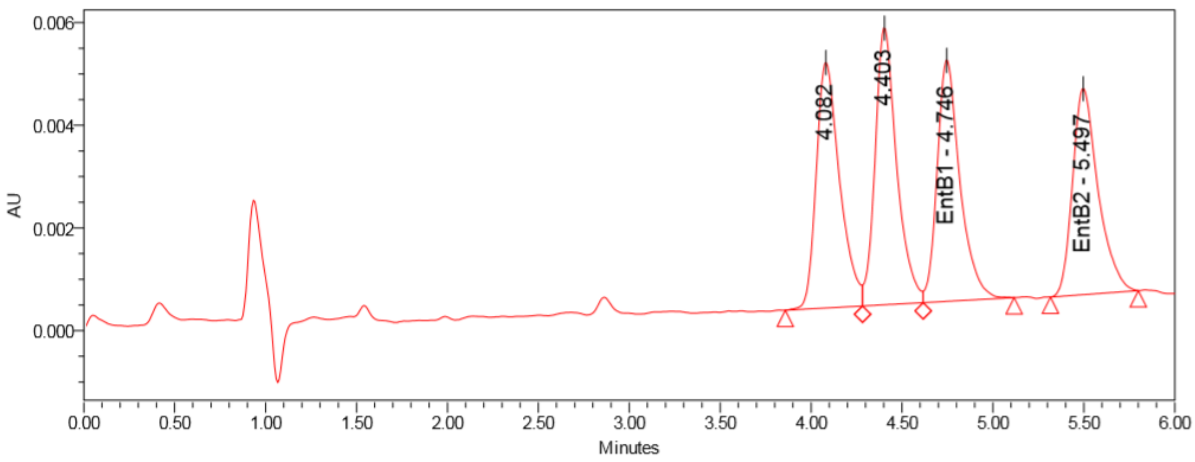

	SampleName	ent1	ent2	ee	EntA1	EntA2
1	NH-444	49.02	50.98	-1.96	42938	44658
2	NH-445	84.21	15.79	68.42	20456	3836
3	NH-446	100.00		100.00	103890	

Supplementary Figure 12: 1D-SFC result for **(±)-4ja'** (NH-444).

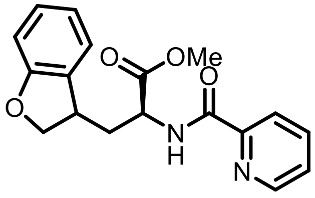

4ja
d.r. = 1.6:1

Methyl (2*S*)-3-(2,3-dihydrobenzofuran-3-yl)-2-(picolinamido)propanoate ((±)-4ja): To a 4-mL scintillation vial equipped with a Teflon-coated magnetic stir bar were added the $Pd(OAc)_2$ (2.2 mg, 0.01 mmol, 10 mol%), methyl (S)-2-(picolinamido)pent-4-enoate (23.4 mg, 0.1 mmol), the 2-iodophenol (26.4 mg, 0.12 mmol, 1.2 equiv), K_2CO_3 (13.8 mg, 0.1 mmol, 1 equiv), and HFIP (0.1 mL). The vial was sealed with a screw-top septum cap and placed in a heating block that was pre-heated to 40 °C. The reaction was allowed to run for 24 h and then was cooled to room temperature. The reaction mixture was then filtered through a short plug of Celite, and concentrated to afford a brown residue, which, upon purification by preparative TLC, with 1:1 hexanes:EtOAc, afforded pure product as a yellow oil (19.6 mg, 60% yield). The reported *d.r.* was determined by ^{1}H NMR analysis of purified **(±)-4ja** and is consistent with that of the crude reaction mixture. The following analytical data correspond to the mixture. The purified sample was diluted with MeCN to approximately 0.1 mg/mL and run on 1D chiral-SFC to determine *ee*. **^{1}H NMR** (500 MHz, $CDCl_3$) δ 8.65–8.54 (m, 5.06H), 8.21 (dd, *J* = 12.5, 7.8 Hz, 2.53H), 7.89 (tt, *J* = 7.7, 2.0 Hz, 2.53H), 7.49 (ddt, *J* = 7.7, 4.8, 1.0 Hz, 2.53H), 7.37 (d, *J* = 7.4 Hz, 1H), 7.21–7.11 (m, 4.06H), 6.89 (dt, *J* = 17.2, 7.4 Hz, 2.53H), 6.81 (d, *J* = 8.0 Hz, 2.53H), 5.03 (td, *J* = 8.9, 5.3 Hz, 1H), 4.95 (td, *J* = 8.5, 6.2 Hz, 1.53H), 4.75

(t, J = 8.9 Hz, 1.53H), 4.66 (t, J = 8.8 Hz, 1H), 4.41 (dd, J = 9.2, 6.3 Hz, 1.53H), 4.29 (dd, J = 8.9, 6.0 Hz, 1H), 3.83–3.78 (m, 7.59H), 3.58 (tt, J = 15.0, 7.3 Hz, 2.53H), 2.48 (dt, J = 14.1, 5.9 Hz, 1H), 2.32–2.19 (m, 3.06H), 2.10 (dt, J = 13.9, 8.4 Hz, 1H). **^{13}C NMR** (150 MHz, $CDCl_3$) δ 171.88, 171.82, 163.94, 163.82, 159.36, 159.22, 148.68, 148.64, 147.90, 147.88, 136.99, 129.24, 129.09, 128.14, 128.09, 126.14, 126.13, 124.28, 123.65, 121.96, 120.23, 120.07, 109.26, 109.24, 75.97, 52.22, 52.15, 50.39, 50.22, 38.57, 38.41, 37.91, 37.79. **HRMS (ESI-TOF)** Calcd for $C_{18}H_{19}N_2O_4^+$ $[M+H]^+$ 327.1345, found 327.1349.

SAMPLE INFORMATION

Sample Name:	NH-446	Acquired By:	System
Sample Type:	Unknown	Sample Set Name	Samples_SFC
Vial:	1:F,3	Acq. Method Set:	G_ENG0308
Injection #:	1	Processing Method	ENG0308B
Injection Volume:	1.00 ul	Channel Name:	PDA Ch1 215nm@12.0nm
Run Time:	6.0 Minutes	Proc. Chnl. Descr.:	PDA Ch1 215nm@12.0nm
Date Acquired:	1/22/2020 9:36:36 AM PST		
Date Processed:	1/22/2020 9:43:50 AM PST		

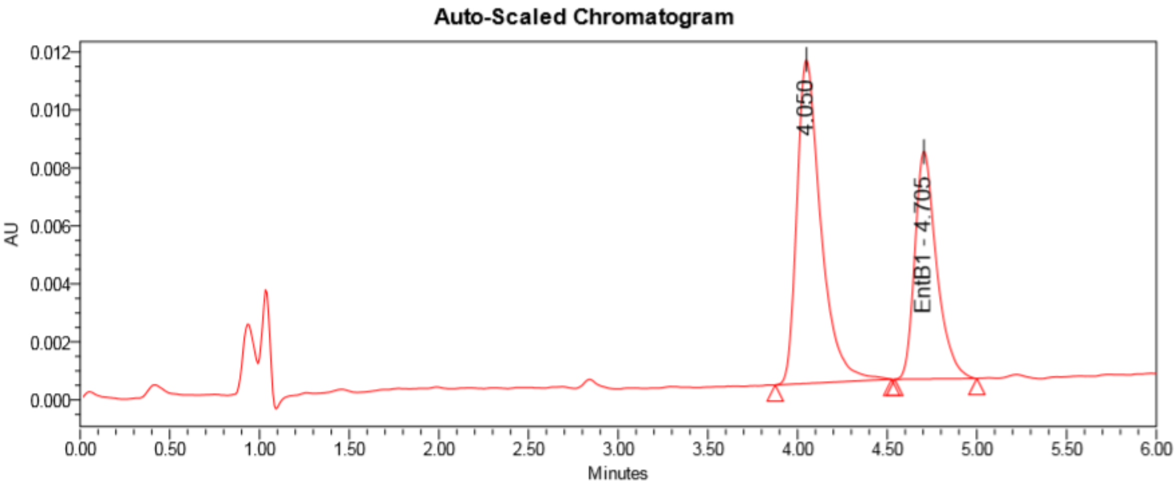

	SampleName	ent1	ent2	ee	EntA1	EntA2
1	NH-444	49.02	50.98	-1.96	42938	44658
2	NH-445	84.21	15.79	68.42	20456	3836
3	NH-446	100.00		100.00	103890	

Supplementary Figure 13: 1D-SFC result for **(±)-4ja** (NH-446).

General Procedure E for Deprotection of AQ Directing Group

To a 4-mL scintillation vial equipped with a Teflon-coated magnetic stir bar were added the Ni(tmhd)$_2$ (4.3 mg, 0.01 mmol), 2,3-dihydrobenzofuran or indoline substrate (0.1 mmol) and dry MeOH (1.0 mL) under an argon atmosphere. The vial was sealed with a screw-top septum cap and placed in a heating block that was pre-heated to 100 °C. The reaction was allowed to run at elevated temperature for 48 h. Then the reaction vial was allowed to cool to room temperature, and the reaction mixture was transferred directly to a preparative TLC, to afforded pure product.

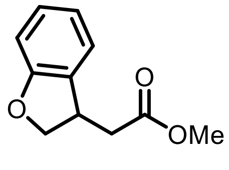

7aa

Methyl 2-(2,3-dihydrobenzofuran-3-yl)acetate (7aa): The title compound was prepared from **3aa** (30.4 mg, 0.1 mmol) according to the general procedure D. Purification using preparative TLC with 10:1 hexanes:EtOAc gave the product as a colorless oil (15.6 mg, 81% yield). **^{1}H NMR** (600 MHz, $CDCl_3$) δ 7.18–7.12 (m, 2H), 6.86 (td, *J* = 7.5, 1.0 Hz, 1H), 6.80 (dd, *J* = 7.8, 1.0 Hz, 1H), 4.75 (t, *J* = 9.0 Hz, 1H), 4.25 (dd, *J* = 9.2, 6.3 Hz, 1H), 3.88 (tdd, *J* = 10.3, 7.9, 4.7 Hz, 1H), 3.73 (s, 3H), 2.80 (dd, *J* = 16.5, 5.3 Hz, 1H), 2.59 (dd, *J* = 16.5, 9.5 Hz, 1H). **^{13}C NMR** (150 MHz, $CDCl_3$) δ 172.31, 159.83, 128.73, 128.09, 124.22, 120.60, 109.78, 76.73, 51.85, 39.26, 38.30. **HRMS (ESI-TOF)** Calcd for $C_{11}H_{13}O_3^+$ [M+H]$^+$ 193.0865, found 193.0862.

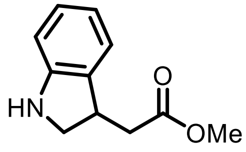

7ba

Methyl 2-(indolin-3-yl)acetate (7ba): The title compound was prepared from **3ba** (30.3 mg, 0.1 mmol) according to the general procedure D. Purification using preparative TLC with 10:1 hexanes:EtOAc gave the product as a colorless oil (17.0 mg, 89% yield). **^{1}H NMR** (600 MHz, $CDCl_3$) δ 7.08 (ddt, *J* = 7.6, 1.5, 0.8 Hz, 1H), 7.05 (tdd, *J* = 7.8, 1.4, 0.7 Hz, 1H), 6.72 (td, *J* = 7.4, 1.0 Hz, 1H), 6.65 (dt, *J* = 7.8, 0.7 Hz, 1H), 3.79 (t, *J* = 8.8 Hz, 1H), 3.72 (s, 4H), 3.27 (dd, *J* = 8.9, 6.5 Hz, 1H), 2.78 (dd, *J* = 16.1, 5.4 Hz, 1H), 2.58 (dd, *J* = 16.1, 9.1 Hz, 1H). **^{13}C NMR** (150 MHz, $CDCl_3$) δ 172.38, 150.76, 130.72, 127.54, 123.42, 118.30, 109.22, 52.90, 51.22, 38.27, 37.99. **HRMS (ESI-TOF)** Calcd for $C_{11}H_{14}O_3^+$ [M+H]$^+$ 192.2025, found 192.2026.

Procedure F for 50 mmol-Scale Reaction

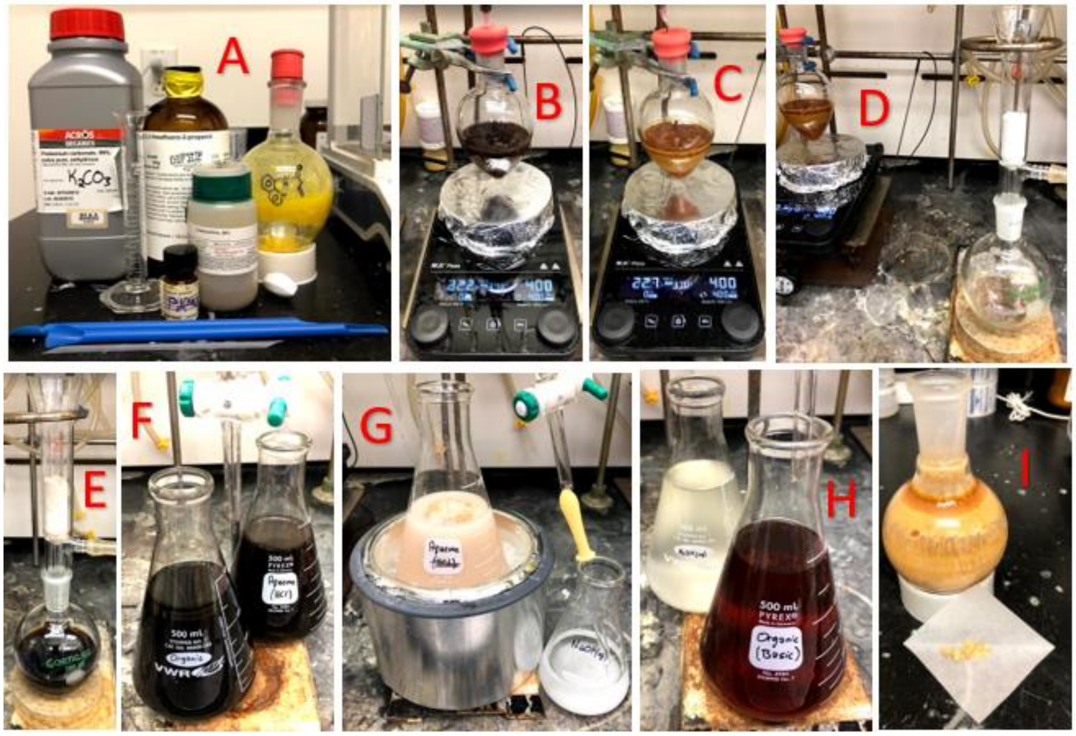

Supplementary Figure 14: Photographic depiction of the reaction procedure. A) Materials; B) t = 0 m; C) t = 18 h; D) Celite® filter setup E) Post Celite® filter; F) Acid workup; G) Basification of aqueous layer; H) Base workup; I) Product

To a solution of *N*-(quinolin-8-yl)but-3-enamide (50 mmol, 10.613 g, 1.0 equiv), 2-iodoaniline (60 mmol, 13.142 g, 1.2 equiv), and $Pd(OAc)_2$ (2.5 mmol, 561 mg, 5 mol%) in HFIP (0.5 M, 100 mL) was added K_2CO_3 (50 mmol, 6.910 g, 1.0 equiv) at ambient temperature (21 °C – 24 °C). The deep brown reaction was placed on a stir plate at 400 rpm. After 18 h, the light brown reaction mixture was filtered through diatomaceous earth with ethyl acetate rinses (3 × 50 mL). The mixture was concentrated under reduced pressure to remove the HFIP, and HCl (300 mL, 2 N) was added to the concentrate. The aqueous phase was passed through a frit filter to remove any solids and transferred to a separatory funnel. The remaining material was dissolved or suspended in DCM, passed through the frit filter, and transferred to the separatory funnel. The aqueous phase was extracted with DCM (3 × 150 mL). The dark red–brown aqueous phase (pH < 2) was placed in an ice–water bath, and at 0 °C the concentrated aqueous NaOH was added until pH > 8 and the solution turned light orange. The aqueous phase (pH > 8) was transferred to a separatory funnel. The remaining material was dissolved in ethyl acetate and transferred to the separatory funnel. The aqueous phase was extracted with ethyl acetate (3 × 150 mL). The combined organic phase was washed with brine (1 × 100 mL) and dried over Na_2SO_4. The reaction was filtered through a cotton plug and concentrated under reduced pressure. The mixture was checked for purity by TLC. If impure, the mixture was dissolved in EtOAc:Hex (1:9) and filtered through a plug of silica gel for three column volumes. The filtrant was checked by TLC with EtOAc:Hex (1:1) to ensure that all unwanted material (R_f = 0.92 and R_f = 0.77) was no longer eluting from the plug of silica gel and that no product (R_f = 0.45) had eluted. Three column volumes of EtOAc:Hex (9:1) were passed through the plug of silica gel eluting the product. The organic phase was concentrated under reduced pressure to afford the pure product as a yellow solid (14.292 g, 94.2%).

Unsuccessful examples

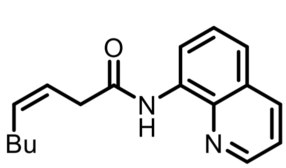

1k
<10%

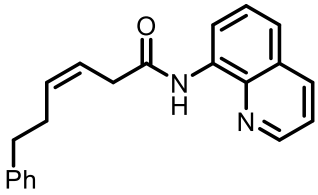

1l
16%

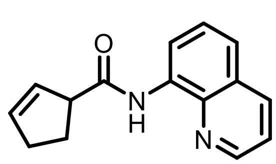

1m
N.D.

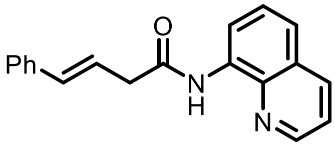

1n
N.D.

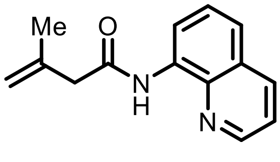

1o
N.D.

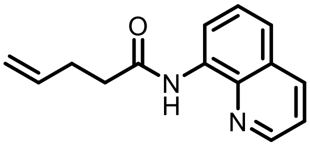

1p
N.D.

Supplementary Figure 15: Unsuccessful alkene examples.

1k-p were prepared according to literature procedures.[1-9] To a 4-mL scintillation vial equipped with a Teflon-coated magnetic stir bar were added the $Pd(OAc)_2$ (1.1 mg, 0.005 mmol, 5 mol%), alkene substrate (21.2 mg, 0.1 mmol), the appropriate 2-iodophenol (26.4 mg, 0.12 mmol, 1.2 equiv), K_2CO_3 (13.8 mg, 0.1 mmol, 1 equiv), and HFIP (0.1 mL). The vial was sealed with a screw-top septum cap and placed in a heating block that was pre-heated to 80 °C. The reaction was

allowed to run for 24 h and then was cooled to room temperature. Yields were determined by ^{1}H crude NMR using CH_2Br_2 as internal standard.

X-Ray Crystallography

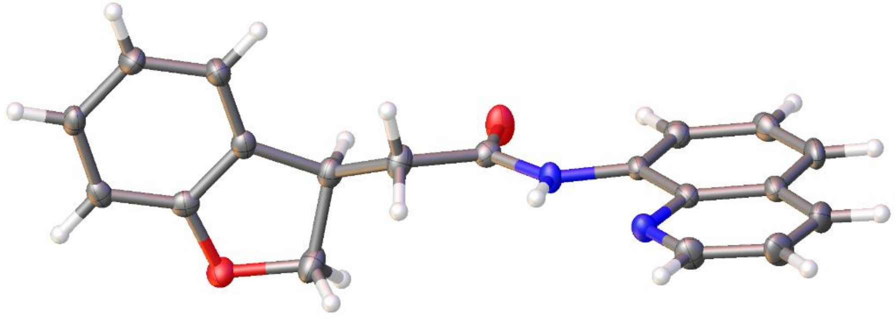

Supplementary Figure 16: Crystal data and structure refinement for **3aa**.

<u>**Experimental Summary**</u>

The single crystal X-ray diffraction studies were carried out on a Bruker APEX II Ultra CCD diffractometer equipped with Mo $K_{□}$ radiation (□ = 0.71073). Crystals of the subject compound were used as received (grown from DCM/Hexanes). A 0.090 x 0.080 x 0.065 mm colorless plank was mounted on a Cryoloop with Paratone oil.

Data were collected in a nitrogen gas stream at 100(2) K using □ and □ scans. Crystal-to-detector distance was 40 mm using exposure time 5.0s (depending on the detector 2□ position) with a scan width of 0.75°. Data collection was 100.0% complete to 25.242° in □. A total of 10384 reflections were collected covering the indices, -13<=h<=15, -20<=k<=19, -8<=l<=6. 2817 reflections were found to be symmetry independent, with a R_{int} of 0.0383. Indexing and unit cell refinement indicated a **Primitive**, **Monoclinic** lattice. The space group was found to be $\boldsymbol{P2_1/c}$. The data were integrated using the Bruker SAINT Software program and scaled using the SADABS software program. Solution by direct methods (SHELXT) produced a complete phasing model consistent with the proposed structure.

All nonhydrogen atoms were refined anisotropically by full-matrix least-squares (SHELXL-2014). All carbon bonded hydrogen atoms were placed using a riding model. Their positions were constrained relative to their parent atom using the appropriate HFIX command in SHELXL-2014.

Notes: Good data and refinement.
Clean residual maps!

Supplementary Table 9. Crystal data and structure refinement for **3aa**.

Report date	2020-09-09	
Identification code	engle267	
Empirical formula	C19 H16 N2 O2	
Molecular formula	C19 H16 N2 O2	
Formula weight	304.34	
Temperature	100.0 K	
Wavelength	0.71073 Å	
Crystal system	Monoclinic	
Space group	P 1 21/c 1	
Unit cell dimensions	a = 12.5000(6) Å	□= 90°.
	b = 16.7572(8) Å	□= 103.430(2)°.
	c = 7.3043(4) Å	□ = 90°.
Volume	1488.16(13) $Å^3$	
Z	4	
Density (calculated)	1.358 Mg/m^3	
Absorption coefficient	0.089 mm^{-1}	
F(000)	640	
Crystal size	0.09 x 0.08 x 0.065 mm^3	
Crystal color, habit	colorless plank	
Theta range for data collection	1.675 to 25.679°.	
Index ranges	-13<=h<=15, -20<=k<=19, -8<=l<=6	
Reflections collected	10384	
Independent reflections	2817 [R(int) = 0.0383]	
Completeness to theta = 25.242°	100.0 %	
Absorption correction	Semi-empirical from equivalents	
Max. and min. transmission	0.5622 and 0.5015	
Refinement method	Full-matrix least-squares on F^2	
Data / restraints / parameters	2817 / 1 / 211	

Goodness-of-fit on F^2	1.039
Final R indices [I>2sigma(I)]	R1 = 0.0445, wR2 = 0.0964
R indices (all data)	R1 = 0.0656, wR2 = 0.1054
Largest diff. peak and hole	0.261 and -0.214 e.$Å^{-3}$

Supplementary Table 10. Atomic coordinates (x 10^4) and equivalent isotropic displacement parameters ($Å^2$x 10^3)
for **3aa**. U(eq) is defined as one third of the trace of the orthogonalized U^{ij} tensor.

	x	y	z	U(eq)
O(1)	6977(1)	4186(1)	4007(2)	24(1)
O(2)	4198(1)	2067(1)	4238(2)	29(1)
N(1)	674(1)	3332(1)	1398(2)	21(1)
N(2)	2677(1)	2808(1)	3002(2)	19(1)
C(1)	5974(2)	3728(1)	3312(3)	30(1)
C(2)	5552(1)	3471(1)	5026(3)	22(1)
C(3)	6147(1)	4055(1)	6486(3)	20(1)
C(4)	6023(2)	4230(1)	8270(3)	25(1)
C(5)	6737(2)	4778(1)	9363(3)	26(1)
C(6)	7567(2)	5126(1)	8664(3)	22(1)
C(7)	7698(1)	4952(1)	6865(3)	20(1)
C(8)	6964(1)	4420(1)	5804(3)	19(1)
C(9)	4303(1)	3488(1)	4723(3)	23(1)
C(10)	3742(1)	2714(1)	3956(3)	19(1)
C(11)	1882(1)	2222(1)	2328(2)	16(1)
C(12)	2059(2)	1416(1)	2459(3)	21(1)
C(13)	1185(2)	883(1)	1747(3)	24(1)
C(14)	161(2)	1151(1)	911(3)	25(1)
C(15)	-51(1)	1981(1)	748(3)	21(1)
C(16)	-1090(2)	2311(1)	-84(3)	27(1)
C(17)	-1225(2)	3116(1)	-161(3)	30(1)
C(18)	-313(2)	3603(1)	605(3)	27(1)
C(19)	809(1)	2523(1)	1469(3)	18(1)

Supplementary Table 11. Bond lengths [Å] and angles [°] for **3aa**.

O(1)-C(1)	1.456(2)	C(15)-C(16)	1.412(3)
O(1)-C(8)	1.374(2)	C(15)-C(19)	1.413(2)
O(2)-C(10)	1.220(2)	C(16)-H(16)	0.9500
N(1)-C(18)	1.316(2)	C(16)-C(17)	1.360(3)
N(1)-C(19)	1.366(2)	C(17)-H(17)	0.9500
N(2)-H(2)	0.868(15)	C(17)-C(18)	1.408(3)
N(2)-C(10)	1.361(2)	C(18)-H(18)	0.9500
N(2)-C(11)	1.403(2)		
C(1)-H(1A)	0.9900	C(8)-O(1)-C(1)	106.45(13)
C(1)-H(1B)	0.9900	C(18)-N(1)-C(19)	117.00(16)
C(1)-C(2)	1.529(3)	C(10)-N(2)-H(2)	118.2(13)
C(2)-H(2A)	1.0000	C(10)-N(2)-C(11)	128.82(16)
C(2)-C(3)	1.510(3)	C(11)-N(2)-H(2)	112.8(13)
C(2)-C(9)	1.525(2)	O(1)-C(1)-H(1A)	110.3
C(3)-C(4)	1.379(3)	O(1)-C(1)-H(1B)	110.3
C(3)-C(8)	1.379(2)	O(1)-C(1)-C(2)	107.21(15)
C(4)-H(4)	0.9500	H(1A)-C(1)-H(1B)	108.5
C(4)-C(5)	1.395(3)	C(2)-C(1)-H(1A)	110.3
C(5)-H(5)	0.9500	C(2)-C(1)-H(1B)	110.3
C(5)-C(6)	1.388(3)	C(1)-C(2)-H(2A)	109.0
C(6)-H(6)	0.9500	C(3)-C(2)-C(1)	100.87(15)
C(6)-C(7)	1.392(3)	C(3)-C(2)-H(2A)	109.0
C(7)-H(7)	0.9500	C(3)-C(2)-C(9)	114.26(15)
C(7)-C(8)	1.381(3)	C(9)-C(2)-C(1)	114.37(16)
C(9)-H(9A)	0.9900	C(9)-C(2)-H(2A)	109.0
C(9)-H(9B)	0.9900	C(4)-C(3)-C(2)	131.27(16)
C(9)-C(10)	1.517(3)	C(8)-C(3)-C(2)	108.51(16)
C(11)-C(12)	1.368(3)	C(8)-C(3)-C(4)	120.16(17)
C(11)-C(19)	1.435(2)	C(3)-C(4)-H(4)	120.6
C(12)-H(12)	0.9500	C(3)-C(4)-C(5)	118.87(17)
C(12)-C(13)	1.413(3)	C(5)-C(4)-H(4)	120.6
C(13)-H(13)	0.9500	C(4)-C(5)-H(5)	120.0
C(13)-C(14)	1.361(3)	C(6)-C(5)-C(4)	120.03(18)
C(14)-H(14)	0.9500	C(6)-C(5)-H(5)	120.0
C(14)-C(15)	1.415(3)	C(5)-C(6)-H(6)	119.3

C(5)-C(6)-C(7)	121.37(17)
C(7)-C(6)-H(6)	119.3
C(6)-C(7)-H(7)	121.4
C(8)-C(7)-C(6)	117.18(16)
C(8)-C(7)-H(7)	121.4
O(1)-C(8)-C(3)	113.09(16)
O(1)-C(8)-C(7)	124.54(16)
C(3)-C(8)-C(7)	122.36(17)
C(2)-C(9)-H(9A)	108.7
C(2)-C(9)-H(9B)	108.7
H(9A)-C(9)-H(9B)	107.6
C(10)-C(9)-C(2)	114.08(15)
C(10)-C(9)-H(9A)	108.7
C(10)-C(9)-H(9B)	108.7
O(2)-C(10)-N(2)	123.55(17)
O(2)-C(10)-C(9)	122.62(16)
N(2)-C(10)-C(9)	113.77(15)
N(2)-C(11)-C(19)	114.85(15)
C(12)-C(11)-N(2)	125.27(16)
C(12)-C(11)-C(19)	119.87(16)
C(11)-C(12)-H(12)	120.0
C(11)-C(12)-C(13)	119.93(17)
C(13)-C(12)-H(12)	120.0
C(12)-C(13)-H(13)	119.2
C(14)-C(13)-C(12)	121.55(18)
C(14)-C(13)-H(13)	119.2
C(13)-C(14)-H(14)	120.0
C(13)-C(14)-C(15)	120.01(17)
C(15)-C(14)-H(14)	120.0
C(16)-C(15)-C(14)	123.78(17)
C(16)-C(15)-C(19)	116.90(18)
C(19)-C(15)-C(14)	119.32(17)
C(15)-C(16)-H(16)	120.0
C(17)-C(16)-C(15)	119.99(18)
C(17)-C(16)-H(16)	120.0
C(16)-C(17)-H(17)	120.8
C(16)-C(17)-C(18)	118.49(18)
C(18)-C(17)-H(17)	120.8
N(1)-C(18)-C(17)	124.39(19)
N(1)-C(18)-H(18)	117.8
C(17)-C(18)-H(18)	117.8
N(1)-C(19)-C(11)	117.46(15)
N(1)-C(19)-C(15)	123.23(16)
C(15)-C(19)-C(11)	119.31(17)

Supplementary Table 12. Anisotropic displacement parameters ($Å^2$x 10^3) for Engle267. The anisotropic
displacement factor exponent takes the form: -2□^{2}[h^2 $a^{*2}U^{11}$ + ... + 2 h k a* b* U^{12}]

	U^{11}	U^{22}	U^{33}	U^{23}	U^{13}	U^{12}
O(1)	24(1)	26(1)	23(1)	-5(1)	7(1)	-2(1)
O(2)	20(1)	21(1)	43(1)	-1(1)	-1(1)	4(1)
N(1)	18(1)	20(1)	24(1)	4(1)	1(1)	-1(1)
N(2)	16(1)	13(1)	25(1)	-1(1)	1(1)	0(1)
C(1)	23(1)	37(1)	26(1)	-11(1)	1(1)	-3(1)
C(2)	20(1)	20(1)	24(1)	-1(1)	0(1)	0(1)
C(3)	18(1)	17(1)	23(1)	3(1)	0(1)	0(1)
C(4)	24(1)	26(1)	24(1)	2(1)	4(1)	-8(1)
C(5)	32(1)	27(1)	19(1)	-2(1)	4(1)	-9(1)
C(6)	22(1)	17(1)	24(1)	1(1)	-1(1)	-3(1)
C(7)	16(1)	16(1)	26(1)	5(1)	3(1)	1(1)
C(8)	19(1)	16(1)	20(1)	3(1)	3(1)	5(1)
C(9)	17(1)	22(1)	27(1)	-5(1)	0(1)	3(1)
C(10)	17(1)	22(1)	19(1)	0(1)	4(1)	2(1)
C(11)	17(1)	17(1)	16(1)	-1(1)	4(1)	-4(1)
C(12)	20(1)	22(1)	22(1)	0(1)	6(1)	-1(1)
C(13)	30(1)	17(1)	25(1)	-2(1)	10(1)	-4(1)
C(14)	27(1)	24(1)	25(1)	-6(1)	6(1)	-13(1)
C(15)	21(1)	24(1)	19(1)	-1(1)	5(1)	-7(1)
C(16)	19(1)	33(1)	26(1)	3(1)	-1(1)	-9(1)
C(17)	18(1)	35(1)	32(1)	10(1)	-2(1)	0(1)
C(18)	23(1)	24(1)	31(1)	7(1)	1(1)	2(1)
C(19)	19(1)	18(1)	17(1)	0(1)	5(1)	-3(1)

Supplementary Table 13. Hydrogen coordinates (x 10^4) and isotropic displacement parameters (Å^{2}x 10^3) for **3aa**.

	x	y	z	U(eq)
H(2)	2413(15)	3289(9)	2840(30)	22
H(1A)	6130	3254	2608	35
H(1B)	5417	4059	2460	35
H(2A)	5822	2920	5402	26
H(4)	5460	3981	8747	30
H(5)	6655	4912	10587	31
H(6)	8057	5492	9429	26
H(7)	8267	5190	6388	24
H(9A)	4115	3609	5937	27
H(9B)	4008	3925	3837	27
H(12)	2769	1215	3029	25
H(13)	1316	325	1855	28
H(14)	-413	781	436	30
H(16)	-1693	1970	-590	33
H(17)	-1920	3345	-719	36
H(18)	-417	4165	545	32

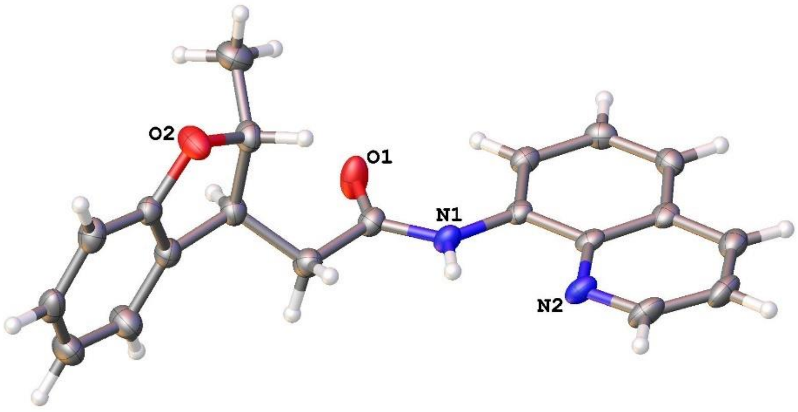

Supplementary Figure 17: Crystal data and structure refinement for major diastereomer of **4ga**.

The single crystal X-ray diffraction studies were carried out on a Bruker APEX II ULTRA CCD diffractometer equipped with Mo K_α radiation (λ = 0.71073 Å). Crystals of the subject compound (grown from toluene/hexane) were used as received. A 0.100 x 0.050 x 0.030 mm colorless block crystal was mounted on a Cryoloop with Paratone N oil.

Data were collected in a nitrogen gas stream at 100(2) K using ϖ and ϕ scans. Crystal-to-detector distance was 40 mm using an exposure time of 10 seconds with a scan width of 0.65°. Data collection was 100.0% complete to 25.242° in θ. A total of 13413 reflections were collected. 3019 reflections were found to be symmetry independent, with an R_{int} of 0.0431. Indexing and unit cell refinement indicated a **Primitive Monoclinic** lattice. The space group was found to be ***$P2_1/c$*.** The data were integrated using the Bruker SAINT Software program and scaled using the SADABS software program. Solution by direct methods (SHELXT) produced a complete phasing model consistent with the proposed structure.
All non-hydrogen atoms were refined anisotropically by full-matrix least-squares (SHELXL-2014). All carbon bonded hydrogen atoms were placed using a riding model. Their positions were constrained relative to their parent atom using the appropriate HFIX command in SHELXL-2014.

Notes: Excellent data and refinement. Although this molecule has chiral centers, it crystallized out in the centrosymmetric space group, **$P2_1/c$**, as the racemate.

Supplementary Table 14: Crystal data and structure refinement for **4ga**.

Report date	2019-11-19	
Identification code	Engle220	
Empirical formula	C20 H18 N2 O2	
Formula weight	318.36	
Temperature	100.0 K	
Wavelength	0.71073 Å	
Crystal system	Monoclinic	
Space group	P 1 21/c 1	
Unit cell dimensions	a = 8.4597(4) Å	□= 90°.
	b = 22.8443(8) Å	□= 103.990(2)°.
	c = 8.4530(4) Å	□ = 90°.
Volume	1585.14(12) Å^3	

Z	4
Density (calculated)	1.334 Mg/m^3
Absorption coefficient	0.087 mm^{-1}
F(000)	672
Crystal size	0.1 x 0.05 x 0.03 mm^3
Theta range for data collection	1.783 to 25.680°.
Index ranges	-10<=h<=10, -18<=k<=27, -10<=l<=10
Reflections collected	13413
Independent reflections	3019 [R(int) = 0.0431]
Completeness to theta = 25.242°	100.0 %
Absorption correction	Semi-empirical from equivalents
Max. and min. transmission	0.7454 and 0.6844
Refinement method	Full-matrix least-squares on F^2
Data / restraints / parameters	3019 / 0 / 218
Goodness-of-fit on F^2	1.048
Final R indices [I>2sigma(I)]	R1 = 0.0458, wR2 = 0.1179
R indices (all data)	R1 = 0.0548, wR2 = 0.1234
Extinction coefficient	n/a
Largest diff. peak and hole	0.431 and -0.218 e.Å^{-3}

Supplementary Table 15: Atomic coordinates (x 10^4) and equivalent isotropic displacement parameters (Å^{2}x 10^3) for **4ga**. U(eq) is defined as one third of the trace of the orthogonalized U^{ij} tensor.

	x	y	z	U(eq)
O(1)	7960(2)	4306(1)	9111(2)	37(1)
O(2)	8161(2)	2244(1)	7936(2)	35(1)
N(1)	7186(2)	4467(1)	6381(2)	24(1)
N(2)	6778(2)	4879(1)	3377(2)	26(1)
C(1)	7194(2)	4159(1)	7752(2)	24(1)
C(2)	7989(2)	4994(1)	6221(2)	20(1)
C(3)	9001(2)	5296(1)	7476(2)	22(1)
C(4)	9784(2)	5810(1)	7142(2)	25(1)
C(5)	9566(2)	6023(1)	5595(2)	25(1)
C(6)	8543(2)	5720(1)	4282(2)	23(1)
C(7)	8297(2)	5895(1)	2633(2)	29(1)
C(8)	7356(2)	5562(1)	1432(2)	32(1)
C(9)	6629(2)	5056(1)	1864(2)	32(1)
C(10)	7749(2)	5204(1)	4588(2)	20(1)
C(11)	6148(2)	3612(1)	7484(2)	27(1)
C(12)	6889(2)	3120(1)	8654(2)	25(1)
C(13)	8462(2)	2866(1)	8310(2)	28(1)

C(14)	9965(2)	2916(1)	9680(3)	40(1)
C(15)	6617(2)	2114(1)	8059(2)	24(1)
C(16)	5930(2)	1565(1)	7793(2)	30(1)
C(17)	4348(2)	1501(1)	7950(2)	31(1)
C(18)	3510(2)	1975(1)	8402(3)	40(1)
C(19)	4246(2)	2521(1)	8681(3)	37(1)
C(20)	5811(2)	2591(1)	8481(2)	26(1)

Supplementary Table 16: Bond lengths [Å] and angles [°] for **4ga**.

O(1)-C(1)	1.222(2)
O(2)-C(13)	1.464(2)
O(2)-C(15)	1.368(2)
N(1)-H(1)	0.8800
N(1)-C(1)	1.355(2)
N(1)-C(2)	1.403(2)
N(2)-C(9)	1.319(2)
N(2)-C(10)	1.366(2)
C(1)-C(11)	1.517(2)
C(2)-C(3)	1.377(2)
C(2)-C(10)	1.429(2)
C(3)-H(3)	0.9500
C(3)-C(4)	1.411(2)
C(4)-H(4)	0.9500
C(4)-C(5)	1.365(2)
C(5)-H(5)	0.9500
C(5)-C(6)	1.412(2)
C(6)-C(7)	1.416(2)
C(6)-C(10)	1.413(2)
C(7)-H(7)	0.9500
C(7)-C(8)	1.362(3)
C(8)-H(8)	0.9500
C(8)-C(9)	1.397(3)
C(9)-H(9)	0.9500
C(11)-H(11A)	0.9900
C(11)-H(11B)	0.9900
C(11)-C(12)	1.527(2)
C(12)-H(12)	1.0000
C(12)-C(13)	1.543(2)
C(12)-C(20)	1.500(2)
C(13)-H(13)	1.0000
C(13)-C(14)	1.503(3)
C(14)-H(14A)	0.9800
C(14)-H(14B)	0.9800
C(14)-H(14C)	0.9800
C(15)-C(16)	1.377(3)

C(15)-C(20)	1.377(2)
C(16)-H(16)	0.9500
C(16)-C(17)	1.385(3)
C(17)-H(17)	0.9500
C(17)-C(18)	1.397(3)
C(18)-H(18)	0.9500
C(18)-C(19)	1.390(3)
C(19)-H(19)	0.9500
C(19)-C(20)	1.384(3)
C(15)-O(2)-C(13)	108.38(13)
C(1)-N(1)-H(1)	115.6
C(1)-N(1)-C(2)	128.75(14)
C(2)-N(1)-H(1)	115.6
C(9)-N(2)-C(10)	117.30(16)
O(1)-C(1)-N(1)	123.52(16)
O(1)-C(1)-C(11)	121.69(15)
N(1)-C(1)-C(11)	114.78(14)
N(1)-C(2)-C(10)	114.94(14)
C(3)-C(2)-N(1)	125.46(15)
C(3)-C(2)-C(10)	119.55(15)
C(2)-C(3)-H(3)	120.1
C(2)-C(3)-C(4)	119.86(15)
C(4)-C(3)-H(3)	120.1
C(3)-C(4)-H(4)	119.1
C(5)-C(4)-C(3)	121.83(16)
C(5)-C(4)-H(4)	119.1
C(4)-C(5)-H(5)	120.2
C(4)-C(5)-C(6)	119.55(16)
C(6)-C(5)-H(5)	120.2
C(5)-C(6)-C(7)	123.49(16)
C(5)-C(6)-C(10)	119.58(15)
C(10)-C(6)-C(7)	116.90(15)
C(6)-C(7)-H(7)	120.1
C(8)-C(7)-C(6)	119.76(17)
C(8)-C(7)-H(7)	120.1
C(7)-C(8)-H(8)	120.6
C(7)-C(8)-C(9)	118.83(16)
C(9)-C(8)-H(8)	120.6
N(2)-C(9)-C(8)	124.26(17)
N(2)-C(9)-H(9)	117.9
C(8)-C(9)-H(9)	117.9
N(2)-C(10)-C(2)	117.45(15)
N(2)-C(10)-C(6)	122.91(15)
C(6)-C(10)-C(2)	119.61(14)
C(1)-C(11)-H(11A)	109.2
C(1)-C(11)-H(11B)	109.2

C(1)-C(11)-C(12)	112.11(14)
H(11A)-C(11)-H(11B)	107.9
C(12)-C(11)-H(11A)	109.2
C(12)-C(11)-H(11B)	109.2
C(11)-C(12)-H(12)	109.6
C(11)-C(12)-C(13)	113.07(14)
C(13)-C(12)-H(12)	109.6
C(20)-C(12)-C(11)	112.67(15)
C(20)-C(12)-H(12)	109.6
C(20)-C(12)-C(13)	102.15(14)
O(2)-C(13)-C(12)	106.99(14)
O(2)-C(13)-H(13)	108.6
O(2)-C(13)-C(14)	108.11(15)
C(12)-C(13)-H(13)	108.6
C(14)-C(13)-C(12)	115.69(16)
C(14)-C(13)-H(13)	108.6
C(13)-C(14)-H(14A)	109.5
C(13)-C(14)-H(14B)	109.5
C(13)-C(14)-H(14C)	109.5
H(14A)-C(14)-H(14B)	109.5
H(14A)-C(14)-H(14C)	109.5
H(14B)-C(14)-H(14C)	109.5
O(2)-C(15)-C(16)	124.03(16)
O(2)-C(15)-C(20)	112.85(15)
C(16)-C(15)-C(20)	123.12(17)
C(15)-C(16)-H(16)	121.3
C(15)-C(16)-C(17)	117.39(17)
C(17)-C(16)-H(16)	121.3
C(16)-C(17)-H(17)	119.6
C(16)-C(17)-C(18)	120.74(17)
C(18)-C(17)-H(17)	119.6
C(17)-C(18)-H(18)	119.8
C(19)-C(18)-C(17)	120.41(18)
C(19)-C(18)-H(18)	119.8
C(18)-C(19)-H(19)	120.5
C(20)-C(19)-C(18)	118.99(18)
C(20)-C(19)-H(19)	120.5
C(15)-C(20)-C(12)	109.61(15)
C(15)-C(20)-C(19)	119.31(17)
C(19)-C(20)-C(12)	131.07(17)

Supplementary Table 17: Anisotropic displacement parameters ($Å^2$x 10^3) for **4ga**. The anisotropic
displacement factor exponent takes the form: $-2\pi^2[h^2 a^{*2}U^{11} + ... + 2 h k a^* b^* U^{12}]$

	U^{11}	U^{22}	U^{33}	U^{23}	U^{13}	U^{12}
O(1)	59(1)	29(1)	21(1)	0(1)	6(1)	-12(1)
O(2)	31(1)	22(1)	57(1)	-1(1)	20(1)	1(1)
N(1)	26(1)	20(1)	21(1)	0(1)	-1(1)	-1(1)
N(2)	29(1)	22(1)	22(1)	0(1)	-3(1)	6(1)
C(1)	28(1)	19(1)	24(1)	0(1)	6(1)	4(1)
C(2)	20(1)	16(1)	24(1)	0(1)	4(1)	5(1)
C(3)	24(1)	23(1)	20(1)	-2(1)	4(1)	5(1)
C(4)	23(1)	23(1)	28(1)	-7(1)	3(1)	0(1)
C(5)	24(1)	20(1)	32(1)	-1(1)	8(1)	0(1)
C(6)	22(1)	21(1)	26(1)	1(1)	7(1)	8(1)
C(7)	29(1)	28(1)	31(1)	6(1)	9(1)	8(1)
C(8)	38(1)	35(1)	22(1)	7(1)	4(1)	14(1)
C(9)	36(1)	31(1)	22(1)	-1(1)	-5(1)	10(1)
C(10)	20(1)	18(1)	22(1)	-1(1)	1(1)	7(1)
C(11)	27(1)	23(1)	30(1)	1(1)	4(1)	0(1)
C(12)	27(1)	23(1)	27(1)	1(1)	10(1)	0(1)
C(13)	31(1)	21(1)	35(1)	3(1)	12(1)	0(1)
C(14)	28(1)	46(1)	47(1)	1(1)	11(1)	-1(1)
C(15)	26(1)	24(1)	22(1)	2(1)	8(1)	1(1)
C(16)	40(1)	22(1)	28(1)	0(1)	9(1)	0(1)
C(17)	36(1)	26(1)	28(1)	4(1)	1(1)	-9(1)
C(18)	29(1)	36(1)	54(1)	7(1)	12(1)	-5(1)
C(19)	32(1)	31(1)	50(1)	2(1)	16(1)	-1(1)
C(20)	27(1)	24(1)	28(1)	4(1)	7(1)	1(1)

Supplementary Table 18: Hydrogen coordinates (x 10^4) and isotropic displacement parameters ($Å^2$x 10^3)
for **4ga**.

	x	y	z	U(eq)
H(1)	6597	4318	5469	28
H(3)	9172	5158	8564	27
H(4)	10483	6014	8018	30
H(5)	10098	6373	5404	30
H(7)	8786	6244	2366	34
H(8)	7196	5671	320	39
H(9)	5986	4826	1012	38
H(11A)	6018	3475	6348	33
H(11B)	5053	3707	7636	33
H(12)	7118	3266	9801	30
H(13)	8671	3065	7327	34
H(14A)	9755	2743	10670	60
H(14B)	10252	3330	9877	60
H(14C)	10867	2709	9387	60
H(16)	6519	1243	7514	36
H(17)	3826	1131	7748	37
H(18)	2429	1923	8519	48
H(19)	3683	2842	9005	44

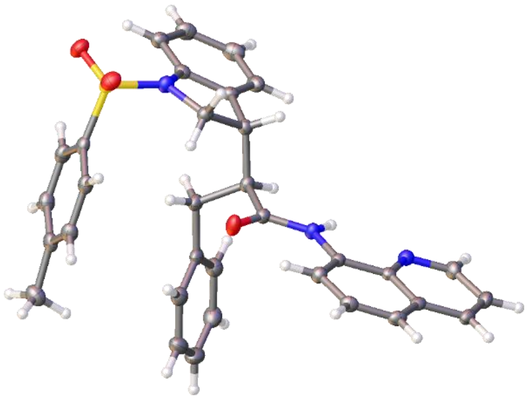

Supplementary Figure 18: Crystal data and structure refinement for major diastereomer of **4cp**.

The single crystal X-ray diffraction studies were carried out on a Bruker SMART APEX II CCD diffractometer equipped with Cu K_α radiation (λ = 1.54178). Crystals of the subject compound were used as received (grown from a toluene/hexane mixture). A 0.2 x 0.12 x 0.08 mm colorless irregular crystal was mounted on a Cryoloop with Paratone oil.

Data were collected in a nitrogen gas stream at 100(2) K using ϕ and ϖ scans. Crystal-to-detector distance was 40 mm using exposure times 2, 4, and 8 seconds (depending on the 2θ position) with a scan width of 1.25°. Data collection was 100.0 complete to 67.500° in θ. A total of 20853 reflections were collected. 10562 reflections were found to be symmetry independent, with a R_{int} of 0.0221. Indexing and unit cell refinement indicated a **Primitive Triclinic** lattice. The space group was found to be ***P*(-1).** The data were integrated using the Bruker SAINT Software program and scaled using the SADABS software program. Solution by direct methods (SHELXT) produced a complete phasing model consistent with the proposed structure.
All nonhydrogen atoms were refined anisotropically by full-matrix least-squares (SHELXL-2014). All carbon bonded hydrogen atoms were placed using a riding model. Their positions were constrained relative to their parent atom using the appropriate HFIX command in SHELXL-2014.

Notes: Excellent quality data. Refined as non-merohedral twin mimicking pseudo-merohedral twinning. During final refinement, HKLF 5 file was used with both components included.
(TWIN law 1.00015 0.00060 -0.00043; -0.50721 -1.00075 -0.00147; -0.00637 0.00479 -0.99939)

Supplementary Table 19: Crystal data and structure refinement for **4cp**.

Identification code	engle228	
Empirical formula	C33 H29 N3 O3 S	
Formula weight	547.65	
Temperature	100.0 K	
Wavelength	1.54178 Å	
Crystal system	Triclinic	
Space group	P-1	
Unit cell dimensions	a = 8.91770(10) Å	α= 102.3630(10)°.
	b = 9.60220(10) Å	β= 90.0930(10)°.
	c = 16.2563(2) Å	γ = 103.6390(10)°.
Volume	1319.40(3) Å^3	
Z	2	
Density (calculated)	1.378 Mg/m^3	
Absorption coefficient	1.423 mm^{-1}	
F(000)	576	
Crystal size	0.2 x 0.12 x 0.08 mm^3	
Theta range for data collection	2.787 to 70.095°.	
Index ranges	-10<=h<=10, -11<=k<=11, -19<=l<=19	
Reflections collected	20853	
Independent reflections	10562 [R(int) = 0.0221]	
Completeness to theta = 67.500°	100.0 %	
Absorption correction	Semi-empirical from equivalents	
Max. and min. transmission	0.87 and 0.76	
Refinement method	Full-matrix least-squares on F^2	
Data / restraints / parameters	10562 / 1 / 366	
Goodness-of-fit on F^2	1.031	
Final R indices [I>2sigma(I)]	R1 = 0.0347, wR2 = 0.0937	
R indices (all data)	R1 = 0.0393, wR2 = 0.0968	

Extinction coefficient	n/a
Largest diff. peak and hole	0.286 and -0.455 e.Å^{-3}

Supplementary Table 20: Atomic coordinates (x 10^4) and equivalent isotropic displacement parameters (Å^{2}x 10^3) for **4cp**. U(eq) is defined as one third of the trace of the orthogonalized U^{ij} tensor.

	x	y	z	U(eq)
S(1)	10394(1)	3671(1)	3046(1)	21(1)
O(1)	10362(1)	2165(1)	2695(1)	27(1)
O(2)	11230(1)	4422(1)	3829(1)	27(1)
O(3)	8645(1)	8366(1)	3421(1)	27(1)
N(1)	8609(2)	3790(1)	3178(1)	21(1)
N(2)	6157(2)	8581(1)	3468(1)	18(1)
N(3)	3617(2)	9571(1)	3642(1)	19(1)
C(1)	12362(2)	7219(2)	404(1)	31(1)
C(2)	11964(2)	6314(2)	1062(1)	24(1)
C(3)	12295(2)	6961(2)	1917(1)	24(1)
C(4)	11867(2)	6162(2)	2531(1)	22(1)
C(5)	11091(2)	4689(2)	2285(1)	21(1)
C(6)	10785(2)	4012(2)	1433(1)	24(1)
C(7)	11230(2)	4823(2)	832(1)	26(1)
C(8)	7265(2)	2868(2)	2704(1)	19(1)
C(9)	7014(2)	1420(2)	2263(1)	24(1)
C(10)	5543(2)	733(2)	1897(1)	27(1)
C(11)	4354(2)	1447(2)	1978(1)	29(1)
C(12)	4629(2)	2909(2)	2420(1)	25(1)
C(13)	6085(2)	3614(2)	2778(1)	20(1)
C(14)	6670(2)	5181(2)	3266(1)	20(1)
C(15)	8287(2)	5195(2)	3628(1)	20(1)
C(16)	6682(2)	6295(2)	2696(1)	19(1)
C(17)	7683(2)	6034(2)	1937(1)	22(1)
C(18)	7632(2)	7019(2)	1331(1)	21(1)
C(19)	6420(2)	6669(2)	720(1)	27(1)
C(20)	6414(2)	7535(2)	135(1)	33(1)
C(21)	7595(2)	8770(2)	168(1)	35(1)
C(22)	8780(2)	9142(2)	782(1)	33(1)
C(23)	8803(2)	8272(2)	1359(1)	26(1)
C(24)	7273(2)	7856(2)	3225(1)	19(1)
C(25)	6325(2)	10009(2)	3961(1)	17(1)
C(26)	7686(2)	10904(2)	4355(1)	21(1)
C(27)	7698(2)	12322(2)	4831(1)	22(1)
C(28)	6394(2)	12838(2)	4913(1)	21(1)
C(29)	4979(2)	11940(2)	4512(1)	19(1)
C(30)	3577(2)	12385(2)	4557(1)	22(1)

C(31)	2254(2)	11431(2)	4172(1)	24(1)
C(32)	2331(2)	10028(2)	3718(1)	23(1)
C(33)	4935(2)	10508(2)	4038(1)	17(1)

Supplementary Table 21: Bond lengths [Å] and angles [°] for **4cp**.

S(1)-O(1)	1.4298(12)	C(15)-H(15A)	0.9900
S(1)-O(2)	1.4311(12)	C(15)-H(15B)	0.9900
S(1)-N(1)	1.6335(14)	C(16)-H(16)	1.0000
S(1)-C(5)	1.7619(17)	C(16)-C(17)	1.536(2)
O(3)-C(24)	1.220(2)	C(16)-C(24)	1.529(2)
N(1)-C(8)	1.421(2)	C(17)-H(17A)	0.9900
N(1)-C(15)	1.483(2)	C(17)-H(17B)	0.9900
N(2)-H(2)	0.860(17)	C(17)-C(18)	1.513(2)
N(2)-C(24)	1.357(2)	C(18)-C(19)	1.396(2)
N(2)-C(25)	1.405(2)	C(18)-C(23)	1.387(2)
N(3)-C(32)	1.319(2)	C(19)-H(19)	0.9500
N(3)-C(33)	1.361(2)	C(19)-C(20)	1.392(2)
C(1)-H(1A)	0.9800	C(20)-H(20)	0.9500
C(1)-H(1B)	0.9800	C(20)-C(21)	1.379(3)
C(1)-H(1C)	0.9800	C(21)-H(21)	0.9500
C(1)-C(2)	1.510(2)	C(21)-C(22)	1.381(3)
C(2)-C(3)	1.397(2)	C(22)-H(22)	0.9500
C(2)-C(7)	1.395(2)	C(22)-C(23)	1.386(2)
C(3)-H(3)	0.9500	C(23)-H(23)	0.9500
C(3)-C(4)	1.386(2)	C(25)-C(26)	1.376(2)
C(4)-H(4)	0.9500	C(25)-C(33)	1.427(2)
C(4)-C(5)	1.392(2)	C(26)-H(26)	0.9500
C(5)-C(6)	1.394(2)	C(26)-C(27)	1.413(2)
C(6)-H(6)	0.9500	C(27)-H(27)	0.9500
C(6)-C(7)	1.378(3)	C(27)-C(28)	1.363(2)
C(7)-H(7)	0.9500	C(28)-H(28)	0.9500
C(8)-C(9)	1.387(2)	C(28)-C(29)	1.416(2)
C(8)-C(13)	1.398(2)	C(29)-C(30)	1.411(2)
C(9)-H(9)	0.9500	C(29)-C(33)	1.417(2)
C(9)-C(10)	1.390(2)	C(30)-H(30)	0.9500
C(10)-H(10)	0.9500	C(30)-C(31)	1.366(2)
C(10)-C(11)	1.384(3)	C(31)-H(31)	0.9500
C(11)-H(11)	0.9500	C(31)-C(32)	1.408(2)
C(11)-C(12)	1.398(3)	C(32)-H(32)	0.9500
C(12)-H(12)	0.9500		
C(12)-C(13)	1.378(2)	O(1)-S(1)-O(2)	120.81(7)
C(13)-C(14)	1.510(2)	O(1)-S(1)-N(1)	107.60(7)
C(14)-H(14)	1.0000	O(1)-S(1)-C(5)	107.77(8)
C(14)-C(15)	1.553(2)	O(2)-S(1)-N(1)	105.93(8)
C(14)-C(16)	1.555(2)	O(2)-S(1)-C(5)	107.53(8)

N(1)-S(1)-C(5)	106.37(7)
C(8)-N(1)-S(1)	127.08(12)
C(8)-N(1)-C(15)	110.28(13)
C(15)-N(1)-S(1)	119.82(11)
C(24)-N(2)-H(2)	120.0(13)
C(24)-N(2)-C(25)	128.45(14)
C(25)-N(2)-H(2)	111.4(13)
C(32)-N(3)-C(33)	117.76(14)
H(1A)-C(1)-H(1B)	109.5
H(1A)-C(1)-H(1C)	109.5
H(1B)-C(1)-H(1C)	109.5
C(2)-C(1)-H(1A)	109.5
C(2)-C(1)-H(1B)	109.5
C(2)-C(1)-H(1C)	109.5
C(3)-C(2)-C(1)	120.52(15)
C(7)-C(2)-C(1)	120.93(16)
C(7)-C(2)-C(3)	118.54(16)
C(2)-C(3)-H(3)	119.4
C(4)-C(3)-C(2)	121.22(16)
C(4)-C(3)-H(3)	119.4
C(3)-C(4)-H(4)	120.5
C(3)-C(4)-C(5)	119.01(16)
C(5)-C(4)-H(4)	120.5
C(4)-C(5)-S(1)	120.46(13)
C(4)-C(5)-C(6)	120.61(16)
C(6)-C(5)-S(1)	118.85(12)
C(5)-C(6)-H(6)	120.3
C(7)-C(6)-C(5)	119.49(15)
C(7)-C(6)-H(6)	120.3
C(2)-C(7)-H(7)	119.5
C(6)-C(7)-C(2)	121.07(16)
C(6)-C(7)-H(7)	119.5
C(9)-C(8)-N(1)	129.16(15)
C(9)-C(8)-C(13)	121.41(15)
C(13)-C(8)-N(1)	109.32(14)
C(8)-C(9)-H(9)	121.2
C(8)-C(9)-C(10)	117.54(16)
C(10)-C(9)-H(9)	121.2
C(9)-C(10)-H(10)	119.1
C(11)-C(10)-C(9)	121.81(16)
C(11)-C(10)-H(10)	119.1
C(10)-C(11)-H(11)	120.1
C(10)-C(11)-C(12)	119.87(16)
C(12)-C(11)-H(11)	120.1
C(11)-C(12)-H(12)	120.4
C(13)-C(12)-C(11)	119.17(16)
C(13)-C(12)-H(12)	120.4
C(8)-C(13)-C(14)	110.81(14)
C(12)-C(13)-C(8)	120.18(15)
C(12)-C(13)-C(14)	129.00(15)
C(13)-C(14)-H(14)	109.1
C(13)-C(14)-C(15)	103.13(12)
C(13)-C(14)-C(16)	111.58(13)
C(15)-C(14)-H(14)	109.1
C(15)-C(14)-C(16)	114.69(13)
C(16)-C(14)-H(14)	109.1
N(1)-C(15)-C(14)	104.97(12)
N(1)-C(15)-H(15A)	110.8
N(1)-C(15)-H(15B)	110.8
C(14)-C(15)-H(15A)	110.8
C(14)-C(15)-H(15B)	110.8
H(15A)-C(15)-H(15B)	108.8
C(14)-C(16)-H(16)	108.5
C(17)-C(16)-C(14)	111.98(13)
C(17)-C(16)-H(16)	108.5
C(24)-C(16)-C(14)	109.09(13)
C(24)-C(16)-H(16)	108.5
C(24)-C(16)-C(17)	110.14(14)
C(16)-C(17)-H(17A)	108.8
C(16)-C(17)-H(17B)	108.8
H(17A)-C(17)-H(17B)	107.7
C(18)-C(17)-C(16)	113.74(13)
C(18)-C(17)-H(17A)	108.8
C(18)-C(17)-H(17B)	108.8
C(19)-C(18)-C(17)	120.62(15)
C(23)-C(18)-C(17)	120.74(15)
C(23)-C(18)-C(19)	118.62(15)
C(18)-C(19)-H(19)	119.7
C(20)-C(19)-C(18)	120.62(17)
C(20)-C(19)-H(19)	119.7
C(19)-C(20)-H(20)	120.1
C(21)-C(20)-C(19)	119.89(18)
C(21)-C(20)-H(20)	120.1
C(20)-C(21)-H(21)	120.1
C(20)-C(21)-C(22)	119.86(16)
C(22)-C(21)-H(21)	120.1
C(21)-C(22)-H(22)	119.8
C(21)-C(22)-C(23)	120.44(18)
C(23)-C(22)-H(22)	119.8
C(18)-C(23)-H(23)	119.7
C(22)-C(23)-C(18)	120.54(17)
C(22)-C(23)-H(23)	119.7
O(3)-C(24)-N(2)	123.98(14)
O(3)-C(24)-C(16)	121.15(15)

N(2)-C(24)-C(16)	114.84(14)
N(2)-C(25)-C(33)	114.70(14)
C(26)-C(25)-N(2)	125.12(15)
C(26)-C(25)-C(33)	120.18(14)
C(25)-C(26)-H(26)	120.3
C(25)-C(26)-C(27)	119.48(15)
C(27)-C(26)-H(26)	120.3
C(26)-C(27)-H(27)	119.1
C(28)-C(27)-C(26)	121.87(15)
C(28)-C(27)-H(27)	119.1
C(27)-C(28)-H(28)	120.1
C(27)-C(28)-C(29)	119.76(15)
C(29)-C(28)-H(28)	120.1
C(28)-C(29)-C(33)	119.39(15)
C(30)-C(29)-C(28)	123.63(15)
C(30)-C(29)-C(33)	116.98(15)
C(29)-C(30)-H(30)	120.1
C(31)-C(30)-C(29)	119.80(15)
C(31)-C(30)-H(30)	120.1
C(30)-C(31)-H(31)	120.6
C(30)-C(31)-C(32)	118.84(16)
C(32)-C(31)-H(31)	120.6
N(3)-C(32)-C(31)	123.65(16)
N(3)-C(32)-H(32)	118.2
C(31)-C(32)-H(32)	118.2
N(3)-C(33)-C(25)	117.75(14)
N(3)-C(33)-C(29)	122.94(14)
C(29)-C(33)-C(25)	119.31(14)

Supplementary Table 22: Anisotropic displacement parameters ($Å^2$x 10^3) for **4cp**. The anisotropic
displacement factor exponent takes the form: $-2\pi^2[h^2 a^{*2}U^{11} + ... + 2 h k a^* b^* U^{12}]$

	U^{11}	U^{22}	U^{33}	U^{23}	U^{13}	U^{12}
S(1)	20(1)	18(1)	24(1)	3(1)	-3(1)	6(1)
O(1)	26(1)	20(1)	35(1)	4(1)	-2(1)	9(1)
O(2)	27(1)	25(1)	28(1)	4(1)	-6(1)	7(1)
O(3)	18(1)	23(1)	37(1)	0(1)	2(1)	6(1)
N(1)	21(1)	18(1)	22(1)	1(1)	1(1)	5(1)
N(2)	16(1)	16(1)	22(1)	2(1)	0(1)	4(1)
N(3)	20(1)	19(1)	20(1)	5(1)	1(1)	5(1)
C(1)	26(1)	36(1)	28(1)	6(1)	3(1)	2(1)
C(2)	16(1)	28(1)	27(1)	4(1)	4(1)	6(1)
C(3)	19(1)	21(1)	29(1)	1(1)	1(1)	2(1)
C(4)	17(1)	23(1)	23(1)	0(1)	0(1)	5(1)
C(5)	16(1)	22(1)	25(1)	3(1)	0(1)	8(1)
C(6)	22(1)	19(1)	29(1)	-1(1)	0(1)	6(1)
C(7)	24(1)	29(1)	23(1)	-1(1)	2(1)	8(1)
C(8)	20(1)	20(1)	17(1)	6(1)	2(1)	2(1)
C(9)	28(1)	20(1)	23(1)	4(1)	4(1)	5(1)
C(10)	32(1)	20(1)	25(1)	2(1)	0(1)	-1(1)
C(11)	27(1)	28(1)	27(1)	7(1)	-6(1)	-3(1)
C(12)	22(1)	28(1)	29(1)	13(1)	2(1)	6(1)
C(13)	23(1)	18(1)	19(1)	7(1)	2(1)	4(1)
C(14)	20(1)	19(1)	19(1)	4(1)	3(1)	5(1)
C(15)	23(1)	18(1)	20(1)	2(1)	1(1)	6(1)
C(16)	19(1)	18(1)	21(1)	2(1)	1(1)	7(1)
C(17)	27(1)	22(1)	21(1)	5(1)	6(1)	12(1)
C(18)	26(1)	22(1)	20(1)	4(1)	8(1)	11(1)
C(19)	27(1)	29(1)	26(1)	6(1)	5(1)	8(1)
C(20)	34(1)	43(1)	26(1)	10(1)	3(1)	18(1)
C(21)	48(1)	37(1)	31(1)	19(1)	13(1)	22(1)
C(22)	39(1)	26(1)	36(1)	11(1)	15(1)	8(1)
C(23)	28(1)	25(1)	27(1)	4(1)	5(1)	9(1)
C(24)	20(1)	18(1)	19(1)	4(1)	3(1)	6(1)
C(25)	20(1)	15(1)	16(1)	4(1)	2(1)	5(1)
C(26)	19(1)	21(1)	21(1)	5(1)	0(1)	5(1)
C(27)	23(1)	19(1)	21(1)	3(1)	-1(1)	0(1)
C(28)	30(1)	15(1)	18(1)	2(1)	0(1)	4(1)
C(29)	26(1)	18(1)	15(1)	6(1)	3(1)	7(1)
C(30)	30(1)	20(1)	20(1)	5(1)	5(1)	12(1)
C(31)	24(1)	29(1)	26(1)	10(1)	4(1)	14(1)
C(32)	16(1)	28(1)	26(1)	7(1)	0(1)	6(1)
C(33)	19(1)	17(1)	15(1)	6(1)	2(1)	4(1)

Supplementary Table 23: Hydrogen coordinates (x 10^4) and isotropic displacement parameters ($Å^2$x 10 ^{3}) for **4cp**.

	x	y	z	U(eq)
H(2)	5210(20)	8190(20)	3286(12)	22
H(1A)	11511	7669	320	46
H(1B)	12528	6586	-129	46
H(1C)	13306	7992	594	46
H(3)	12823	7968	2082	29
H(4)	12100	6614	3111	26
H(6)	10274	3001	1268	29
H(7)	11034	4359	251	31
H(9)	7818	915	2213	28
H(10)	5349	-253	1582	33
H(11)	3352	943	1732	35
H(12)	3821	3409	2474	30
H(14)	5988	5379	3742	23
H(15A)	9078	6043	3521	25
H(15B)	8276	5253	4244	25
H(16)	5598	6179	2482	23
H(17A)	8767	6193	2146	27
H(17B)	7332	4997	1628	27
H(19)	5592	5832	704	33
H(20)	5597	7276	-287	39
H(21)	7594	9363	-231	42
H(22)	9586	10001	809	40
H(23)	9627	8536	1776	32
H(26)	8611	10571	4307	25
H(27)	8642	12934	5103	26
H(28)	6435	13796	5237	26
H(30)	3553	13345	4855	27
H(31)	1298	11710	4209	29
H(32)	1404	9374	3452	27

Disorder:

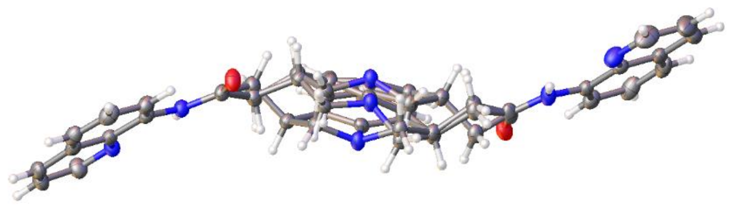

cis:

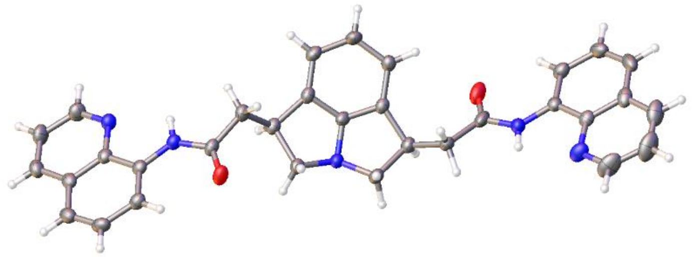

trans:

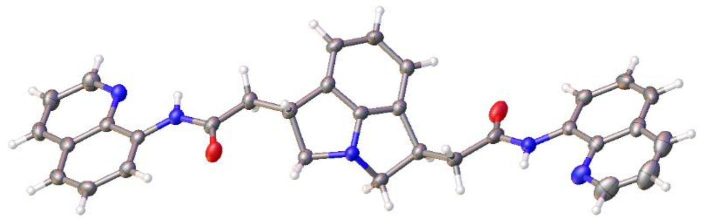

Supplementary Figure 19: Crystal data and structure refinement for **3bn'**.

The single crystal X-ray diffraction studies were carried out on a Bruker Smart APEX II CCD diffractometer equipped with Cu K_{α} radiation (λ =1.54178 Å).

Crystals of the subject compound were used as received. A 0.22 x 0.16 x 0.14 mm piece of a colorless crystal was mounted on a Cryoloop with Paratone oil. Data were collected in a nitrogen gas stream at 100(2) K using ϕ and ϖ scans. Crystal-to-detector distance was 40 mm and exposure time was 1, 2, or 5 seconds depending on the 2θ range per frame using a scan width of 1.25°. Data collection was 98.2 % complete to 67.679° in θ. A total of 16276 reflections were collected covering the indices, -40<=h<=39, -7<=k<=7, -39<=l<=39. 5349 reflections were found to be

symmetry independent, with a R_{int} of 0.0349. Indexing and unit cell refinement indicated a **C-centered**, **Monoclinic** lattice. The space group was found to be ***C*2/*c***. The data were integrated using the Bruker SAINT Software program and scaled using the SADABS software program. Solution by direct methods (SHELXT) produced a complete phasing model consistent with the proposed structure.

All nonhydrogen atoms were refined anisotropically by full-matrix least-squares (SHELXL-2014). All carbon bonded hydrogen atoms were placed using a riding model. Their positions were constrained relative to their parent atom using the appropriate HFIX command in SHELXL-2014. Crystallographic data are summarized in Table 1.

Notes: Great data! There is one copy of the molecule in the asymmetric unit and one molecule of DCM. Both *cis* and *trans* isomers are in the structure with a ratio of ~55/45. The DCM is also positionally disordered between two positions. The chemical formula of the molecule is $C_{33}H_{27}N_5O_2$.

Supplementary Table 24. Crystal data and structure refinement for **3bn'**.

Identification code	prelim_a	
Empirical formula	C33 H29 Cl2 N5 O2	
Formula weight	598.51	
Temperature	100.0 K	
Wavelength	1.54178 Å	
Crystal system	Monoclinic	
Space group	C 1 2/c 1	
Unit cell dimensions	a = 32.8993(5) Å	α= 90°.
	b = 6.09050(10) Å	β= 117.2380(10)°.
	c = 32.4083(5) Å	γ = 90°.
Volume	5773.68(16) $Å^3$	
Z	8	

Density (calculated)	1.377 Mg/m^3
Absorption coefficient	2.347 mm^{-1}
F(000)	2496
Crystal size	0.22 x 0.16 x 0.14 mm^3
Theta range for data collection	3.021 to 70.100°.
Index ranges	-40<=h<=39, -7<=k<=7, -39<=l<=39
Reflections collected	16276
Independent reflections	5349 [R(int) = 0.0349]
Completeness to theta = 67.679°	98.2 %
Absorption correction	None
Max. and min. transmission	0.5220 and 0.4374
Refinement method	Full-matrix least-squares on F^2
Data / restraints / parameters	5349 / 127 / 482
Goodness-of-fit on F^2	1.126
Final R indices [I>2sigma(I)]	R1 = 0.0668, wR2 = 0.1434
R indices (all data)	R1 = 0.0774, wR2 = 0.1483
Extinction coefficient	n/a
Largest diff. peak and hole	0.438 and -0.311 e.Å^{-3}

Supplementary Table 25. Atomic coordinates (x 10^4) and equivalent isotropic displacement parameters (Å^{2}x 10^3)
for **3bn'**. U(eq) is defined as one third of the trace of the orthogonalized U^{ij} tensor.

	x	y	z	U(eq)
O(1)	4195(1)	3295(4)	6108(1)	39(1)
O(2)	3057(1)	12028(4)	3435(1)	46(1)
N(1)	2987(1)	-2(4)	6379(1)	31(1)
N(2)	3579(1)	2220(4)	6199(1)	27(1)
N(4)	3721(1)	13071(4)	3447(1)	33(1)
N(5)	4368(1)	14983(5)	3305(1)	40(1)
C(1)	2682(1)	-1114(5)	6452(1)	38(1)
C(2)	2774(1)	-3122(5)	6694(1)	40(1)
C(3)	3202(1)	-3978(5)	6870(1)	35(1)
C(4)	3548(1)	-2840(4)	6811(1)	30(1)
C(5)	4004(1)	-3601(5)	6985(1)	35(1)
C(6)	4308(1)	-2433(5)	6899(1)	37(1)
C(7)	4184(1)	-460(5)	6638(1)	32(1)
C(8)	3746(1)	330(4)	6469(1)	26(1)
C(9)	3418(1)	-853(4)	6557(1)	26(1)
C(10)	3796(1)	3548(5)	6026(1)	29(1)
C(23)	3469(1)	11792(5)	3583(1)	39(1)
C(24)	3589(1)	14926(4)	3162(1)	26(1)
C(25)	3158(1)	15822(5)	2954(1)	34(1)
C(26)	3076(1)	17769(5)	2695(1)	42(1)
C(27)	3412(1)	18804(5)	2641(1)	43(1)
C(28)	3858(1)	17916(5)	2839(1)	36(1)
C(29)	3949(1)	15945(5)	3102(1)	28(1)
C(30)	4223(1)	18850(6)	2786(1)	52(1)
C(31)	4632(1)	17876(8)	2978(1)	62(1)
C(32)	4700(1)	15956(8)	3245(1)	57(1)
N(3)	3869(2)	7090(8)	4828(2)	28(1)
C(11)	3485(7)	5220(30)	5687(7)	30(2)

C(12)	3731(3)	6699(14)	5490(2)	28(1)
C(13)	3412(3)	8430(19)	5154(3)	28(1)
C(14)	3034(4)	9620(20)	5097(4)	31(2)
C(15)	2777(6)	10710(30)	4672(5)	36(2)
C(16)	2887(5)	10640(30)	4302(5)	29(3)
C(17)	3266(6)	9400(30)	4360(5)	27(2)
C(18)	3465(5)	8460(20)	4050(4)	29(1)
C(19)	3737(3)	6449(17)	4342(3)	30(2)
C(20)	3909(3)	5454(10)	5182(2)	26(2)
C(21)	3509(2)	8437(10)	4781(2)	26(1)
C(22)	3766(9)	10010(30)	3942(9)	30(2)
N(3A)	3553(2)	5711(9)	4629(2)	28(1)
C(11A)	3543(7)	5610(30)	5772(4)	30(2)
C(12A)	3359(2)	5302(11)	5249(2)	28(1)
C(13A)	3141(2)	7386(11)	4971(2)	28(1)
C(14A)	2875(3)	9112(14)	4975(4)	31(2)
C(15A)	2789(7)	10850(30)	4658(6)	36(2)
C(16A)	2972(6)	10920(30)	4342(6)	29(3)
C(17A)	3229(8)	9120(30)	4330(6)	27(2)
C(18A)	3513(6)	8440(30)	4084(5)	29(1)
C(19A)	3838(5)	6710(20)	4432(4)	30(2)
C(20A)	3736(2)	4778(12)	5096(2)	26(2)
C(21A)	3281(2)	7456(10)	4628(2)	26(1)
C(22A)	3774(10)	10260(30)	3986(11)	30(2)
N(3B)	3972(4)	8036(19)	4989(4)	28(1)
C(11B)	3462(17)	5230(60)	5670(20)	30(2)
C(12B)	3698(8)	7010(40)	5525(6)	28(1)
C(13B)	3358(8)	8580(50)	5158(8)	28(1)
C(14B)	2959(7)	9660(50)	5060(8)	31(2)
C(15B)	2779(15)	11110(90)	4678(15)	36(2)
C(16B)	2960(7)	11290(50)	4362(8)	29(3)
C(17B)	3375(4)	10250(20)	4473(4)	27(2)
C(18B)	3706(4)	9930(20)	4265(3)	29(1)
C(19B)	3975(5)	7870(20)	4535(4)	30(2)
C(20B)	3970(6)	6130(20)	5269(5)	26(2)
C(21B)	3563(4)	9120(20)	4880(4)	26(1)

C(22B)	3505(5)	9640(20)	3743(3)	30(2)
Cl(1)	5207(3)	7524(14)	4856(3)	82(2)
Cl(2)	4876(5)	10640(20)	4094(4)	97(3)
C(1S')	5179(7)	8190(30)	4316(5)	58(1)
Cl(1S)	4958(1)	11359(4)	4042(1)	58(1)
Cl(2S)	4987(1)	7528(3)	4603(2)	92(1)
C(1S)	5310(2)	9316(9)	4435(2)	58(1)

Supplementary Table 26. Bond lengths [Å] and angles [°] for **3bn'**.

O(1)-C(10)	1.222(3)	C(8)-C(9)	1.432(4)
O(2)-C(23)	1.222(4)	C(10)-C(11)	1.506(17)
N(1)-C(1)	1.318(4)	C(10)-C(11A)	1.523(17)
N(1)-C(9)	1.364(4)	C(10)-C(11B)	1.56(4)
N(2)-H(2)	0.8800	C(23)-C(22)	1.56(2)
N(2)-C(8)	1.398(3)	C(23)-C(22A)	1.54(3)
N(2)-C(10)	1.359(3)	C(23)-C(22B)	1.394(14)
N(4)-H(4)	0.8800	C(24)-C(25)	1.372(4)
N(4)-C(23)	1.350(4)	C(24)-C(29)	1.429(4)
N(4)-C(24)	1.398(3)	C(25)-H(25)	0.9500
N(5)-C(29)	1.358(4)	C(25)-C(26)	1.406(4)
N(5)-C(32)	1.333(4)	C(26)-H(26)	0.9500
C(1)-H(1)	0.9500	C(26)-C(27)	1.354(5)
C(1)-C(2)	1.407(4)	C(27)-H(27)	0.9500
C(2)-H(2A)	0.9500	C(27)-C(28)	1.413(5)
C(2)-C(3)	1.357(4)	C(28)-C(29)	1.422(4)
C(3)-H(3)	0.9500	C(28)-C(30)	1.408(4)
C(3)-C(4)	1.418(4)	C(30)-H(30)	0.9500
C(4)-C(5)	1.419(4)	C(30)-C(31)	1.336(6)
C(4)-C(9)	1.415(4)	C(31)-H(31)	0.9500
C(5)-H(5)	0.9500	C(31)-C(32)	1.409(6)
C(5)-C(6)	1.357(4)	C(32)-H(32)	0.9500
C(6)-H(6)	0.9500	N(3)-C(19)	1.483(9)
C(6)-C(7)	1.418(4)	N(3)-C(20)	1.480(6)
C(7)-H(7)	0.9500	N(3)-C(21)	1.391(6)
C(7)-C(8)	1.372(4)	C(11)-H(11A)	0.9900

C(11)-H(11B)	0.9900	C(14A)-C(15A)	1.411(8)
C(11)-C(12)	1.529(10)	C(15A)-H(15A)	0.9500
C(12)-H(12)	1.0000	C(15A)-C(16A)	1.405(7)
C(12)-C(13)	1.534(6)	C(16A)-H(16A)	0.9500
C(12)-C(20)	1.565(6)	C(16A)-C(17A)	1.397(8)
C(13)-C(14)	1.377(6)	C(17A)-C(18A)	1.536(7)
C(13)-C(21)	1.386(6)	C(17A)-C(21A)	1.358(9)
C(14)-H(14)	0.9500	C(18A)-H(18A)	1.0000
C(14)-C(15)	1.410(7)	C(18A)-C(19A)	1.559(8)
C(15)-H(15)	0.9500	C(18A)-C(22A)	1.520(7)
C(15)-C(16)	1.404(6)	C(19A)-H(19C)	0.9900
C(16)-H(16)	0.9500	C(19A)-H(19D)	0.9900
C(16)-C(17)	1.397(8)	C(20A)-H(20C)	0.9900
C(17)-C(18)	1.536(6)	C(20A)-H(20D)	0.9900
C(17)-C(21)	1.357(9)	C(22A)-H(22C)	0.9900
C(18)-H(18)	1.0000	C(22A)-H(22D)	0.9900
C(18)-C(19)	1.557(7)	N(3B)-C(19B)	1.480(10)
C(18)-C(22)	1.522(6)	N(3B)-C(20B)	1.476(9)
C(19)-H(19A)	0.9900	N(3B)-C(21B)	1.390(8)
C(19)-H(19B)	0.9900	C(11B)-H(11E)	0.9900
C(20)-H(20A)	0.9900	C(11B)-H(11F)	0.9900
C(20)-H(20B)	0.9900	C(11B)-C(12B)	1.527(12)
C(22)-H(22A)	0.9900	C(12B)-H(12B)	1.0000
C(22)-H(22B)	0.9900	C(12B)-C(13B)	1.535(9)
N(3A)-C(19A)	1.484(10)	C(12B)-C(20B)	1.567(9)
N(3A)-C(20A)	1.466(7)	C(13B)-C(14B)	1.373(8)
N(3A)-C(21A)	1.389(7)	C(13B)-C(21B)	1.387(8)
C(11A)-H(11C)	0.9900	C(14B)-H(14B)	0.9500
C(11A)-H(11D)	0.9900	C(14B)-C(15B)	1.411(9)
C(11A)-C(12A)	1.526(11)	C(15B)-H(15B)	0.9500
C(12A)-H(12A)	1.0000	C(15B)-C(16B)	1.406(8)
C(12A)-C(13A)	1.532(7)	C(16B)-H(16B)	0.9500
C(12A)-C(20A)	1.565(7)	C(16B)-C(17B)	1.395(9)
C(13A)-C(14A)	1.371(7)	C(17B)-C(18B)	1.533(8)
C(13A)-C(21A)	1.384(7)	C(17B)-C(21B)	1.361(10)
C(14A)-H(14A)	0.9500	C(18B)-H(18B)	1.0000

C(18B)-C(19B)	1.556(9)	C(9)-C(4)-C(5)	119.6(3)
C(18B)-C(22B)	1.521(9)	C(4)-C(5)-H(5)	120.2
C(19B)-H(19E)	0.9900	C(6)-C(5)-C(4)	119.6(3)
C(19B)-H(19F)	0.9900	C(6)-C(5)-H(5)	120.2
C(20B)-H(20E)	0.9900	C(5)-C(6)-H(6)	119.1
C(20B)-H(20F)	0.9900	C(5)-C(6)-C(7)	121.8(3)
C(22B)-H(22E)	0.9900	C(7)-C(6)-H(6)	119.1
C(22B)-H(22F)	0.9900	C(6)-C(7)-H(7)	120.0
Cl(1)-C(1S')	1.760(8)	C(8)-C(7)-C(6)	120.0(3)
Cl(2)-C(1S')	1.751(8)	C(8)-C(7)-H(7)	120.0
C(1S')-H(1SA)	0.9900	N(2)-C(8)-C(9)	115.0(2)
C(1S')-H(1SB)	0.9900	C(7)-C(8)-N(2)	125.3(2)
Cl(1S)-C(1S)	1.777(4)	C(7)-C(8)-C(9)	119.6(2)
Cl(2S)-C(1S)	1.772(5)	N(1)-C(9)-C(4)	123.4(2)
C(1S)-H(1SC)	0.9900	N(1)-C(9)-C(8)	117.2(2)
C(1S)-H(1SD)	0.9900	C(4)-C(9)-C(8)	119.4(2)
		O(1)-C(10)-N(2)	123.3(3)
C(1)-N(1)-C(9)	117.1(2)	O(1)-C(10)-C(11)	123.2(5)
C(8)-N(2)-H(2)	115.9	O(1)-C(10)-C(11A)	119.3(8)
C(10)-N(2)-H(2)	115.9	O(1)-C(10)-C(11B)	124.4(9)
C(10)-N(2)-C(8)	128.3(2)	N(2)-C(10)-C(11)	113.3(6)
C(23)-N(4)-H(4)	115.4	N(2)-C(10)-C(11A)	116.9(7)
C(23)-N(4)-C(24)	129.2(3)	N(2)-C(10)-C(11B)	111.9(11)
C(24)-N(4)-H(4)	115.4	O(2)-C(23)-N(4)	122.7(3)
C(32)-N(5)-C(29)	116.9(3)	O(2)-C(23)-C(22)	125.3(7)
N(1)-C(1)-H(1)	118.0	O(2)-C(23)-C(22A)	125.3(8)
N(1)-C(1)-C(2)	124.0(3)	O(2)-C(23)-C(22B)	98.5(7)
C(2)-C(1)-H(1)	118.0	N(4)-C(23)-C(22)	112.0(7)
C(1)-C(2)-H(2A)	120.5	N(4)-C(23)-C(22A)	111.6(8)
C(3)-C(2)-C(1)	119.1(3)	N(4)-C(23)-C(22B)	134.6(6)
C(3)-C(2)-H(2A)	120.5	N(4)-C(24)-C(29)	114.6(2)
C(2)-C(3)-H(3)	120.1	C(25)-C(24)-N(4)	125.7(2)
C(2)-C(3)-C(4)	119.7(3)	C(25)-C(24)-C(29)	119.7(2)
C(4)-C(3)-H(3)	120.1	C(24)-C(25)-H(25)	120.0
C(3)-C(4)-C(5)	123.7(3)	C(24)-C(25)-C(26)	120.1(3)
C(9)-C(4)-C(3)	116.7(3)	C(26)-C(25)-H(25)	120.0

C(25)-C(26)-H(26)	119.2	C(14)-C(13)-C(12)	137.7(5)
C(27)-C(26)-C(25)	121.7(3)	C(14)-C(13)-C(21)	115.9(5)
C(27)-C(26)-H(26)	119.2	C(21)-C(13)-C(12)	105.4(4)
C(26)-C(27)-H(27)	119.9	C(13)-C(14)-H(14)	120.6
C(26)-C(27)-C(28)	120.2(3)	C(13)-C(14)-C(15)	118.7(6)
C(28)-C(27)-H(27)	119.9	C(15)-C(14)-H(14)	120.6
C(27)-C(28)-C(29)	119.1(3)	C(14)-C(15)-H(15)	118.5
C(30)-C(28)-C(27)	123.8(3)	C(16)-C(15)-C(14)	123.1(7)
C(30)-C(28)-C(29)	117.2(3)	C(16)-C(15)-H(15)	118.5
N(5)-C(29)-C(24)	117.8(2)	C(15)-C(16)-H(16)	121.1
N(5)-C(29)-C(28)	123.0(3)	C(17)-C(16)-C(15)	117.8(7)
C(28)-C(29)-C(24)	119.2(3)	C(17)-C(16)-H(16)	121.1
C(28)-C(30)-H(30)	120.1	C(16)-C(17)-C(18)	137.1(9)
C(31)-C(30)-C(28)	119.8(3)	C(21)-C(17)-C(16)	116.6(6)
C(31)-C(30)-H(30)	120.1	C(21)-C(17)-C(18)	105.5(5)
C(30)-C(31)-H(31)	120.1	C(17)-C(18)-H(18)	109.1
C(30)-C(31)-C(32)	119.8(3)	C(17)-C(18)-C(19)	101.1(5)
C(32)-C(31)-H(31)	120.1	C(19)-C(18)-H(18)	109.1
N(5)-C(32)-C(31)	123.4(3)	C(22)-C(18)-C(17)	116.0(8)
N(5)-C(32)-H(32)	118.3	C(22)-C(18)-H(18)	109.1
C(31)-C(32)-H(32)	118.3	C(22)-C(18)-C(19)	111.9(7)
C(20)-N(3)-C(19)	121.8(6)	N(3)-C(19)-C(18)	104.2(7)
C(21)-N(3)-C(19)	102.6(5)	N(3)-C(19)-H(19A)	110.9
C(21)-N(3)-C(20)	104.6(4)	N(3)-C(19)-H(19B)	110.9
C(10)-C(11)-H(11A)	109.0	C(18)-C(19)-H(19A)	110.9
C(10)-C(11)-H(11B)	109.0	C(18)-C(19)-H(19B)	110.9
C(10)-C(11)-C(12)	113.0(11)	H(19A)-C(19)-H(19B)	108.9
H(11A)-C(11)-H(11B)	107.8	N(3)-C(20)-C(12)	104.0(4)
C(12)-C(11)-H(11A)	109.0	N(3)-C(20)-H(20A)	111.0
C(12)-C(11)-H(11B)	109.0	N(3)-C(20)-H(20B)	111.0
C(11)-C(12)-H(12)	109.3	C(12)-C(20)-H(20A)	111.0
C(11)-C(12)-C(13)	112.3(6)	C(12)-C(20)-H(20B)	111.0
C(11)-C(12)-C(20)	113.9(7)	H(20A)-C(20)-H(20B)	109.0
C(13)-C(12)-H(12)	109.3	C(13)-C(21)-N(3)	115.3(4)
C(13)-C(12)-C(20)	102.5(4)	C(17)-C(21)-N(3)	116.4(5)
C(20)-C(12)-H(12)	109.3	C(17)-C(21)-C(13)	127.8(5)

C(23)-C(22)-H(22A)	109.6
C(23)-C(22)-H(22B)	109.6
C(18)-C(22)-C(23)	110.5(14)
C(18)-C(22)-H(22A)	109.6
C(18)-C(22)-H(22B)	109.6
H(22A)-C(22)-H(22B)	108.1
C(20A)-N(3A)-C(19A)	124.2(7)
C(21A)-N(3A)-C(19A)	102.8(6)
C(21A)-N(3A)-C(20A)	104.9(5)
C(10)-C(11A)-H(11C)	109.7
C(10)-C(11A)-H(11D)	109.7
C(10)-C(11A)-C(12A)	109.9(11)
H(11C)-C(11A)-H(11D)	108.2
C(12A)-C(11A)-H(11C)	109.7
C(12A)-C(11A)-H(11D)	109.7
C(11A)-C(12A)-H(12A)	109.1
C(11A)-C(12A)-C(13A)	113.2(8)
C(11A)-C(12A)-C(20A)	114.0(8)
C(13A)-C(12A)-H(12A)	109.1
C(13A)-C(12A)-C(20A)	101.9(5)
C(20A)-C(12A)-H(12A)	109.1
C(14A)-C(13A)-C(12A)	139.3(6)
C(14A)-C(13A)-C(21A)	115.6(6)
C(21A)-C(13A)-C(12A)	105.1(5)
C(13A)-C(14A)-H(14A)	120.5
C(13A)-C(14A)-C(15A)	119.0(7)
C(15A)-C(14A)-H(14A)	120.5
C(14A)-C(15A)-H(15A)	118.5
C(16A)-C(15A)-C(14A)	122.9(8)
C(16A)-C(15A)-H(15A)	118.5
C(15A)-C(16A)-H(16A)	121.2
C(17A)-C(16A)-C(15A)	117.6(8)
C(17A)-C(16A)-H(16A)	121.2
C(16A)-C(17A)-C(18A)	138.3(8)
C(21A)-C(17A)-C(16A)	116.5(7)
C(21A)-C(17A)-C(18A)	105.0(6)

C(17A)-C(18A)-H(18A)	109.0
C(17A)-C(18A)-C(19A)	100.8(6)
C(19A)-C(18A)-H(18A)	109.0
C(22A)-C(18A)-C(17A)	116.5(9)
C(22A)-C(18A)-H(18A)	109.0
C(22A)-C(18A)-C(19A)	112.2(9)
N(3A)-C(19A)-C(18A)	103.2(7)
N(3A)-C(19A)-H(19C)	111.1
N(3A)-C(19A)-H(19D)	111.1
C(18A)-C(19A)-H(19C)	111.1
C(18A)-C(19A)-H(19D)	111.1
H(19C)-C(19A)-H(19D)	109.1
N(3A)-C(20A)-C(12A)	103.8(5)
N(3A)-C(20A)-H(20C)	111.0
N(3A)-C(20A)-H(20D)	111.0
C(12A)-C(20A)-H(20C)	111.0
C(12A)-C(20A)-H(20D)	111.0
H(20C)-C(20A)-H(20D)	109.0
C(13A)-C(21A)-N(3A)	115.2(5)
C(17A)-C(21A)-N(3A)	116.3(5)
C(17A)-C(21A)-C(13A)	127.9(6)
C(23)-C(22A)-H(22C)	108.8
C(23)-C(22A)-H(22D)	108.8
C(18A)-C(22A)-C(23)	114.0(17)
C(18A)-C(22A)-H(22C)	108.8
C(18A)-C(22A)-H(22D)	108.8
H(22C)-C(22A)-H(22D)	107.6
C(20B)-N(3B)-C(19B)	124.1(10)
C(21B)-N(3B)-C(19B)	103.2(7)
C(21B)-N(3B)-C(20B)	104.6(7)
C(10)-C(11B)-H(11E)	108.7
C(10)-C(11B)-H(11F)	108.7
H(11E)-C(11B)-H(11F)	107.6
C(12B)-C(11B)-C(10)	114(3)
C(12B)-C(11B)-H(11E)	108.7
C(12B)-C(11B)-H(11F)	108.7

C(11B)-C(12B)-H(12B)	109.5
C(11B)-C(12B)-C(13B)	112.7(12)
C(11B)-C(12B)-C(20B)	114.1(12)
C(13B)-C(12B)-H(12B)	109.5
C(13B)-C(12B)-C(20B)	101.1(7)
C(20B)-C(12B)-H(12B)	109.5
C(14B)-C(13B)-C(12B)	139.3(8)
C(14B)-C(13B)-C(21B)	115.8(8)
C(21B)-C(13B)-C(12B)	104.6(7)
C(13B)-C(14B)-H(14B)	120.9
C(13B)-C(14B)-C(15B)	118.3(9)
C(15B)-C(14B)-H(14B)	120.9
C(14B)-C(15B)-H(15B)	118.6
C(16B)-C(15B)-C(14B)	122.9(10)
C(16B)-C(15B)-H(15B)	118.6
C(15B)-C(16B)-H(16B)	121.1
C(17B)-C(16B)-C(15B)	117.8(10)
C(17B)-C(16B)-H(16B)	121.1
C(16B)-C(17B)-C(18B)	138.7(9)
C(21B)-C(17B)-C(16B)	116.0(8)
C(21B)-C(17B)-C(18B)	105.2(7)
C(17B)-C(18B)-H(18B)	108.4
C(17B)-C(18B)-C(19B)	101.0(7)
C(19B)-C(18B)-H(18B)	108.4
C(22B)-C(18B)-C(17B)	118.0(9)
C(22B)-C(18B)-H(18B)	108.4
C(22B)-C(18B)-C(19B)	112.2(9)
N(3B)-C(19B)-C(18B)	103.9(7)
N(3B)-C(19B)-H(19E)	111.0
N(3B)-C(19B)-H(19F)	111.0
C(18B)-C(19B)-H(19E)	111.0
C(18B)-C(19B)-H(19F)	111.0
H(19E)-C(19B)-H(19F)	109.0
N(3B)-C(20B)-C(12B)	102.6(8)
N(3B)-C(20B)-H(20E)	111.2
N(3B)-C(20B)-H(20F)	111.2
C(12B)-C(20B)-H(20E)	111.2
C(12B)-C(20B)-H(20F)	111.2
H(20E)-C(20B)-H(20F)	109.2
C(13B)-C(21B)-N(3B)	115.1(7)
C(17B)-C(21B)-N(3B)	116.3(7)
C(17B)-C(21B)-C(13B)	127.4(8)
C(23)-C(22B)-C(18B)	102.9(9)
C(23)-C(22B)-H(22E)	111.2
C(23)-C(22B)-H(22F)	111.2
C(18B)-C(22B)-H(22E)	111.2
C(18B)-C(22B)-H(22F)	111.2
H(22E)-C(22B)-H(22F)	109.1
Cl(1)-C(1S')-H(1SA)	109.3
Cl(1)-C(1S')-H(1SB)	109.3
Cl(2)-C(1S')-Cl(1)	111.4(8)
Cl(2)-C(1S')-H(1SA)	109.3
Cl(2)-C(1S')-H(1SB)	109.3
H(1SA)-C(1S')-H(1SB)	108.0
Cl(1S)-C(1S)-H(1SC)	109.3
Cl(1S)-C(1S)-H(1SD)	109.3
Cl(2S)-C(1S)-Cl(1S)	111.5(3)
Cl(2S)-C(1S)-H(1SC)	109.3
Cl(2S)-C(1S)-H(1SD)	109.3
H(1SC)-C(1S)-H(1SD)	108.0

Symmetry transformations used to generate equivalent atoms:

Supplementary Table 27. Anisotropic displacement parameters ($Å^2$x 10^3) for Engle271. The anisotropic
displacement factor exponent takes the form: $-2\pi^2$[h^2 $a^{*2}U^{11}$ + ... + 2 h k a* b* U^{12}]

	U^{11}	U^{22}	U^{33}	U^{23}	U^{13}	U^{12}
O(1)	30(1)	53(1)	39(1)	12(1)	19(1)	0(1)
O(2)	58(2)	47(1)	49(1)	-2(1)	36(1)	-15(1)
N(1)	29(1)	32(1)	33(1)	3(1)	16(1)	-1(1)
N(2)	25(1)	32(1)	28(1)	2(1)	14(1)	0(1)
N(4)	39(1)	32(1)	36(1)	8(1)	24(1)	7(1)
N(5)	27(1)	60(2)	31(1)	1(1)	12(1)	2(1)
C(1)	29(2)	43(2)	43(2)	4(1)	18(1)	-2(1)
C(2)	42(2)	39(2)	44(2)	3(1)	24(1)	-10(1)
C(3)	46(2)	27(1)	33(1)	2(1)	20(1)	-2(1)
C(4)	38(2)	28(1)	23(1)	-2(1)	15(1)	1(1)
C(5)	44(2)	32(1)	30(1)	3(1)	17(1)	9(1)
C(6)	32(2)	45(2)	33(1)	2(1)	14(1)	14(1)
C(7)	29(1)	41(2)	28(1)	1(1)	15(1)	1(1)
C(8)	27(1)	30(1)	21(1)	-2(1)	11(1)	0(1)
C(9)	31(1)	27(1)	25(1)	-4(1)	14(1)	-2(1)
C(10)	29(1)	36(2)	27(1)	-1(1)	16(1)	-5(1)
C(23)	55(2)	34(2)	46(2)	-1(1)	40(2)	-1(1)
C(24)	31(1)	25(1)	24(1)	-2(1)	15(1)	0(1)
C(25)	30(1)	44(2)	30(1)	0(1)	17(1)	2(1)
C(26)	46(2)	49(2)	33(1)	8(1)	19(1)	22(2)
C(27)	70(2)	30(2)	33(1)	7(1)	27(2)	12(2)
C(28)	51(2)	33(2)	26(1)	-6(1)	20(1)	-11(1)
C(29)	28(1)	34(1)	22(1)	-4(1)	11(1)	-5(1)
C(30)	65(2)	52(2)	43(2)	-6(2)	26(2)	-28(2)
C(31)	51(2)	89(3)	49(2)	-8(2)	25(2)	-36(2)
C(32)	25(2)	95(3)	47(2)	-9(2)	13(1)	-11(2)
N(3)	33(2)	27(2)	31(2)	4(2)	20(2)	3(2)

C(11)	30(4)	33(4)	32(3)	2(3)	17(4)	-4(3)
C(12)	29(2)	32(3)	24(2)	-2(2)	13(2)	-4(2)
C(13)	30(3)	32(2)	26(2)	-1(2)	15(2)	-4(2)
C(14)	32(5)	37(3)	28(3)	-5(2)	18(4)	-1(3)
C(15)	35(2)	38(5)	42(2)	5(2)	25(1)	11(2)
C(16)	28(5)	32(5)	29(2)	4(3)	13(3)	1(5)
C(17)	29(3)	30(4)	26(2)	-1(3)	15(3)	-4(3)
C(18)	33(3)	33(2)	27(2)	1(2)	17(2)	-1(2)
C(19)	34(5)	33(3)	31(4)	1(3)	21(4)	2(3)
C(20)	29(4)	25(4)	27(3)	-2(3)	15(3)	-4(3)
C(21)	27(2)	24(3)	31(3)	-3(2)	18(2)	-4(2)
C(22)	33(2)	31(3)	33(4)	3(3)	22(3)	4(3)
N(3A)	33(2)	27(2)	31(2)	4(2)	20(2)	3(2)
C(11A)	30(4)	33(4)	32(3)	2(3)	17(4)	-4(3)
C(12A)	29(2)	32(3)	24(2)	-2(2)	13(2)	-4(2)
C(13A)	30(3)	32(2)	26(2)	-1(2)	15(2)	-4(2)
C(14A)	32(5)	37(3)	28(3)	-5(2)	18(4)	-1(3)
C(15A)	35(2)	38(5)	42(2)	5(2)	25(1)	11(2)
C(16A)	28(5)	32(5)	29(2)	4(3)	13(3)	1(5)
C(17A)	29(3)	30(4)	26(2)	-1(3)	15(3)	-4(3)
C(18A)	33(3)	33(2)	27(2)	1(2)	17(2)	-1(2)
C(19A)	34(5)	33(3)	31(4)	1(3)	21(4)	2(3)
C(20A)	29(4)	25(4)	27(3)	-2(3)	15(3)	-4(3)
C(21A)	27(2)	24(3)	31(3)	-3(2)	18(2)	-4(2)
C(22A)	33(2)	31(3)	33(4)	3(3)	22(3)	4(3)
N(3B)	33(2)	27(2)	31(2)	4(2)	20(2)	3(2)
C(11B)	30(4)	33(4)	32(3)	2(3)	17(4)	-4(3)
C(12B)	29(2)	32(3)	24(2)	-2(2)	13(2)	-4(2)
C(13B)	30(3)	32(2)	26(2)	-1(2)	15(2)	-4(2)
C(14B)	32(5)	37(3)	28(3)	-5(2)	18(4)	-1(3)
C(15B)	35(2)	38(5)	42(2)	5(2)	25(1)	11(2)
C(16B)	28(5)	32(5)	29(2)	4(3)	13(3)	1(5)
C(17B)	29(3)	30(4)	26(2)	-1(3)	15(3)	-4(3)
C(18B)	33(3)	33(2)	27(2)	1(2)	17(2)	-1(2)
C(19B)	34(5)	33(3)	31(4)	1(3)	21(4)	2(3)
C(20B)	29(4)	25(4)	27(3)	-2(3)	15(3)	-4(3)

C(21B)	27(2)	24(3)	31(3)	-3(2)	18(2)	-4(2)
C(22B)	33(2)	31(3)	33(4)	3(3)	22(3)	4(3)
Cl(1)	49(4)	118(5)	77(4)	15(4)	28(4)	14(4)
Cl(2)	95(7)	95(7)	104(5)	49(5)	49(5)	47(6)
C(1S')	54(3)	52(3)	73(3)	19(3)	32(3)	12(2)
Cl(1S)	51(1)	61(1)	64(1)	15(1)	29(1)	16(1)
Cl(2S)	74(2)	75(1)	143(3)	41(1)	64(2)	16(1)
C(1S)	54(3)	52(3)	73(3)	19(3)	32(3)	12(2)

Table S28. Hydrogen coordinates (x 10^4) and isotropic displacement parameters (Å^{2}x 10 ^{3}) for **3bn’**.

	x	y	z	U(eq)
H(2)	3298	2599	6133	33
H(4)	4009	12680	3553	40
H(1)	2383	-525	6335	45
H(2A)	2540	-3867	6732	48
H(3)	3269	-5334	7033	41
H(5)	4096	-4921	7160	42
H(6)	4614	-2952	7017	44
H(7)	4403	315	6580	38
H(25)	2916	15126	2986	40
H(26)	2777	18373	2554	51
H(27)	3348	20132	2468	52
H(30)	4178	20166	2613	63
H(31)	4877	18477	2936	75
H(32)	4996	15323	3388	68
H(11A)	3351	6152	5844	36
H(11B)	3231	4456	5428	36
H(12)	3993	7451	5751	34
H(14)	2948	9704	5339	37
H(15)	2517	11541	4635	43

H(16)	2709	11405	4021	35
H(18)	3209	7932	3753	35
H(19A)	3544	5115	4254	37
H(19B)	4011	6169	4297	37
H(20A)	4231	4988	5369	32
H(20B)	3720	4144	5036	32
H(22A)	3940	9166	3814	36
H(22B)	3988	10736	4231	36
H(11C)	3752	6882	5877	36
H(11D)	3286	5902	5844	36
H(12A)	3127	4095	5142	34
H(14A)	2751	9135	5188	37
H(15A)	2599	12027	4659	43
H(16A)	2922	12151	4144	35
H(18A)	3308	7691	3786	35
H(19C)	3933	5592	4271	37
H(19D)	4114	7412	4677	37
H(20C)	4030	5475	5308	32
H(20D)	3782	3174	5091	32
H(22C)	4007	9586	3913	36
H(22D)	3936	11150	4271	36
H(11E)	3248	4419	5387	36
H(11F)	3279	5933	5803	36
H(12B)	3908	7873	5805	34
H(14B)	2807	9447	5246	37
H(15B)	2524	11997	4632	43
H(16B)	2805	12098	4081	35
H(18B)	3922	11205	4359	35
H(19E)	3822	6502	4371	37
H(19F)	4292	7883	4574	37
H(20E)	4285	5699	5493	32
H(20F)	3814	4856	5070	32
H(22E)	3711	8762	3660	36
H(22F)	3202	8920	3614	36
H(1SA)	5493	8357	4351	70
H(1SB)	5028	6986	4094	70

H(1SC)	5462	8453	4286	70
H(1SD)	5550	10035	4714	70

—

—

COMPUTATIONAL DETAILS

All calculations were carried out using Gaussian 16.[17] Geometries of intermediates and transition states were optimized using the dispersion-corrected B3LYP functional,[18] using Grimme's D3 dispersion correction,[19] with a mixed basis set of SDD for Pd, I, and 6-31G(d) for other atoms in the gas phase. Vibrational frequency calculations were performed for all the stationary points to confirm if each optimized structure is a local minimum or a transition state structure. Solvation energy corrections were calculated in HFIP solvent with the SMD continuum solvation model[20] based on the gas-phase optimized geometries. The M06 functional[21] with a mixed basis set of SDD for Pd, I, and 6-311++G(d,p) for other atoms was used for solvation single-point energy calculations. Truhlar's quasi-harmonic corrections[22] were applied for entropy calculations using 100 cm^{-1} as the frequency cutoff. Translational entropy in HFIP solution was calculated using the method proposed by Whitesides.[23]

Additional Computational Results

Several nucleopalladation mechanisms were explored. The anionic *anti*-addition (**TS1**) was found to be the most favorable pathway.

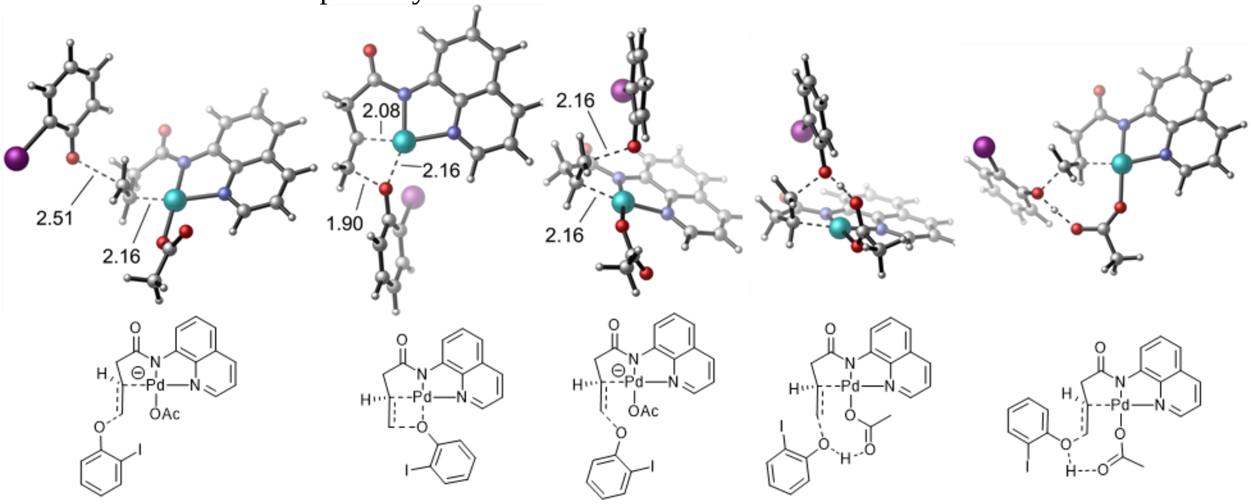

TS1 $\Delta G^{‡} = 18.0$ **TS2** $\Delta G^{‡} = 33.9$ **TS10** $\Delta G^{‡} = 29.6$ **TS11** $\Delta G^{‡} = 30.7$ **TS12** $\Delta G^{‡} = 38.5$

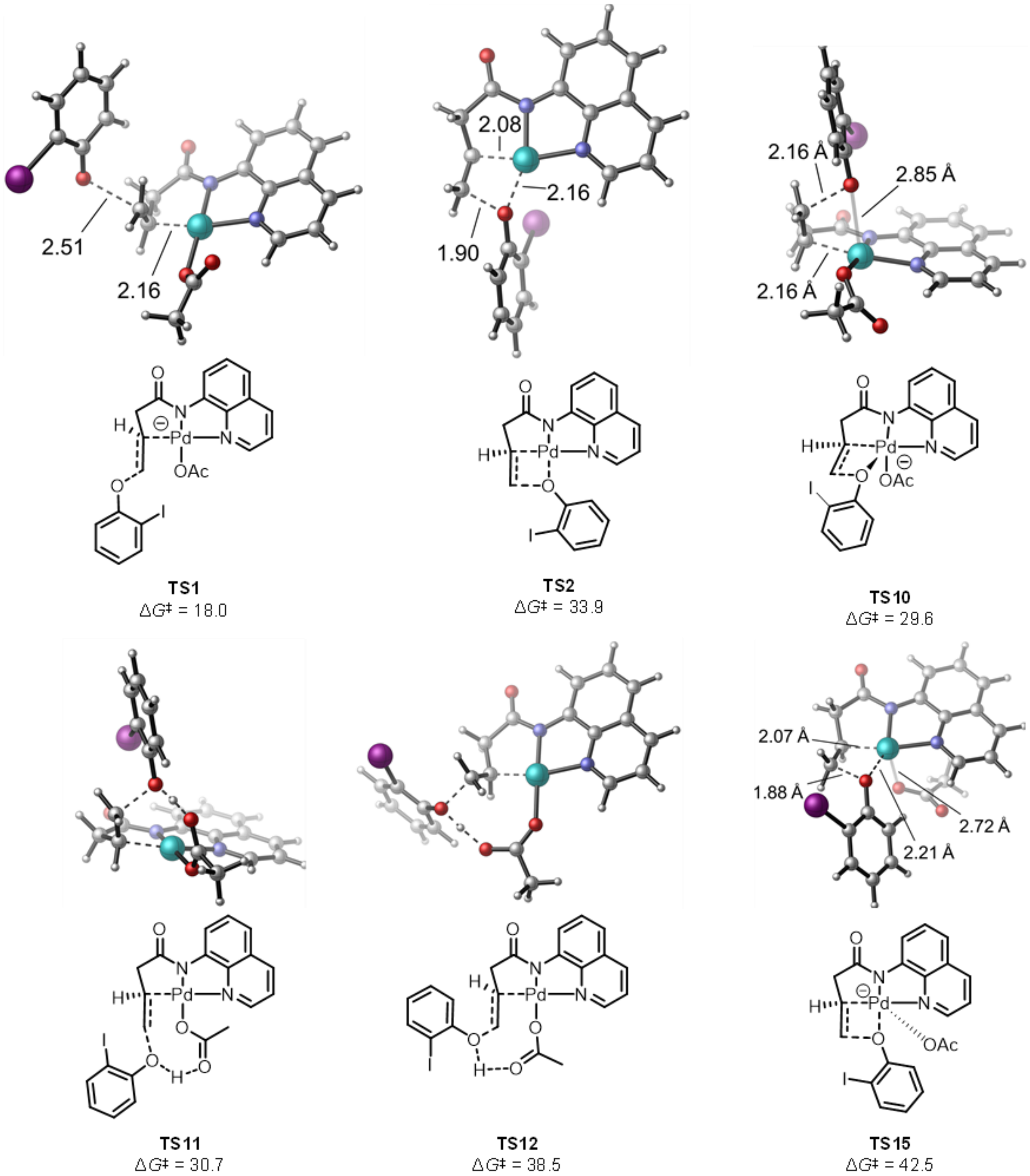

Supplementary Figure 20. Structures and energies of different nucleopalladation transition states. All energies are in kcal/mol with respect to complex **10**.

The intermolecular coupling reaction between complex **12** and **2aa** was computed to compare to the intramolecular coupling reaction. The calculations show that the intramolecular pathway is favored not only entropically, but also by decreased steric repulsions. The enthalpies (ΔH) of the intermolecular oxidative addition (**TS13**) and the reductive elimination (**TS14**) transition states are higher than those of the intramolecular pathway (**TS3** and **TS5**, respectively). This suggests that the ring strain associated with the intramolecular reaction is relatively small and outweighed by the steric repulsion with the ortho OH group in intermolecular reaction.

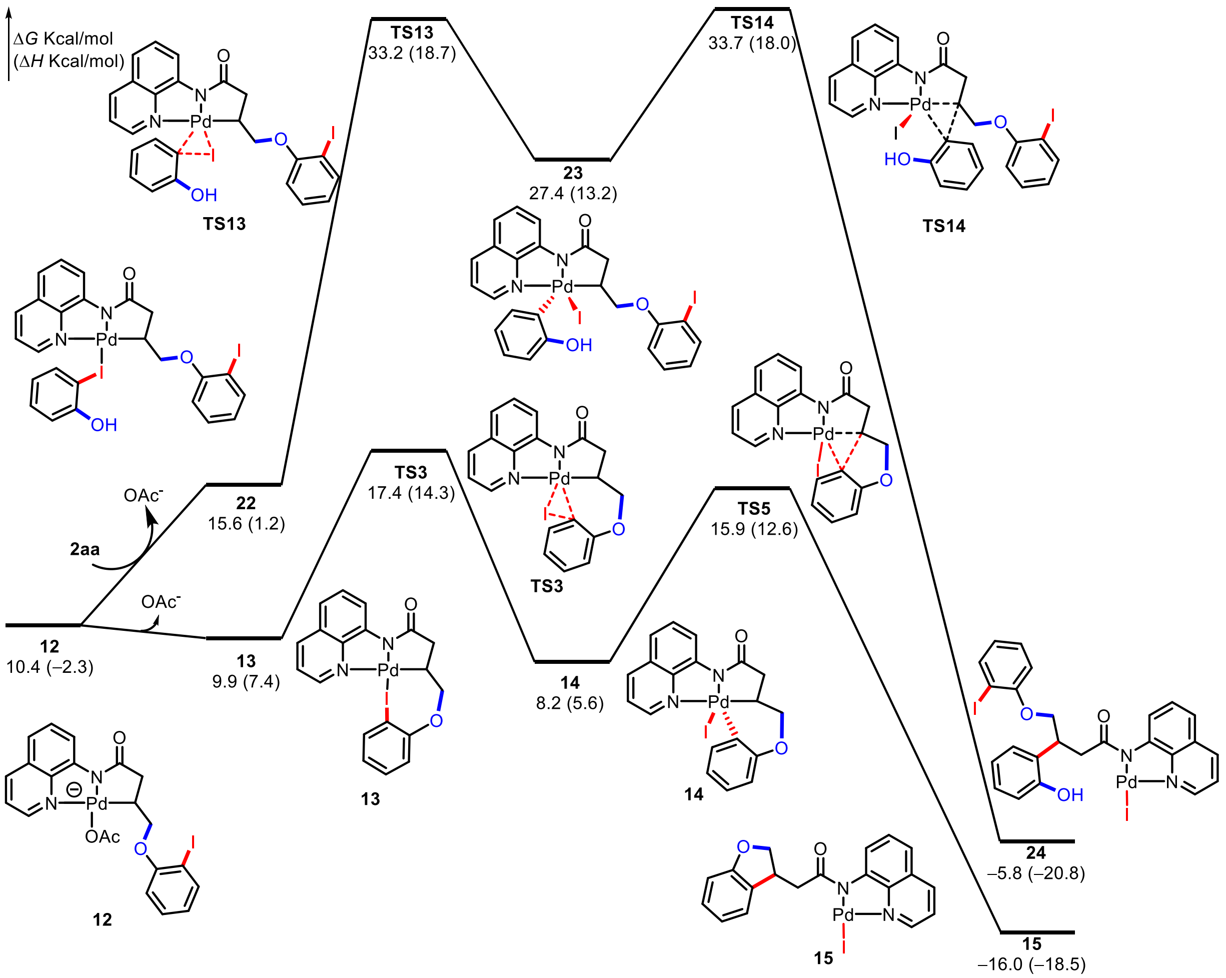

Supplementary Figure 21. Comparison of intramolecular and intermolecular arylations of complex **12**. All energies are with respect to complex **10** and **2aa**.

Other possible conformations for the intramolecular oxidative addition transition state were also considered. Puckering of the methylene group (**TS3b**) causes highlighted dihedral to adopt an almost eclipsed conformation, which destabilizes the transition state. Placement of the iodo- group anti to the α-C-H (**TS3c**) can cause increased strain on the palladacycle destabilizing the transition state.

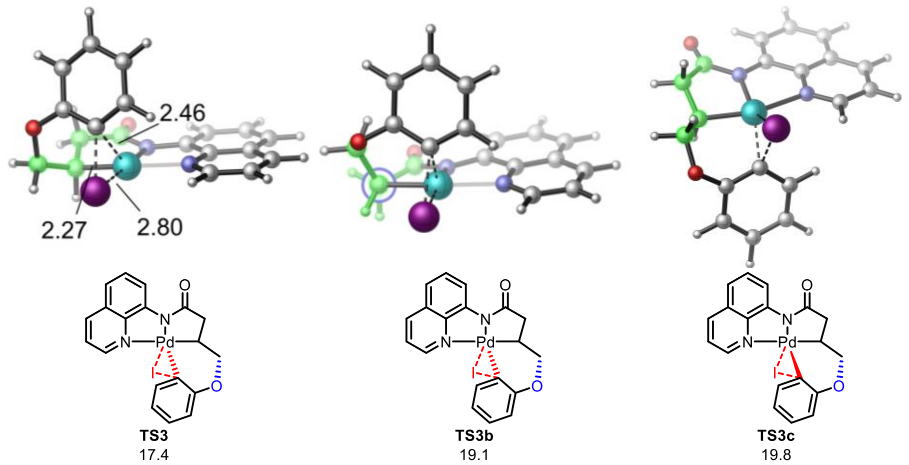

Supplementary Figure 22. Structures and energies of oxidative addition transition states. All Gibbs free energies are in kcal/mol with respect to complex **10** and **2aa**.

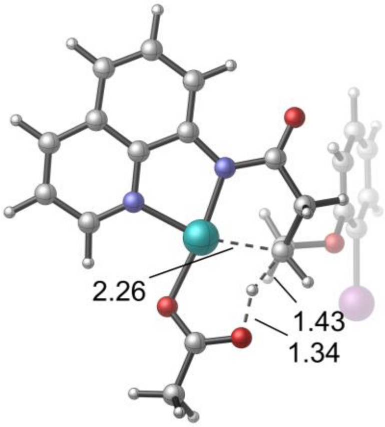

TS4

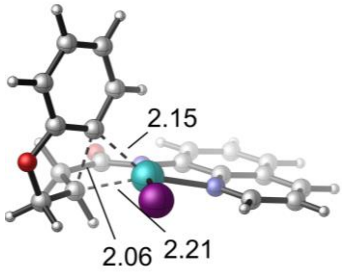

TS5

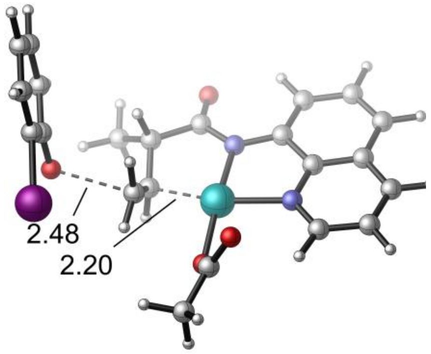

TS6

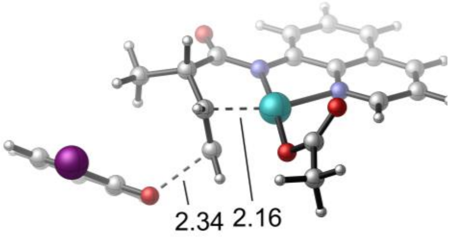

TS7

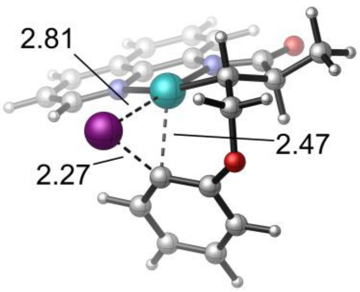

TS8

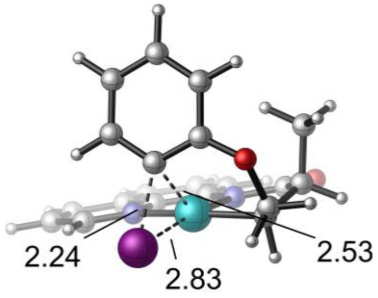

TS9

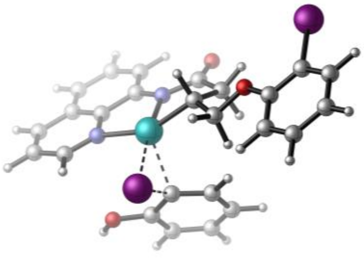

TS13

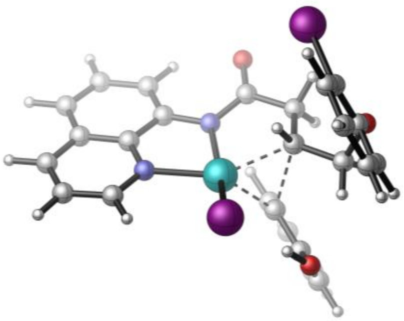

TS14

Supplementary Figure 23. 3D Structures of other transition states.

***Supplementary Table 29.* Comparison of free energies of key TS and intermediates using different levels of theory for geometry optimization.** All energies are in kcal/mol relative to **10** and **2aa**. In all calculations, single point energies were calculated at the M06/SDD–6-311++G(d,p) level using the SMD solvation model in HFIP.

**Structure**	**Method for Geometry Optimization**	
	B3LYP-D3/SDD–6-31G(d)	M06/SDD–6-31G(d)
**10**	0.0	0.0
**TS1**	18.2	18.0
**TS2**	33.4	33.9
**13**	10.0	9.9
**TS3**	18.6	17.4

Both B3LYP-D3 and M06 were used to optimize key intermediates and transition state structures. These results show that there is no significant difference between these two methods. All computed values reported in the main manuscript were calculated using M06 for single point and B3LYP-D3 for geometry optimization.

NMR SPECTRA

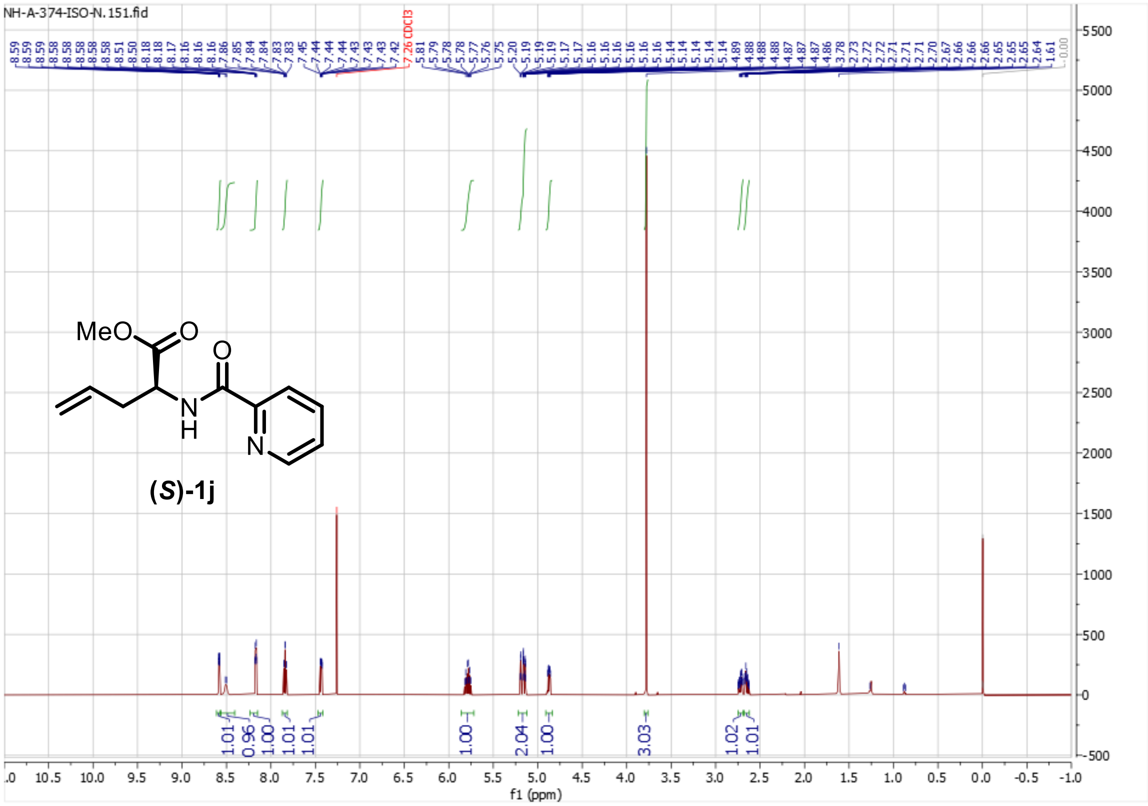

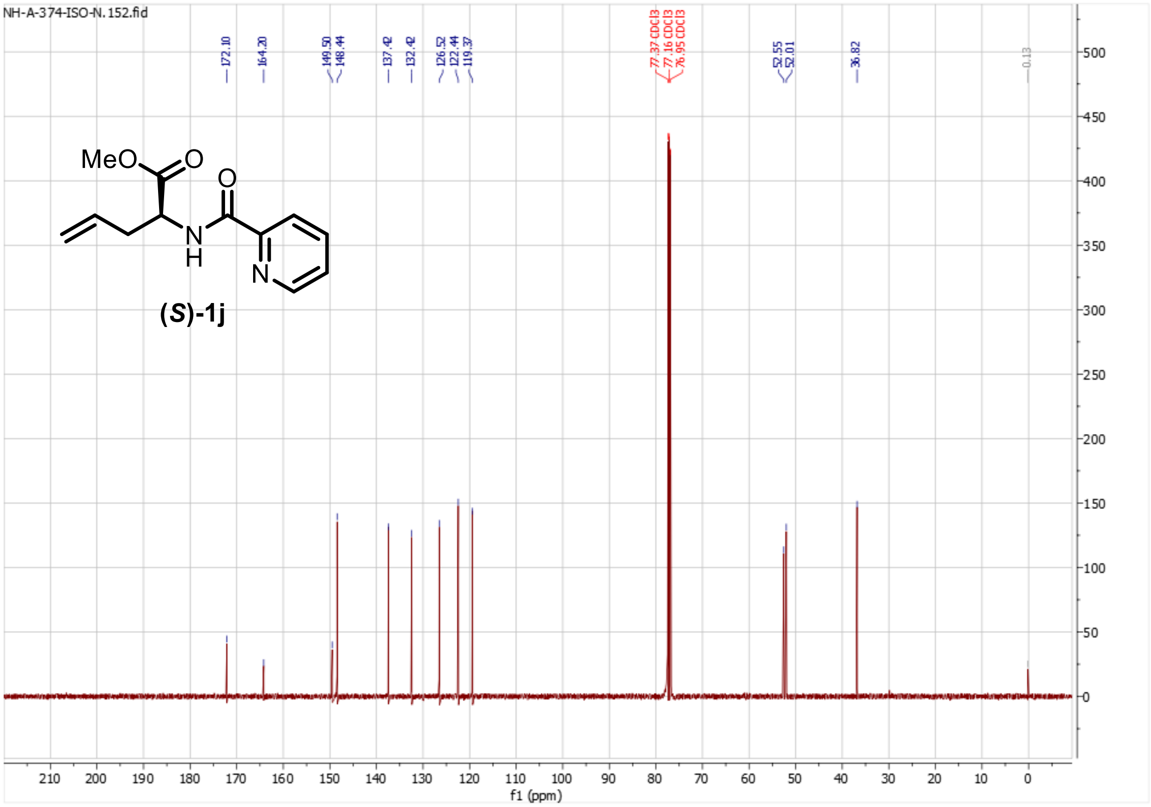
NH-A-374-ISO-N.152.fid
172.10
164.20
149.50
148.44
137.42
132.42
126.52
122.44
119.37
77.37 CDCl3
77.16 CDCl3
76.95 CDCl3
52.55
52.01
36.82
0.13
MeO
O
O
N
H
N
(S)-1j
f1 (ppm)

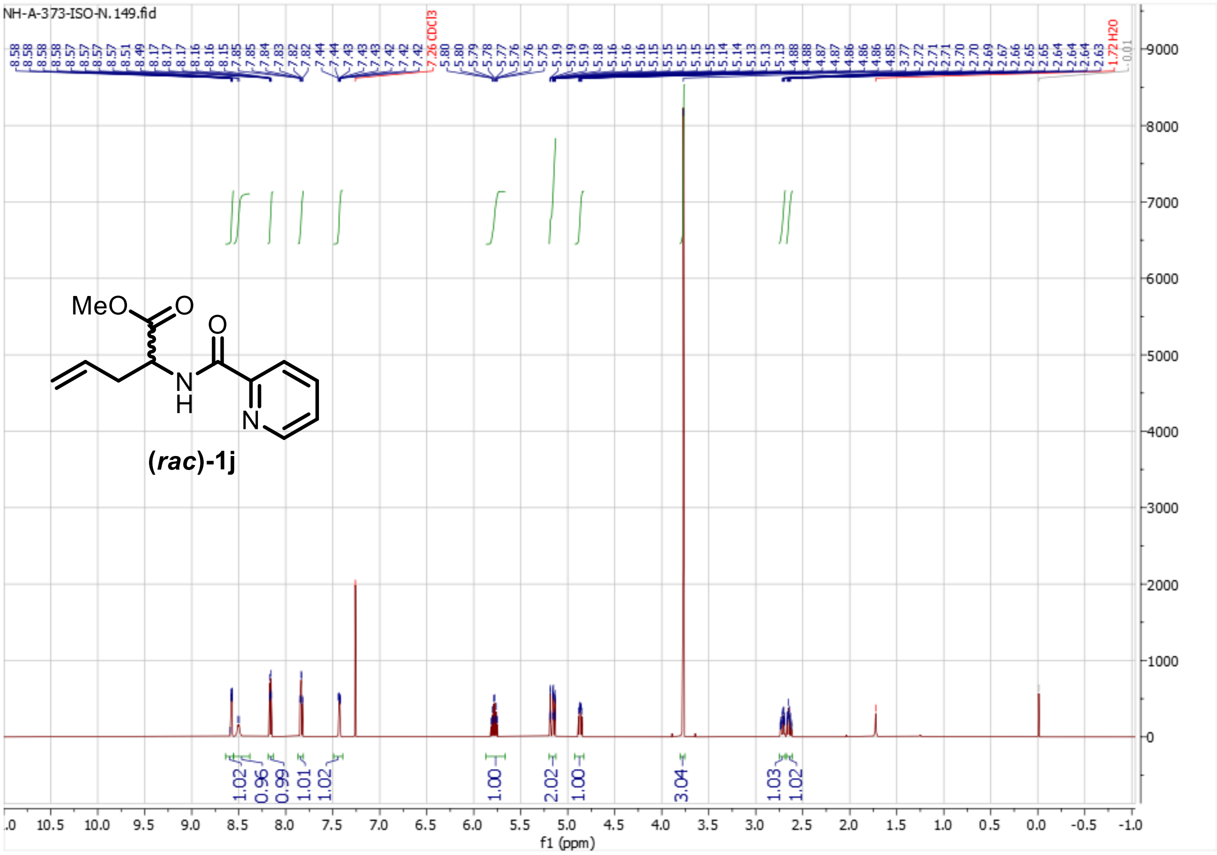
NH-A-373-ISO-N.149.fid
7.26 CDCl3
1.72 H2O
MeO
O
O
N
H
N
(rac)-1j
1.02
0.96
0.99
1.01
1.02
1.00
2.02
1.00
3.04
1.03
1.02
f1 (ppm)

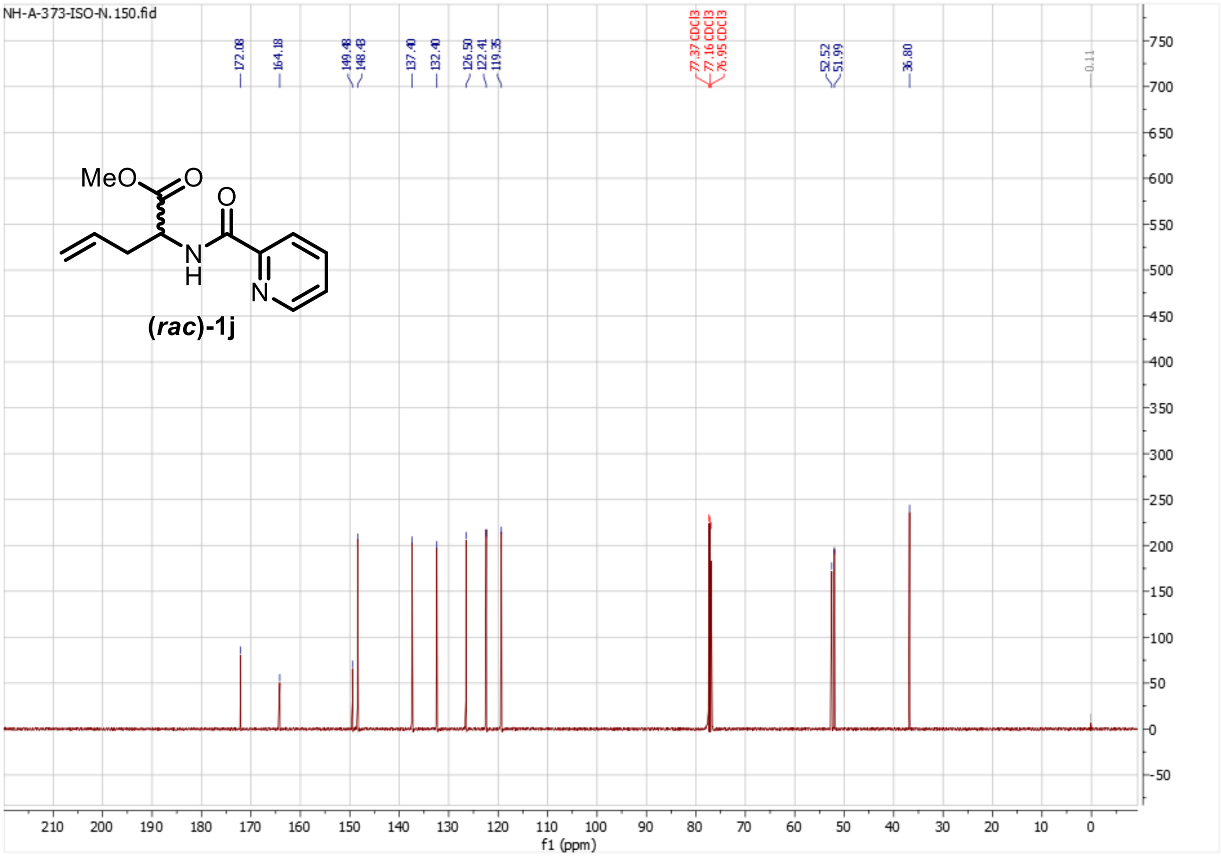
NH-A-373-ISO-N.150.fid
172.08
164.18
149.48
148.43
137.40
132.40
126.50
122.41
119.35
77.37 CDCl3
77.16 CDCl3
76.95 CDCl3
52.52
51.99
36.80
MeO
O
O
N
H
N
(rac)-1j
f1 (ppm)

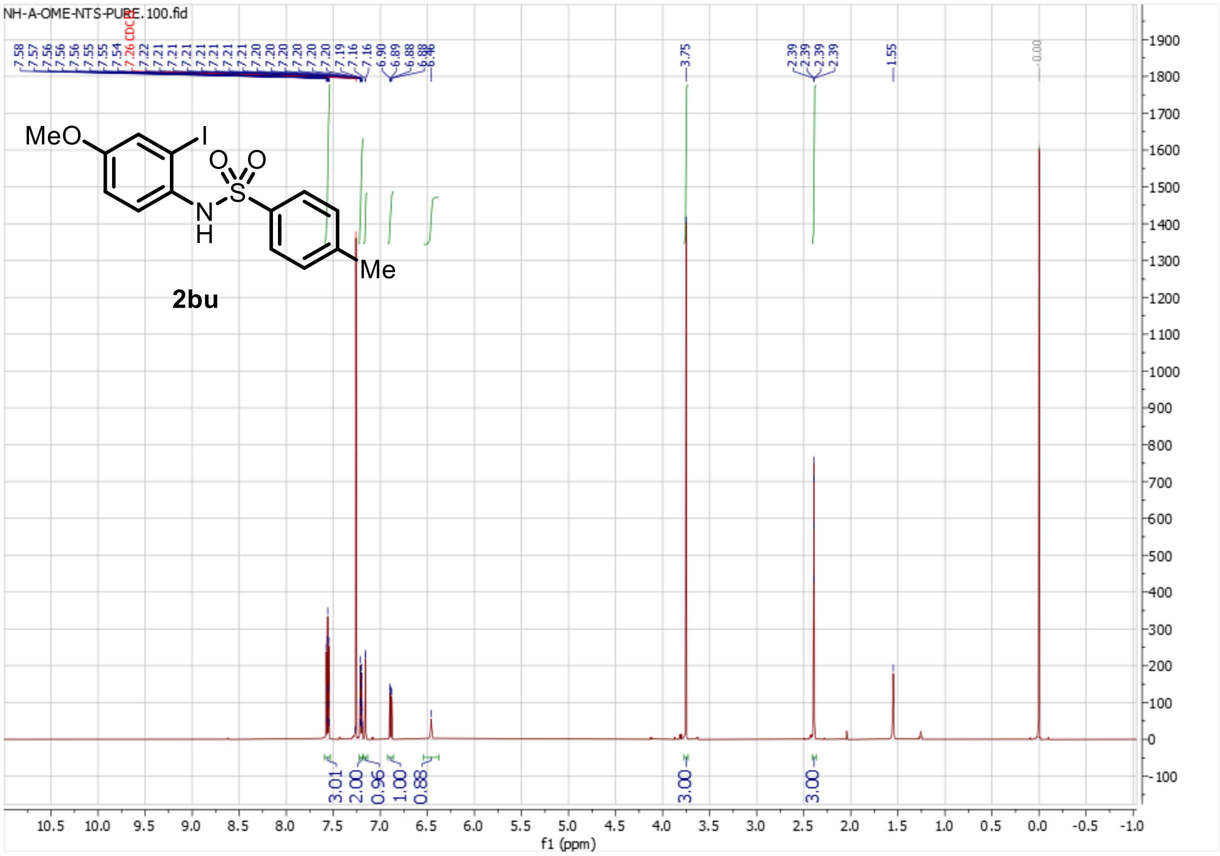
NH-A-OME-NTS-PURE.100.fid
7.26 CDCl3
3.75
1.55
MeO
I
O
O
S
N
H
Me
2bu
3.01
2.00
0.96
1.00
0.88
3.00
3.00
f1 (ppm)

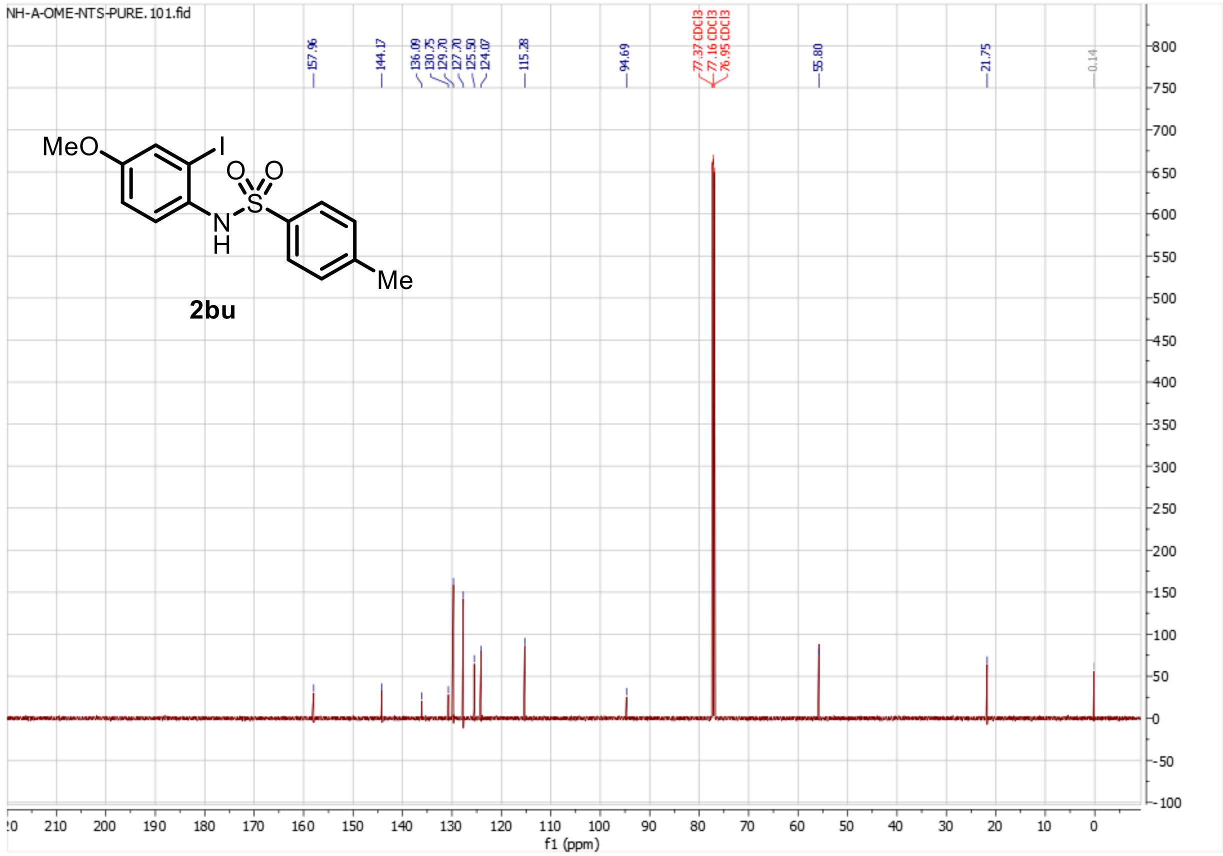
NH-A-OME-NTS-PURE.101.fid
MeO
I
O
O
S
N
H
Me
2bu
f1 (ppm)

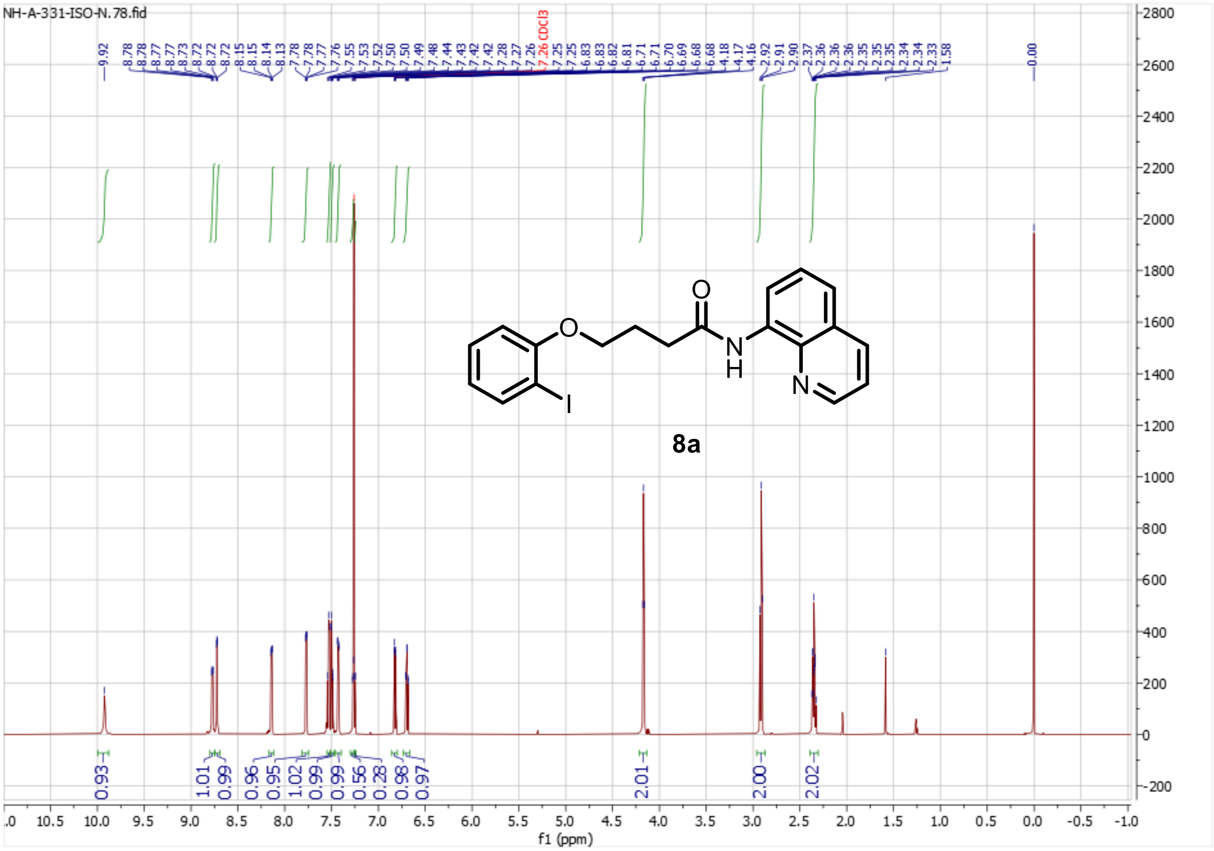
NH-A-331-ISO-N.78.fid
O
O
N
H
N
I
8a
f1 (ppm)

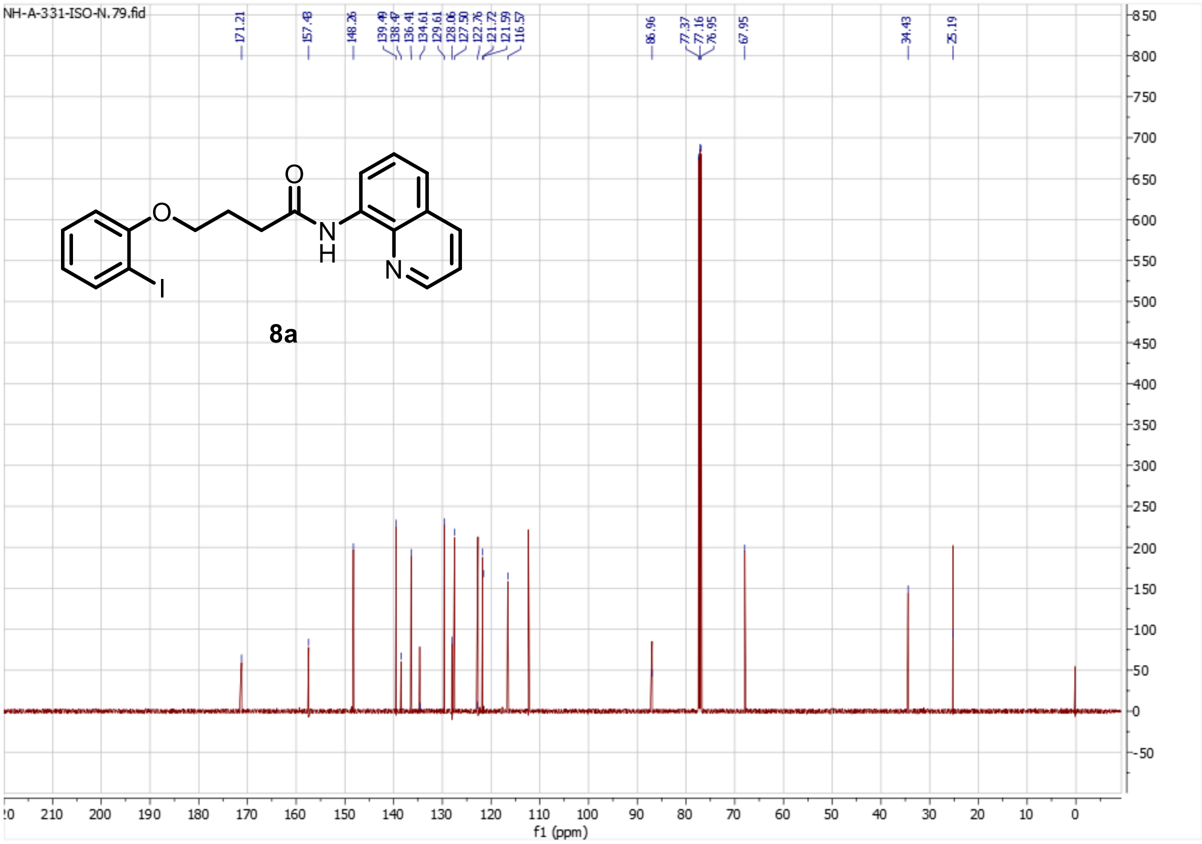

NH-A-331-ISO-N.79.fid
171.21
157.48
148.26
139.49
138.47
136.41
134.61
129.61
128.06
127.50
122.76
121.72
121.59
116.57
86.96
77.37
77.16
76.95
67.95
34.43
25.19
O
N
H
N
I
8a
f1 (ppm)

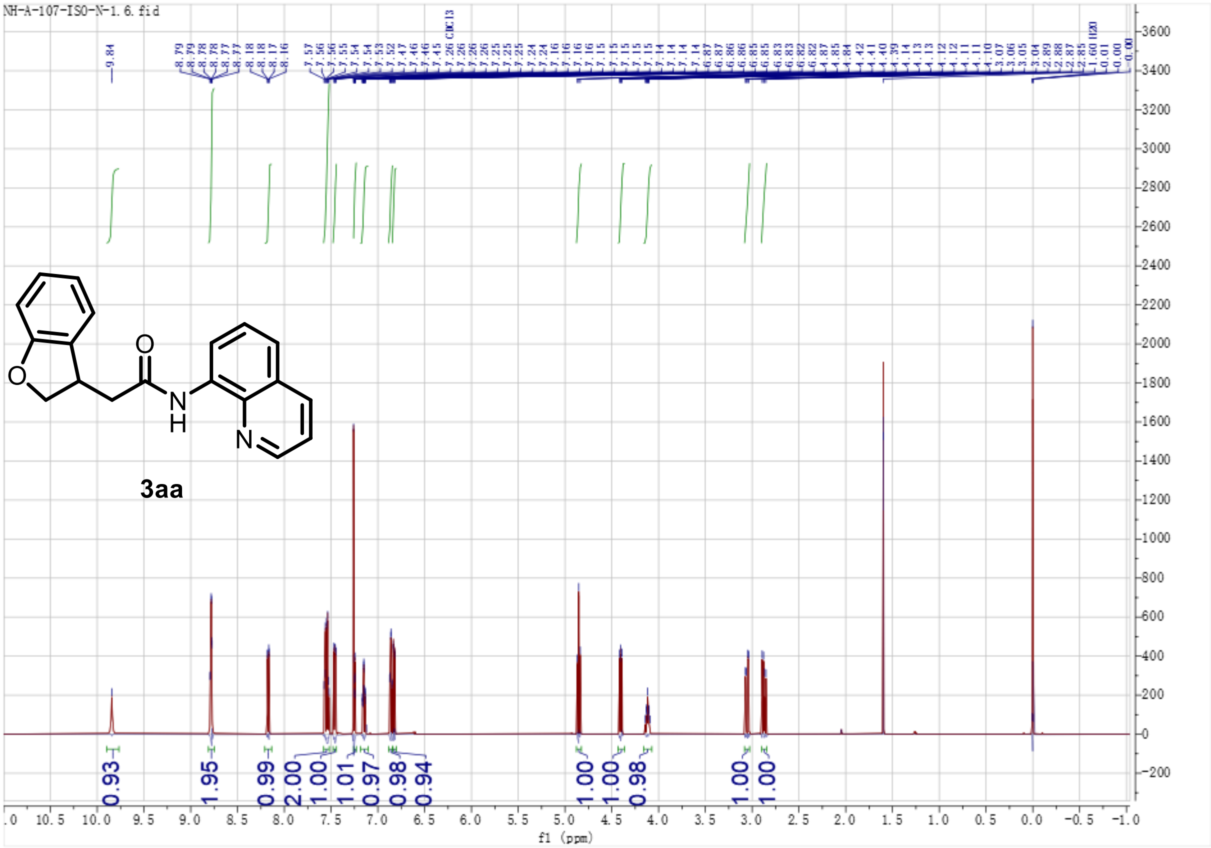

NH-A-107-ISO-N-1.6.fid
9.84
O
N
H
N
O
3aa
0.93
1.95
0.99
2.00
1.00
1.01
0.97
0.98
0.94
1.00
1.00
0.98
1.00
1.00
f1 (ppm)

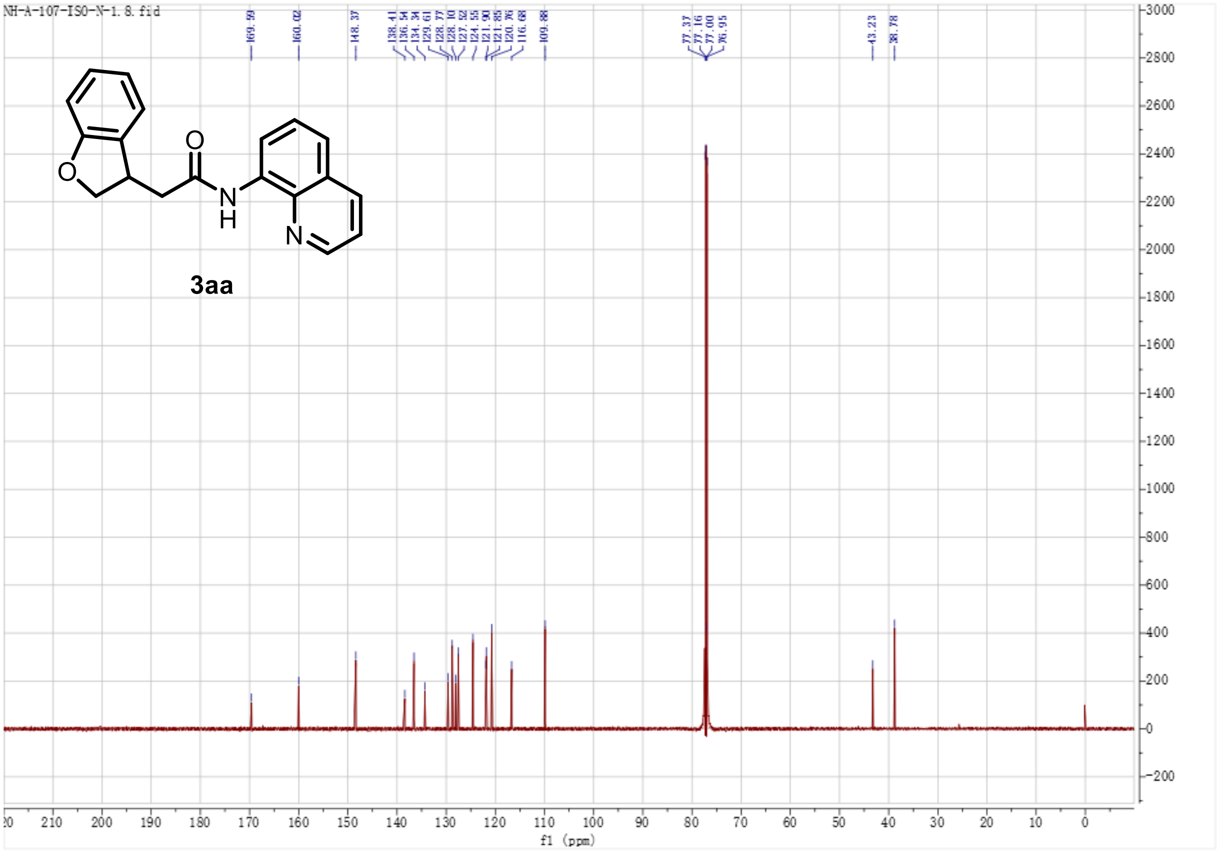
NH-A-107-ISO-N-1.8.fid
3aa
f1 (ppm)

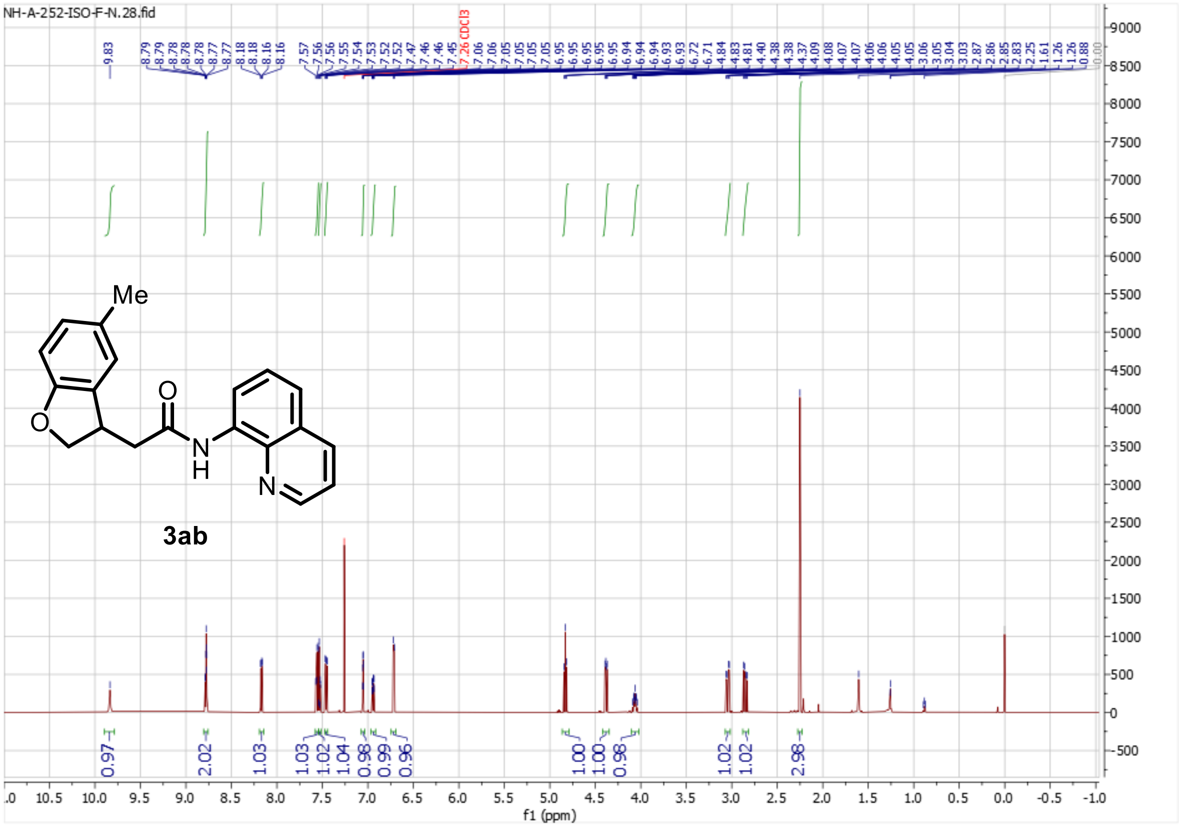
NH-A-252-ISO-F-N.28.fid
Me
3ab
f1 (ppm)

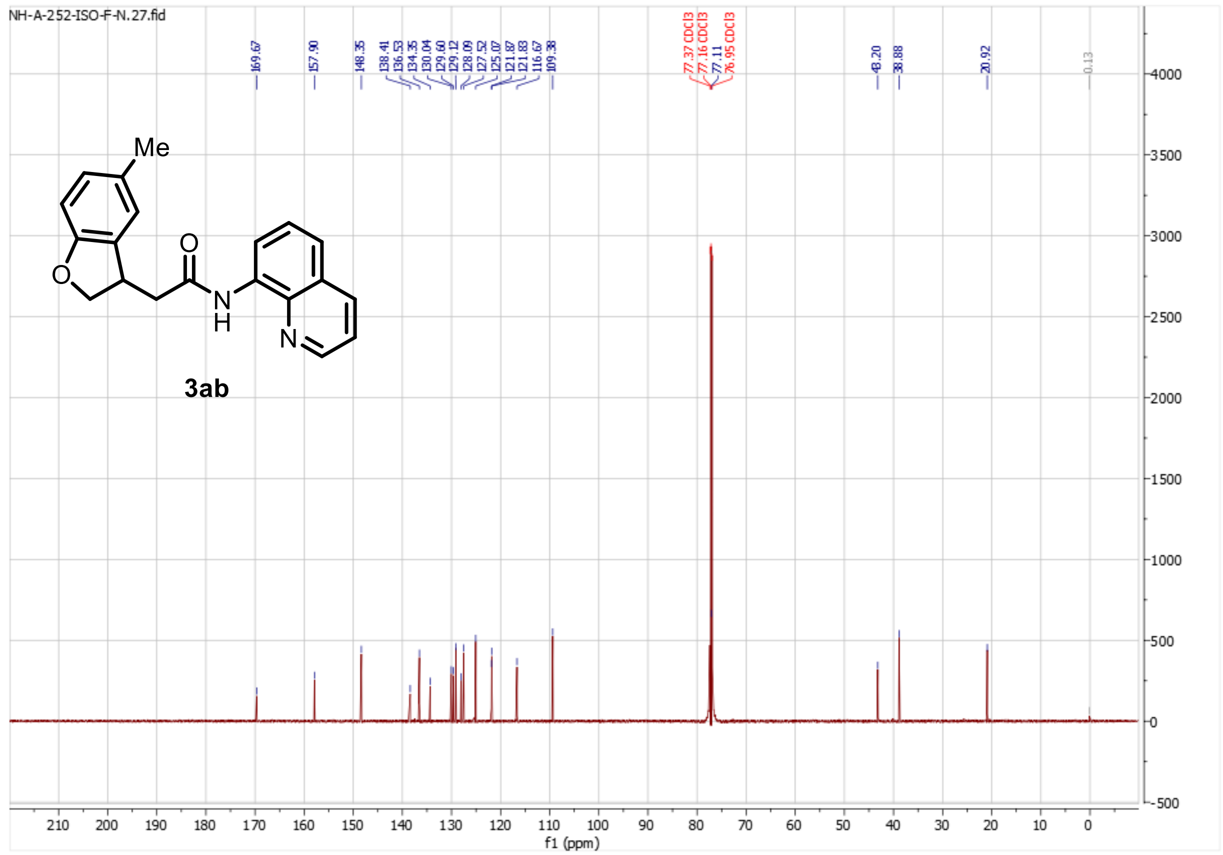
NH-A-252-ISO-F-N.27.fid
169.67
157.90
148.35
138.41
136.53
134.35
130.04
129.60
129.12
128.09
127.52
125.07
121.87
121.83
116.67
109.38
77.37 CDCl3
77.16 CDCl3
77.11
76.95 CDCl3
43.20
38.88
20.92
0.13
Me
O
O
N
H
N
3ab
f1 (ppm)

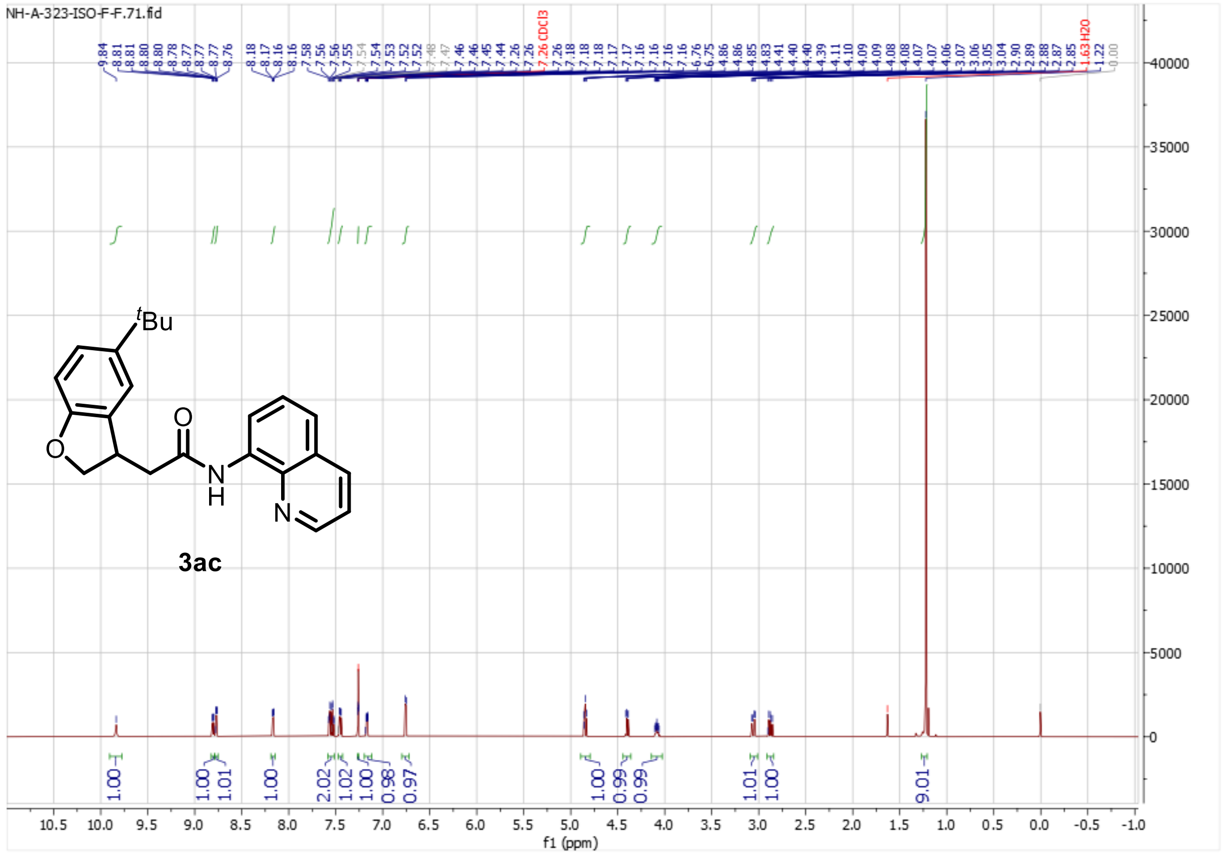
NH-A-323-ISO-F-F.71.fid
7.26 CDCl3
1.63 H2O
tBu
O
O
N
H
N
3ac
1.00
1.00
1.01
1.00
2.02
1.02
1.00
0.98
0.97
1.00
0.99
0.99
1.01
1.00
9.01
f1 (ppm)

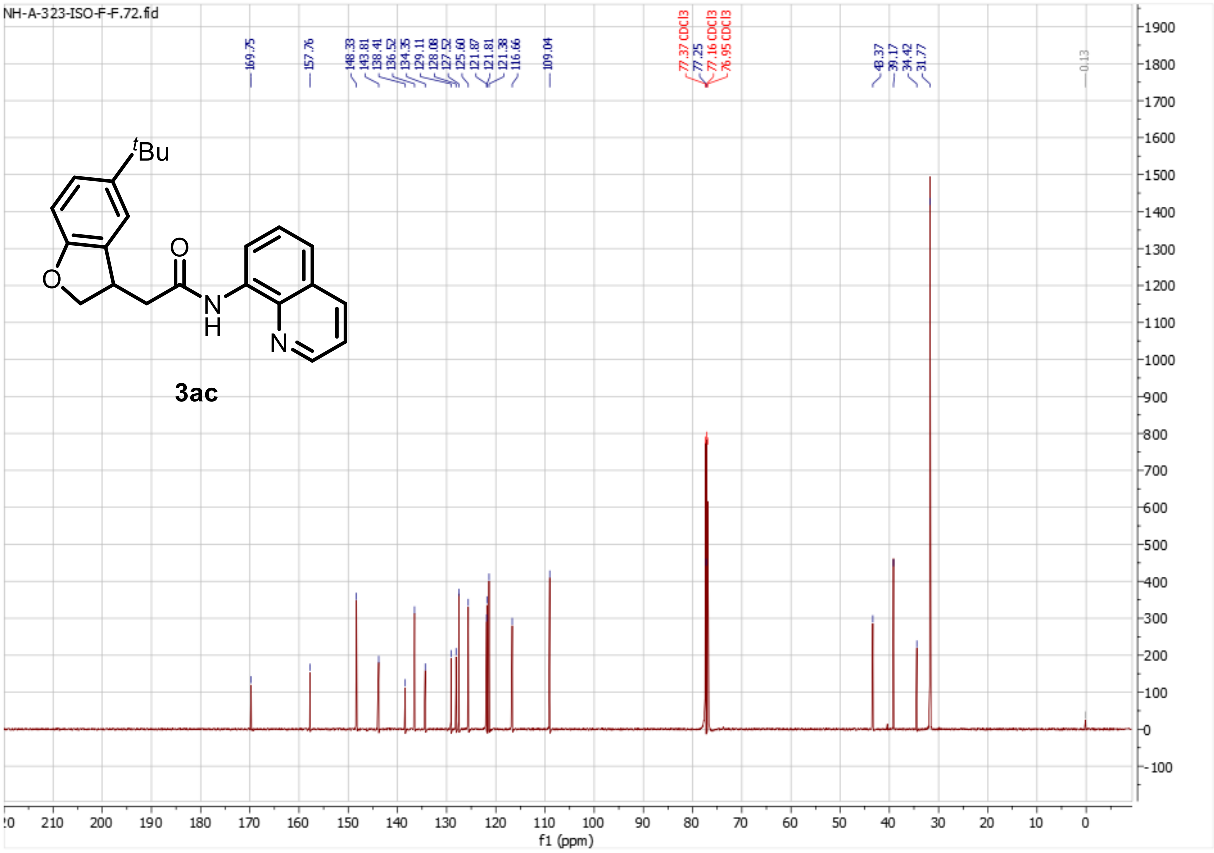
NH-A-323-ISO-F-F.72.fid
169.75
157.76
148.33
143.81
138.41
136.52
134.35
129.11
128.08
127.52
125.60
121.87
121.81
121.38
116.66
109.04
77.37 CDCl3
77.25
77.16 CDCl3
76.95 CDCl3
43.37
39.17
34.42
31.77
0.13
tBu
O
N
H
3ac
f1 (ppm)

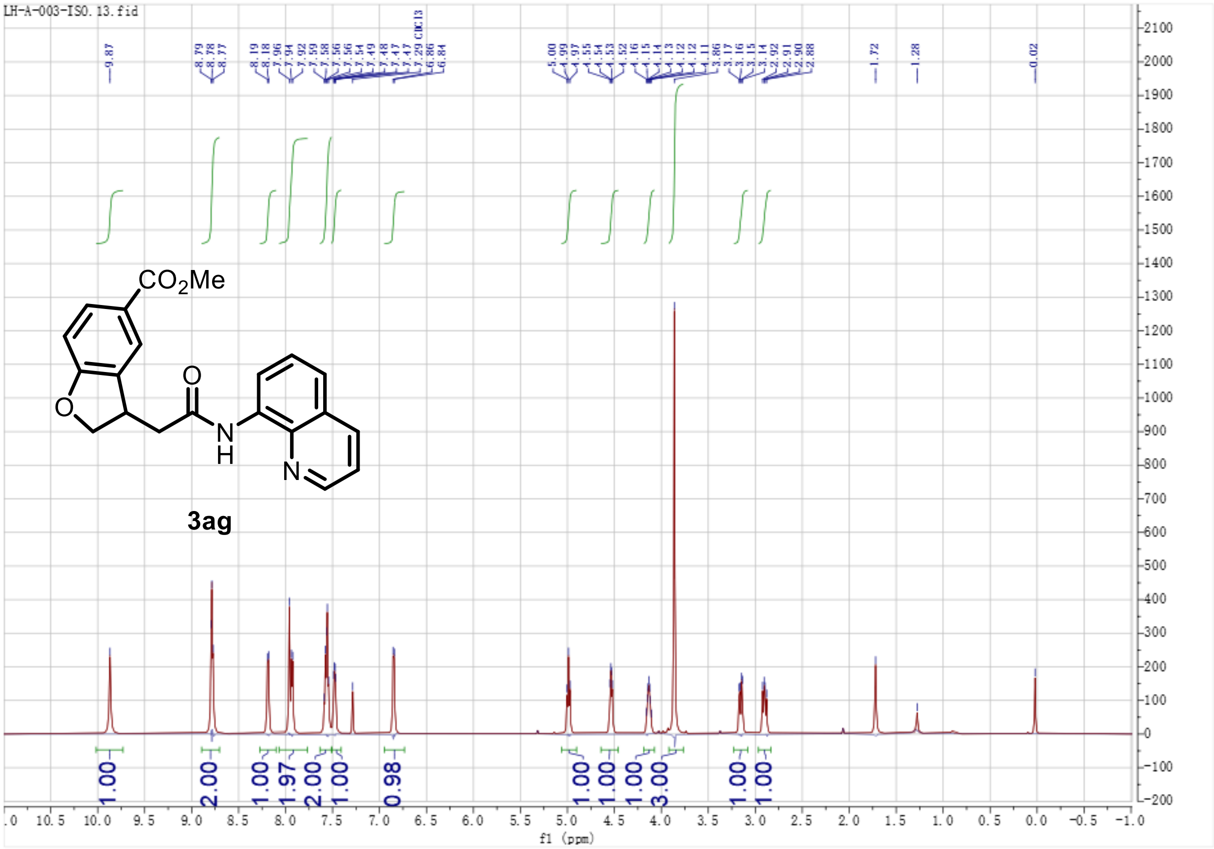
LH-A-003-ISO.13.fid
CO2Me
O
N
H
3ag
1.00
2.00
1.00
1.97
2.00
1.00
0.98
1.00
1.00
1.00
3.00
1.00
1.00
f1 (ppm)

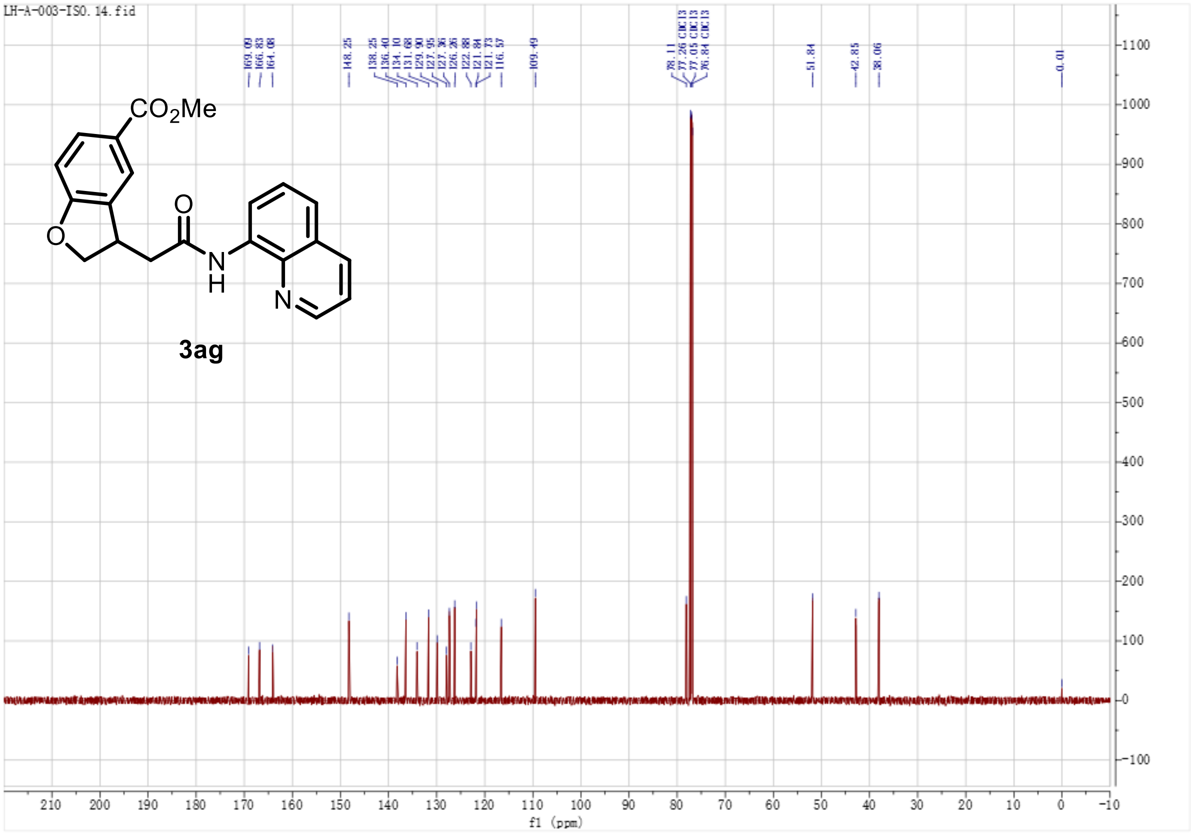
LH-A-003-ISO.14.fid
169.09
166.83
164.08
148.25
138.25
136.40
134.10
131.68
129.90
127.95
127.36
126.26
122.88
121.84
121.73
116.57
109.49
78.11
77.26 CDCl3
77.05 CDCl3
76.84 CDCl3
51.84
42.85
38.06
-0.01
CO2Me
O
N
H
N
3ag
f1 (ppm)

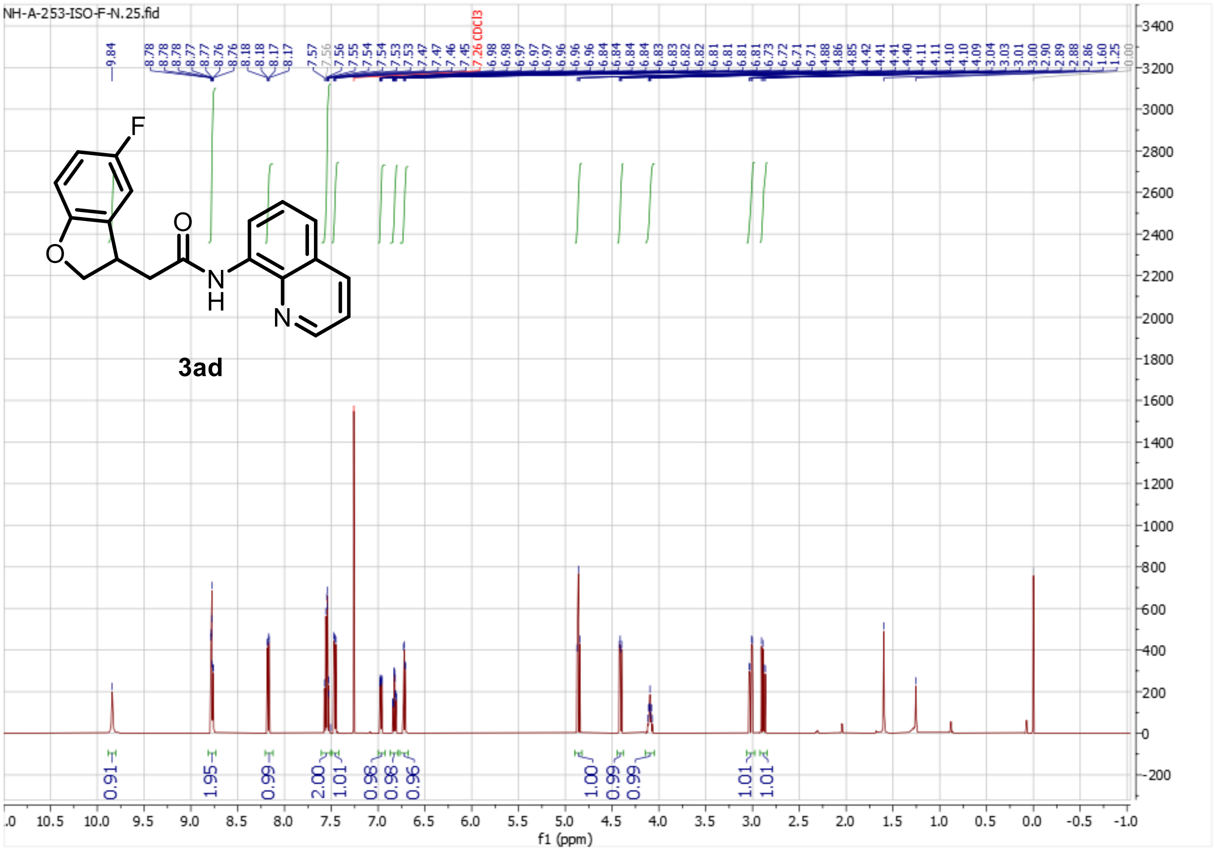
NH-A-253-ISO-F-N.25.fid
F
O
N
H
N
3ad
0.91
1.95
0.99
2.00
1.01
0.98
0.98
0.96
1.00
0.99
0.99
1.01
1.01
f1 (ppm)

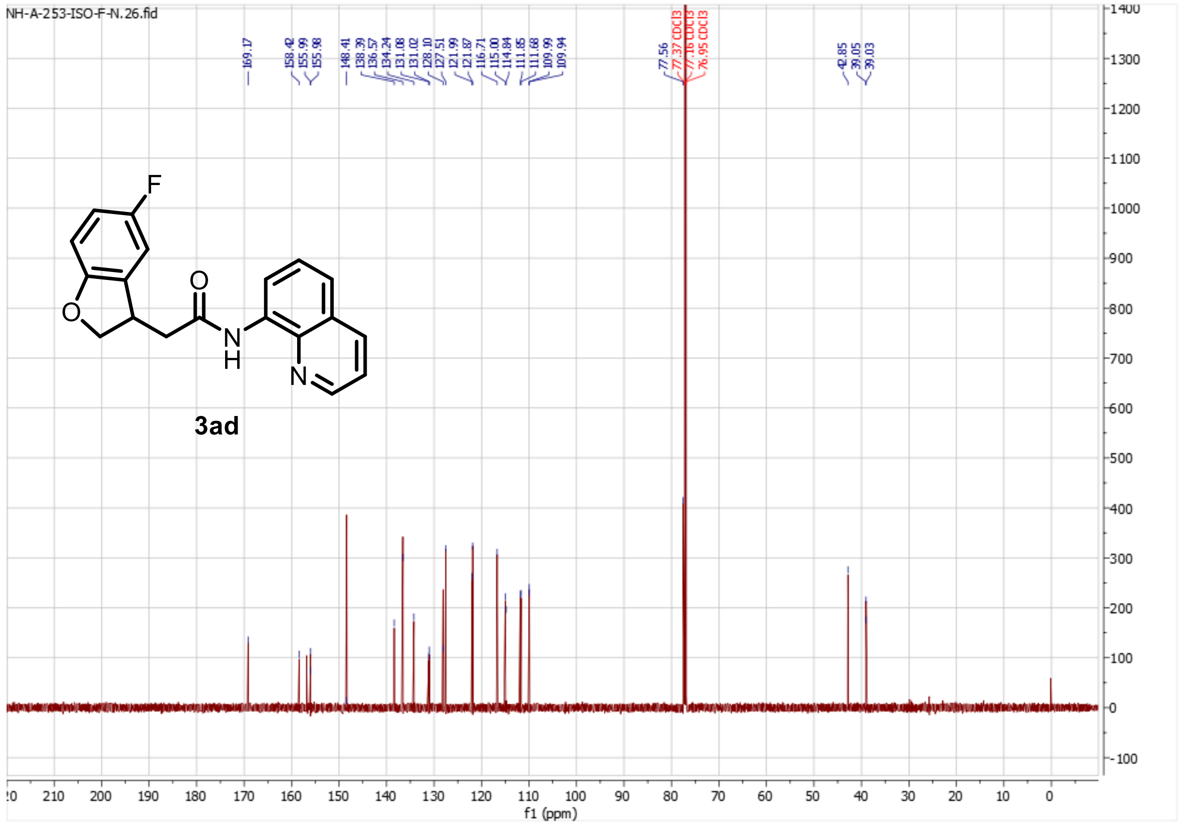
NH-A-253-ISO-F-N.26.fid
169.17
158.42
155.99
155.98
148.41
138.39
136.57
134.24
131.08
131.02
128.10
127.51
121.99
121.87
116.71
115.00
114.84
111.85
111.68
109.99
109.94
77.56
77.37 CDCl3
77.16 CDCl3
76.95 CDCl3
42.85
39.05
39.03
F
O
O
N
H
N
3ad
f1 (ppm)

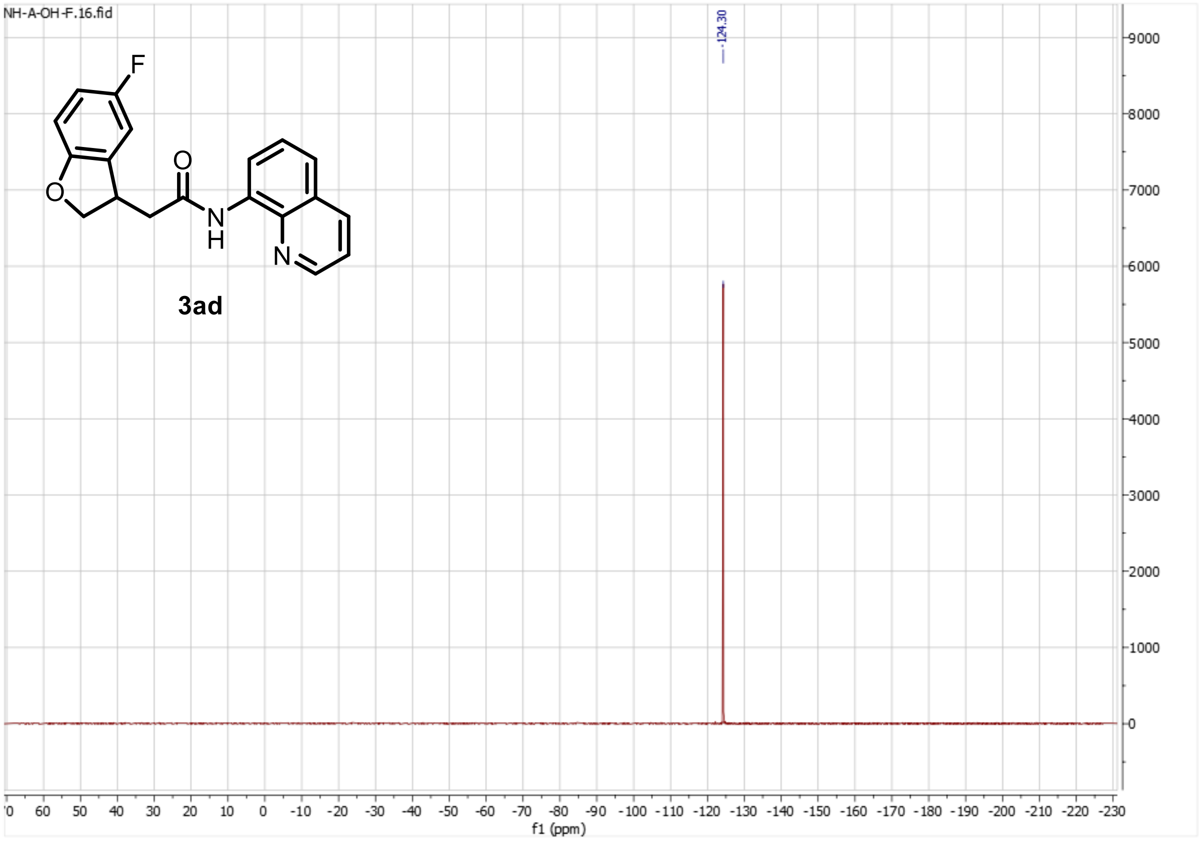
NH-A-OH-F.16.fid
-124.30
F
O
O
N
H
N
3ad
f1 (ppm)

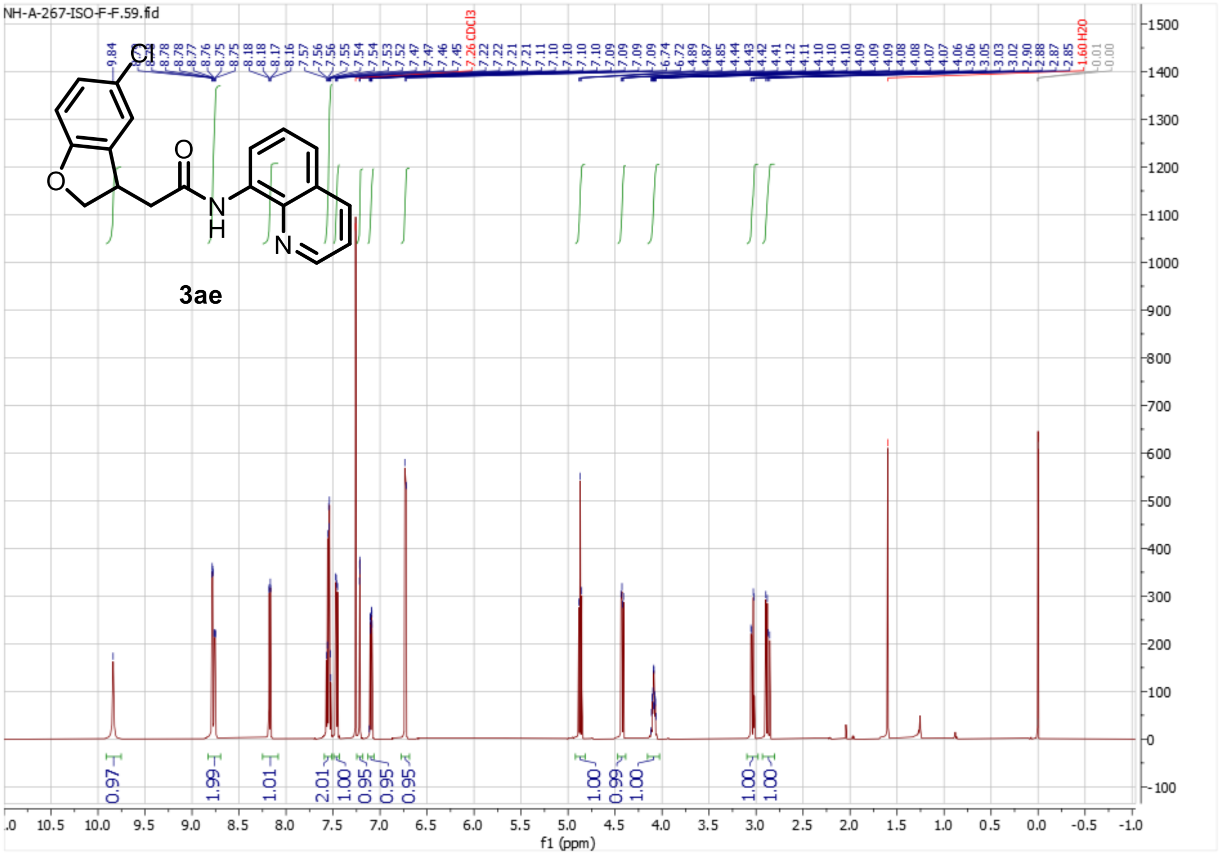
NH-A-267-ISO-F-F.59.fid
3ae
f1 (ppm)

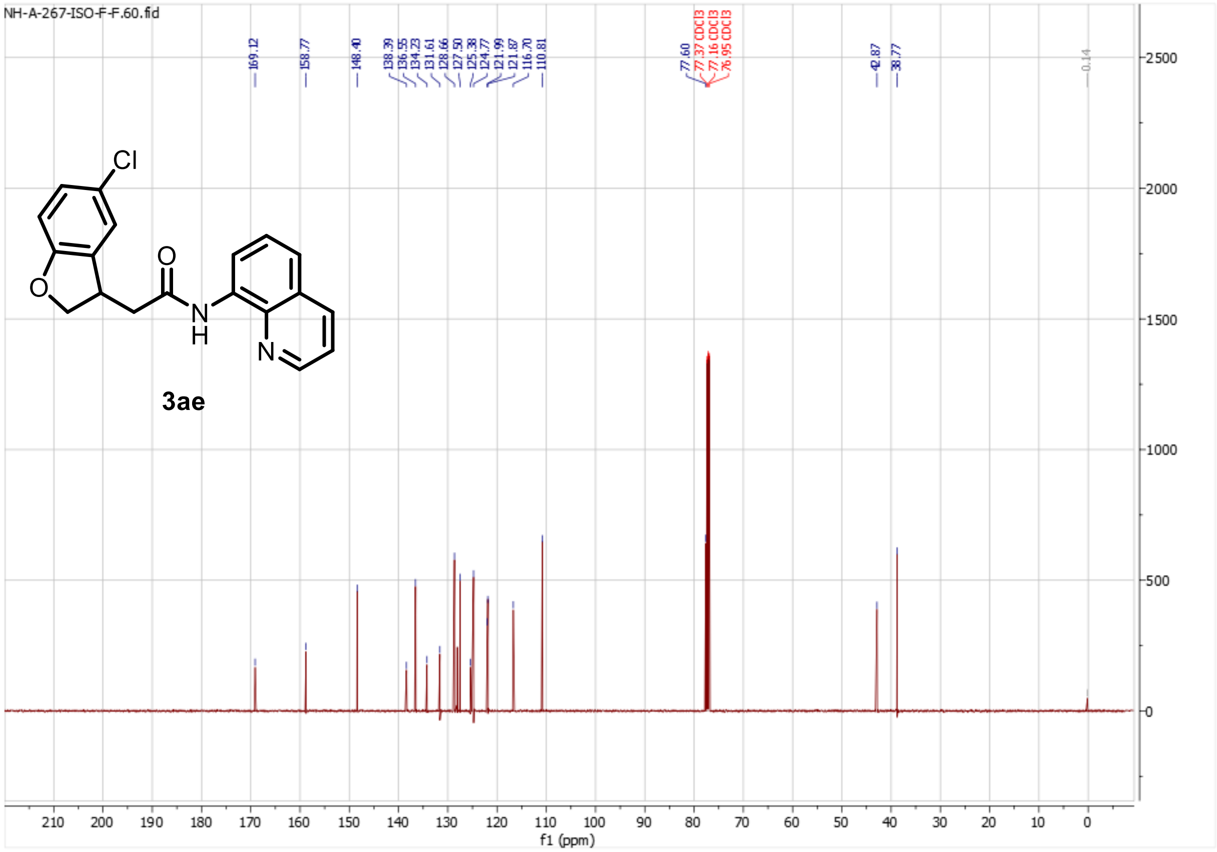
NH-A-267-ISO-F-F.60.fid
169.12
158.77
148.40
110.81
42.87
38.77
3ae
f1 (ppm)

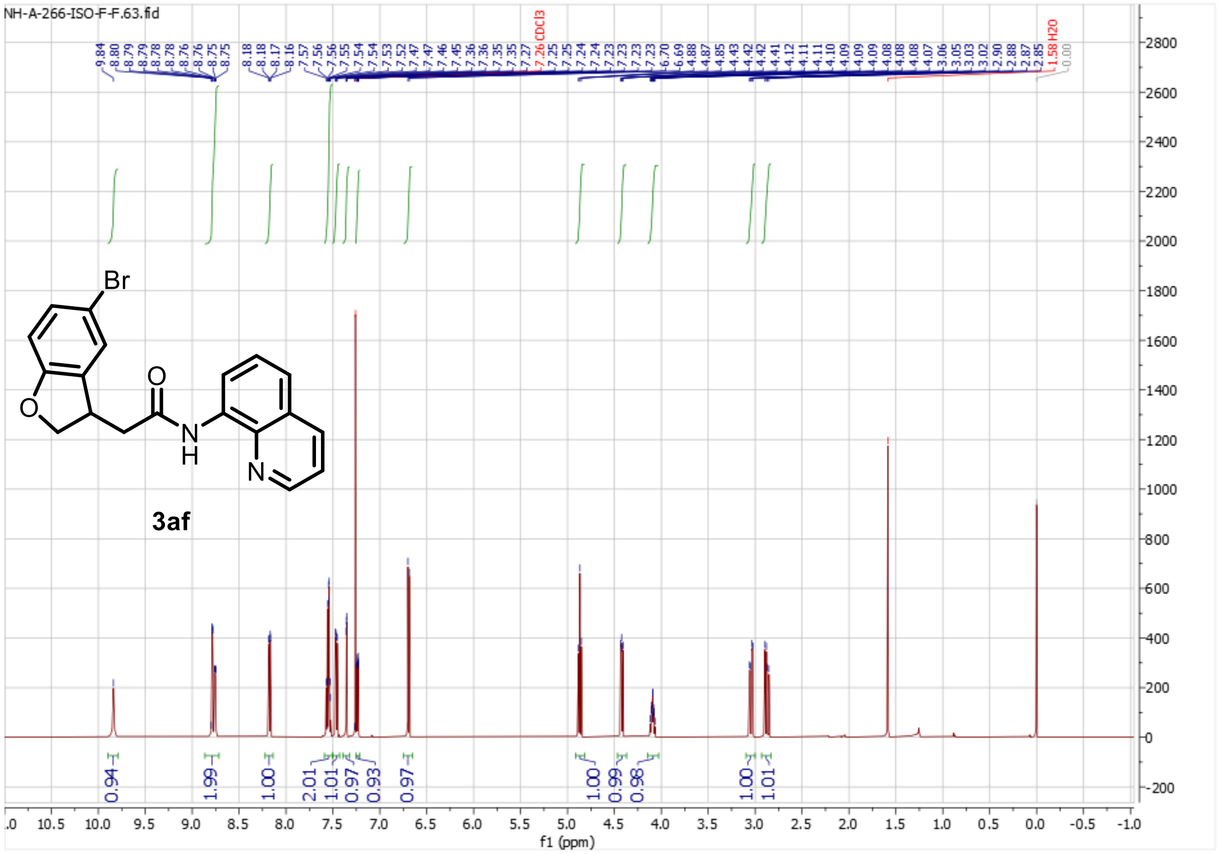
NH-A-266-ISO-F-F.63.fid
7.26 CDCl3
1.58 H2O
Br
O
N
H
N
3af
f1 (ppm)

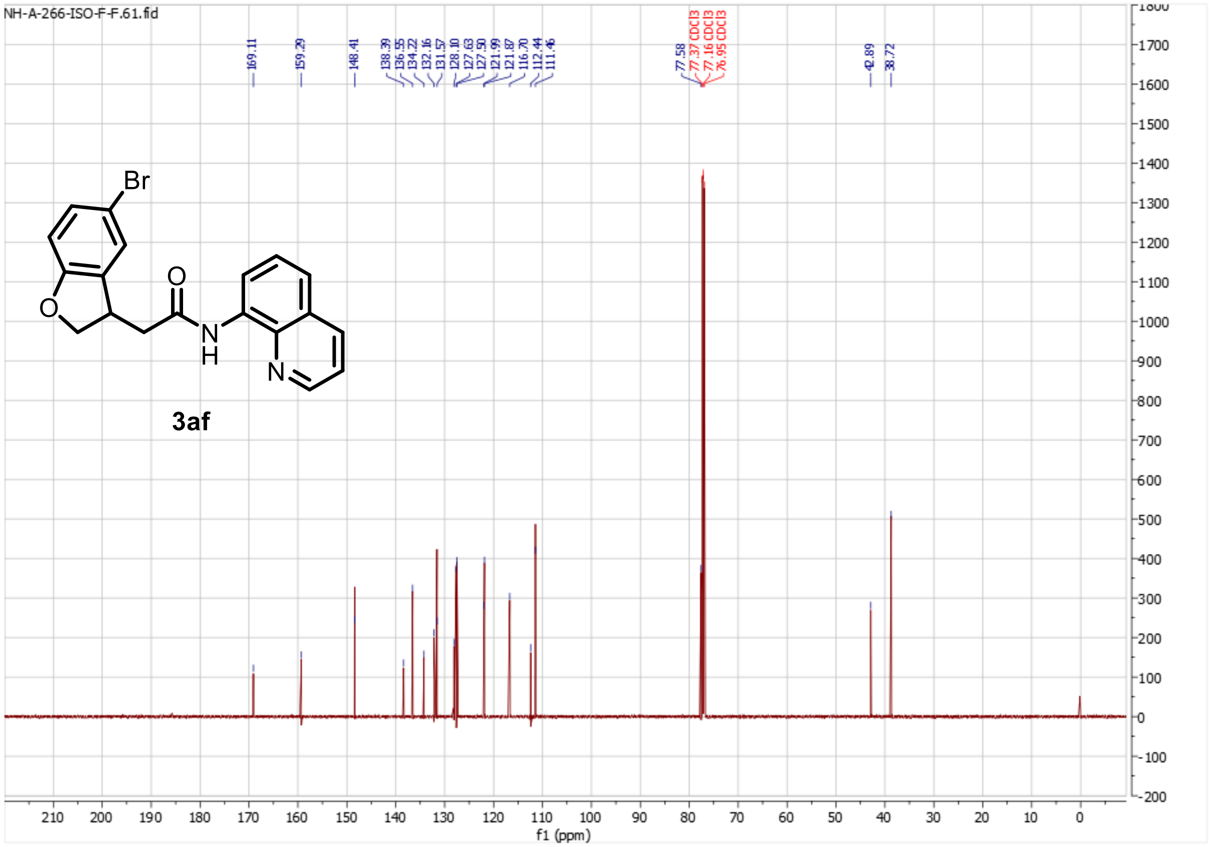
NH-A-266-ISO-F-F.61.fid
Br
O
N
H
N
3af
f1 (ppm)

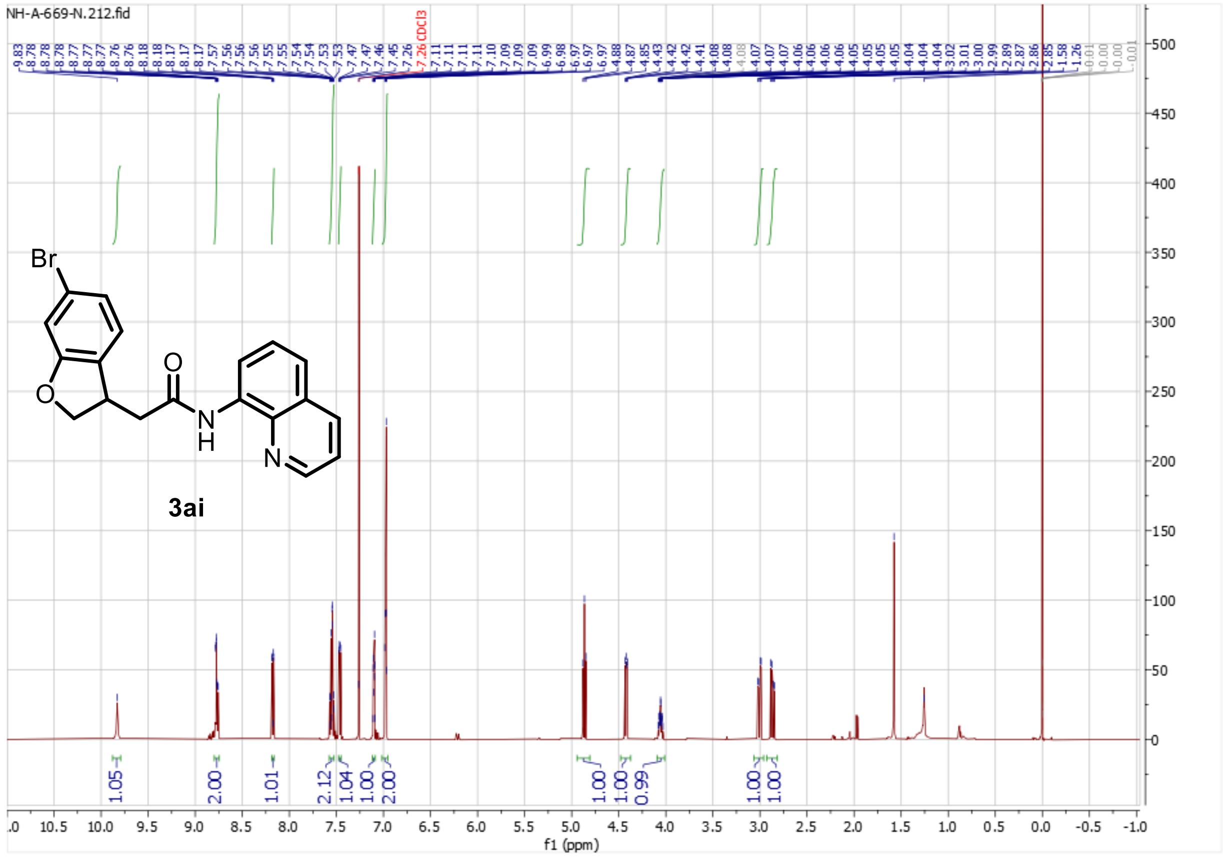
NH-A-669-N.212.fid
Br
O
N
H
N
3ai
f1 (ppm)

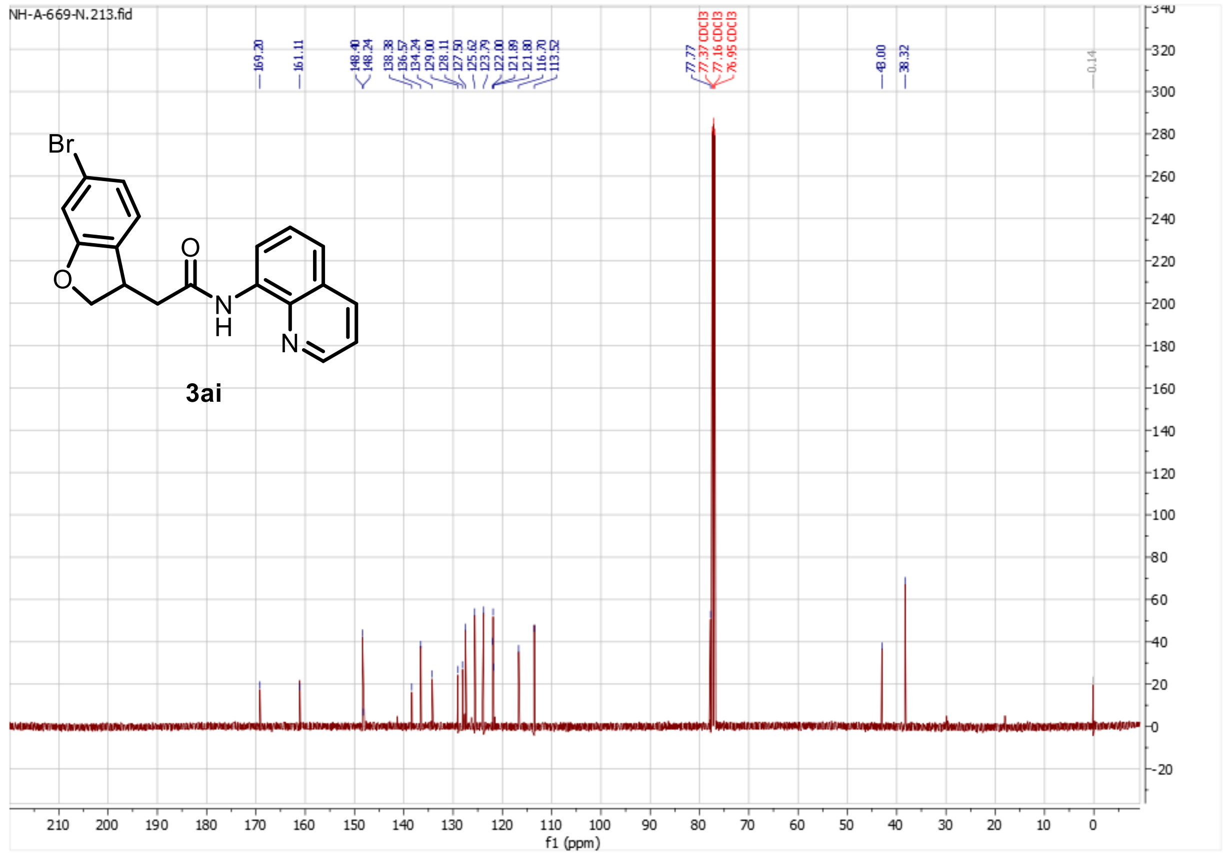
NH-A-669-N.213.fid
169.20
161.11
148.40
148.24
138.38
136.57
134.24
129.00
128.11
127.50
125.62
123.79
122.00
121.89
121.80
116.70
113.52
77.37 CDCl3
77.16 CDCl3
76.95 CDCl3
43.00
38.32
Br
O
N
H
N
3ai
f1 (ppm)

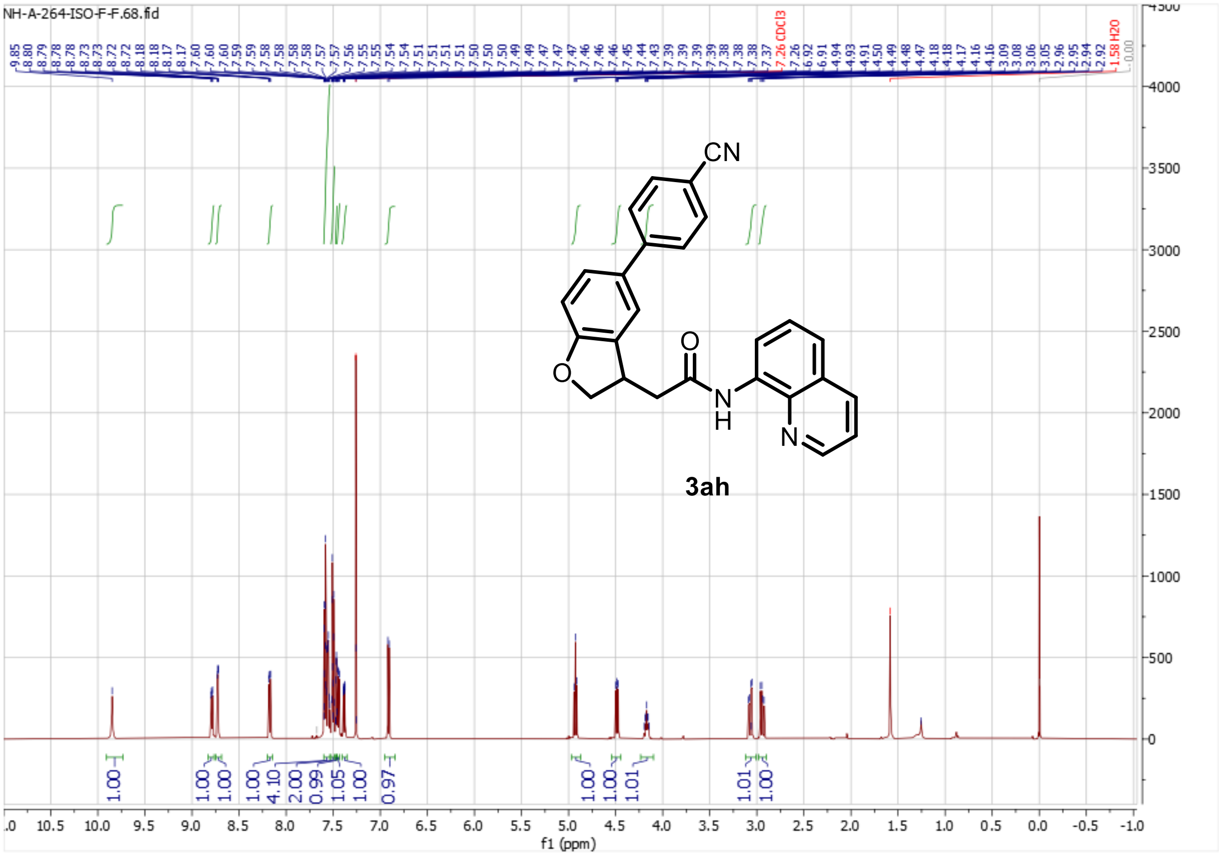
NH-A-264-ISO-F-F.68.fid
CN
O
N
H
N
O
3ah
f1 (ppm)

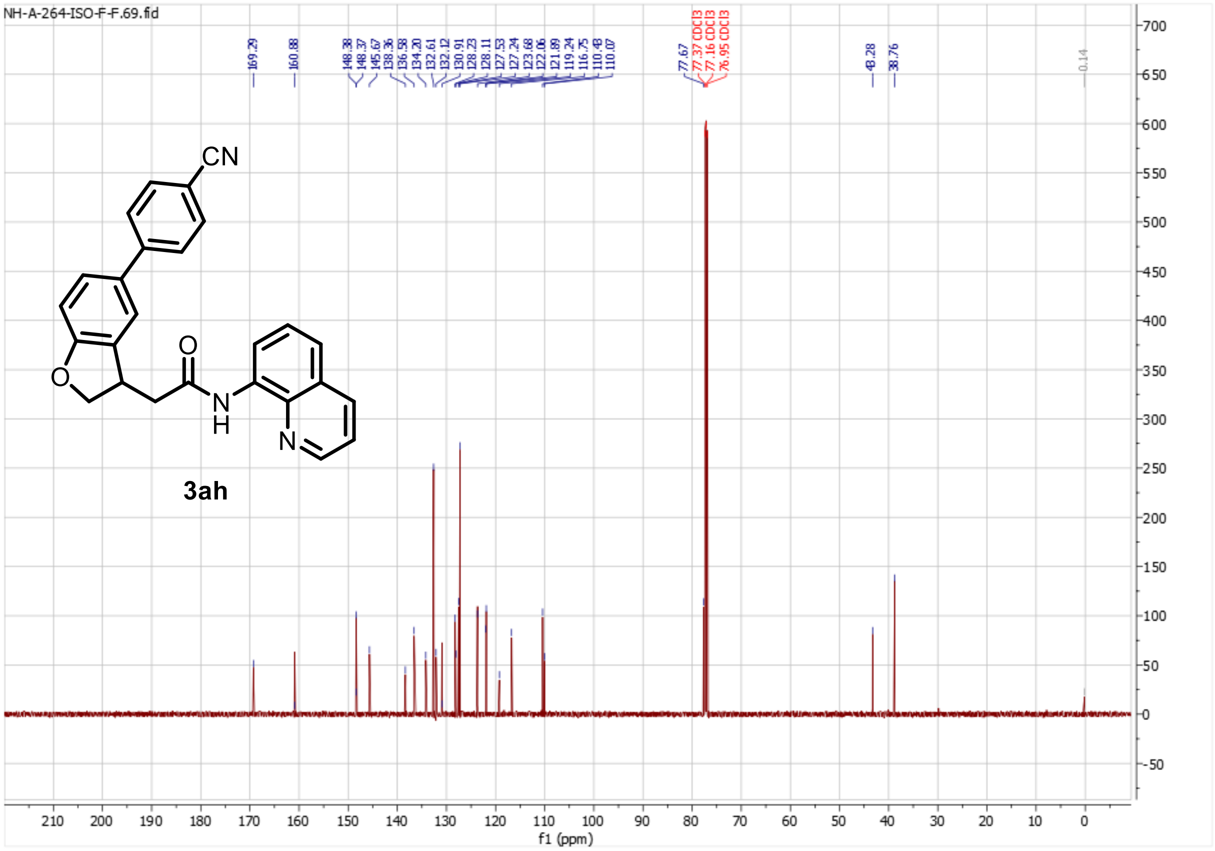
NH-A-264-ISO-F-F.69.fid
CN
O
N
H
N
O
3ah
f1 (ppm)

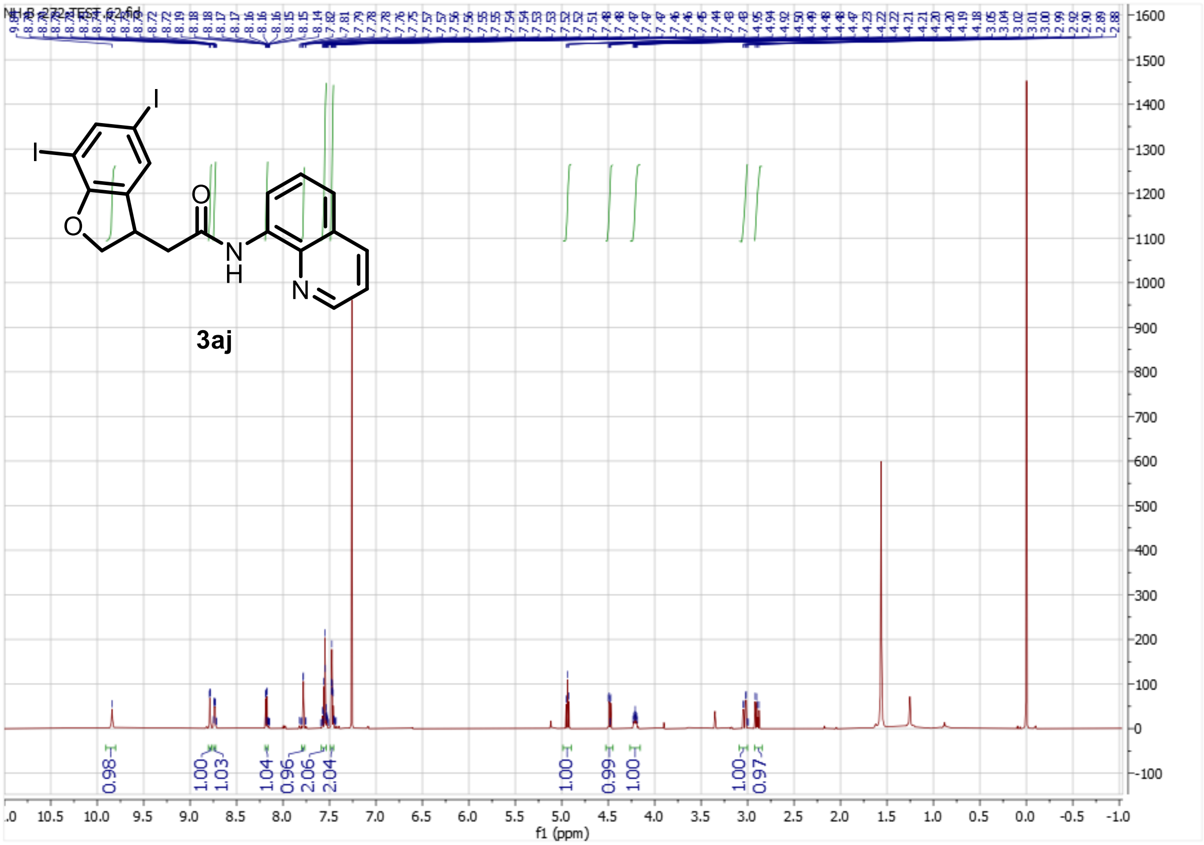
NH-B-272-TEST.62.fid
3aj
f1 (ppm)

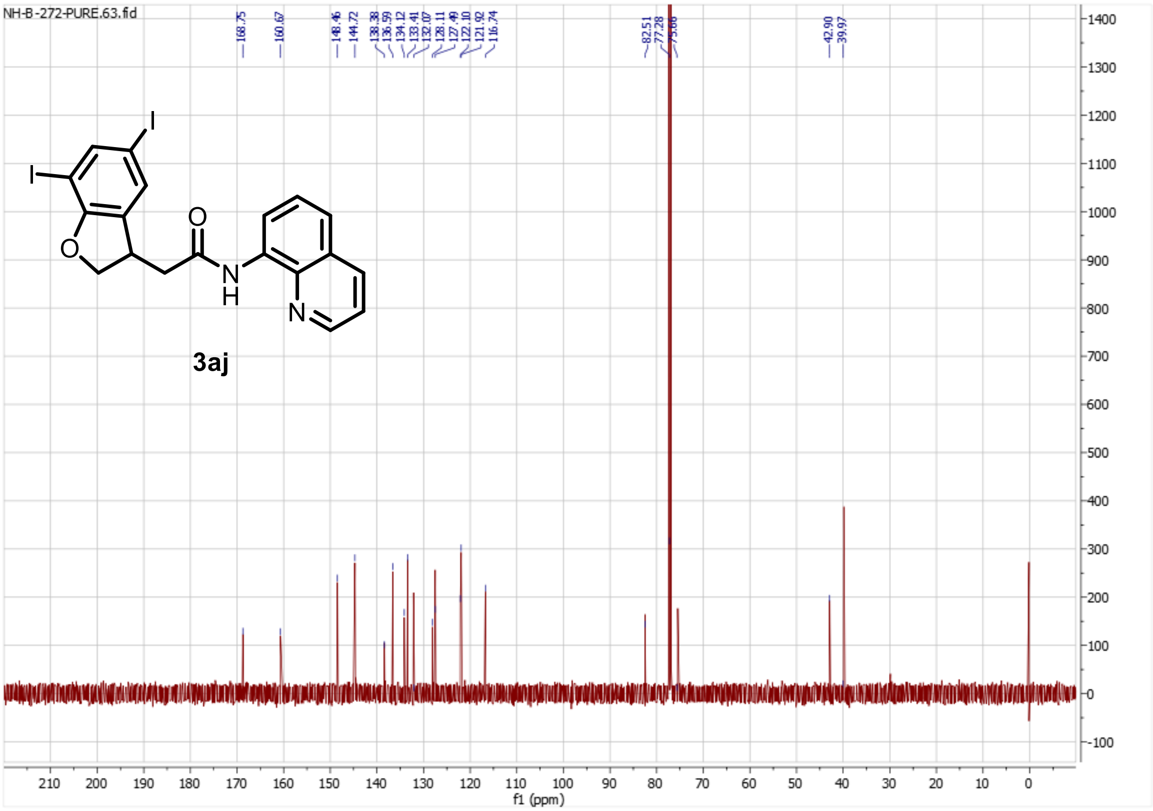
NH-B-272-PURE.63.fid
168.75
160.67
148.46
144.72
82.51
77.28
42.90
39.97
3aj
f1 (ppm)

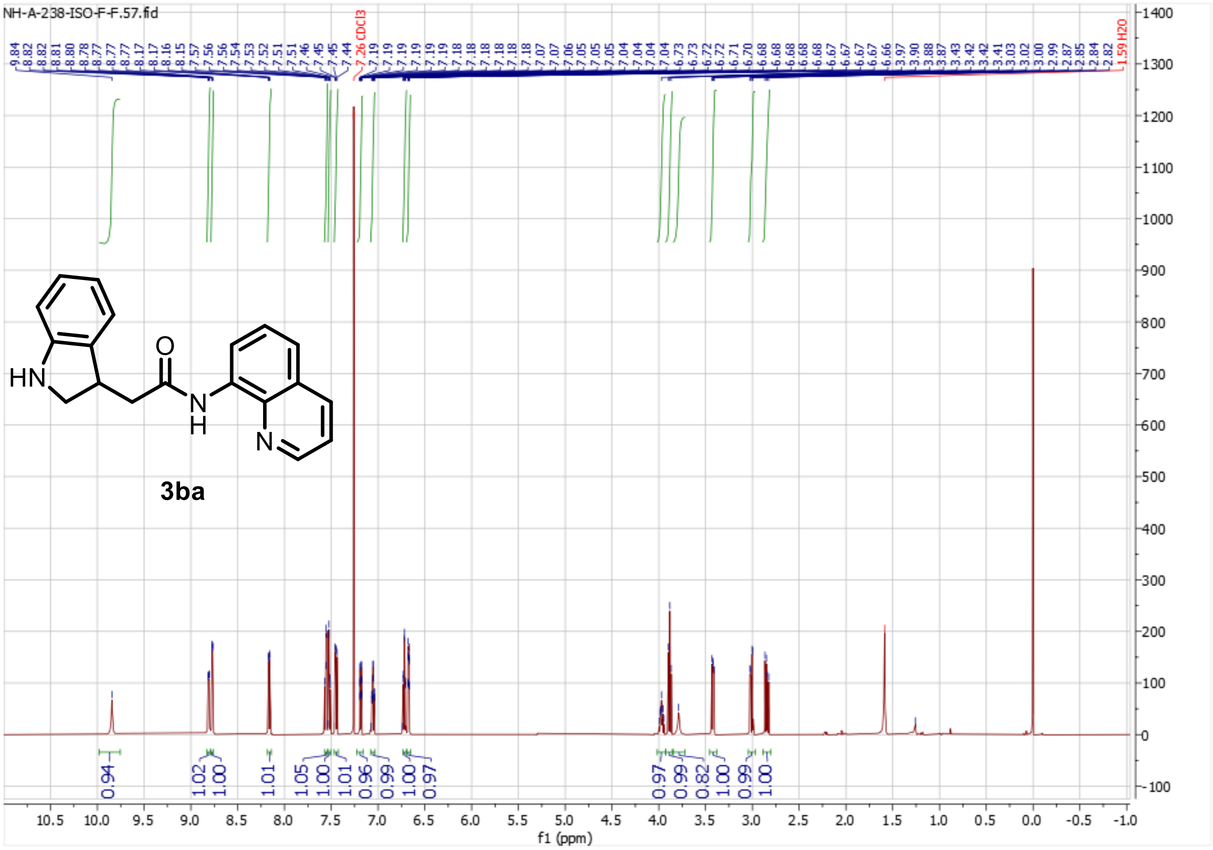
NH-A-238-ISO-F-F.57.fid
7.26 CDCl3
1.59 H2O
3ba
f1 (ppm)

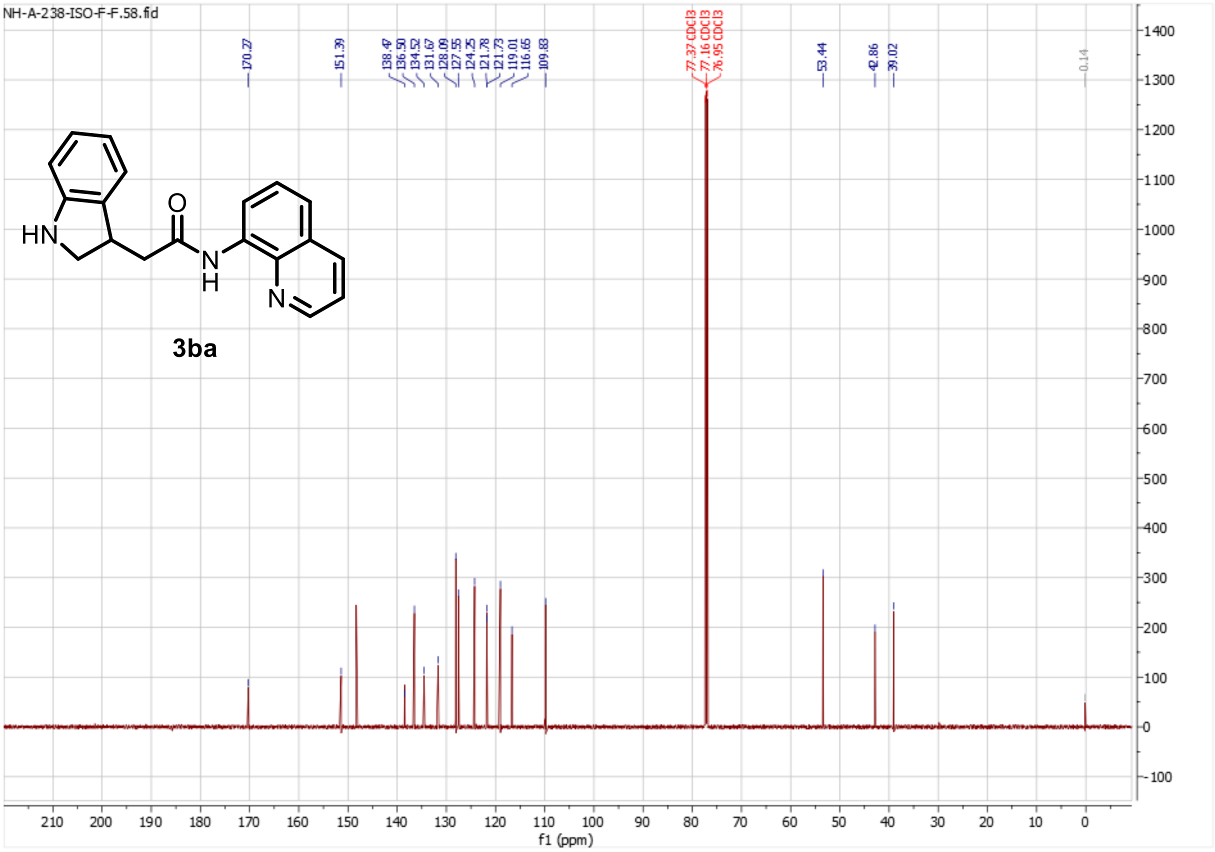
NH-A-238-ISO-F-F.58.fid
170.27
151.39
77.37 CDCl3
77.16 CDCl3
76.95 CDCl3
53.44
3ba
f1 (ppm)

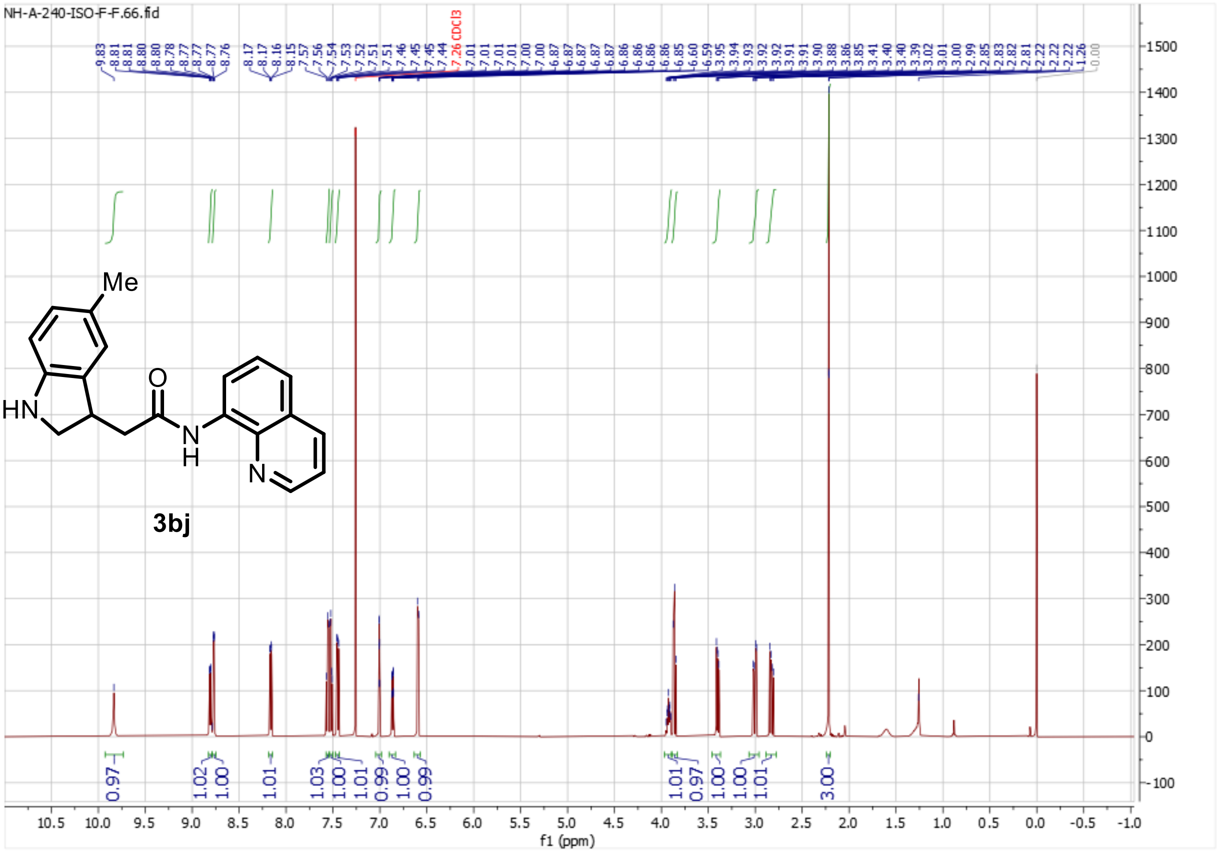
NH-A-240-ISO-F-F.66.fid
3bj
f1 (ppm)

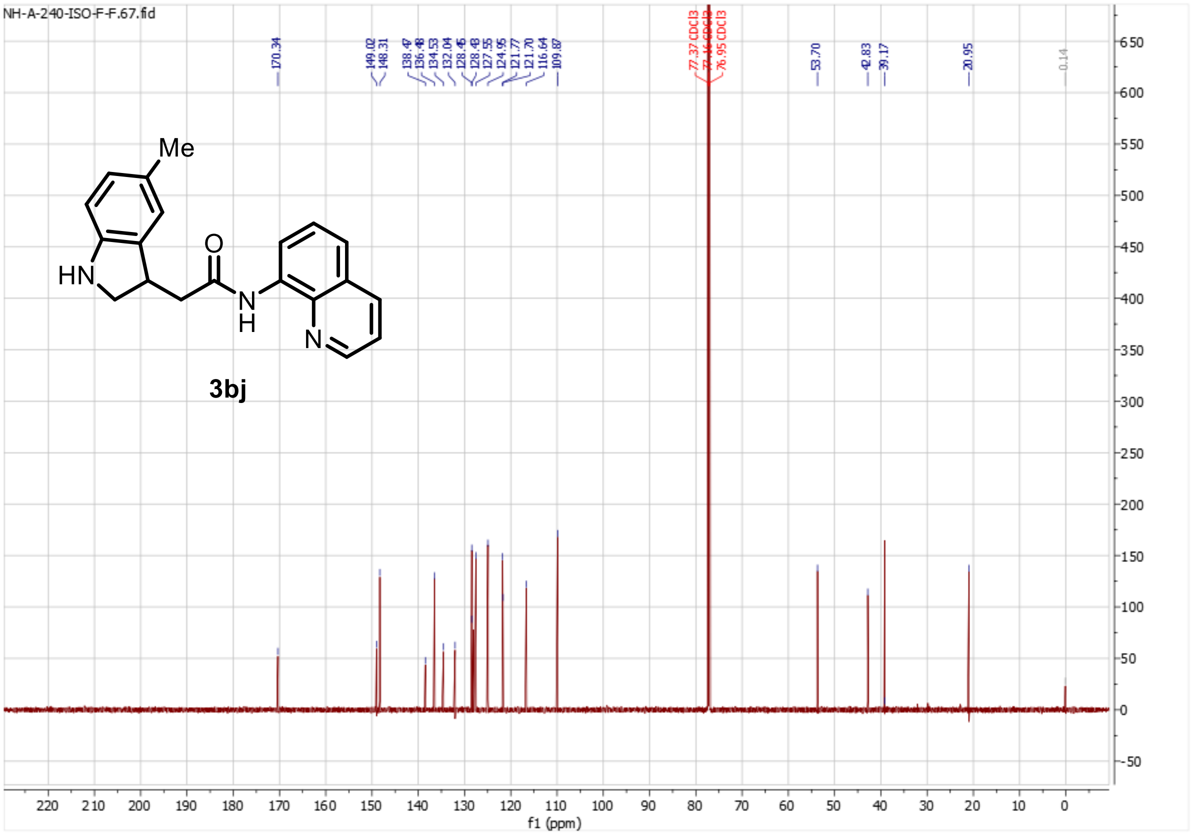
NH-A-240-ISO-F-F.67.fid
170.34
149.02
148.31
138.47
136.48
134.53
132.04
128.45
128.43
127.55
124.95
121.77
121.70
116.64
109.87
77.37 CDCl3
76.95 CDCl3
53.70
42.83
39.17
20.95
3bj
f1 (ppm)

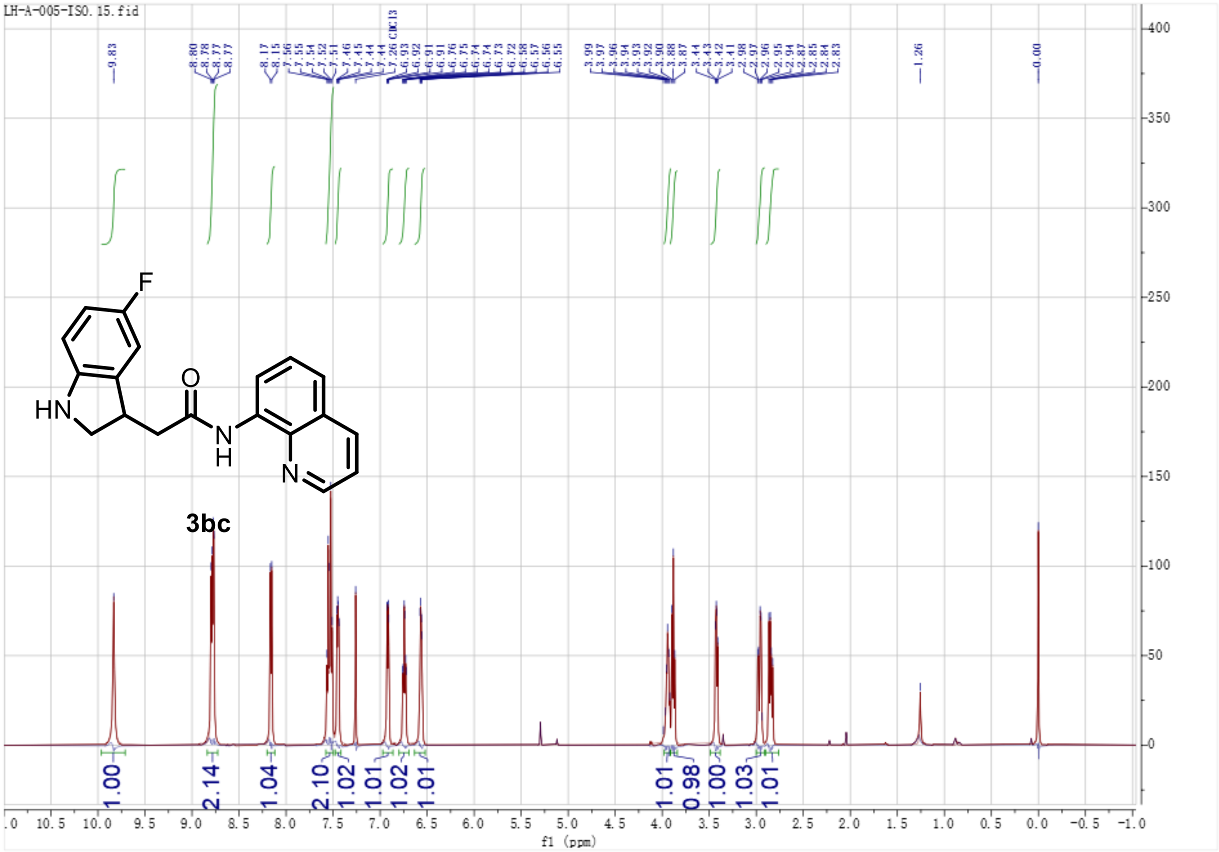
LH-A-005-ISO.15.fid
3bc
f1 (ppm)

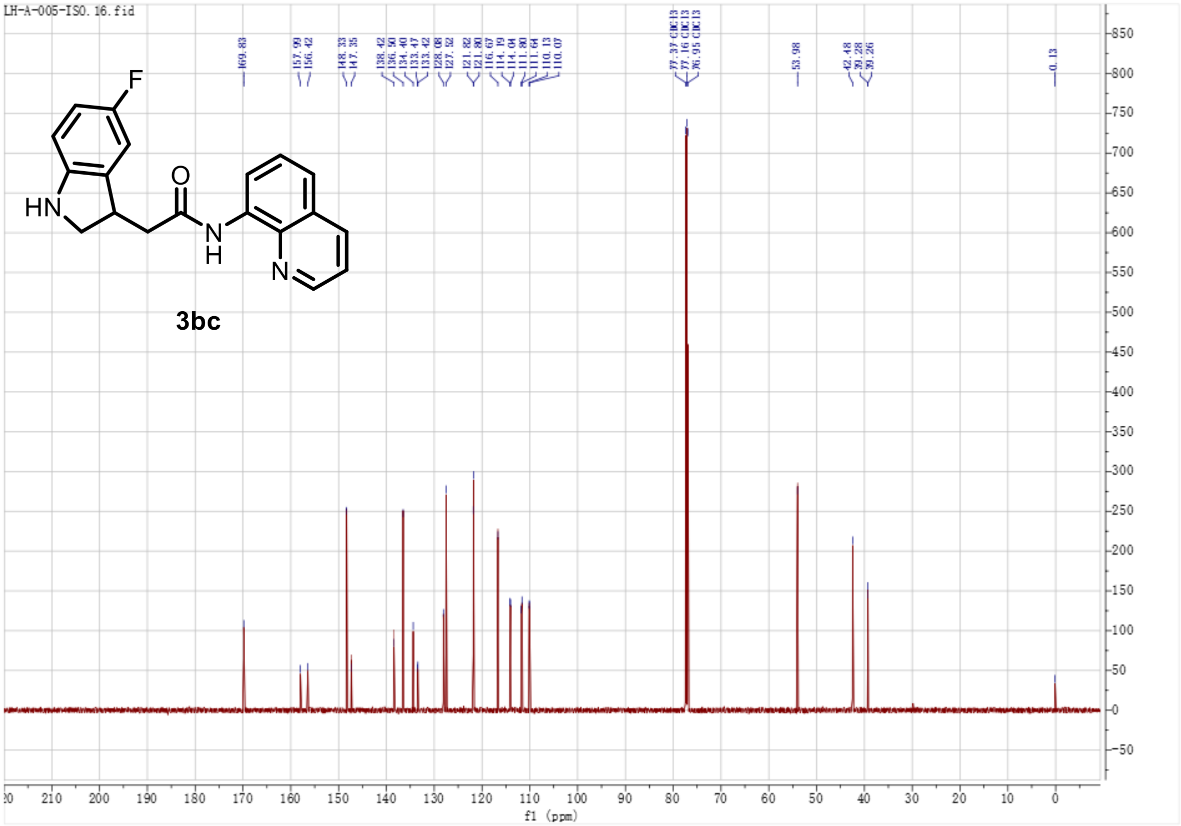
LH-A-005-ISO.16.fid
3bc
f1 (ppm)

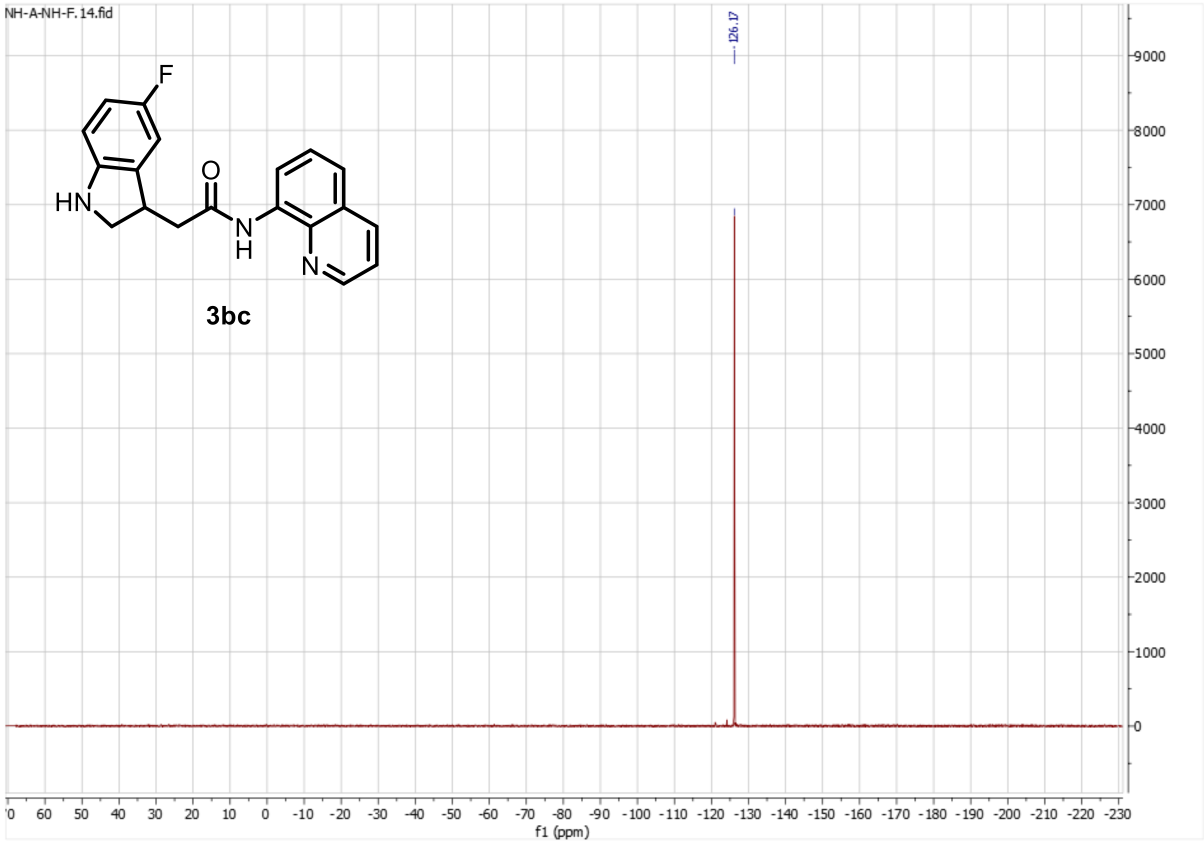
NH-A-NH-F.14.fid
126.17
f1 (ppm)

3bc

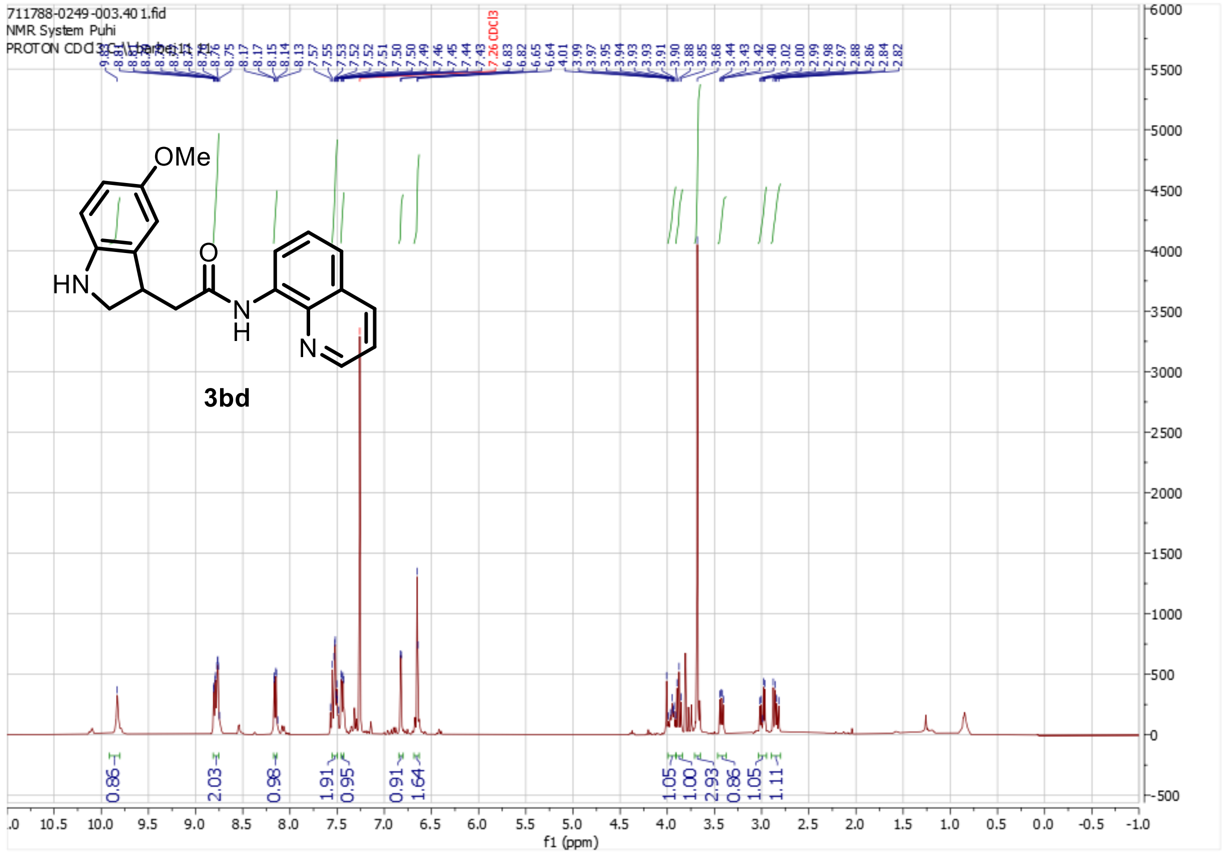
711788-0249-003.401.fid
NMR System Puhi
PROTON CDCl3
7.26 CDCl3
f1 (ppm)

3bd

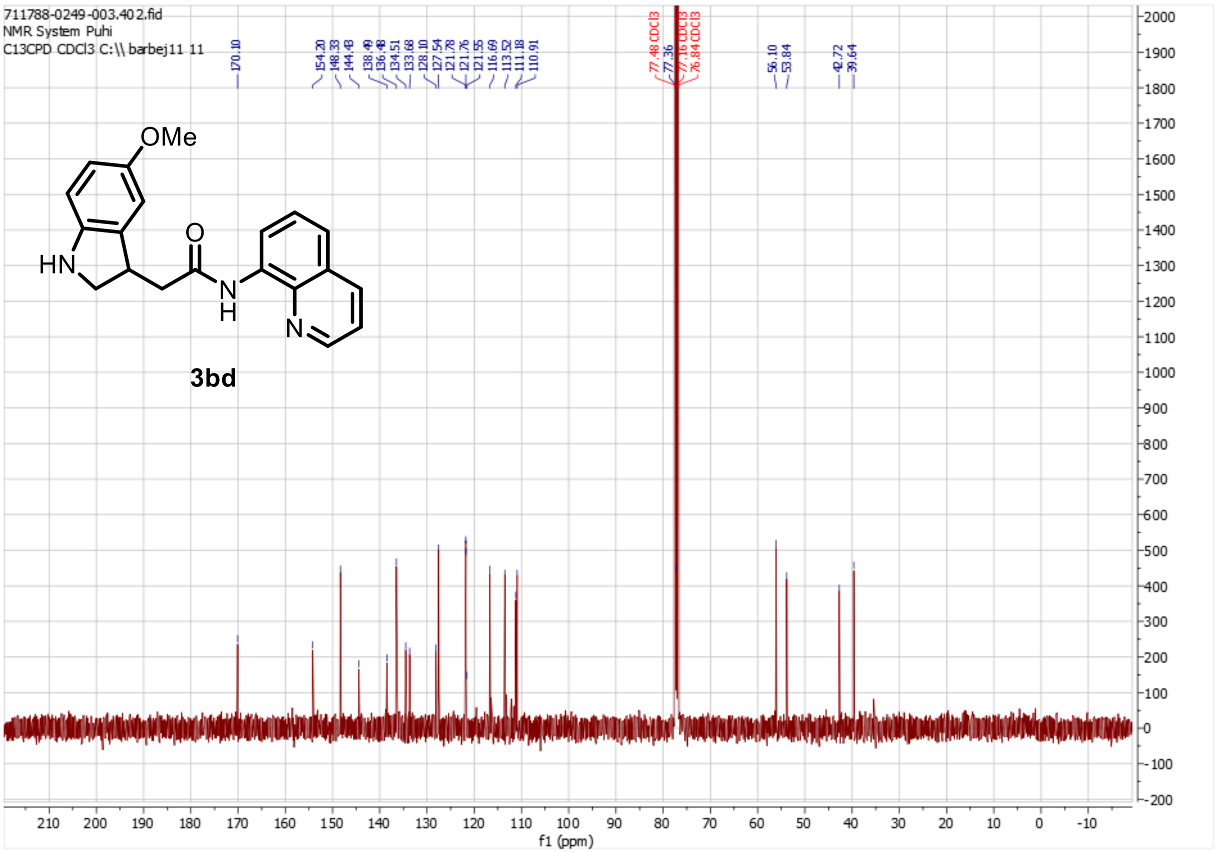
711788-0249-003.402.fid
NMR System Puhi
C13CPD CDCl3 C:\\ barbej11 11
OMe
HN
O
N
H
N
f1 (ppm)

3bd

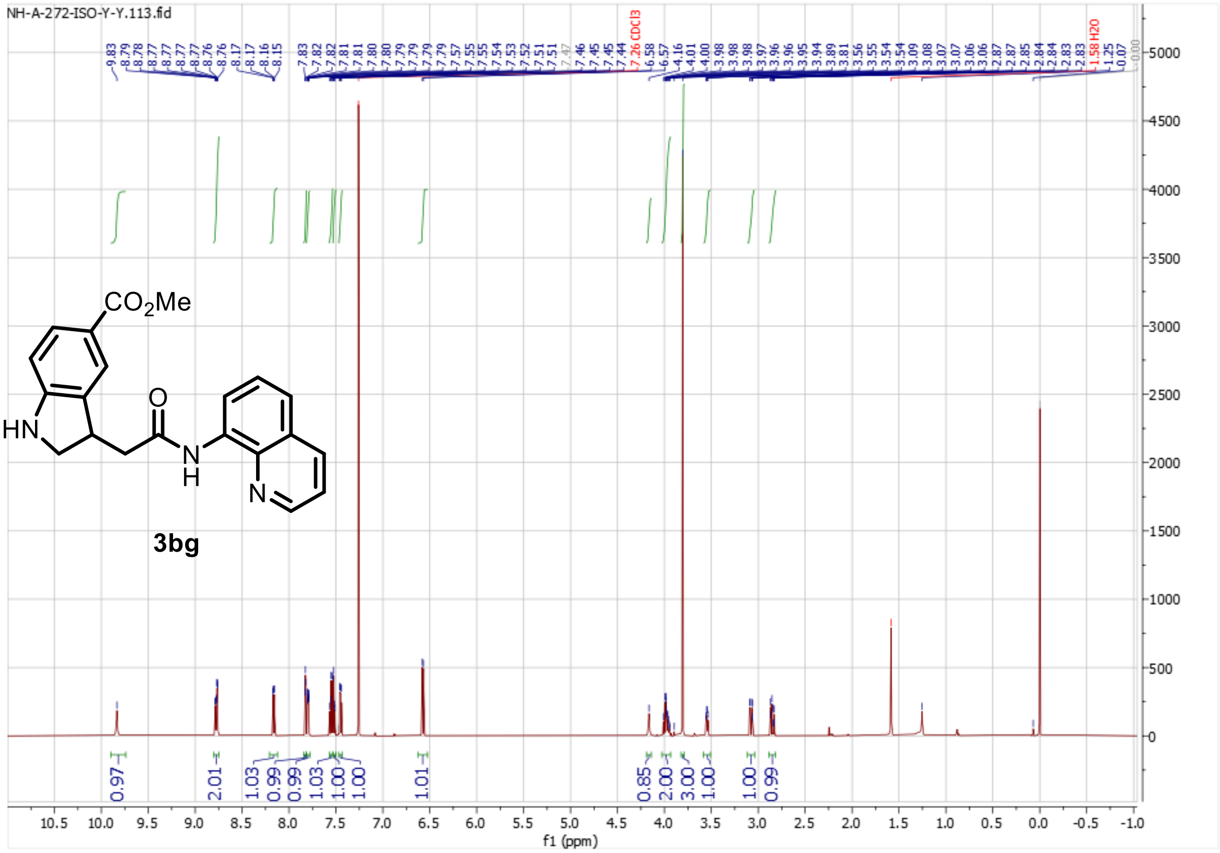
NH-A-272-ISO-Y-Y.113.fid
CO_2Me
HN
O
N
H
N
f1 (ppm)

3bg

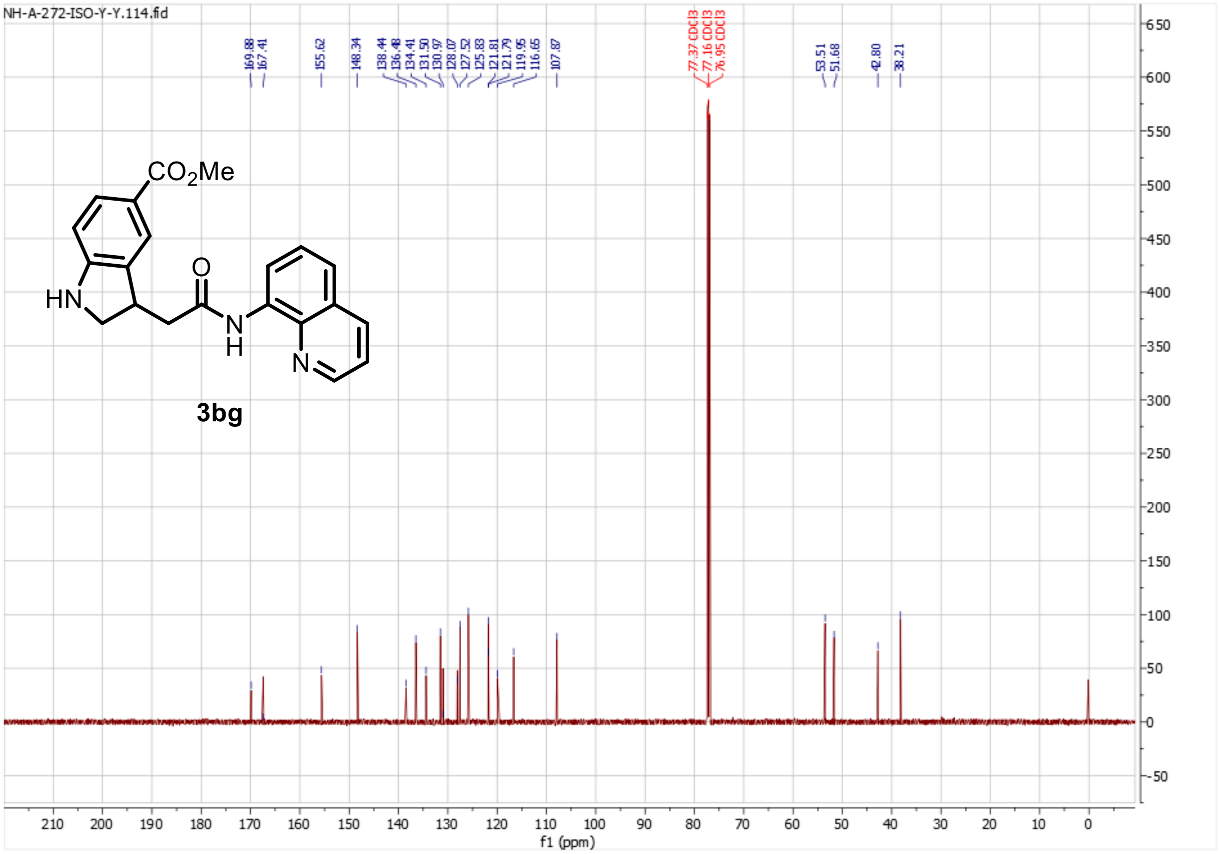
NH-A-272-ISO-Y-Y.114.fid
169.88
167.41
155.62
148.34
138.44
136.48
134.41
131.50
130.97
128.07
127.52
125.83
121.81
121.79
119.95
116.65
107.87
77.37 CDCl3
77.16 CDCl3
76.95 CDCl3
53.51
51.68
42.80
38.21
CO2Me
HN
O
N
H
N
3bg
f1 (ppm)

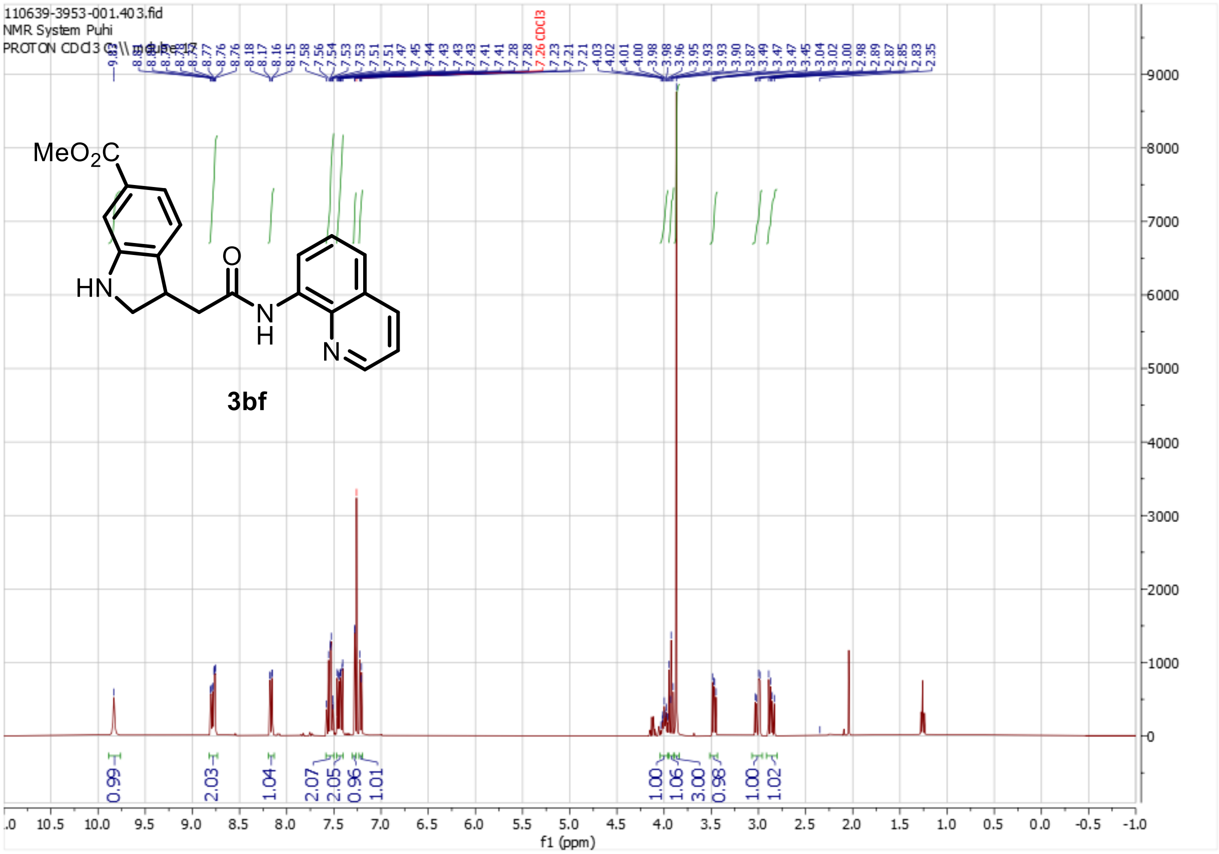
110639-3953-001.403.fid
NMR System Puhi
PROTON CDCl3
7.26 CDCl3
MeO2C
HN
O
N
H
N
3bf
0.99
2.03
1.04
2.07
2.05
0.96
1.01
1.00
1.06
3.00
0.98
1.00
1.02
f1 (ppm)

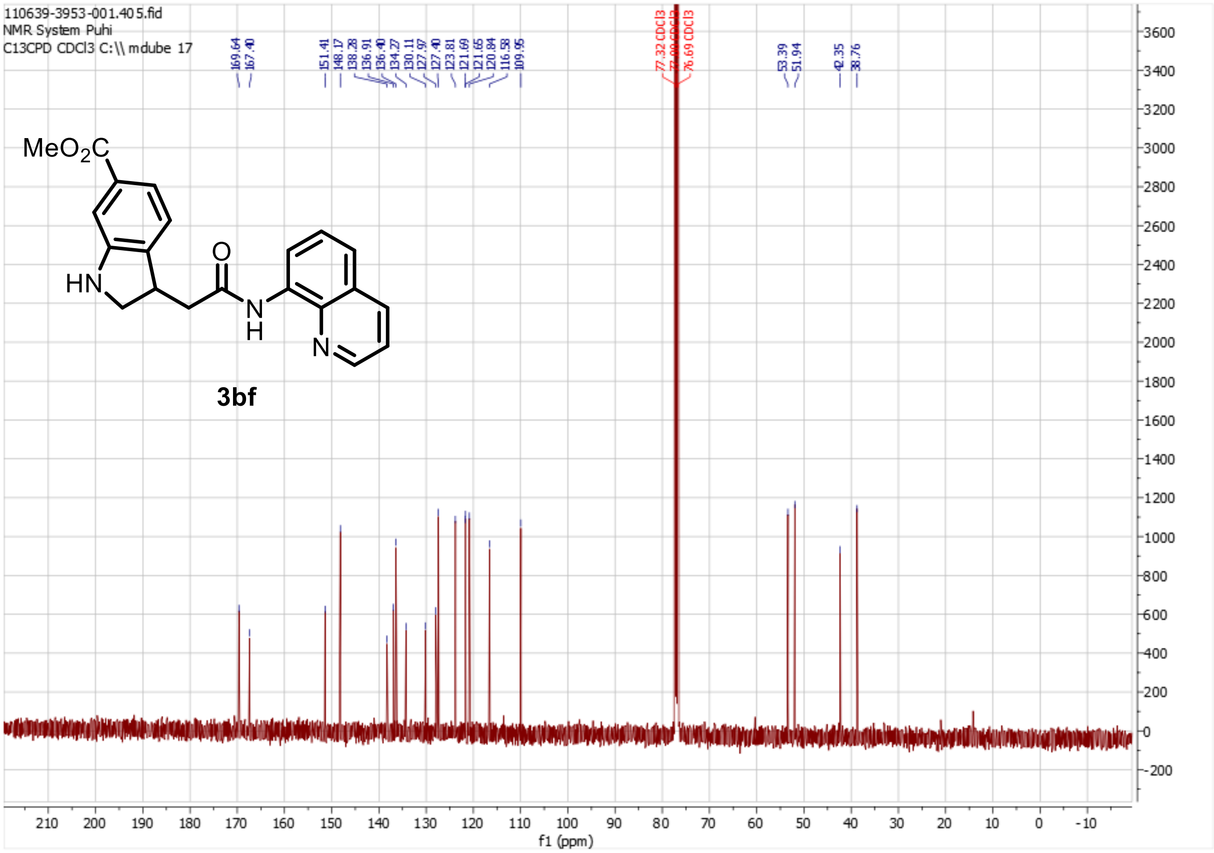
110639-3953-001.405.fid
NMR System Puhi
C13CPD CDCl3 C:\\ mdube 17
169.64
167.40
151.41
148.17
138.28
136.91
136.40
134.27
130.11
127.97
127.40
123.81
121.69
121.65
120.84
116.58
109.95
77.32 CDCl3
76.69 CDCl3
53.39
51.94
42.35
38.76
MeO2C
HN
O
N
H
N
3bf
f1 (ppm)

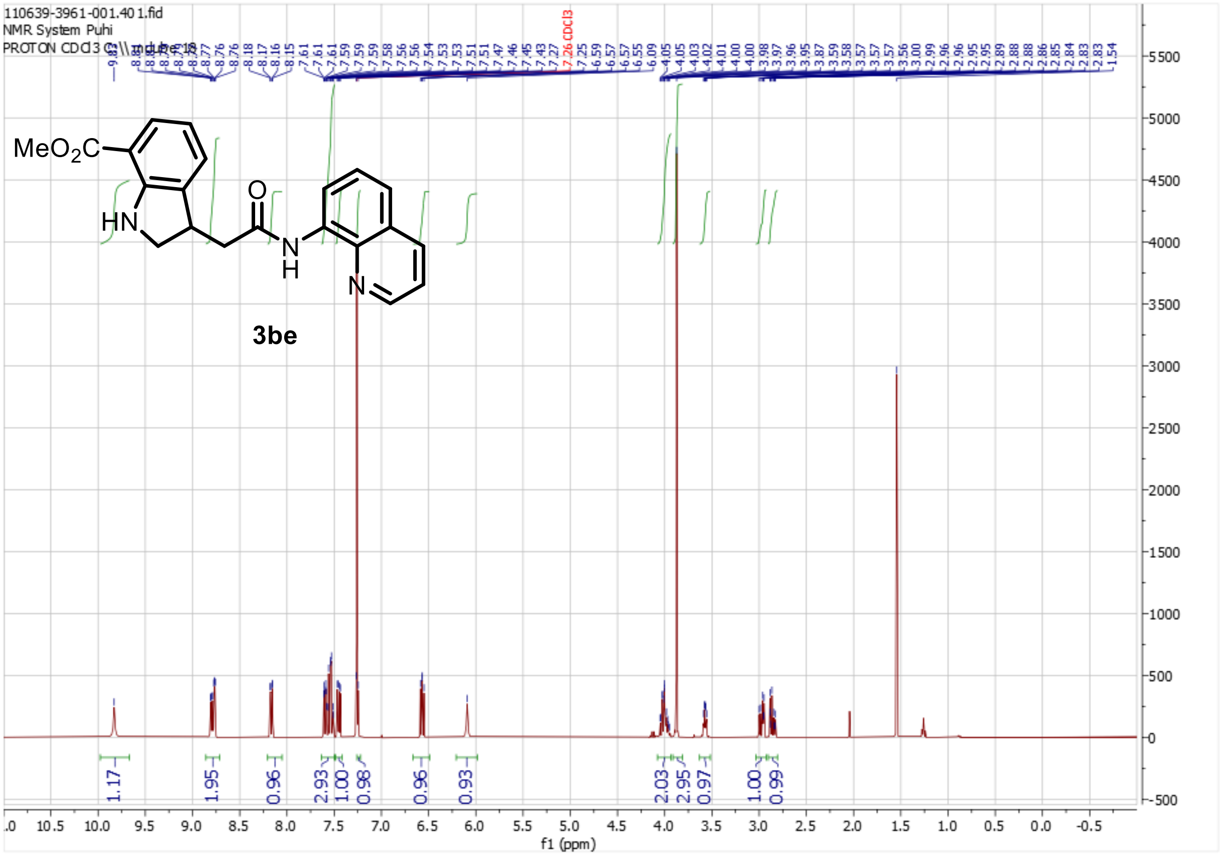
110639-3961-001.401.fid
NMR System Puhi
PROTON CDCl3
7.26 CDCl3
MeO2C
HN
O
N
H
N
3be
1.17
1.95
0.96
2.93
1.00
0.98
0.96
0.93
2.03
2.95
0.97
1.00
0.99
f1 (ppm)

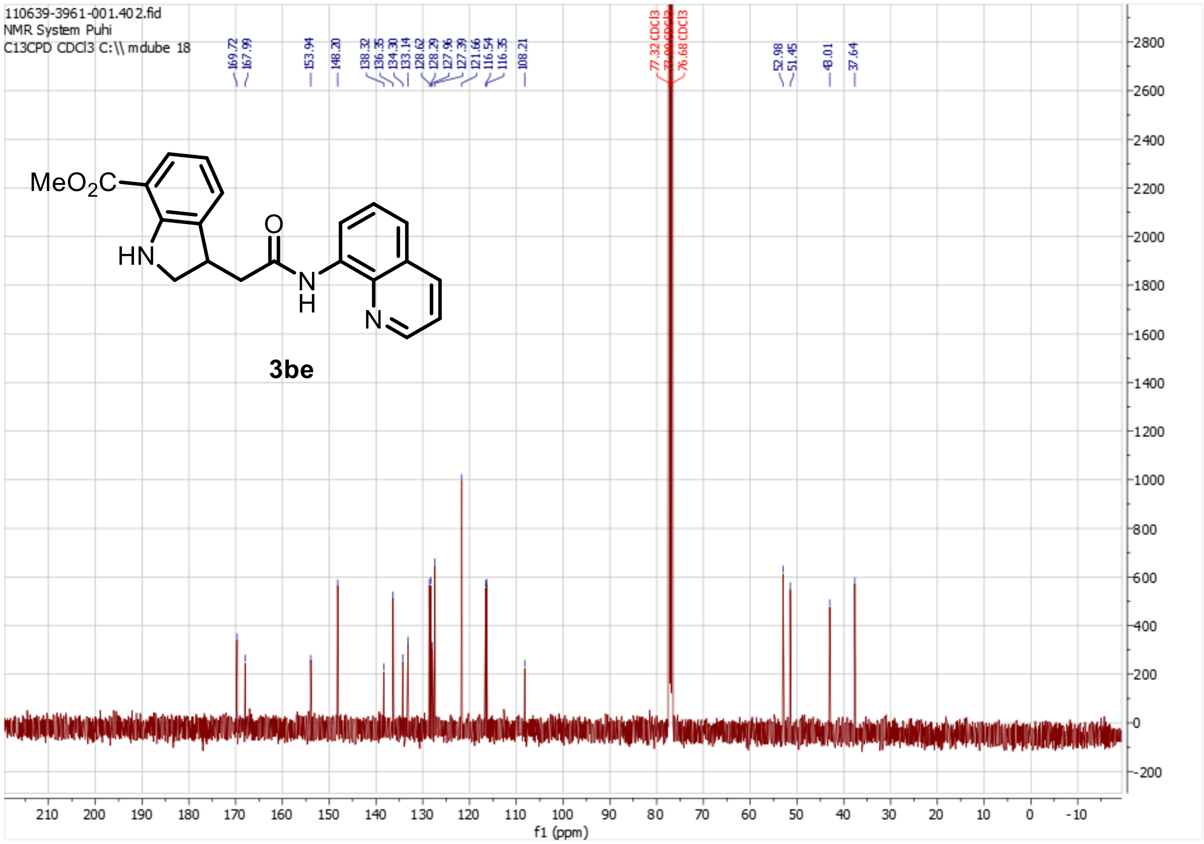
110639-3961-001.402.fid
NMR System Puhi
C13CPD CDCl3 C:\\ mdube 18
169.72
167.99
153.94
148.20
138.32
136.35
134.30
133.14
128.62
128.29
127.96
127.39
121.66
116.54
116.35
108.21
77.32 CDCl3
76.68 CDCl3
52.98
51.45
43.01
37.64
MeO2C
HN
O
N
H
N
3be
f1 (ppm)

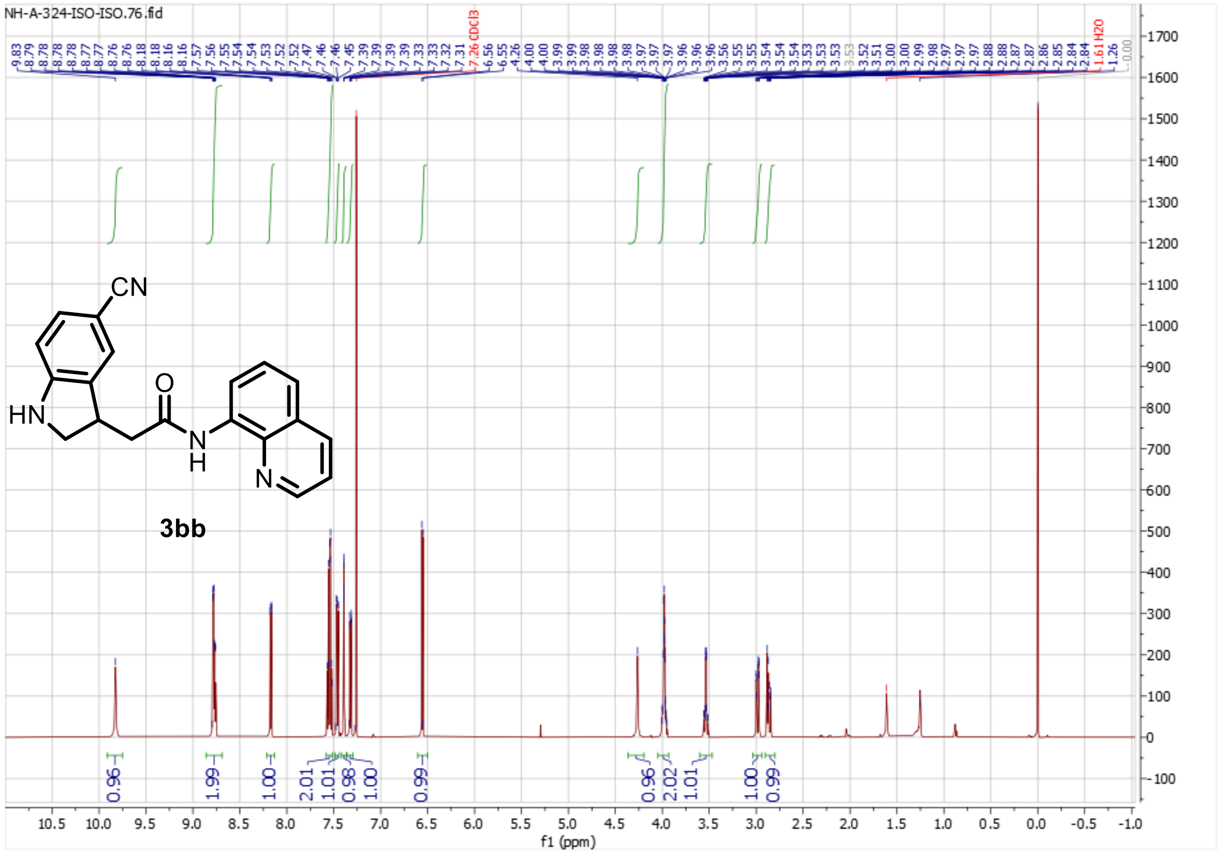
NH-A-324-ISO-ISO.76.fid
7.26 CDCl3
1.61 H2O
CN
HN
O
N
H
N
3bb
0.96
1.99
1.00
2.01
1.01
0.98
1.00
0.99
0.96
2.02
1.01
1.00
0.99
f1 (ppm)

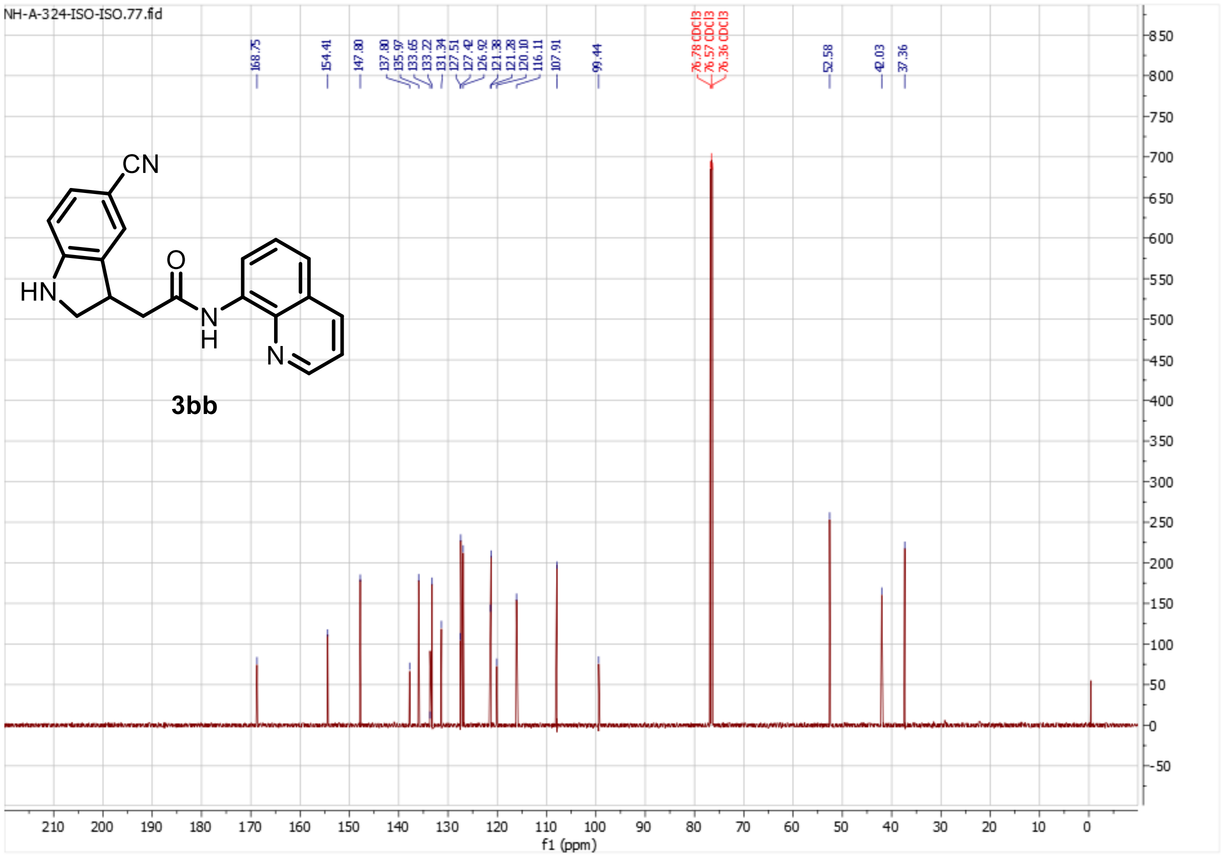
NH-A-324-ISO-ISO.77.fid
CN
HN
O
N
H
N
3bb
f1 (ppm)

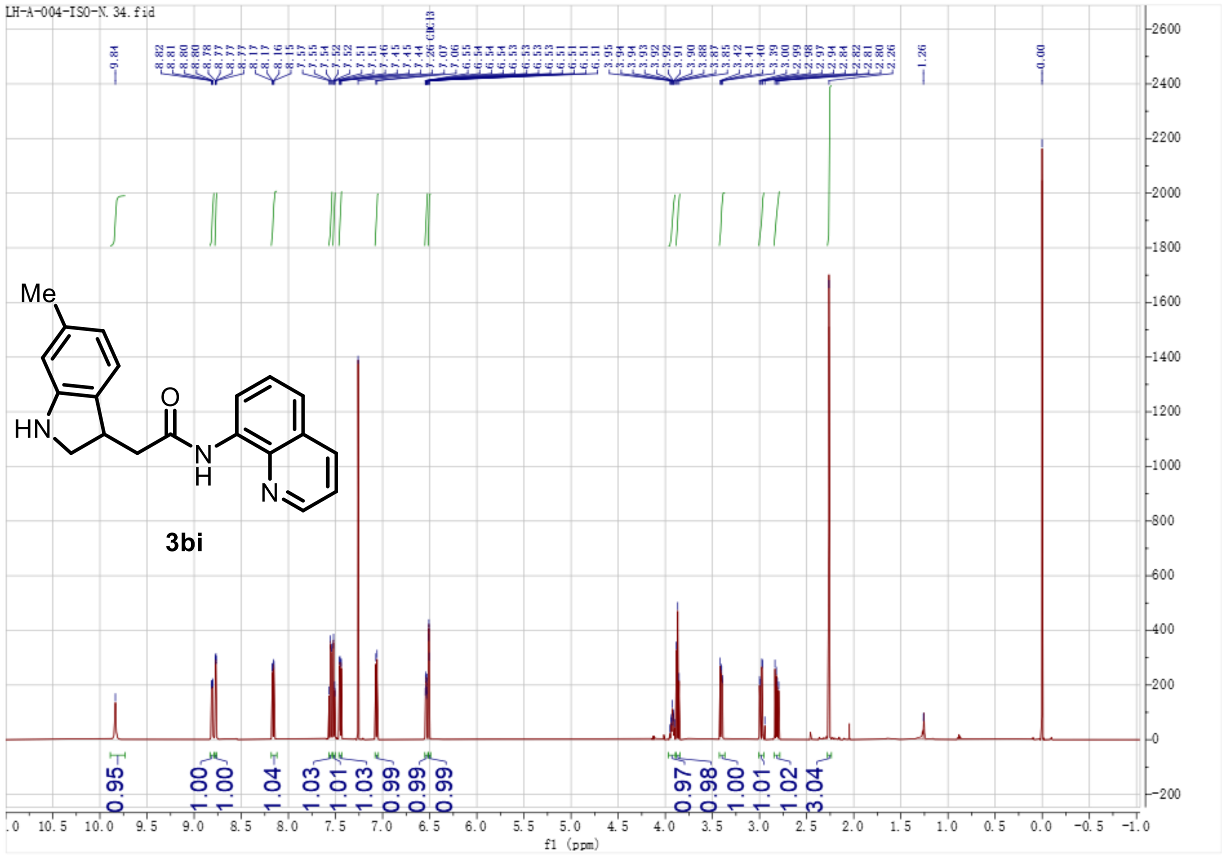
LH-A-004-ISO-N. 34. fid
Me
HN
O
N
H
N
3bi
f1 (ppm)

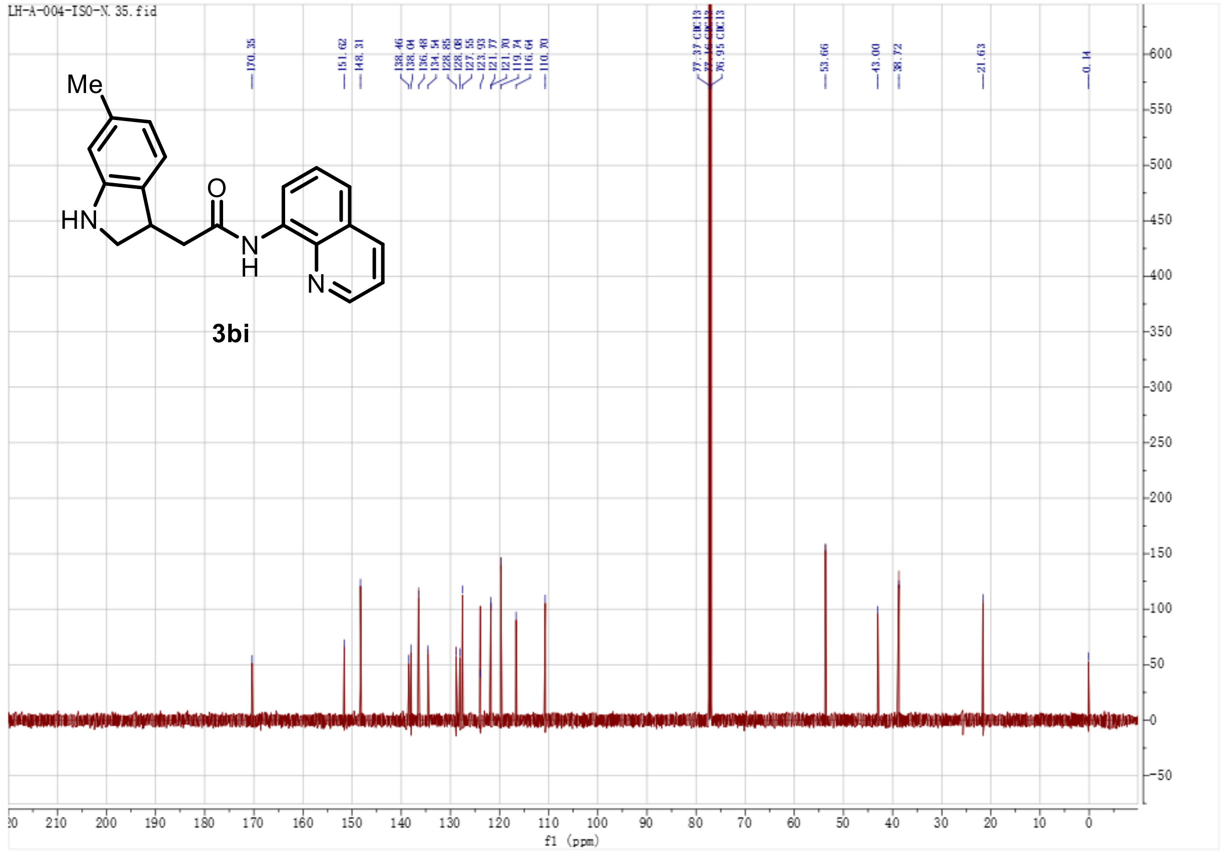

3bi

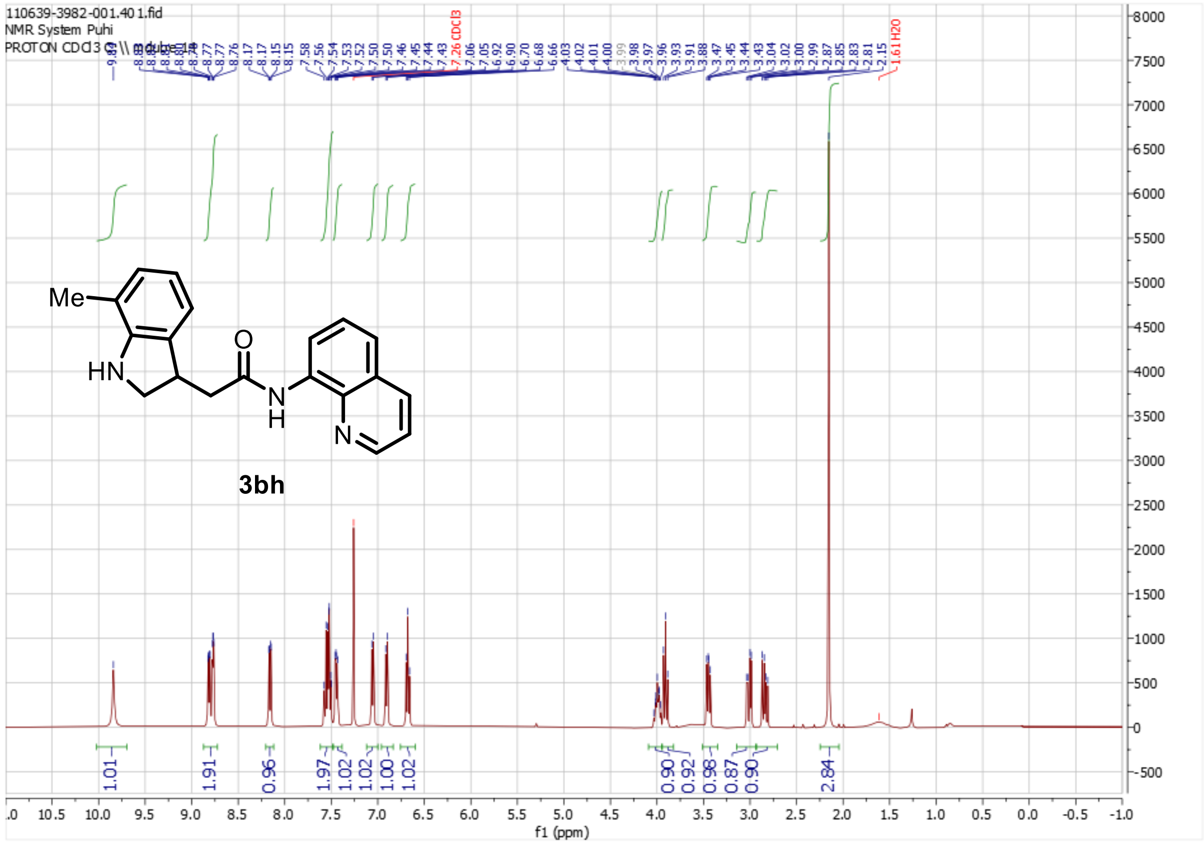

3bh

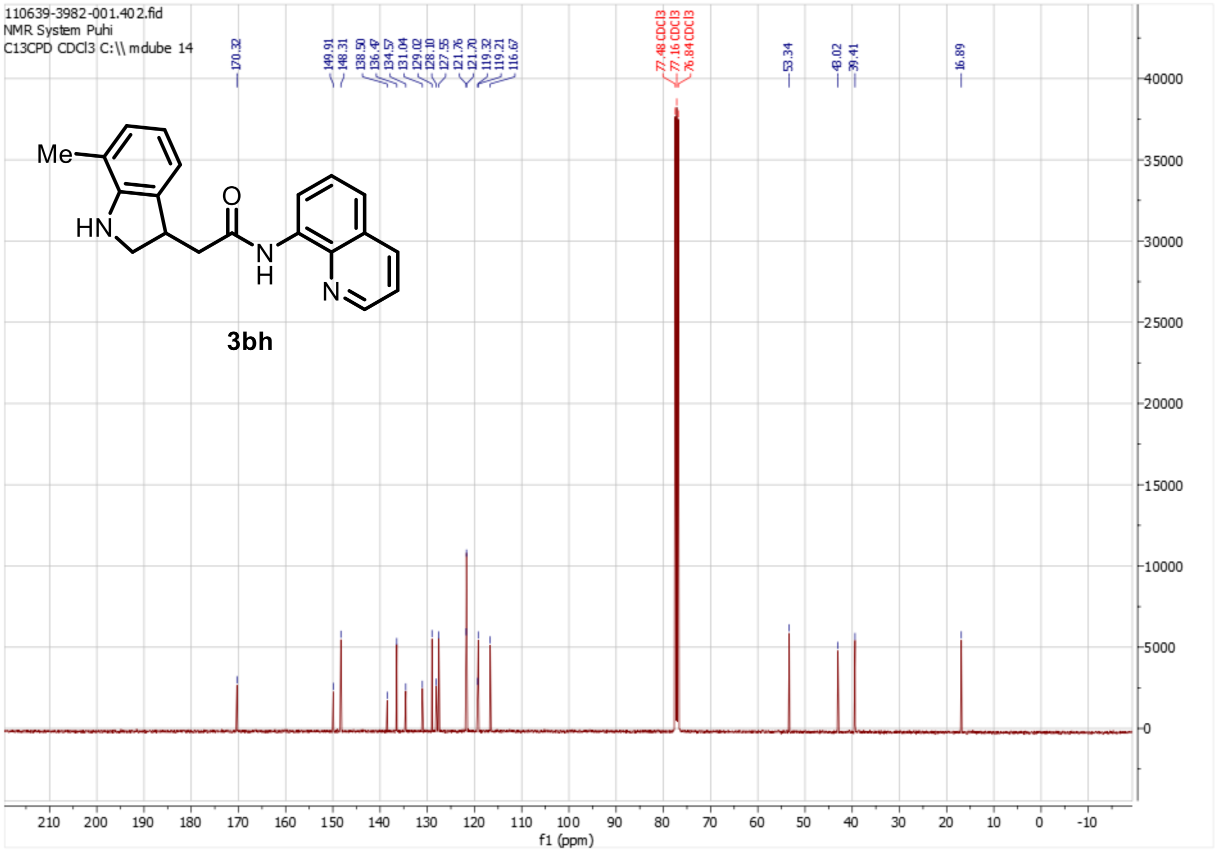
110639-3982-001.402.fid
NMR System Puhi
C13CPD CDCl3 C:\\ mdube 14
Me
HN
O
N
H
N
f1 (ppm)

3bh

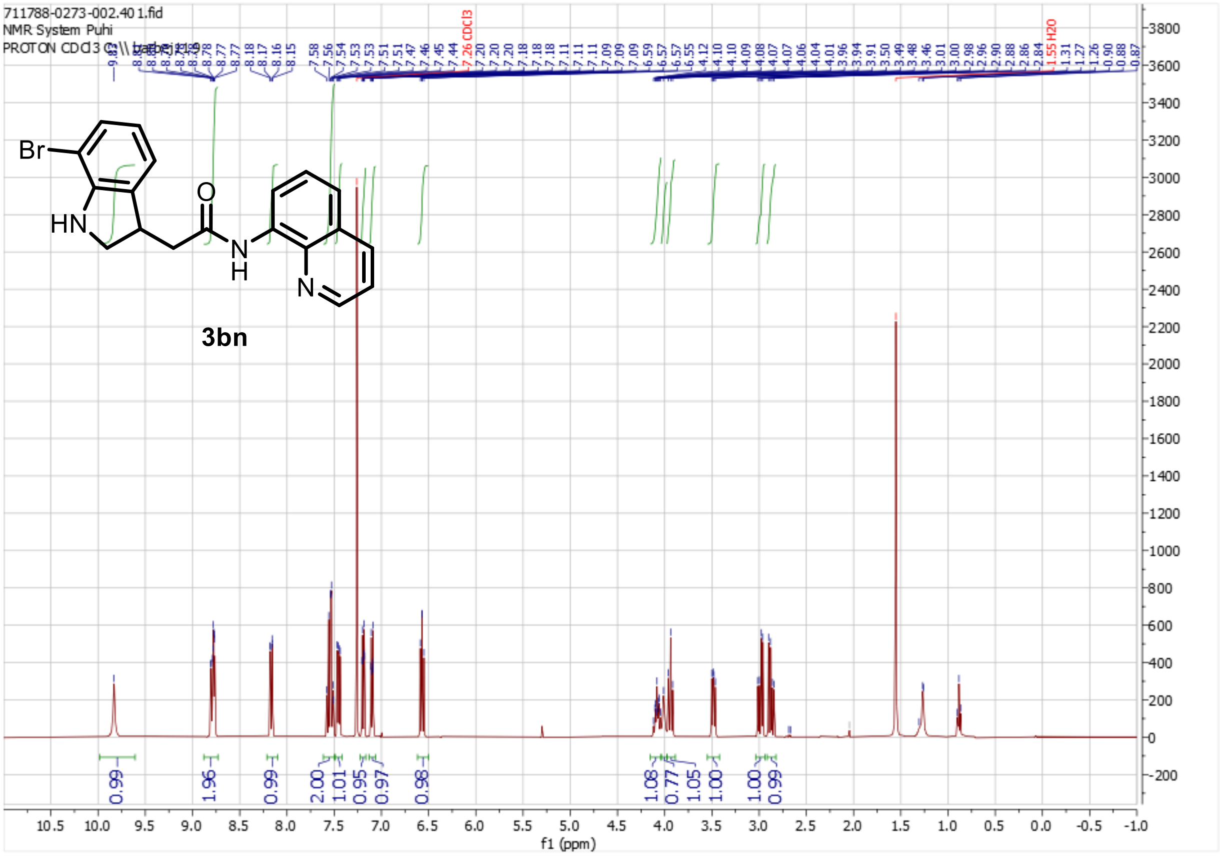
711788-0273-002.401.fid
NMR System Puhi
PROTON CDCl3
Br
HN
O
N
H
N
f1 (ppm)

3bn

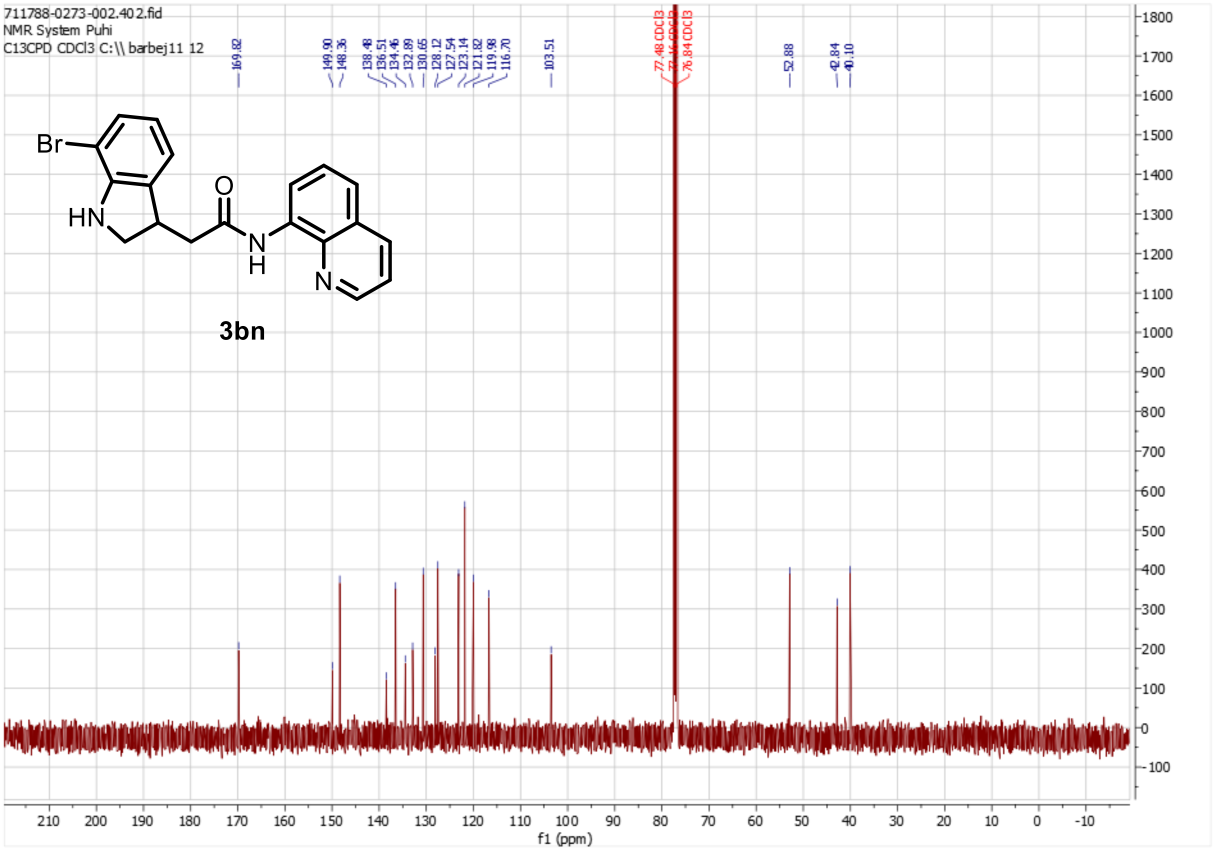
711788-0273-002.402.fid
NMR System Puhi
C13CPD CDCl3 C:\\ barbej11 12
169.82
149.90
148.36
138.48
136.51
134.46
132.89
130.65
128.12
127.54
123.14
121.82
119.98
116.70
103.51
77.48 CDCl3
76.84 CDCl3
52.88
42.84
40.10
Br
HN
O
N
H
N
3bn
f1 (ppm)

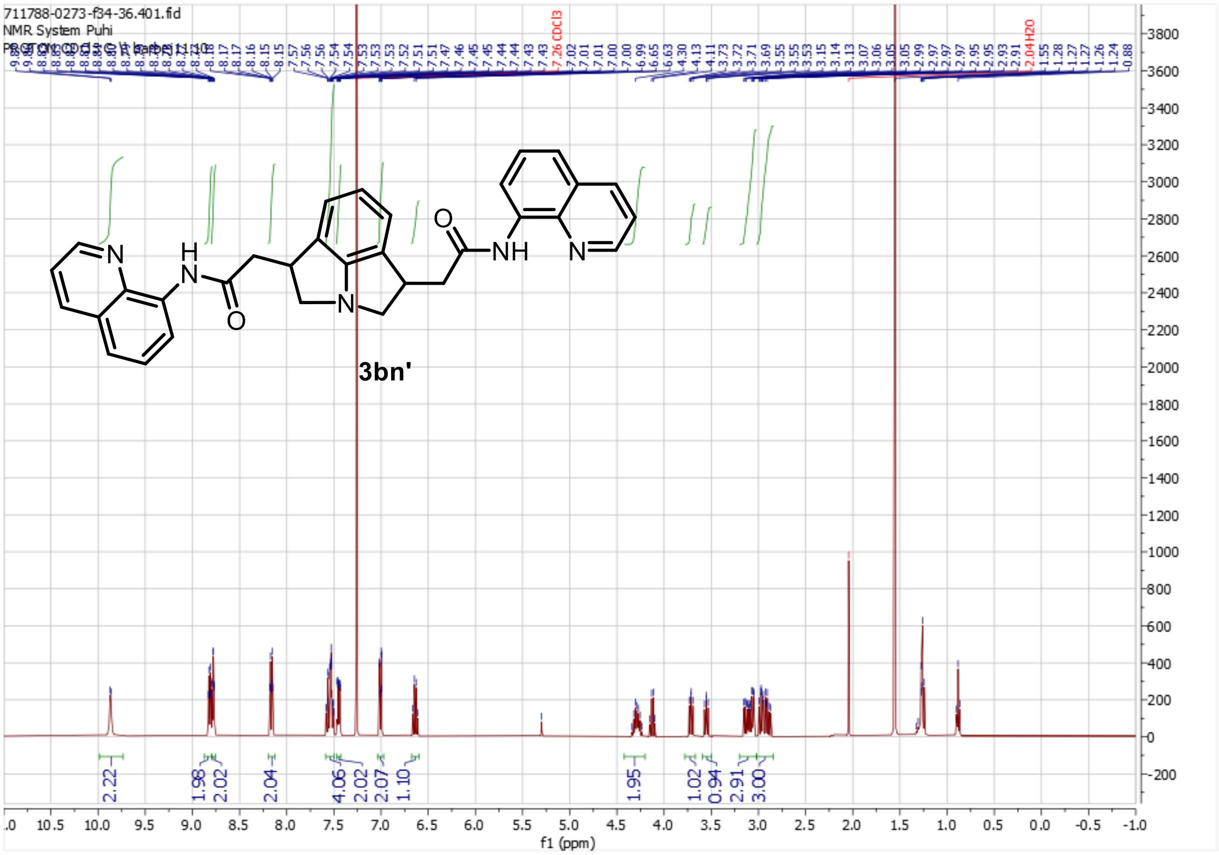
711788-0273-f34-36.401.fid
NMR System Puhi
7.26 CDCl3
2.04 H2O
N
H
N
O
N
O
NH
N
3bn'
2.22
1.98
2.02
2.04
4.06
2.02
2.07
1.10
1.95
1.02
0.94
2.91
3.00
f1 (ppm)

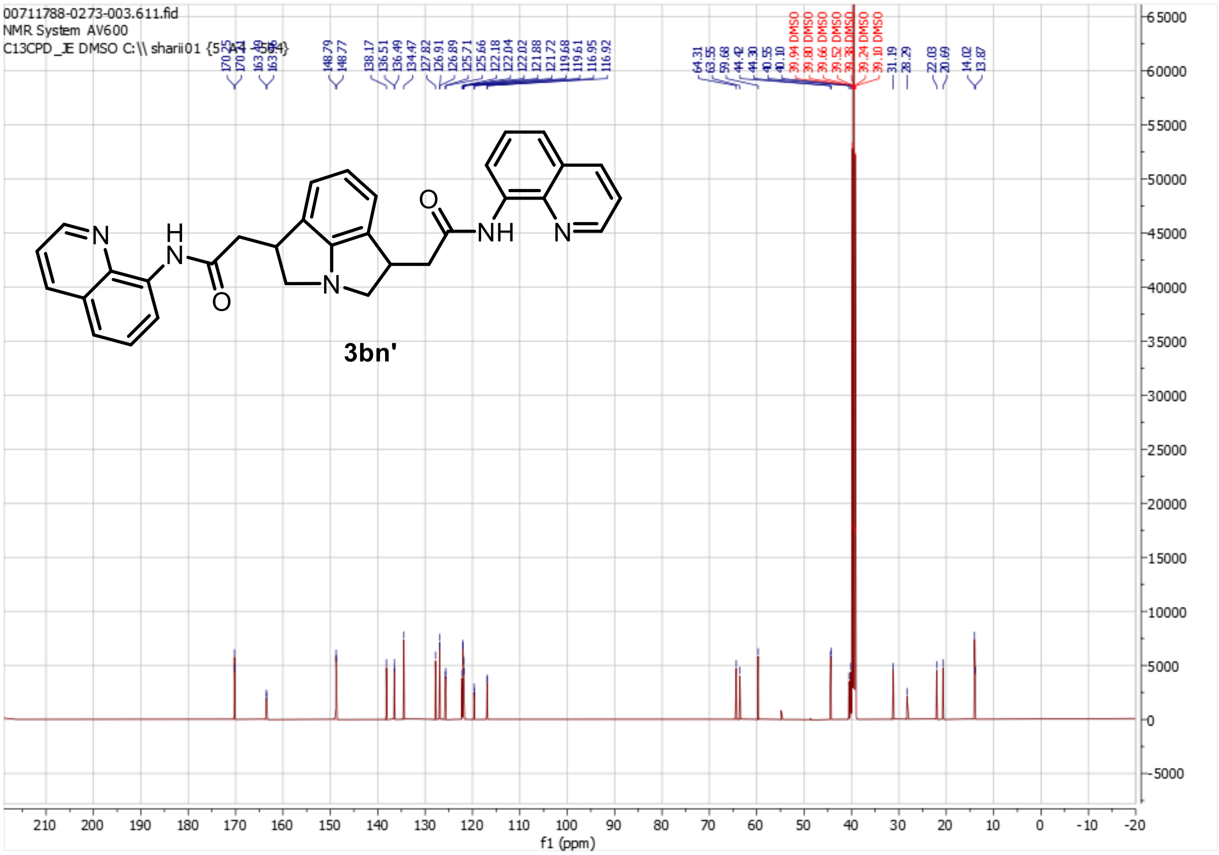

Open Access Analytical Sciences
http://links/OASIS

Spectrum is consistent with proposed structure.
Impurities observed: Ethyl acetate and hexane

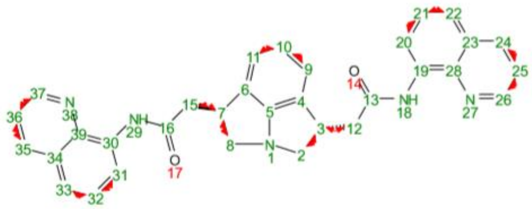

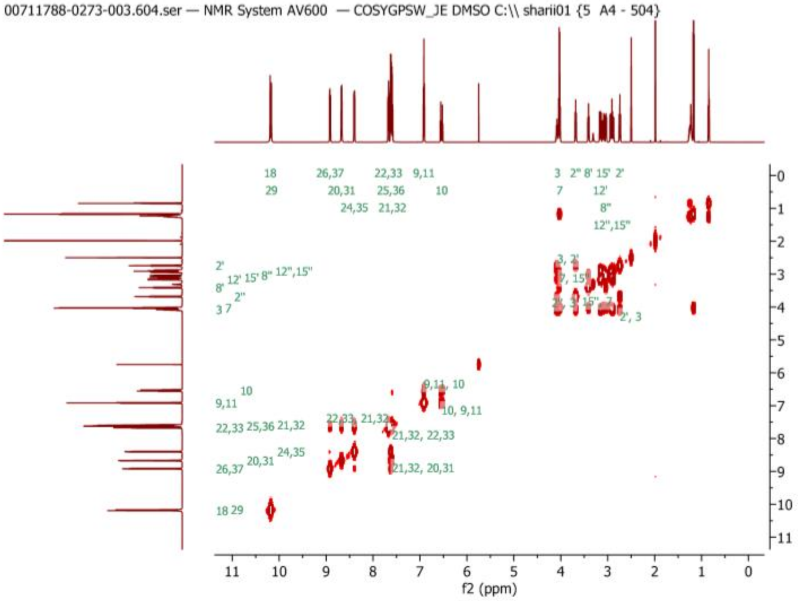

Parameters

	Parameter	Value
1	Solvent	DMSO
2	Temperature	303.2
3	Pulse Sequence	cosygpppqf
4	Number of Scans	4
5	Receiver Gain	17
6	Relaxation Delay	1.7579
7	Pulse Width	11.0000
8	Acquisition Time	0
9	Acquisition Date	2019-10-31T20:08:09
10	Modification Date	2019-10-31T20:24:47
11	Nucleus	(1H, 1H)
12	Absolute Reference	(Absolute Referencing used Me4Si CDCl3, φ = 1% and Ratio of 100.000000,)

Spectrum is consistent with proposed structure.
Impurities observed: Ethyl acetate and hexane

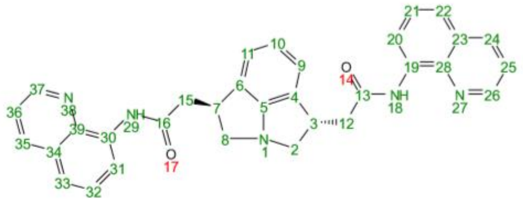

00711788-0273-003.605.ser — NMR System AV600 — HSQCEDETGP_JE DMSO C:\\ sharii01 {5 A4 - 504}

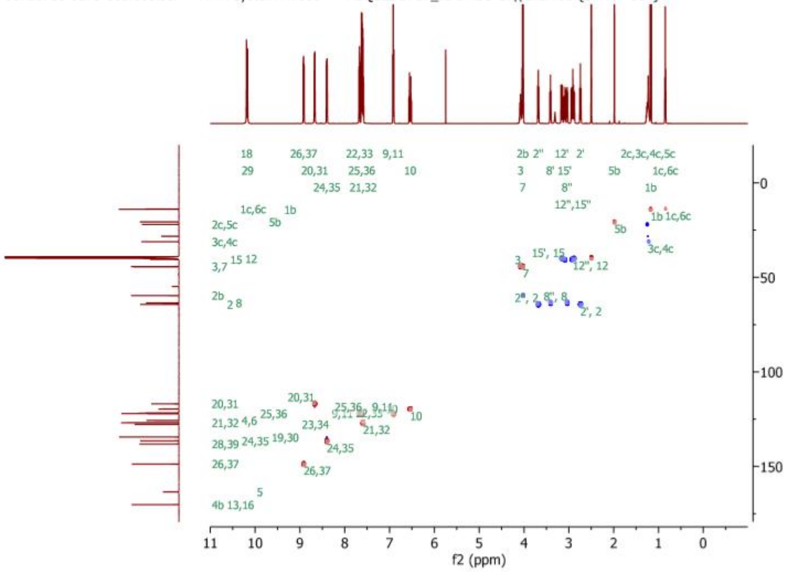

Parameters		
	Parameter	Value
1	Solvent	DMSO
2	Temperature	303.2
3	Pulse Sequence	hsqcedetgp
4	Number of Scans	16
5	Receiver Gain	181
6	Relaxation Delay	1.5000
7	Pulse Width	11.0000
8	Acquisition Time	0
9	Acquisition Date	2019-10-31T20:26:43
10	Modification Date	2019-10-31T20:58:52
11	Nucleus	(1H, 13C)
12	Absolute Reference	(Absolute Referencing used Me4Si CDCl3, φ = 1% and Ratio of 100.000000,)

Spectrum is consistent with proposed structure.
Impurities observed: Ethyl acetate and hexane

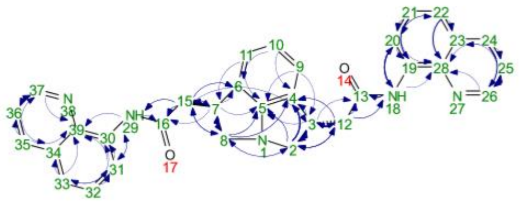

00711788-0273-003.607.ser — NMR System AV600 — HMBCGP_JE DMSO C:\\ sharii01 {5 A4 - 504}

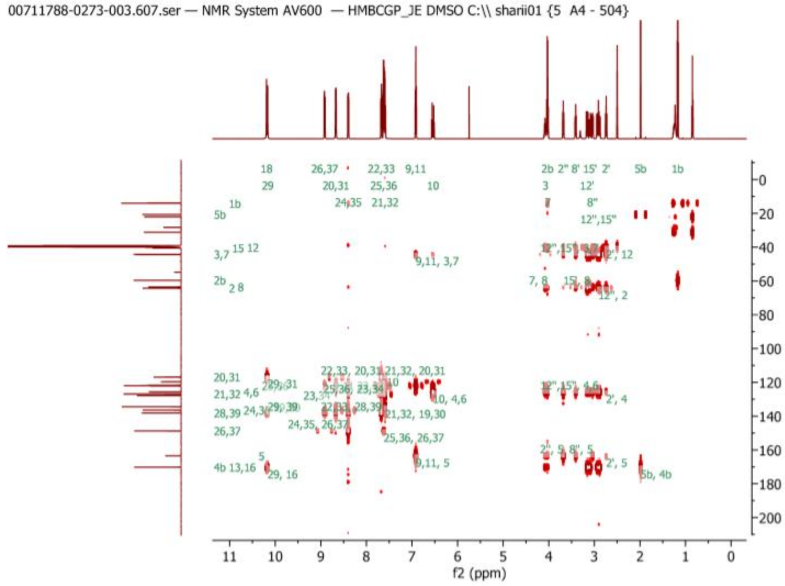

Parameters		
	Parameter	Value
1	Solvent	DMSO
2	Temperature	303.1
3	Pulse Sequence	hmbcgplpndqf
4	Number of Scans	128
5	Receiver Gain	181
6	Relaxation Delay	1.5000
7	Pulse Width	11.0000
8	Acquisition Time	0
9	Acquisition Date	2019-10-31T22:19:28
10	Modification Date	2019-11-01T00:34:28
11	Nucleus	(1H, 13C)
12	Absolute Reference	(Absolute Referencing used Me4Si CDCl3, φ = 1% and Ratio of 100.000000,)

Spectrum is consistent with proposed structure.
Impurities observed: Ethyl acetate and hexane

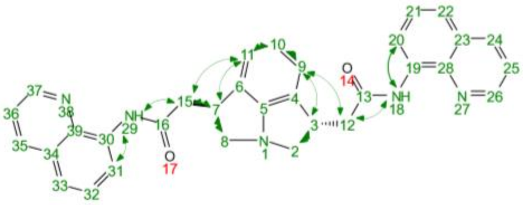

00711788-0273-003.606.ser — NMR System AV600 — NOESYPHSW_JE DMSO C:\\ sharii01 {5 A4 - 504}

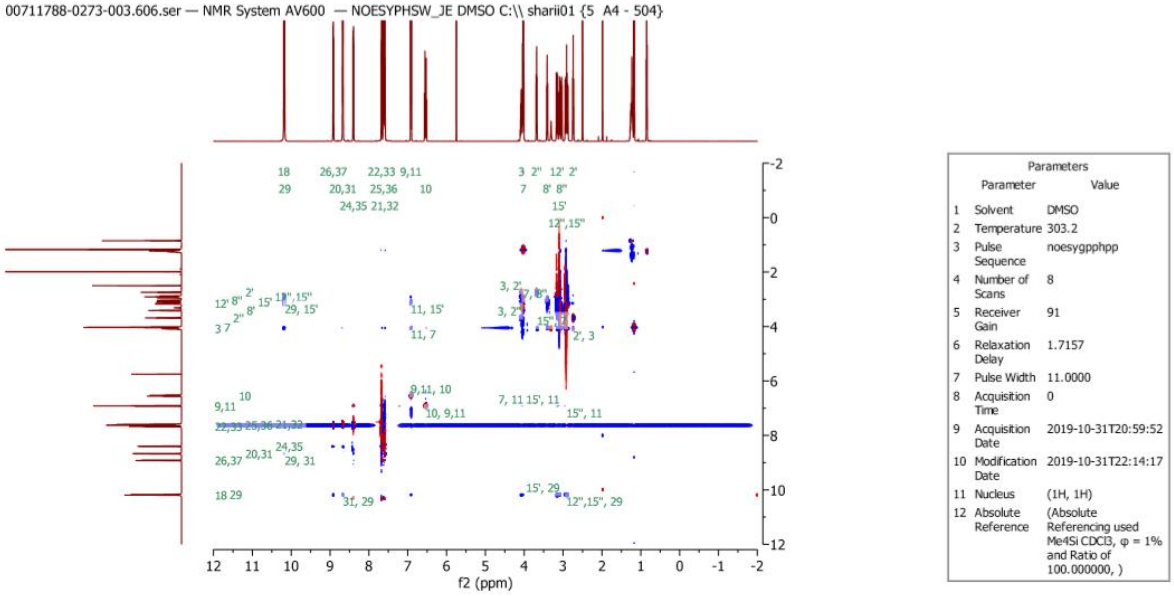

Parameters

	Parameter	Value
1	Solvent	DMSO
2	Temperature	303.2
3	Pulse Sequence	noesygpphpp
4	Number of Scans	8
5	Receiver Gain	91
6	Relaxation Delay	1.7157
7	Pulse Width	11.0000
8	Acquisition Time	0
9	Acquisition Date	2019-10-31T20:59:52
10	Modification Date	2019-10-31T22:14:17
11	Nucleus	(1H, 1H)
12	Absolute Reference	(Absolute Referencing used Me4Si CDCl3, φ = 1% and Ratio of 100.000000,)

Spectrum is consistent with proposed structure.
Impurities observed: Ethyl acetate and hexane

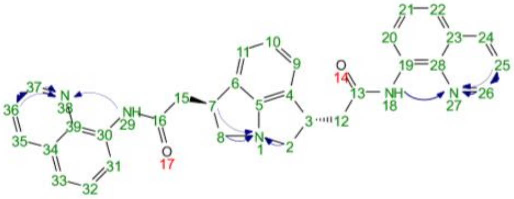

00711788-0273-003.609.ser — NMR System AV600 — HMBC15N_JE DMSO C:\\ sharii01 {5 A4 - 504}

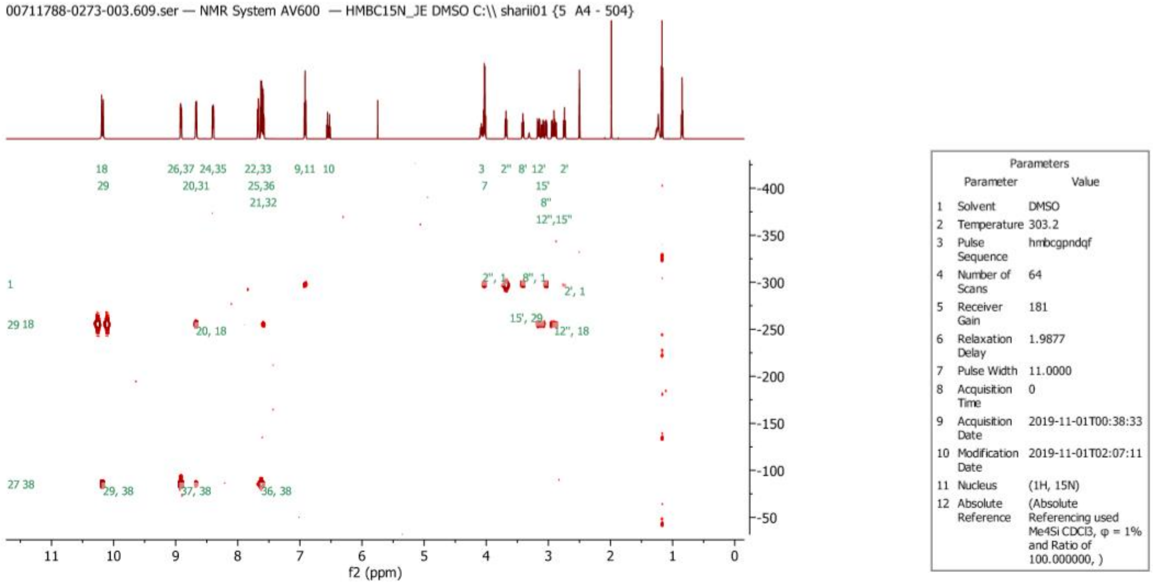

Parameters

	Parameter	Value
1	Solvent	DMSO
2	Temperature	303.2
3	Pulse Sequence	hmbcgpndqf
4	Number of Scans	64
5	Receiver Gain	181
6	Relaxation Delay	1.9877
7	Pulse Width	11.0000
8	Acquisition Time	0
9	Acquisition Date	2019-11-01T00:38:33
10	Modification Date	2019-11-01T02:07:11
11	Nucleus	(1H, 15N)
12	Absolute Reference	(Absolute Referencing used Me4Si CDCl3, φ = 1% and Ratio of 100.000000,)

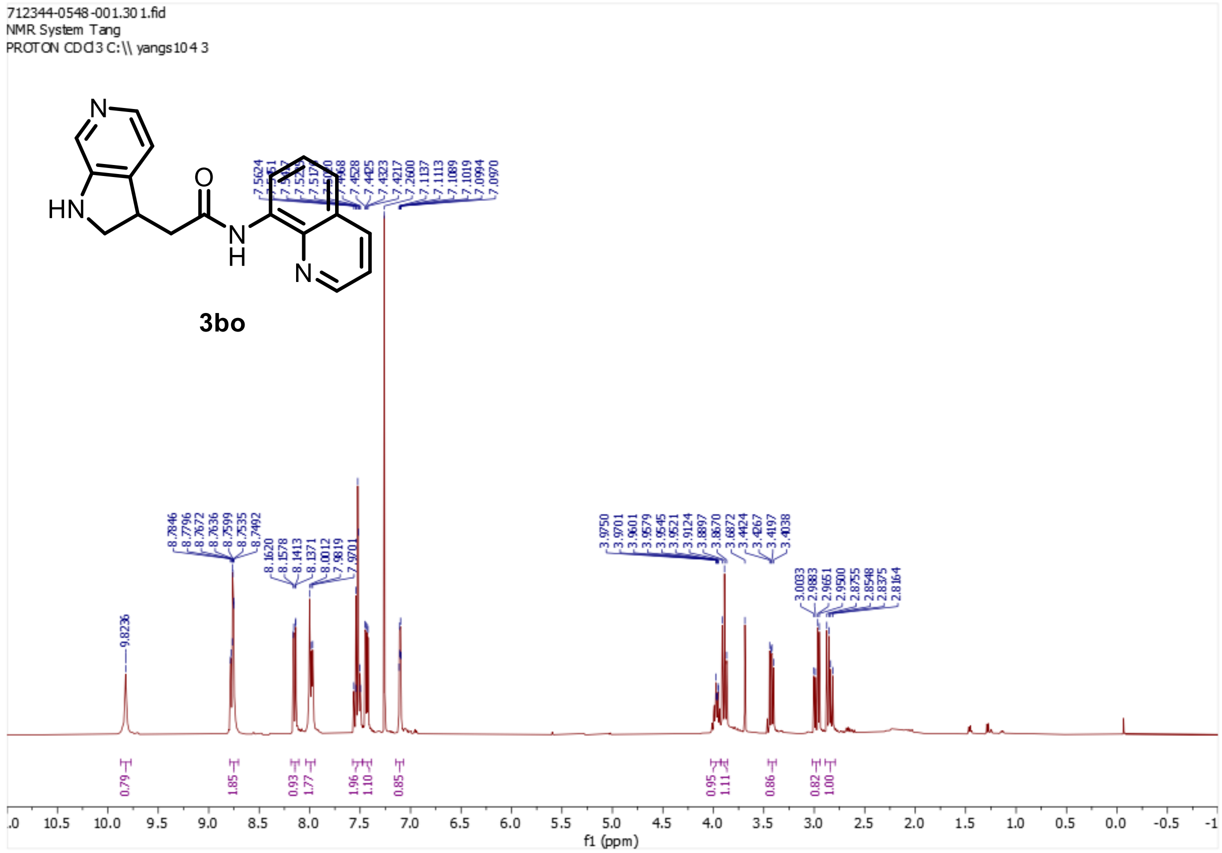
712344-0548-001.301.fid
NMR System Tang
PROTON CDCl3 C:\\ yangs104 3
HN
N
O
N
H
N
3bo
f1 (ppm)

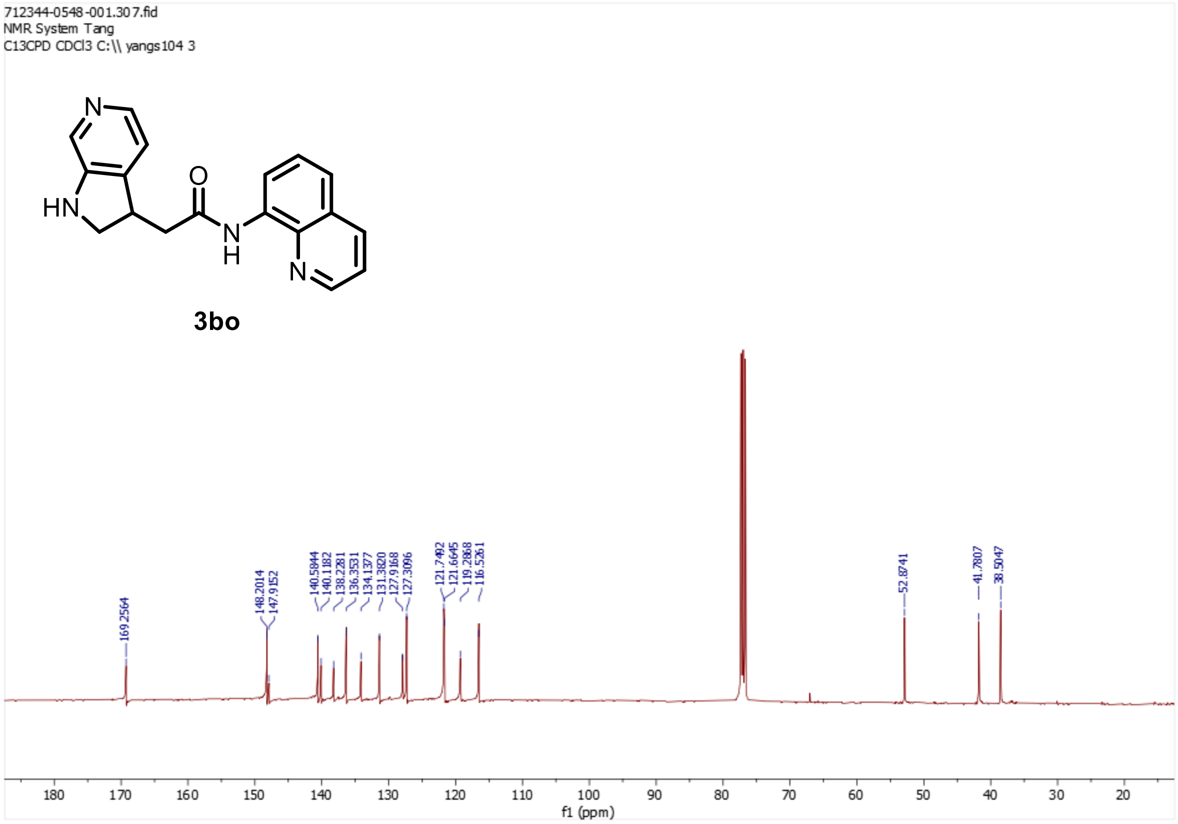
712344-0548-001.307.fid
NMR System Tang
C13CPD CDCl3 C:\\ yangs104 3
HN
N
O
N
H
N
3bo
169.2564
148.2014
147.9152
140.5844
140.1182
138.2281
136.3531
134.1377
131.3820
127.9168
127.3096
121.7492
121.6645
119.2868
116.5261
52.8741
41.7807
38.5047
f1 (ppm)

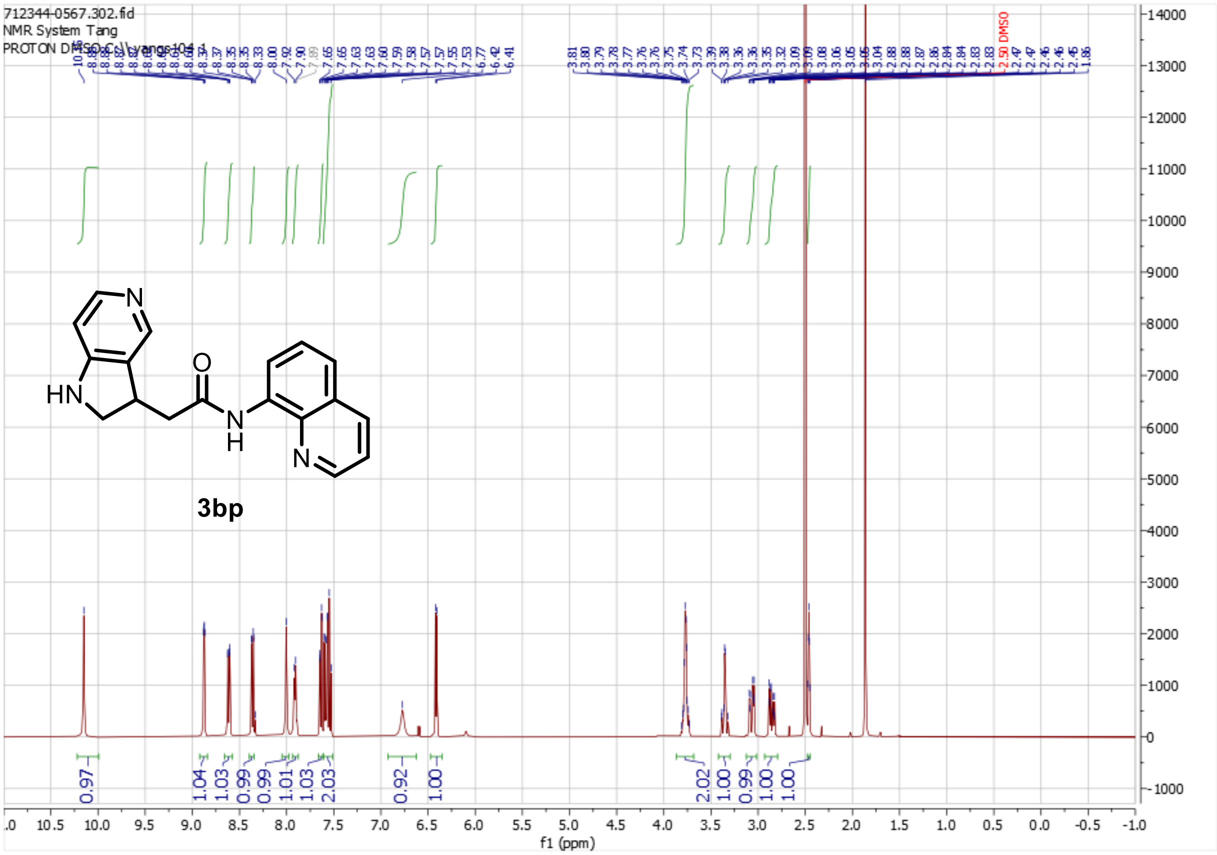
712344-0567.302.fid
NMR System Tang
PROTON DMSO C:\\ yangs104 1
HN
N
O
N
H
N
3bp
f1 (ppm)

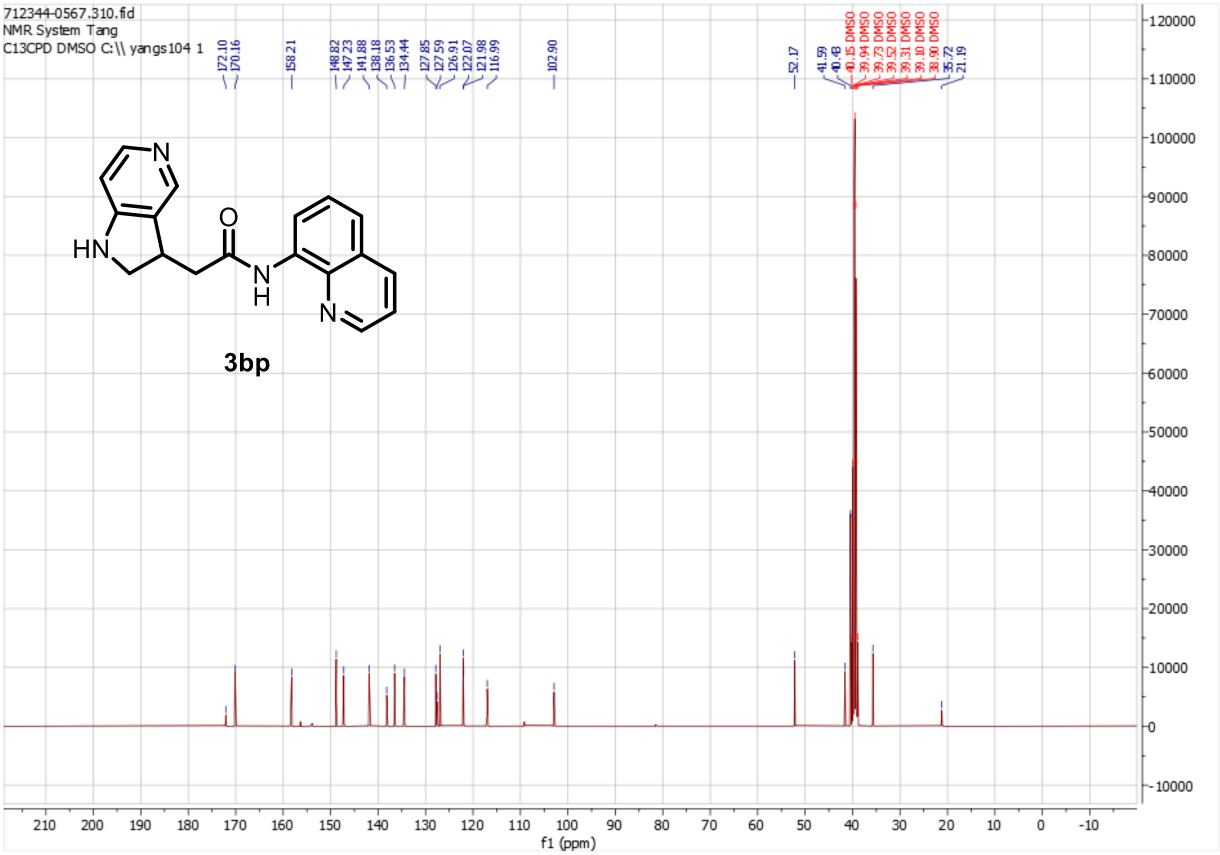
712344-0567.310.fid
NMR System Tang
C13CPD DMSO C:\\ yangs104 1
172.10
170.16
158.21
148.82
147.23
141.88
138.18
136.53
134.44
127.85
127.59
126.91
122.07
121.98
116.99
102.90
52.17
41.59
40.48
40.15 DMSO
39.94 DMSO
39.73 DMSO
39.52 DMSO
39.31 DMSO
39.10 DMSO
38.90 DMSO
35.72
21.19
HN
N
O
N
H
N
3bp
f1 (ppm)

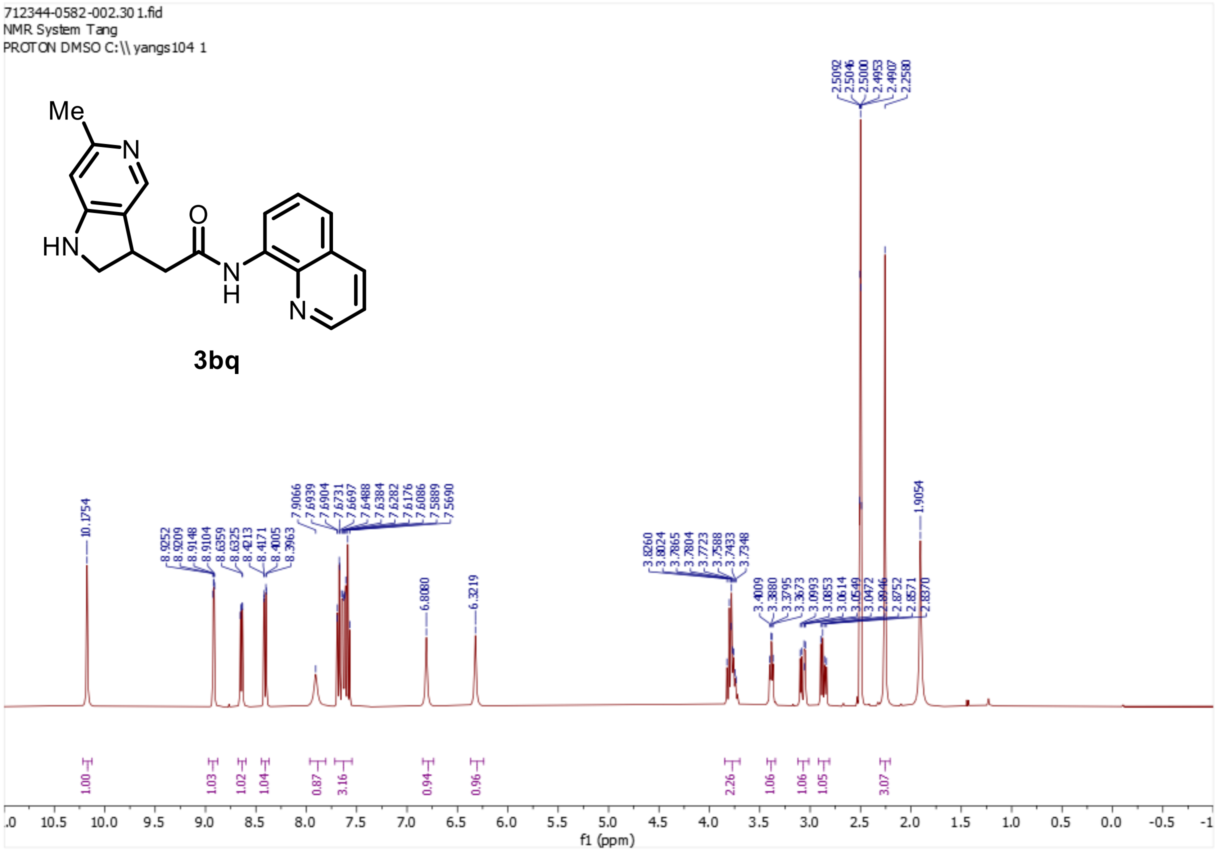
712344-0582-002.30 1.fid
NMR System Tang
PROTON DMSO C:\\ yangs104 1
Me
N
O
HN
N
H
N
3bq
10.1754
8.9252
8.9209
8.9148
8.9104
8.6359
8.6325
8.4213
8.4171
8.4005
8.3963
7.9066
7.6939
7.6904
7.6731
7.6697
7.6488
7.6384
7.6282
7.6176
7.6086
7.5889
7.5690
6.8080
6.3219
3.8260
3.8024
3.7865
3.7804
3.7723
3.7588
3.7433
3.7348
3.4009
3.3880
3.3795
3.3673
3.0993
3.0853
3.0614
3.0549
3.0472
2.8946
2.8752
2.8571
2.8370
2.5092
2.5046
2.5000
2.4953
2.4907
2.2580
1.9054
1.00
1.03
1.02
1.04
0.87
3.16
0.94
0.96
2.26
1.06
1.06
1.05
3.07
f1 (ppm)

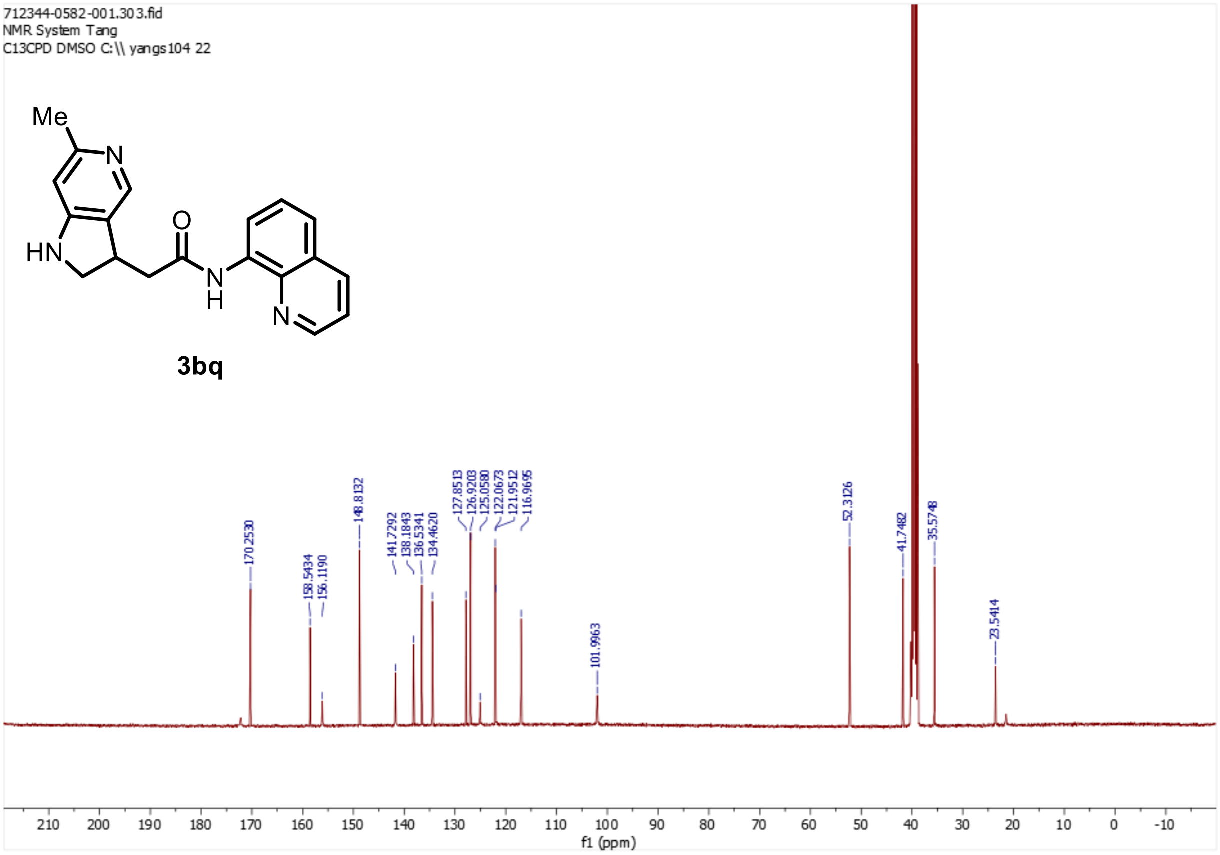
712344-0582-001.30 3.fid
NMR System Tang
C13CPD DMSO C:\\ yangs104 22
Me
N
O
HN
N
H
N
3bq
170.2530
158.5434
156.1190
148.8132
141.2292
138.1843
136.5341
134.4620
127.8513
126.9203
125.0580
122.0673
121.9512
116.9695
101.9963
52.3126
41.7482
35.5748
23.5414
f1 (ppm)

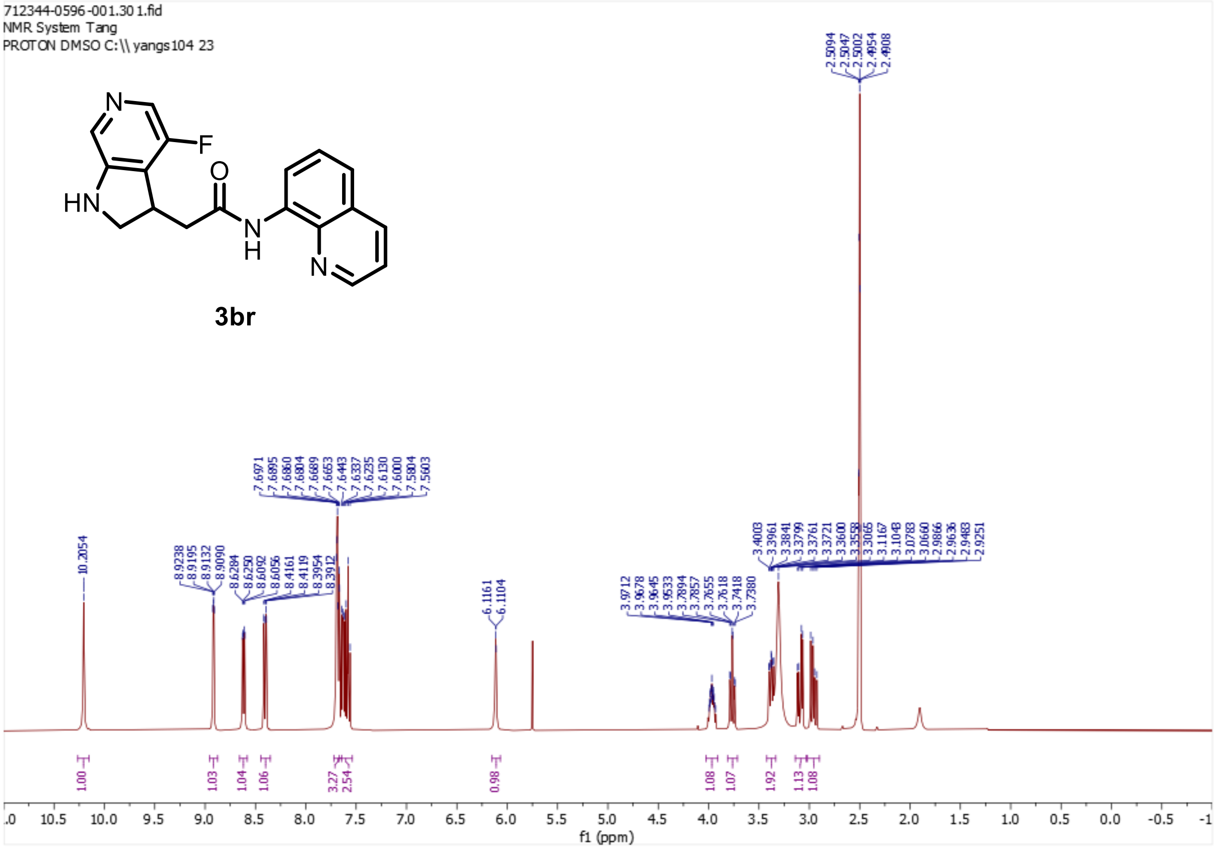
712344-0596-001.301.fid
NMR System Tang
PROTON DMSO C:\\ yangs104 23
3br
f1 (ppm)

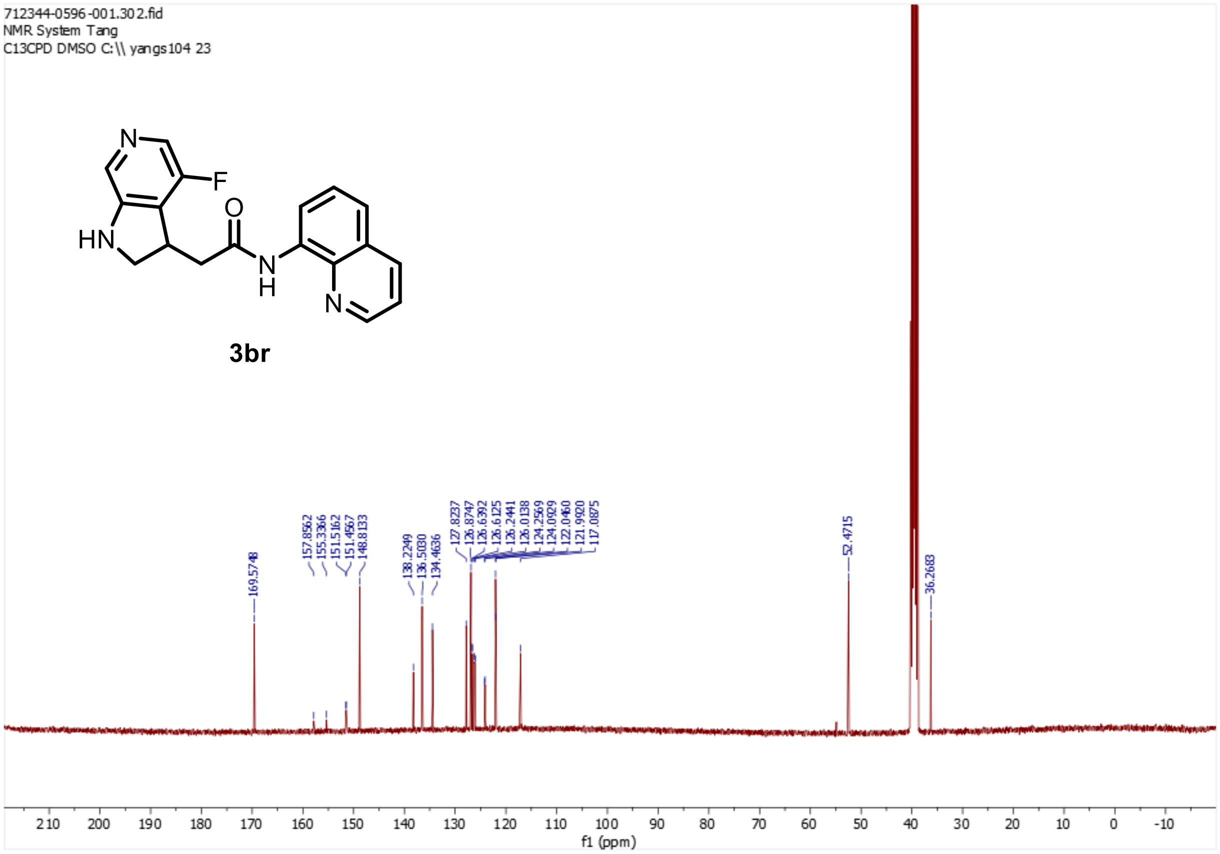
712344-0596-001.302.fid
NMR System Tang
C13CPD DMSO C:\\ yangs104 23
3br
f1 (ppm)

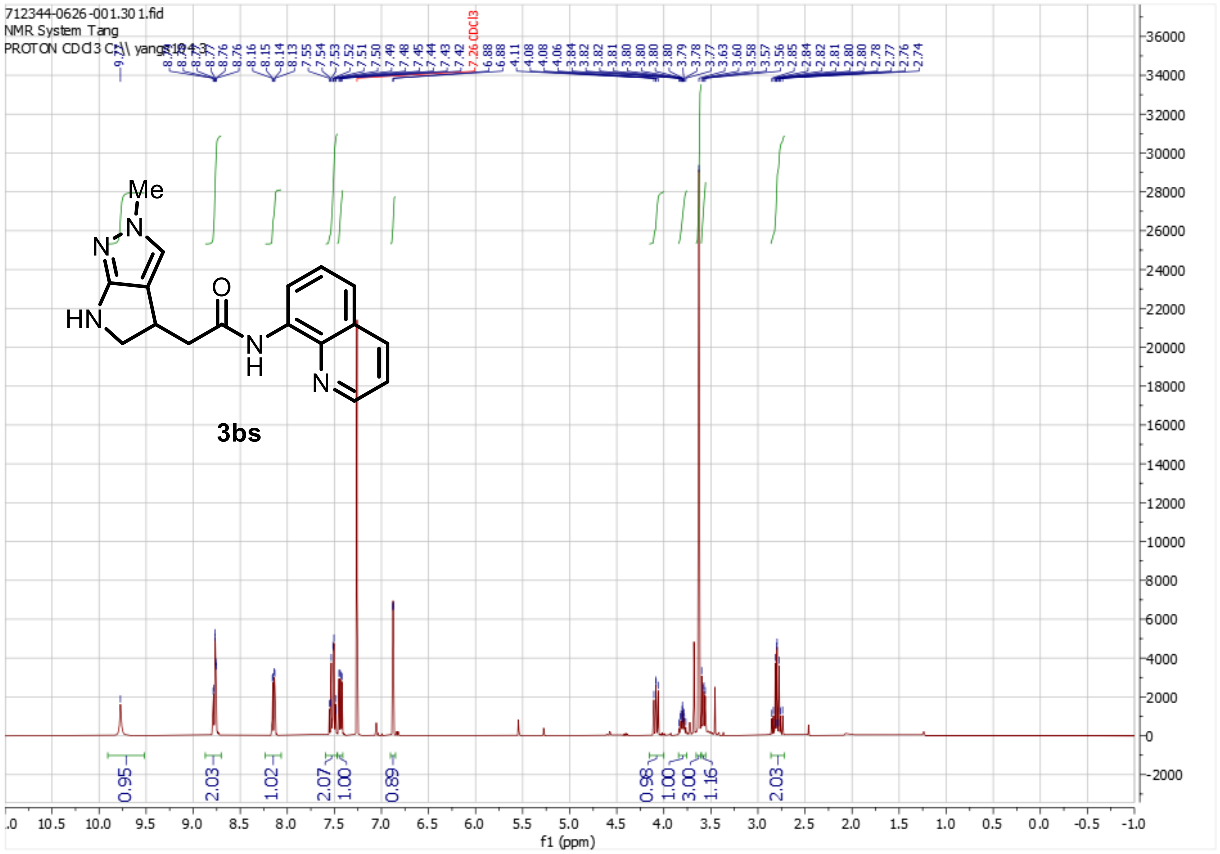
712344-0626-001.301.fid
NMR System Tang
PROTON CDCl3 C:\\ yangs104 3
3bs
f1 (ppm)

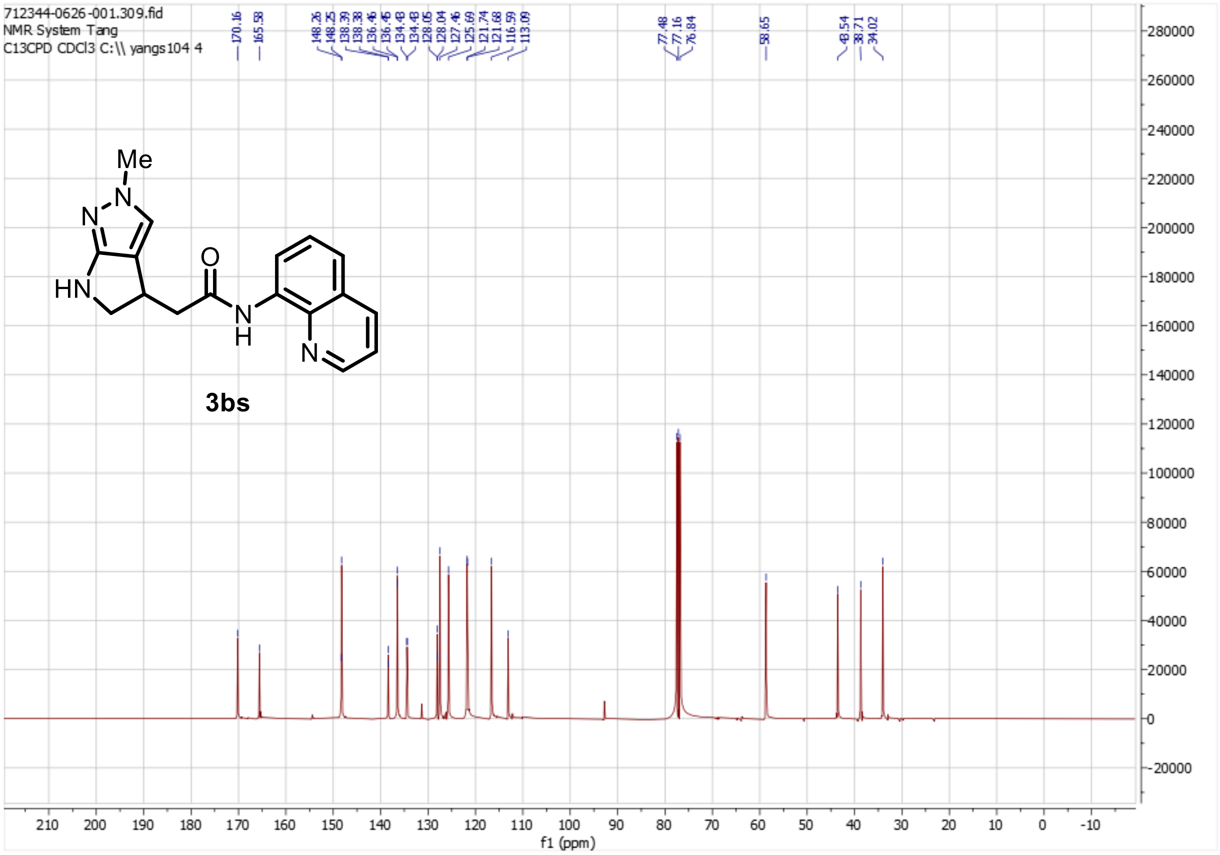
712344-0626-001.309.fid
NMR System Tang
C13CPD CDCl3 C:\\ yangs104 4
3bs
f1 (ppm)

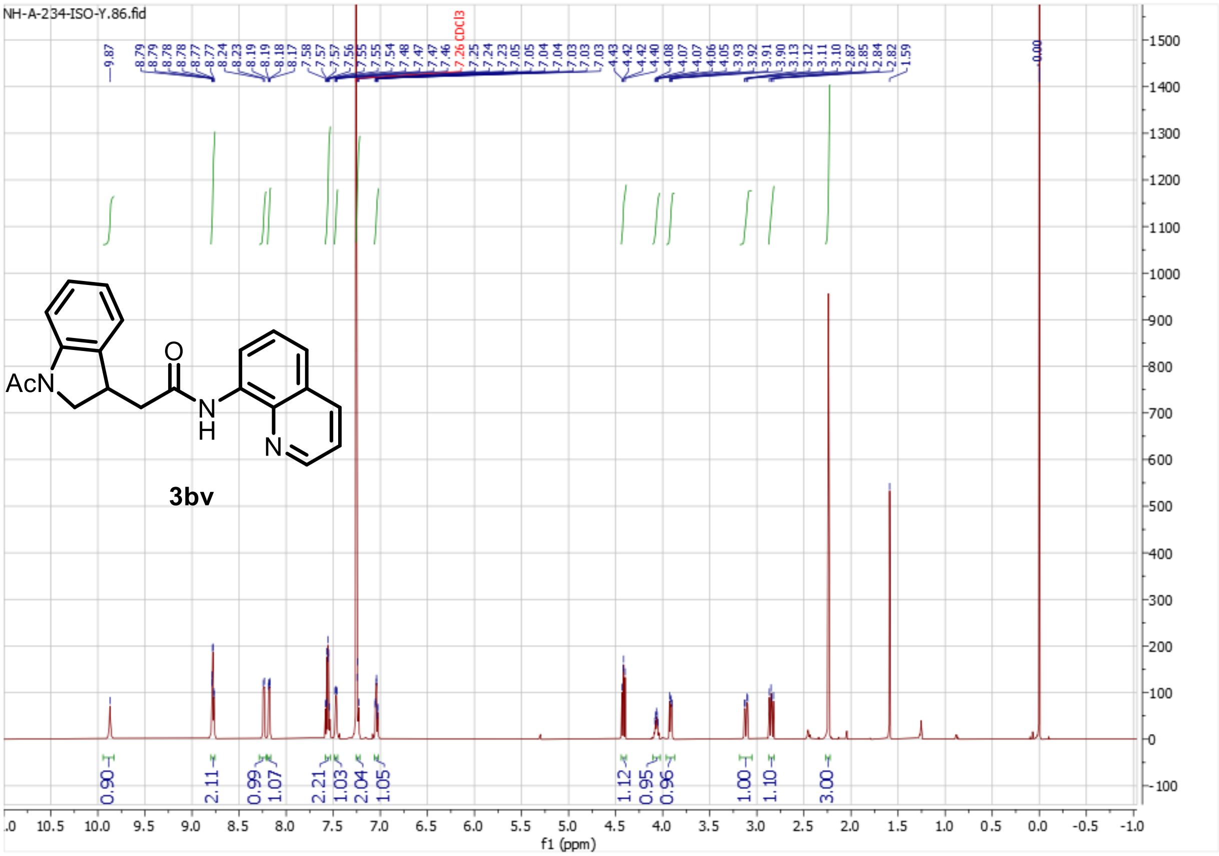

3bv

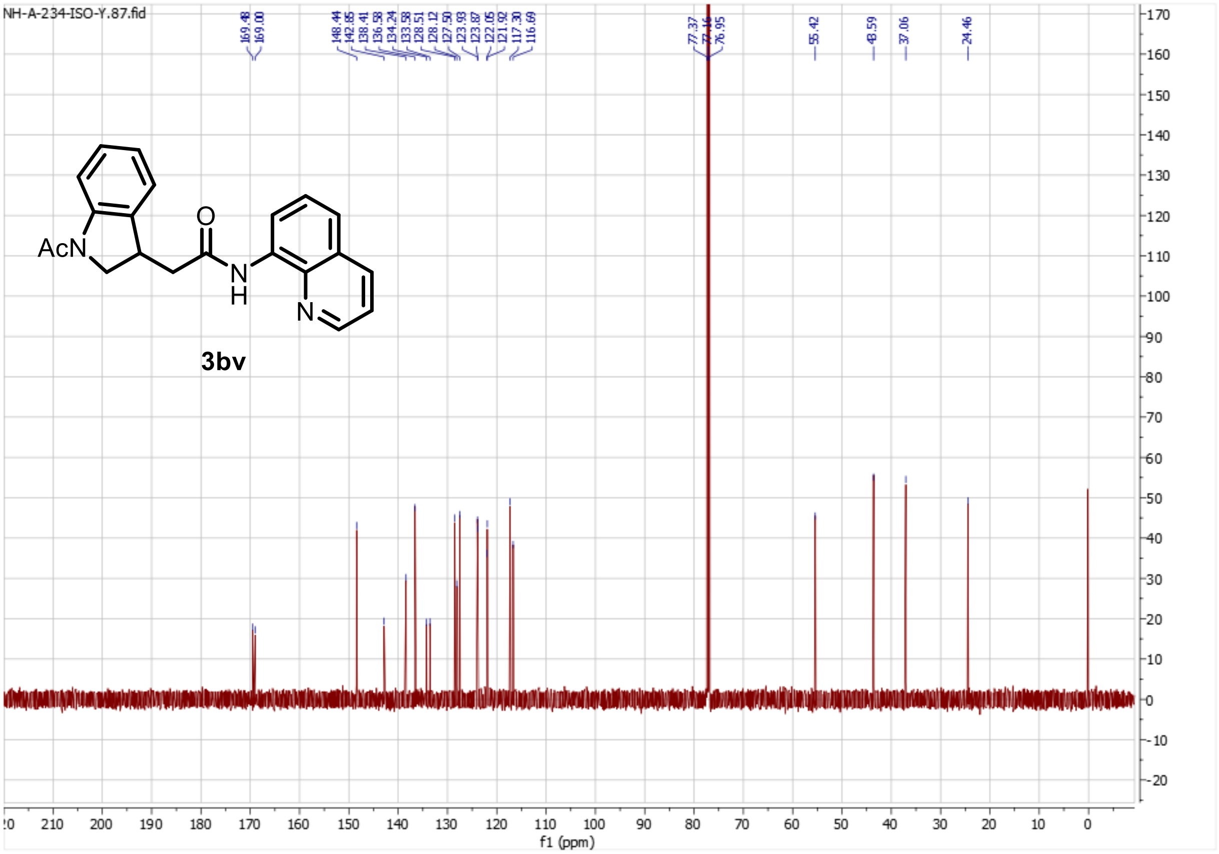

3bv

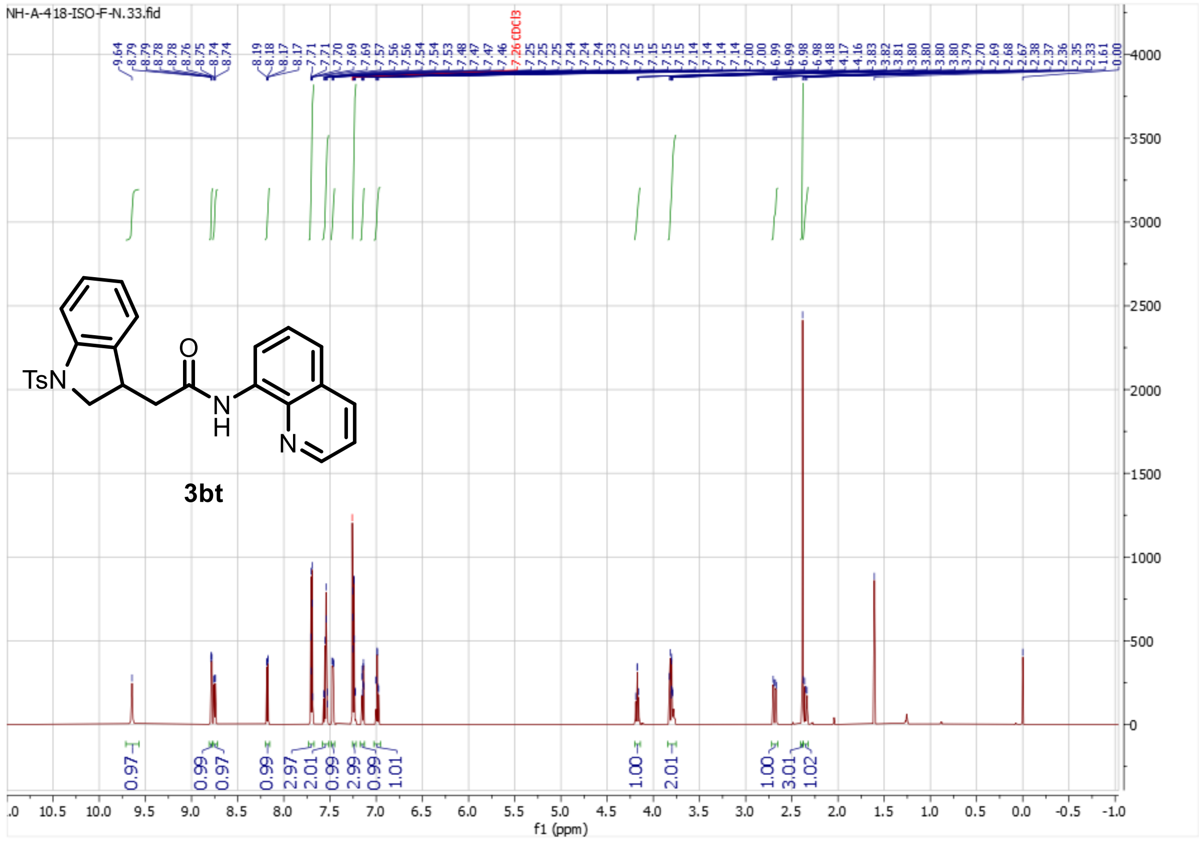
NH-A-418-ISO-F-N.33.fid
TsN
O
N
H
N
3bt
f1 (ppm)

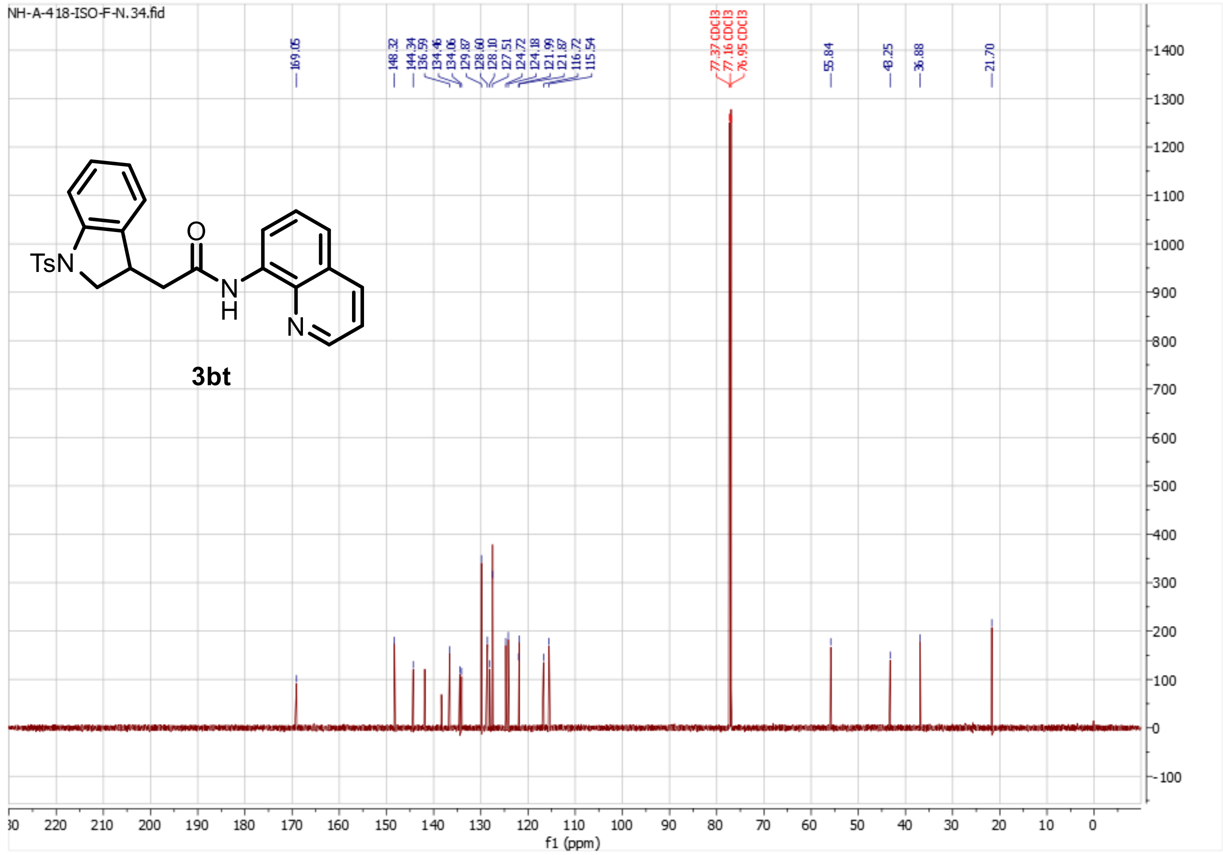
NH-A-418-ISO-F-N.34.fid
TsN
O
N
H
N
3bt
f1 (ppm)

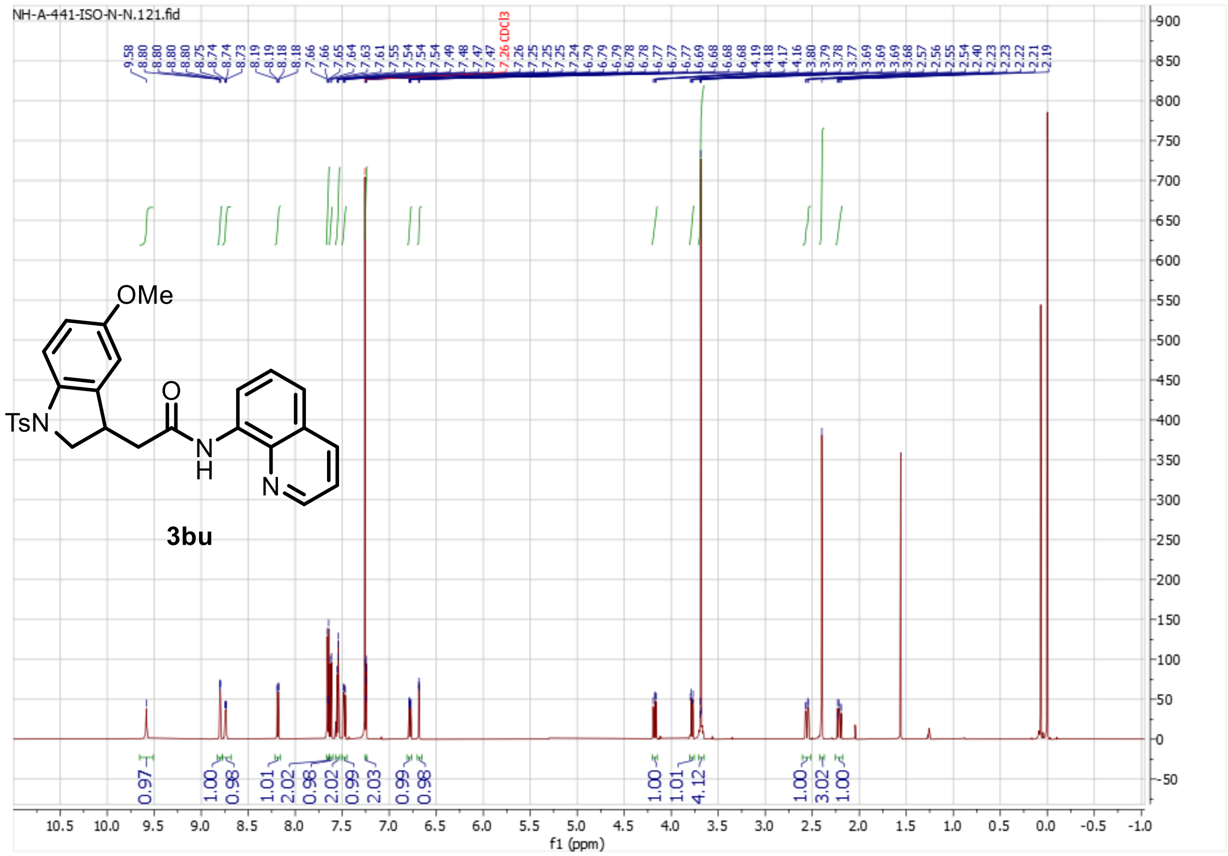
NH-A-441-ISO-N-N.121.fid
OMe
TsN
O
N
H
N
3bu
f1 (ppm)

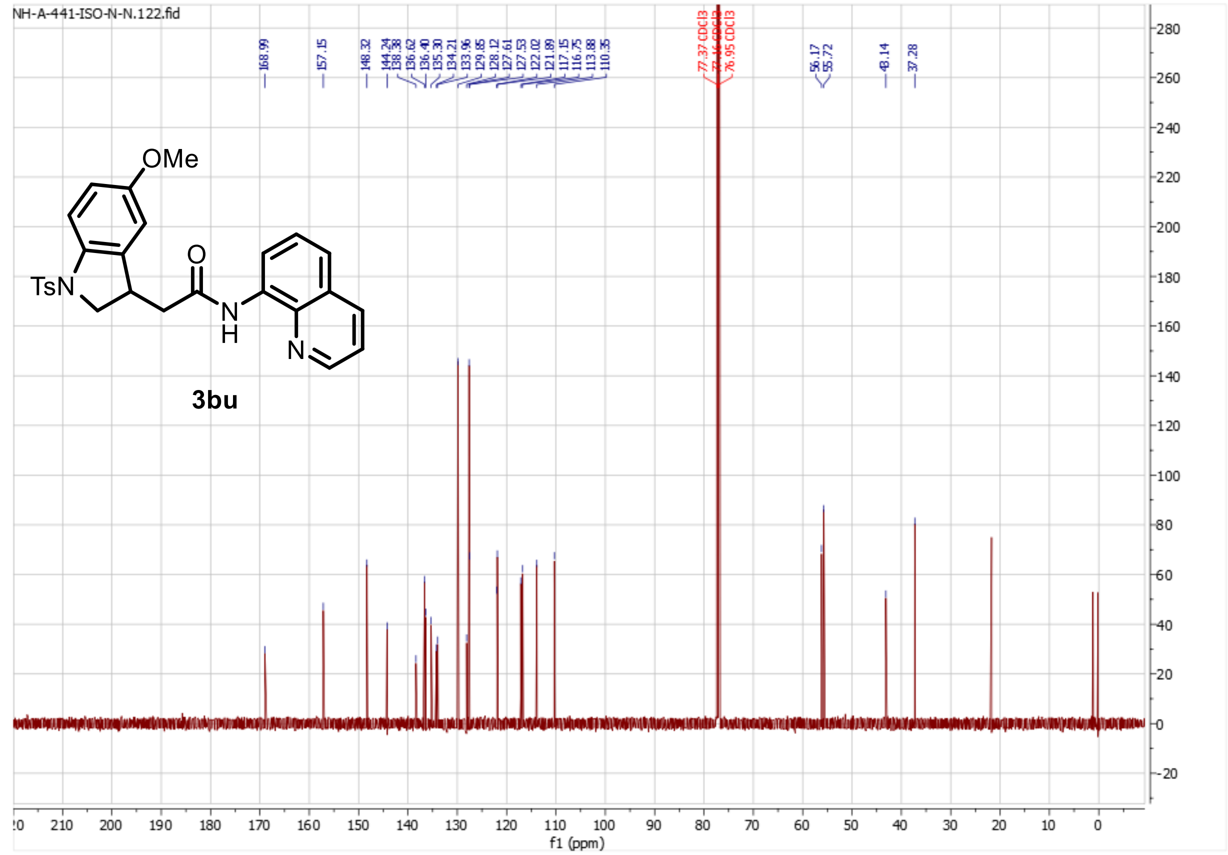
NH-A-441-ISO-N-N.122.fid
168.99
157.15
148.32
144.24
138.38
136.62
136.40
135.30
134.21
133.96
129.85
128.12
127.61
127.53
122.02
121.89
117.15
116.75
113.88
110.35
77.37 CDCl3
76.95 CDCl3
56.17
55.72
43.14
37.28
OMe
TsN
O
N
H
N
3bu
f1 (ppm)

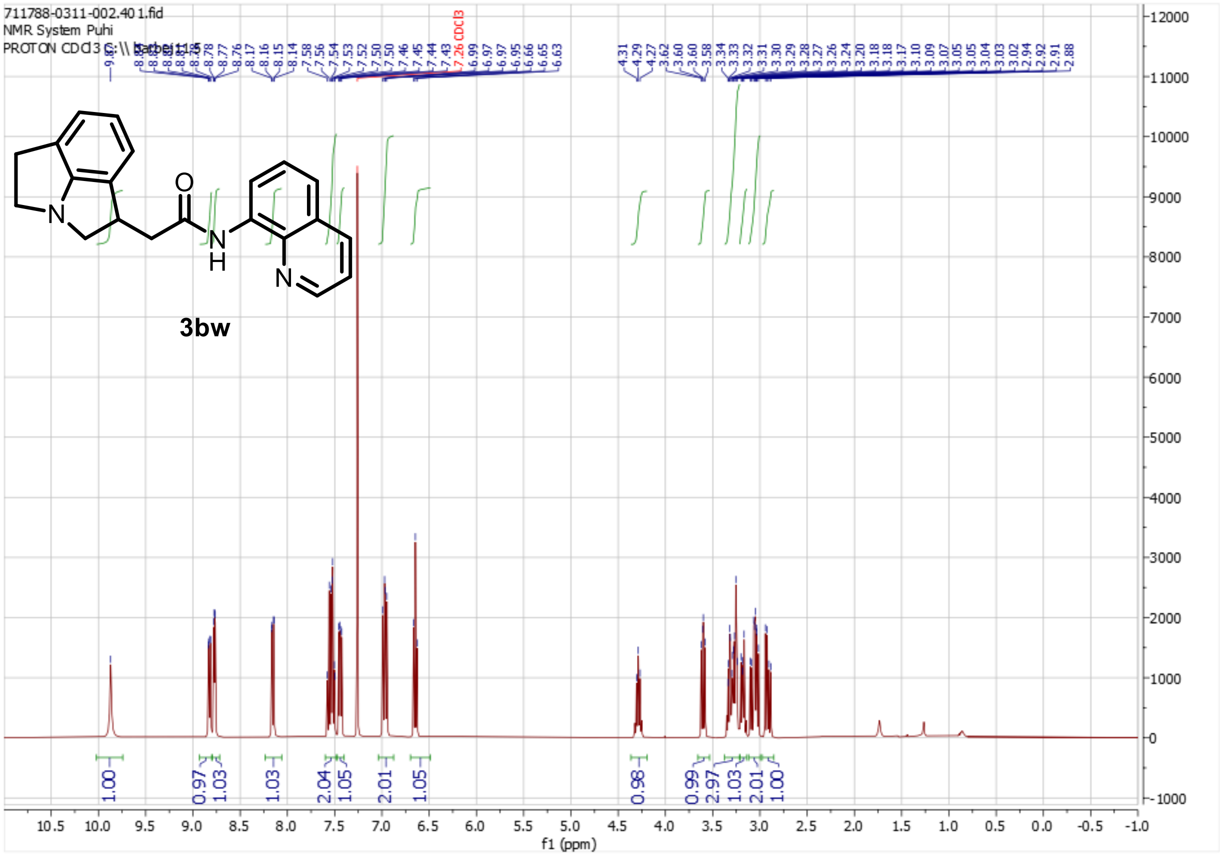
711788-0311-002.40 1.fid
NMR System Puhi
PROTON CDCl3 C:\\ barbej11 5
3bw
f1 (ppm)

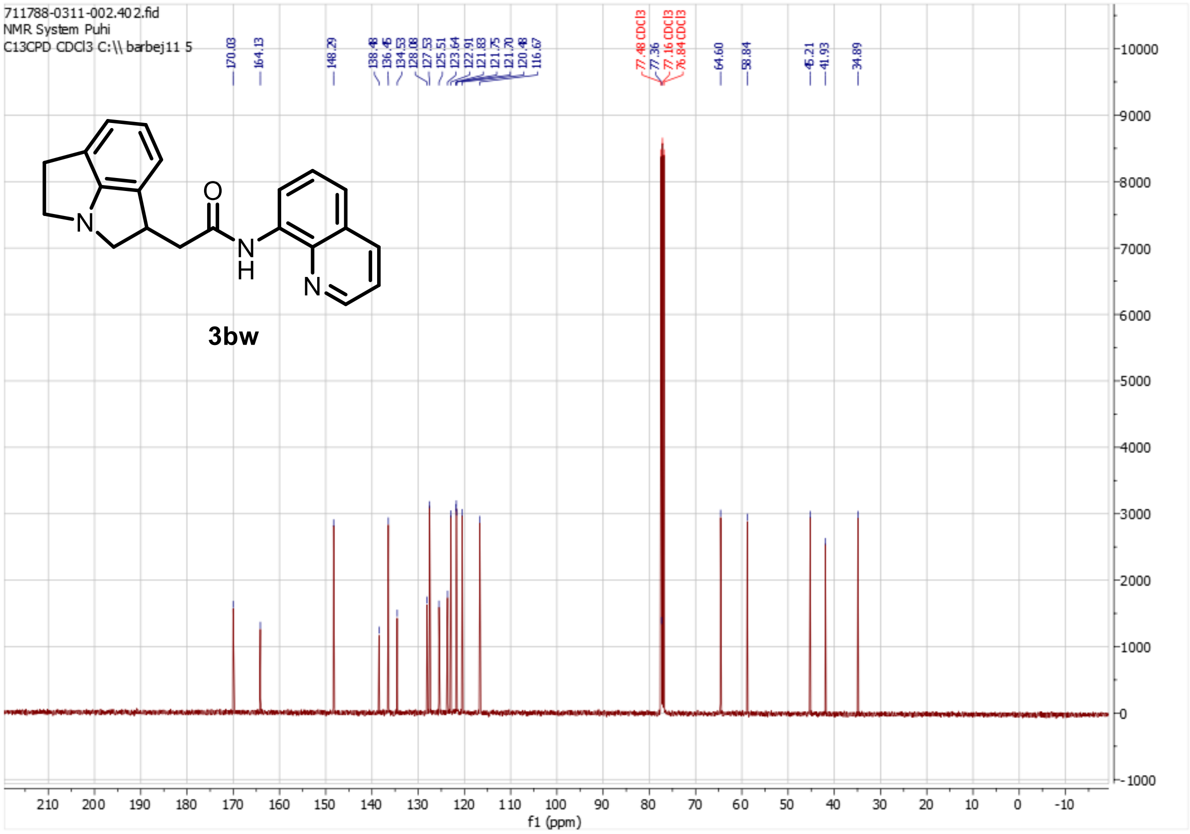
711788-0311-002.40 2.fid
NMR System Puhi
C13CPD CDCl3 C:\\ barbej11 5
3bw
f1 (ppm)

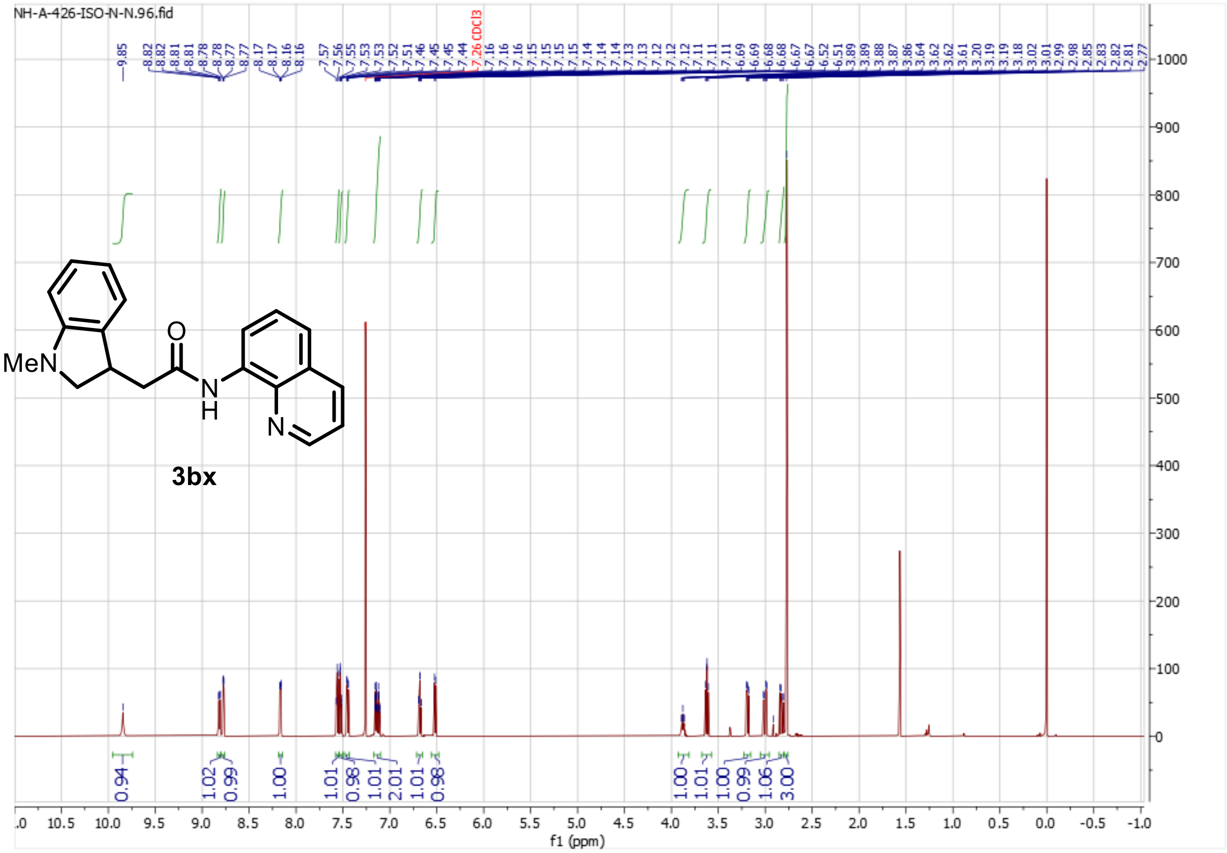
NH-A-426-ISO-N-N.96.fid
9.85
8.82 8.82 8.81 8.81 8.78 8.78 8.77 8.77
8.17 8.17 8.16 8.16
7.57 7.56 7.55 7.53 7.53 7.52 7.51 7.46 7.45 7.45 7.44
7.26 CDCl3
7.16 7.16 7.16 7.15 7.15 7.15 7.15 7.14 7.14 7.13 7.13 7.13 7.12 7.12 7.12 7.11 7.11 7.11
6.69 6.69 6.68 6.68 6.67 6.67 6.52 6.51
3.89 3.89 3.88 3.87 3.86 3.64 3.62 3.62 3.61 3.20 3.19 3.19 3.18 3.02 3.01 2.99 2.98 2.85 2.83 2.82 2.81 2.77
0.94 1.02 0.99 1.00 1.01 0.98 1.01 2.01 1.01 0.98 1.00 1.01 1.00 0.99 1.06 3.00
O
MeN
N
H
N
3bx
f1 (ppm)

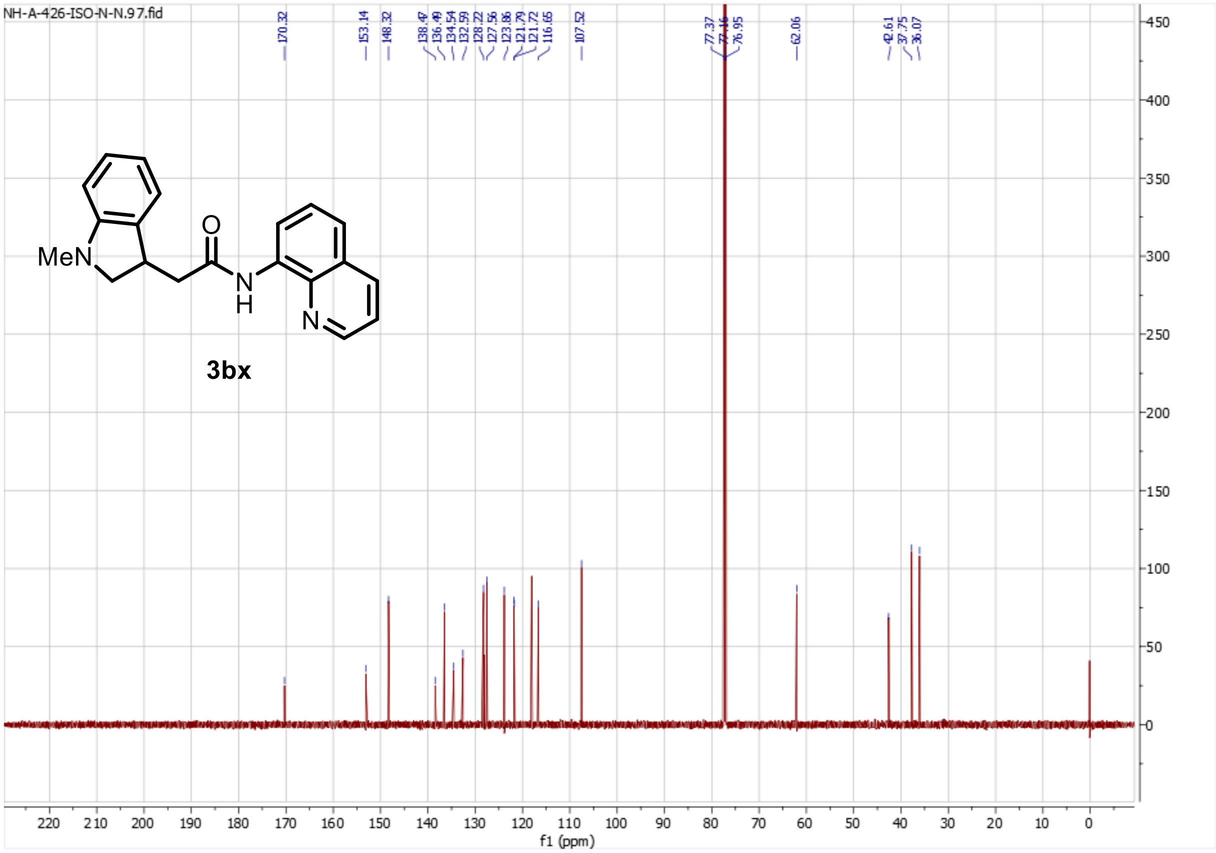
NH-A-426-ISO-N-N.97.fid
170.32
153.14
148.32
138.47 136.49 134.54 132.59 128.22 127.56 123.86 121.79 121.72 116.65
107.52
77.37 77.16 76.95
62.06
42.61 37.75 36.07
O
MeN
N
H
N
3bx
f1 (ppm)

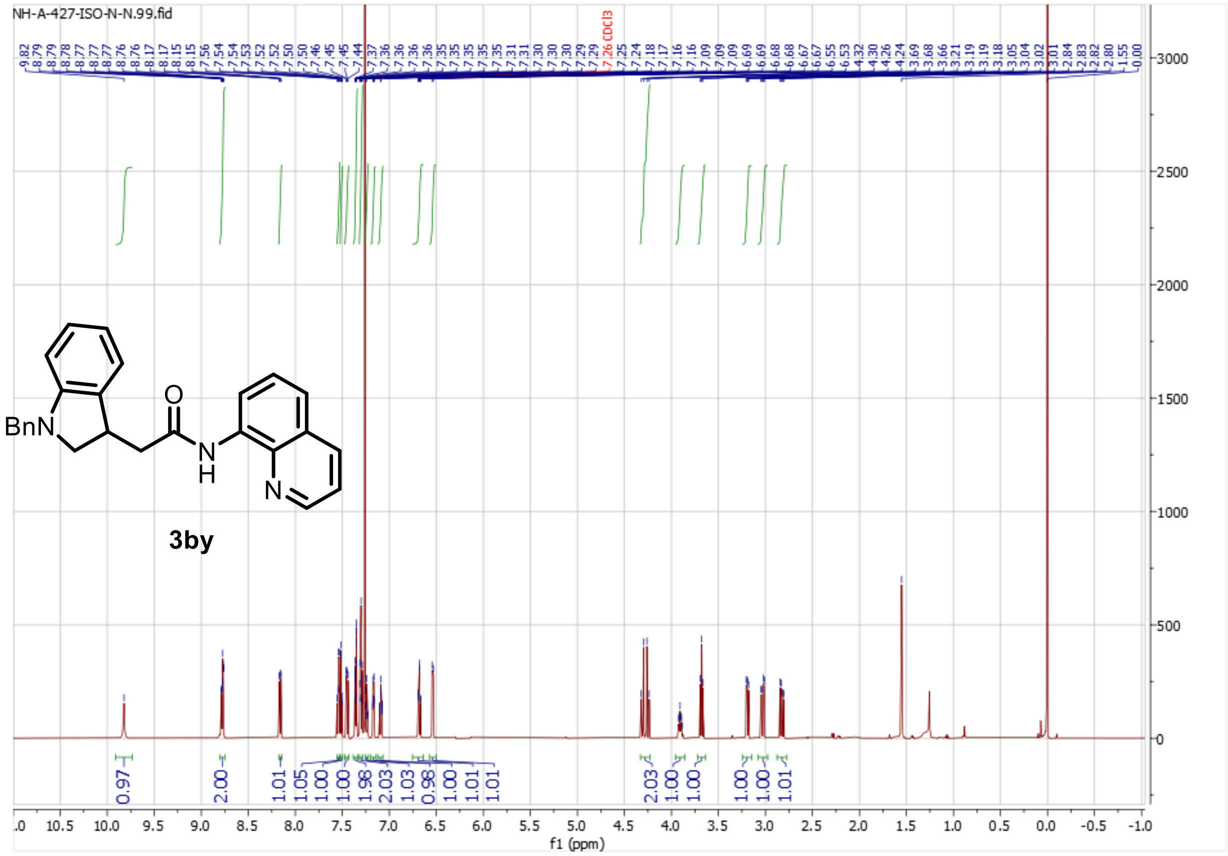
NH-A-427-ISO-N-N.99.fid
7.26 CDCl3
BnN
O
N
H
N
3by
0.97
2.00
1.01
1.05
1.00
1.00
1.98
2.03
1.03
0.98
1.00
1.01
1.01
2.03
1.00
1.00
1.00
1.00
1.01
f1 (ppm)

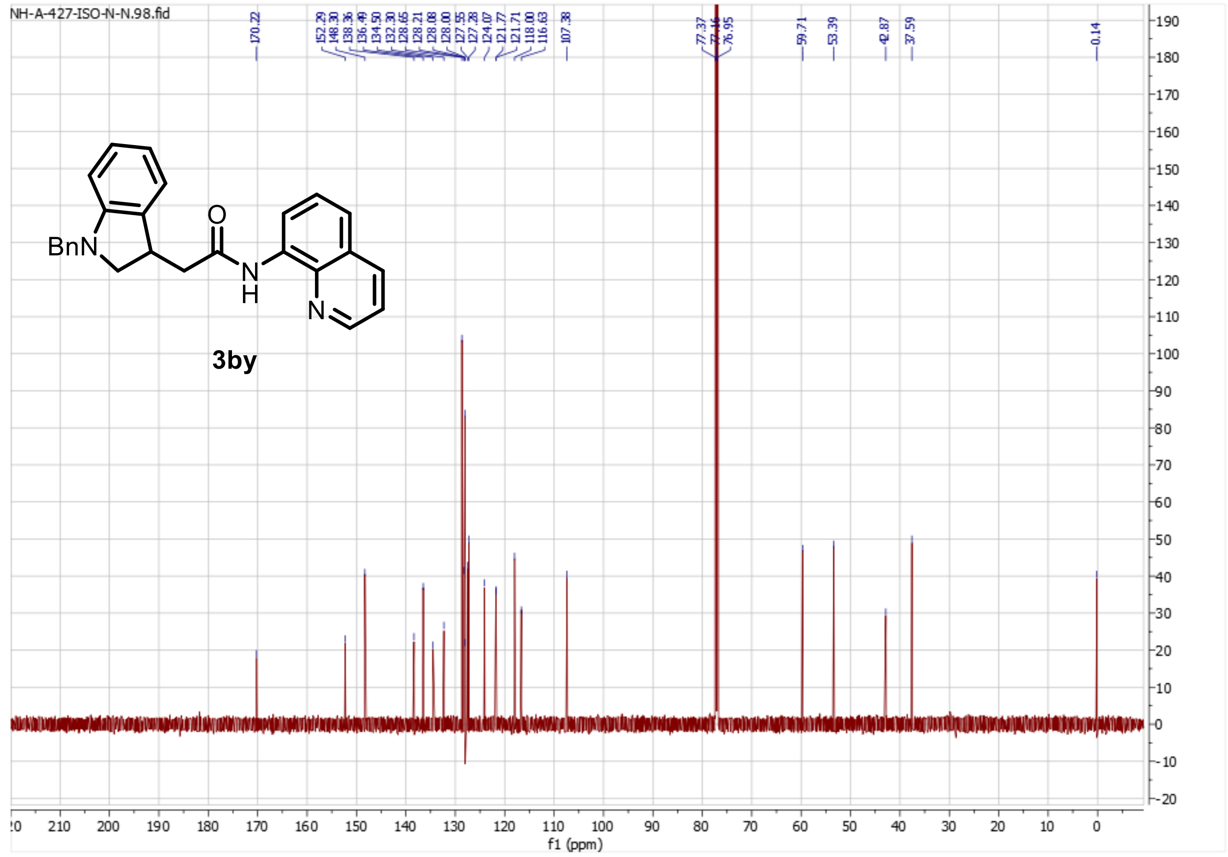
NH-A-427-ISO-N-N.98.fid
170.22
107.38
59.71
53.39
42.87
37.59
0.14
BnN
O
N
H
N
3by
f1 (ppm)

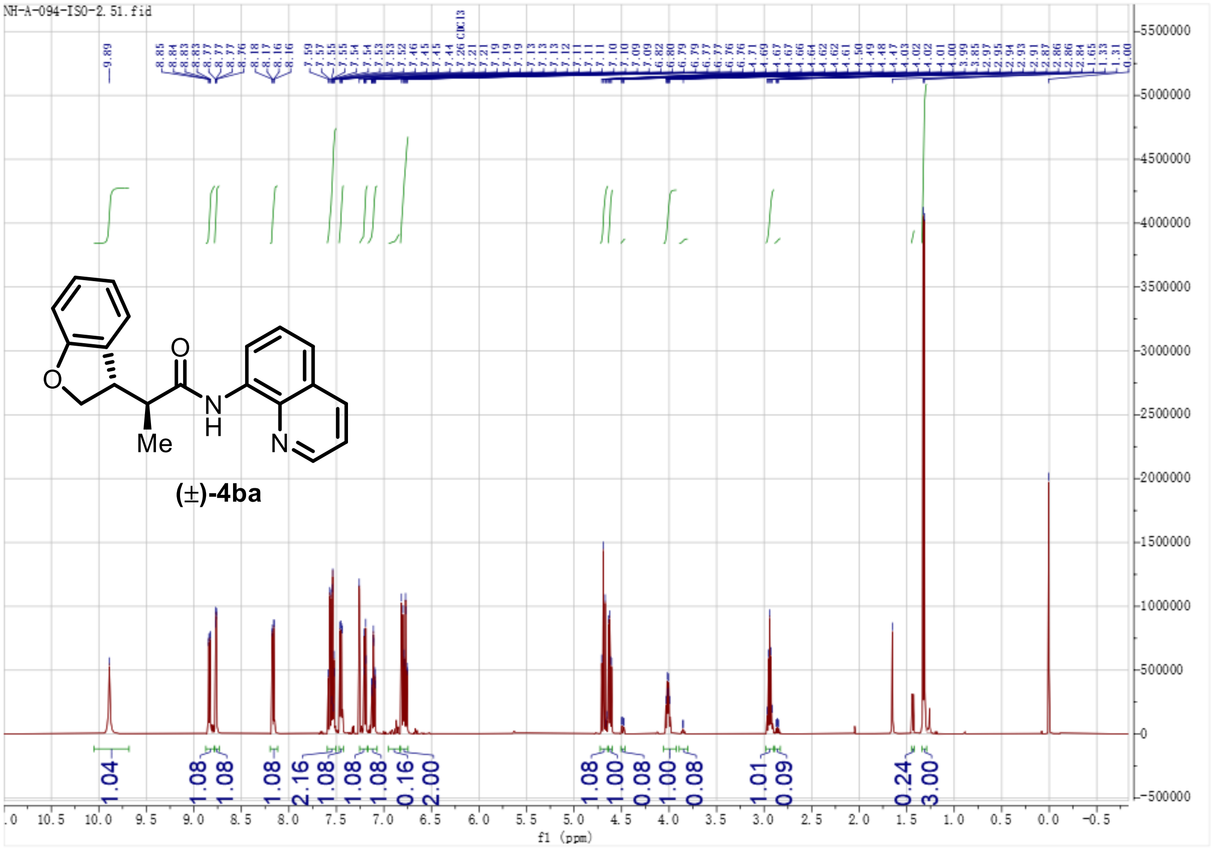
NH-A-094-ISO-2.51.fid
(±)-4ba
Me
f1 (ppm)

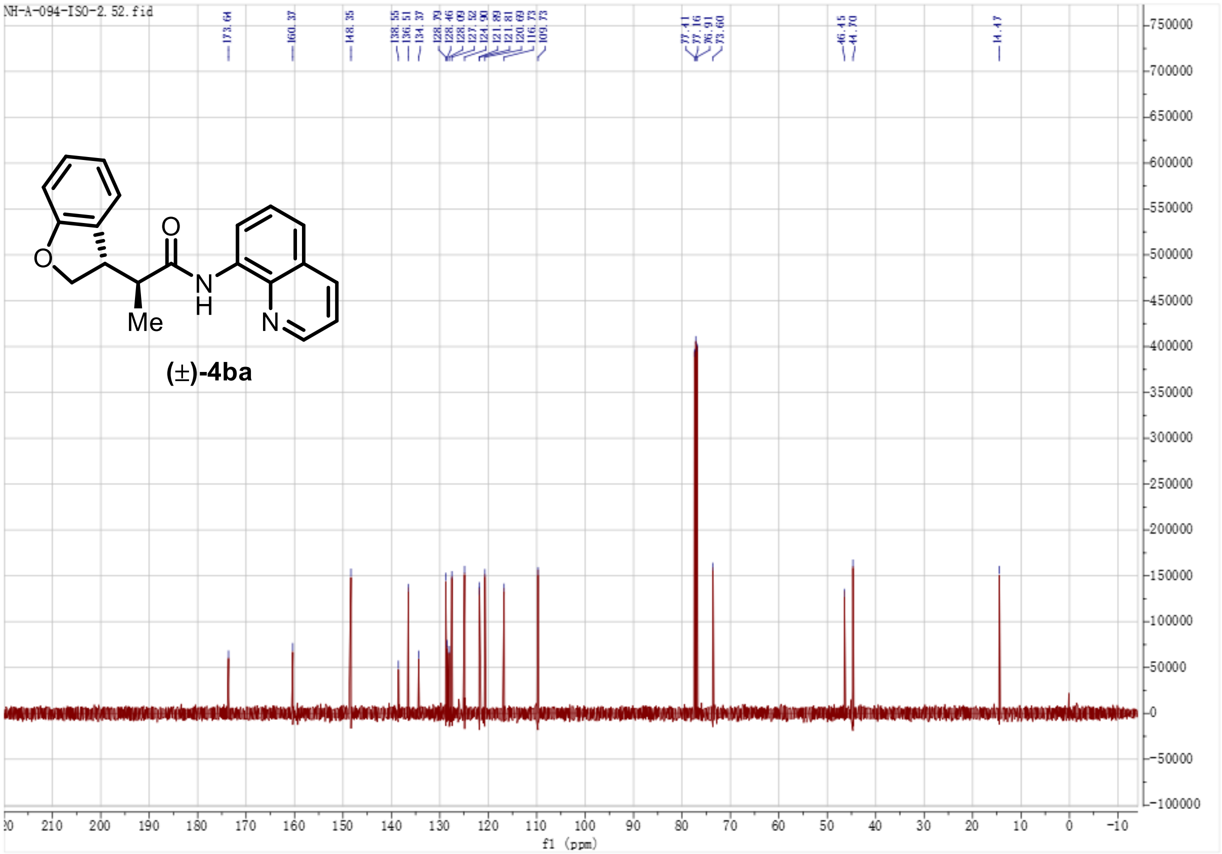
NH-A-094-ISO-2.52.fid
173.64
160.37
148.35
138.55
136.51
134.37
128.79
128.46
128.09
127.52
124.90
121.89
121.81
120.69
116.73
109.73
77.41
77.16
76.91
73.60
46.45
44.70
14.47
(±)-4ba
f1 (ppm)

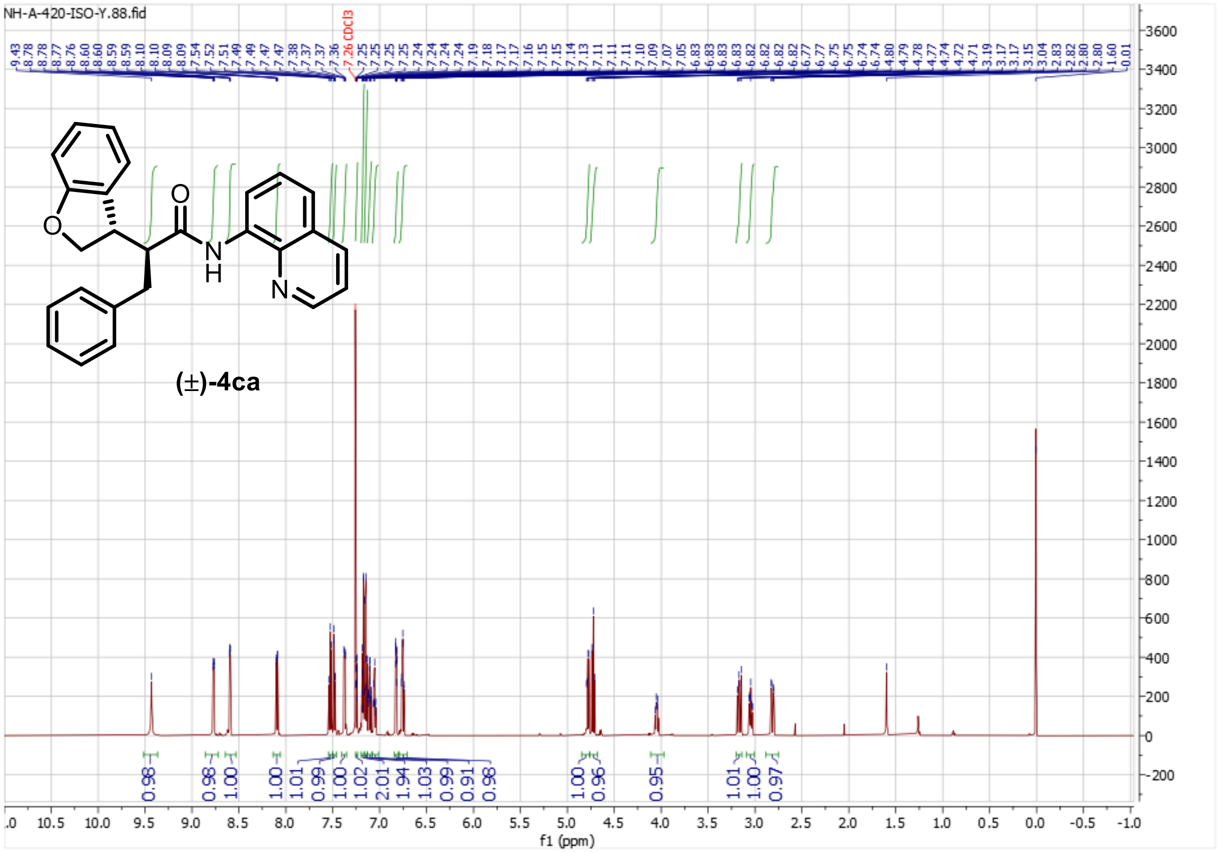
NH-A-420-ISO-Y.88.fid
7.26 CDCl3
(±)-4ca
f1 (ppm)

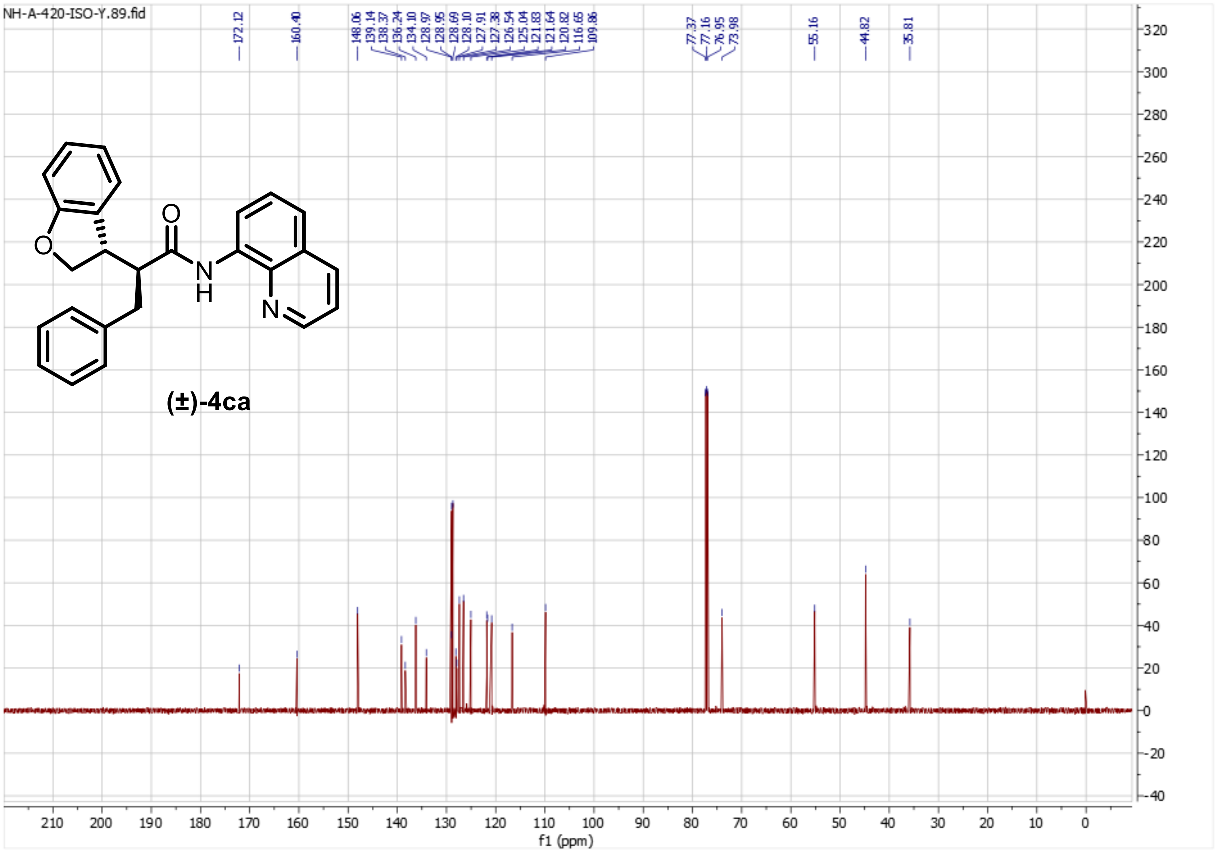

(±)-4ca

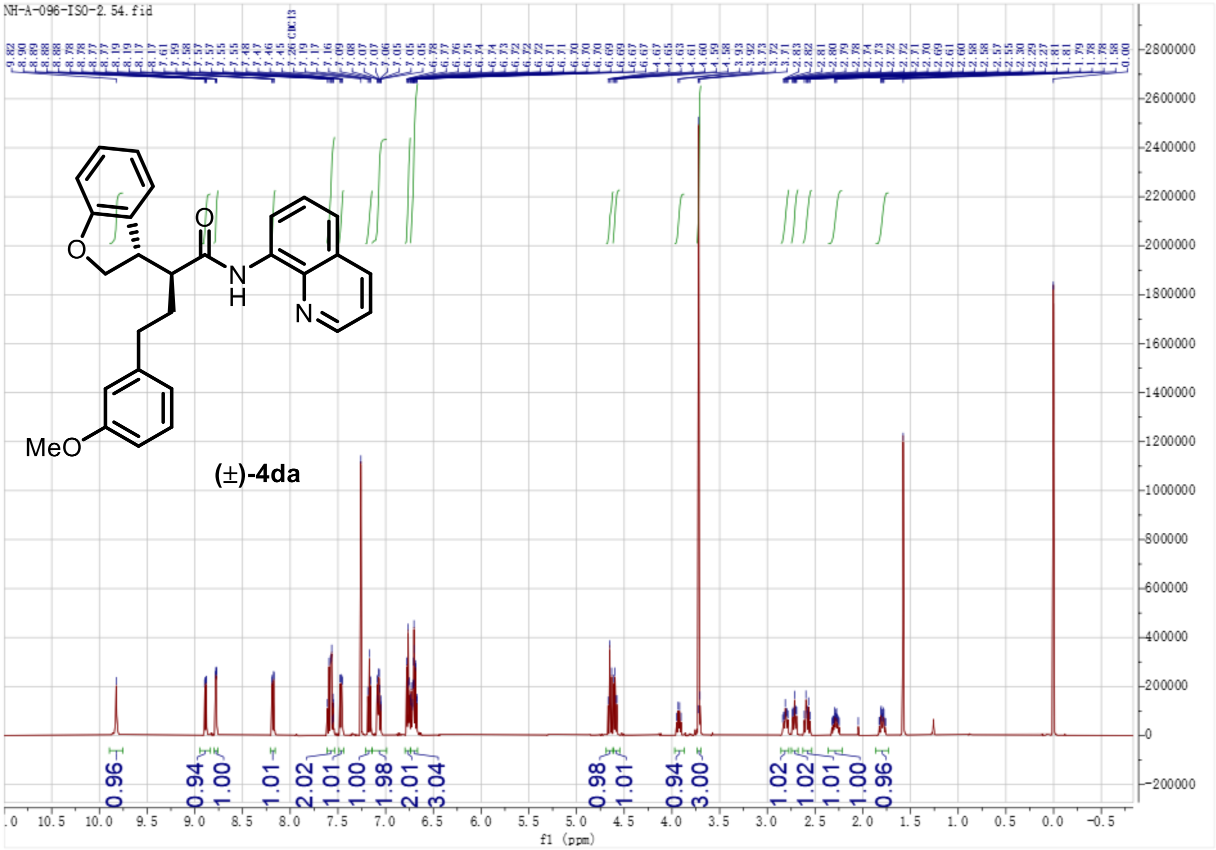

(±)-4da

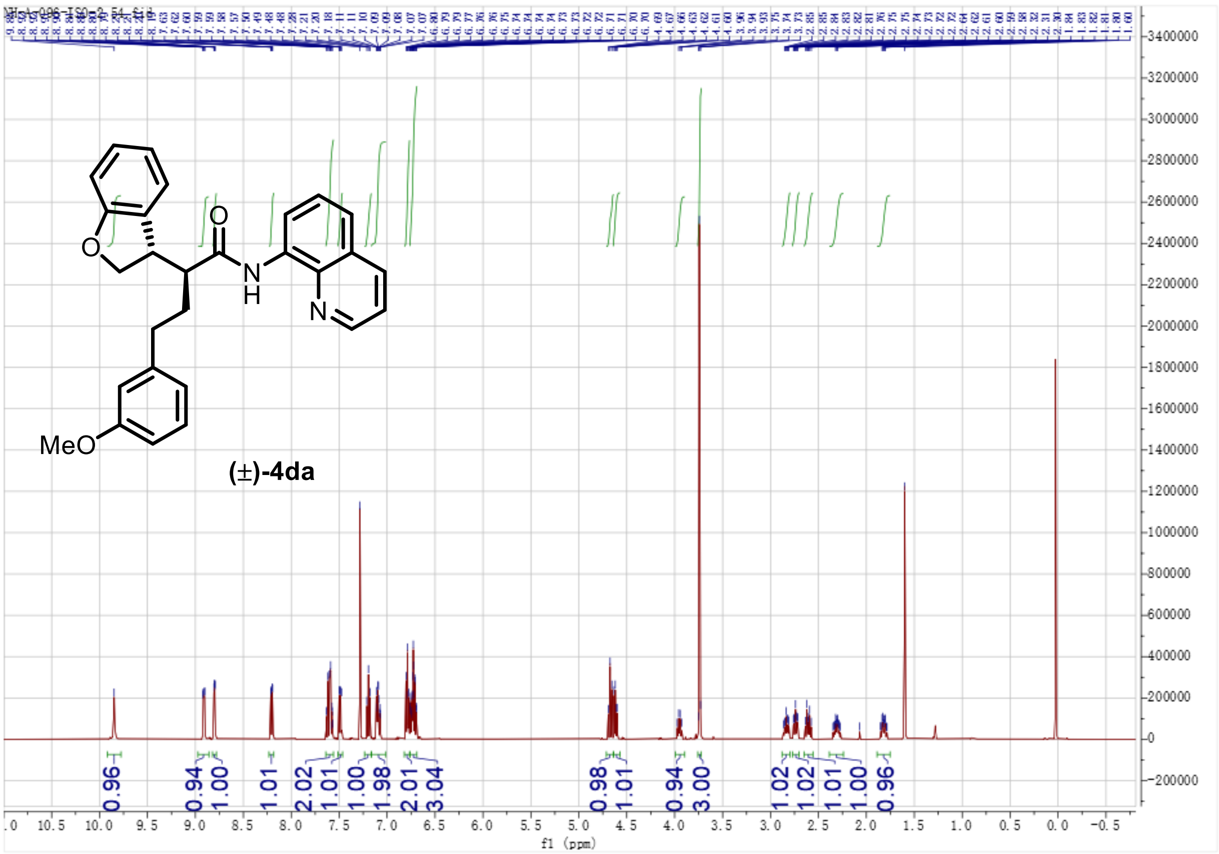
MeO
(±)-4da
f1 (ppm)

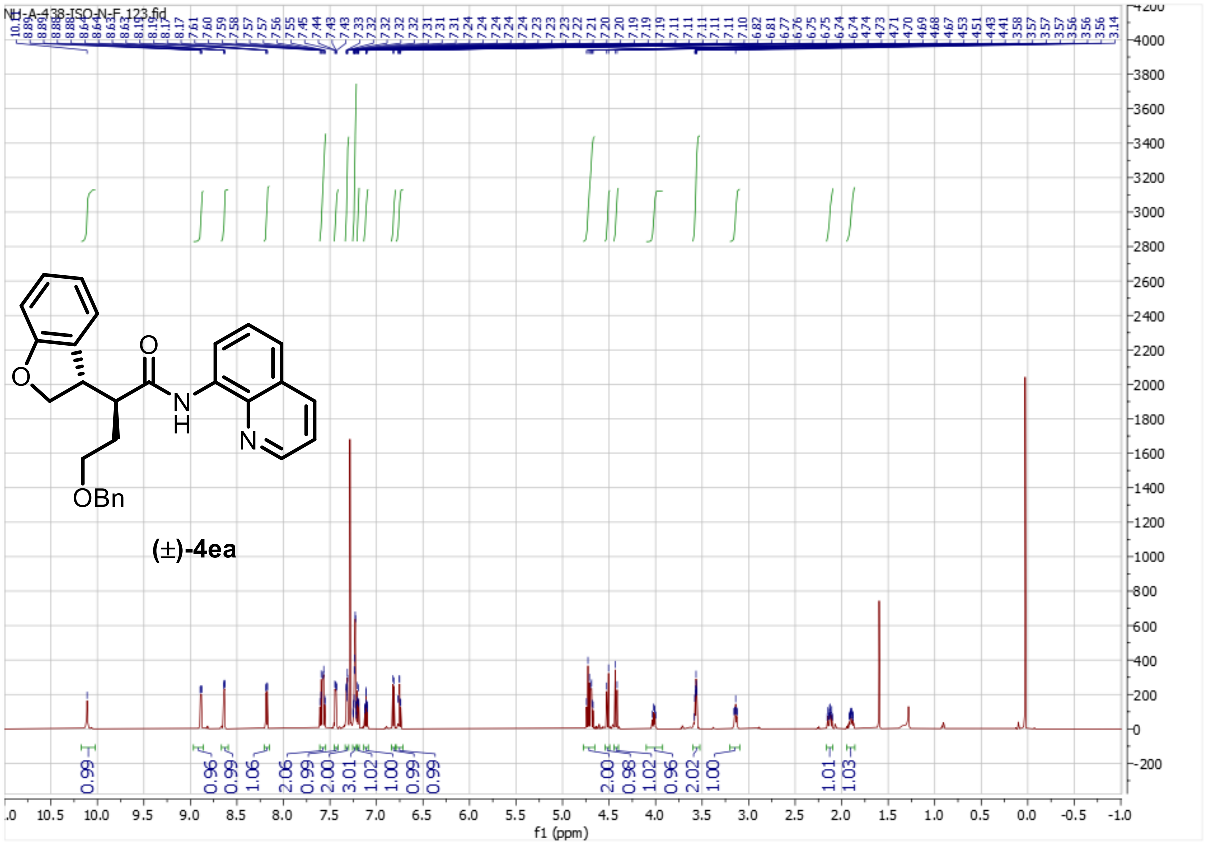
OBn
(±)-4ea
f1 (ppm)

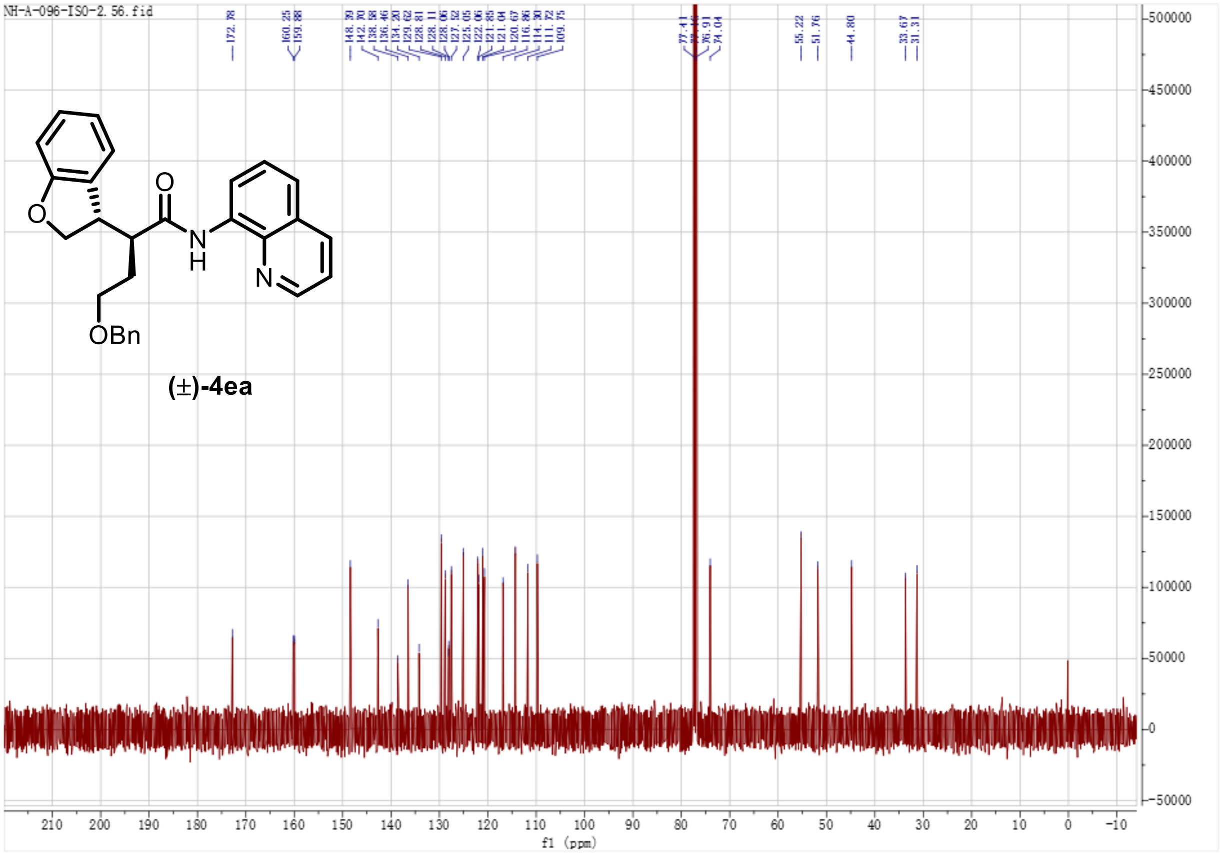

NH-A-096-ISO-2.56.fid
OBn
(±)-4ea
f1 (ppm)

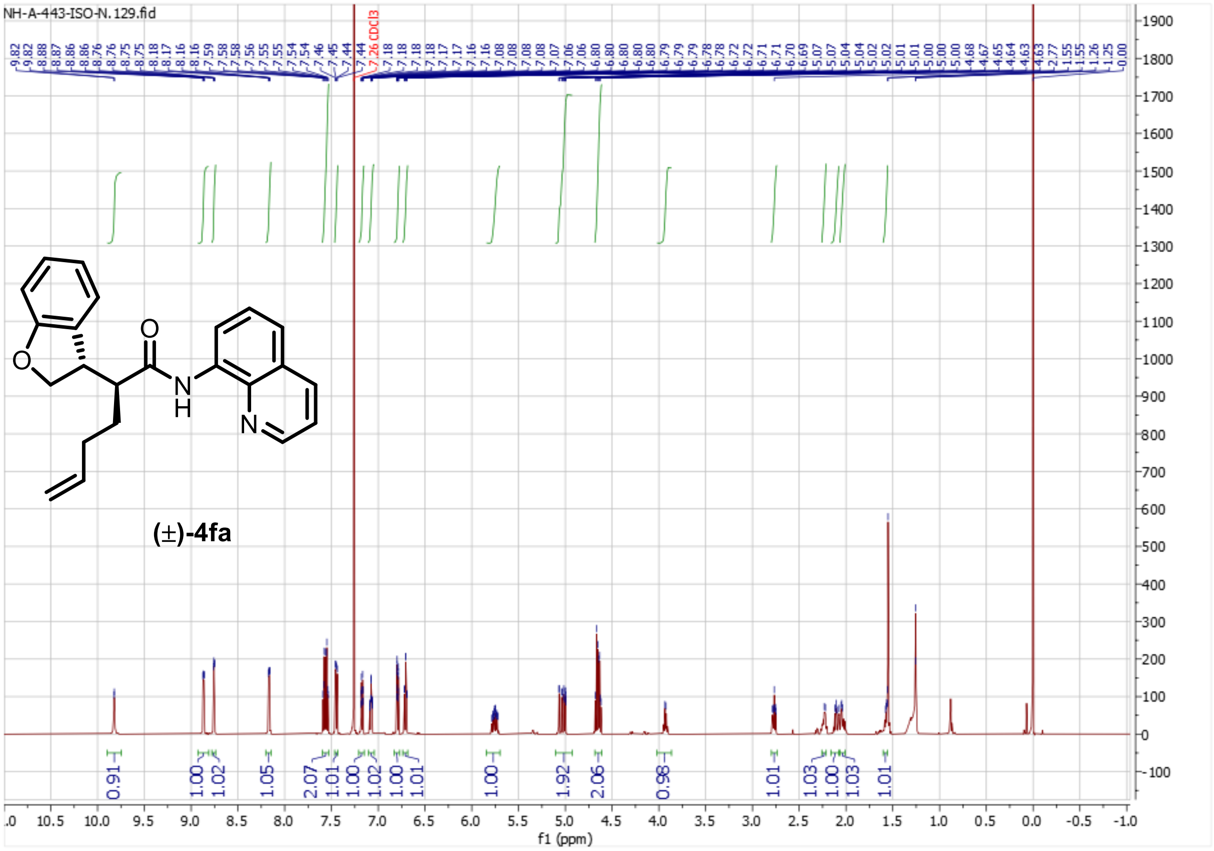

NH-A-443-ISO-N.129.fid
7.26 CDCl3
(±)-4fa
f1 (ppm)

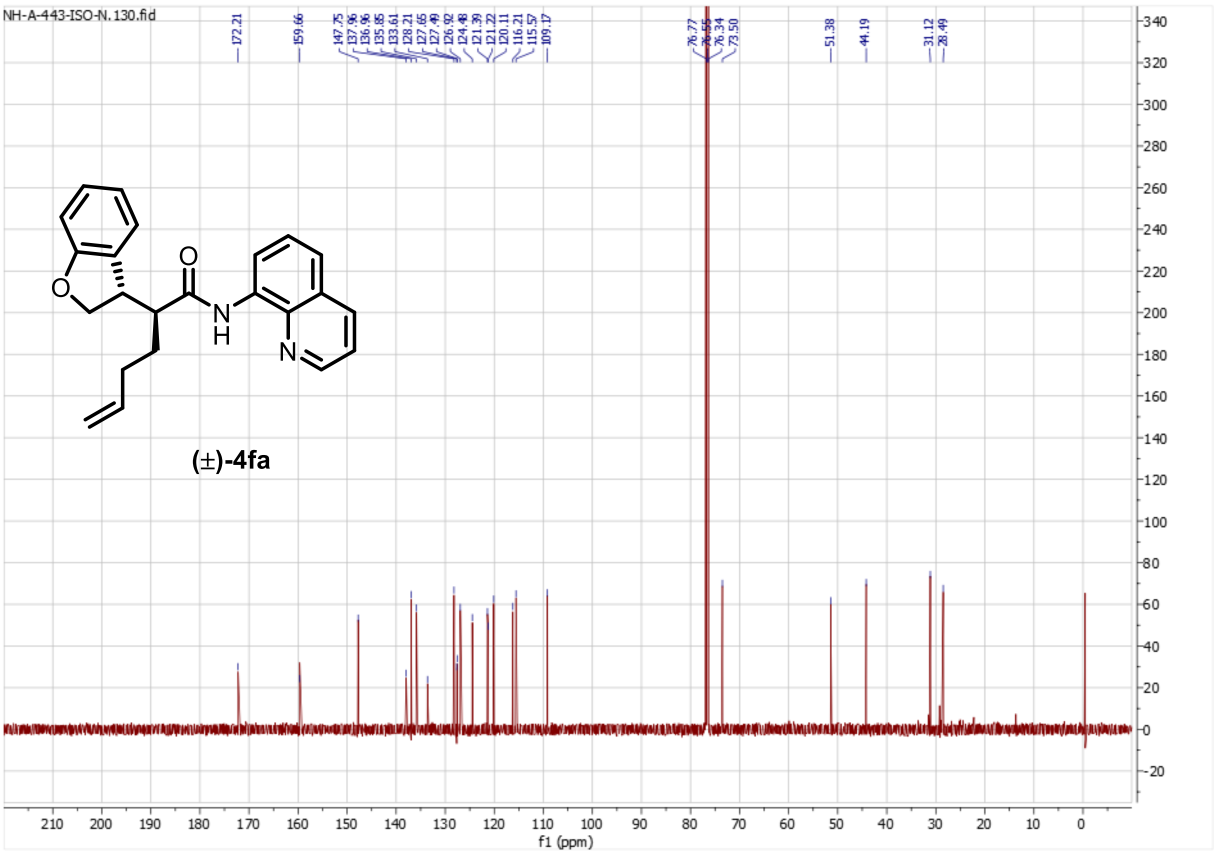
NH-A-443-ISO-N.130.fid
O
N
H
N
f1 (ppm)

(±)-4fa

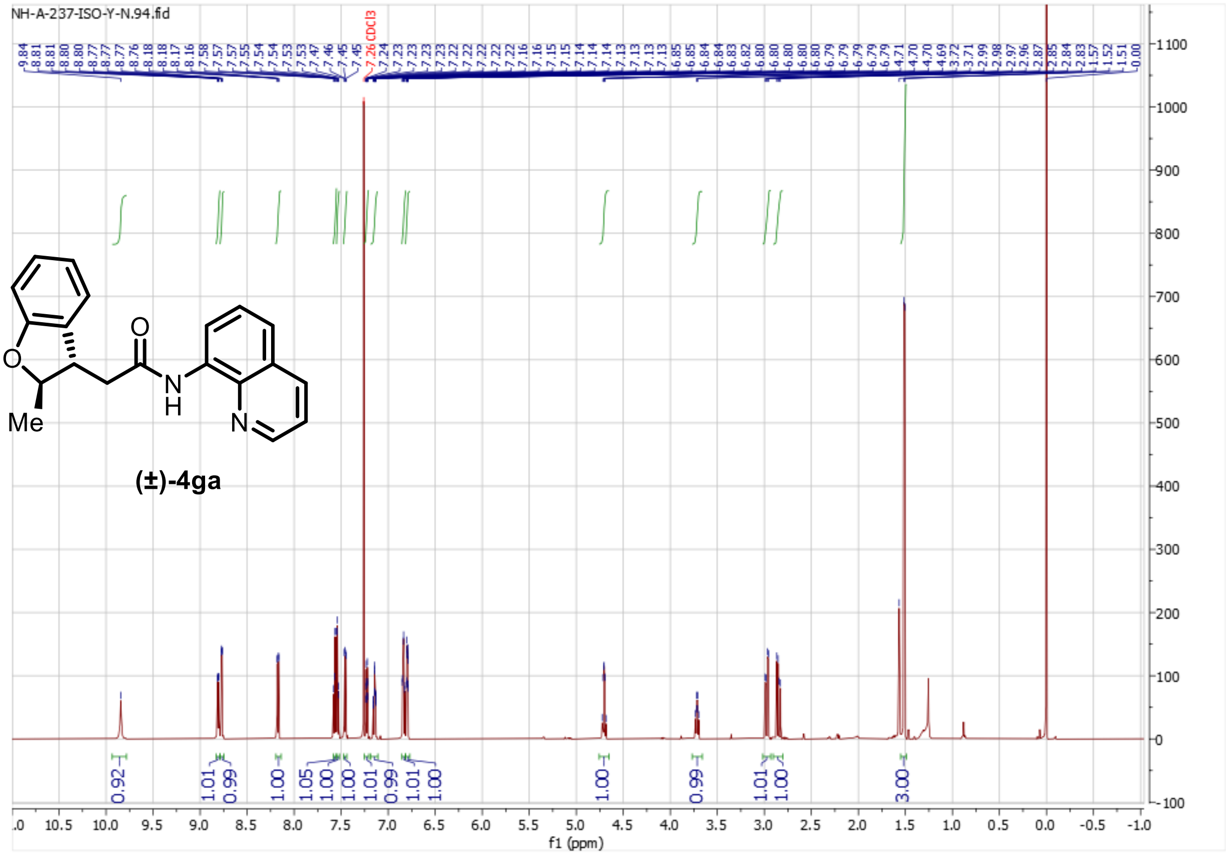
NH-A-237-ISO-Y-N.94.fid
7.26 CDCl3
O
O
N
H
N
Me
f1 (ppm)

(±)-4ga

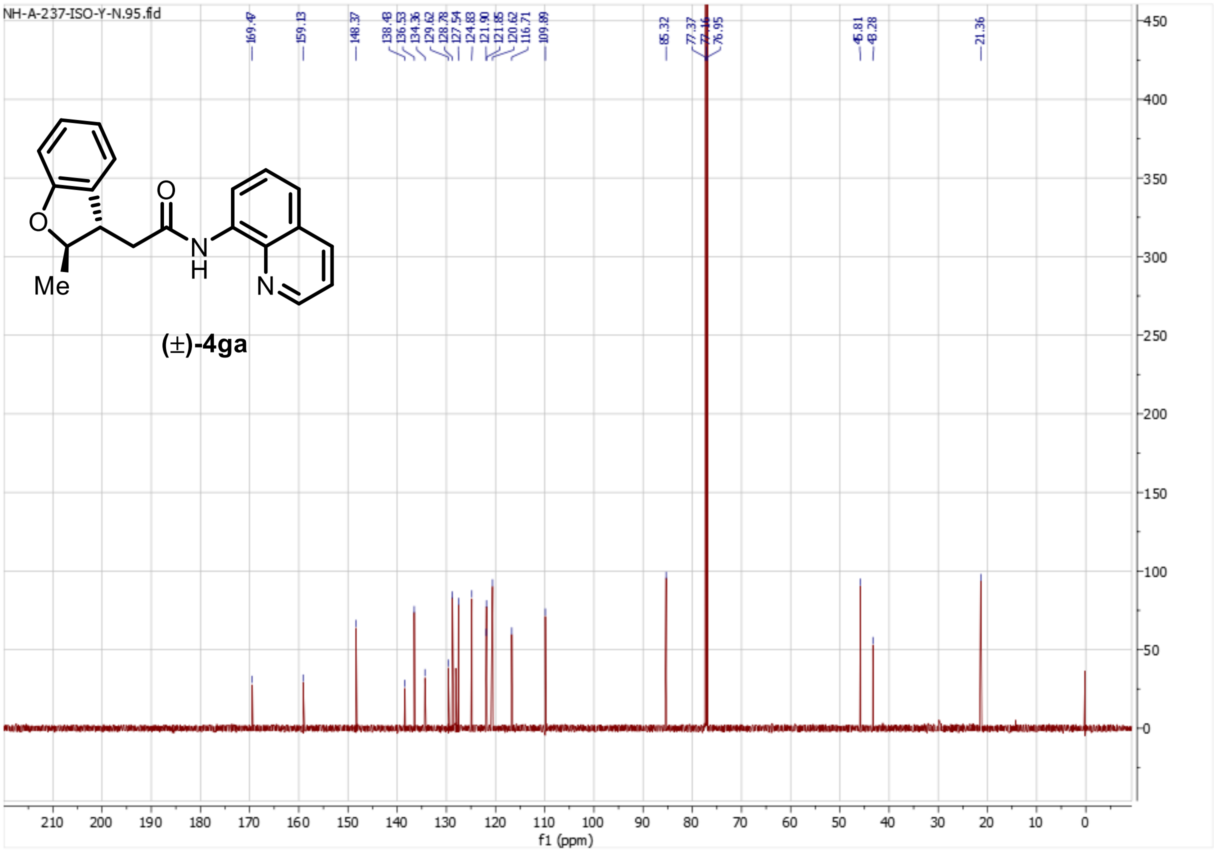
NH-A-237-ISO-Y-N.95.fid
f1 (ppm)

(±)-4ga

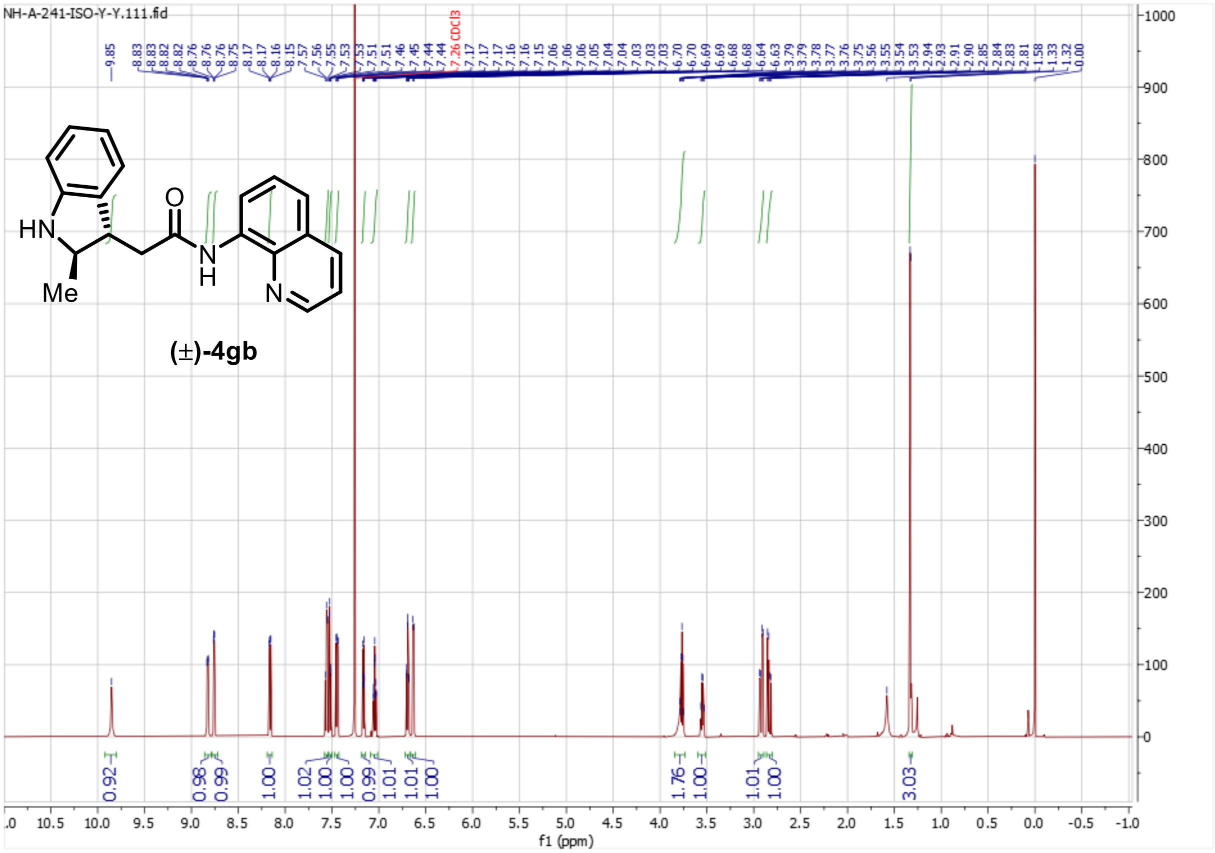
NH-A-241-ISO-Y-Y.111.fid
7.26 CDCl3
f1 (ppm)

(±)-4gb

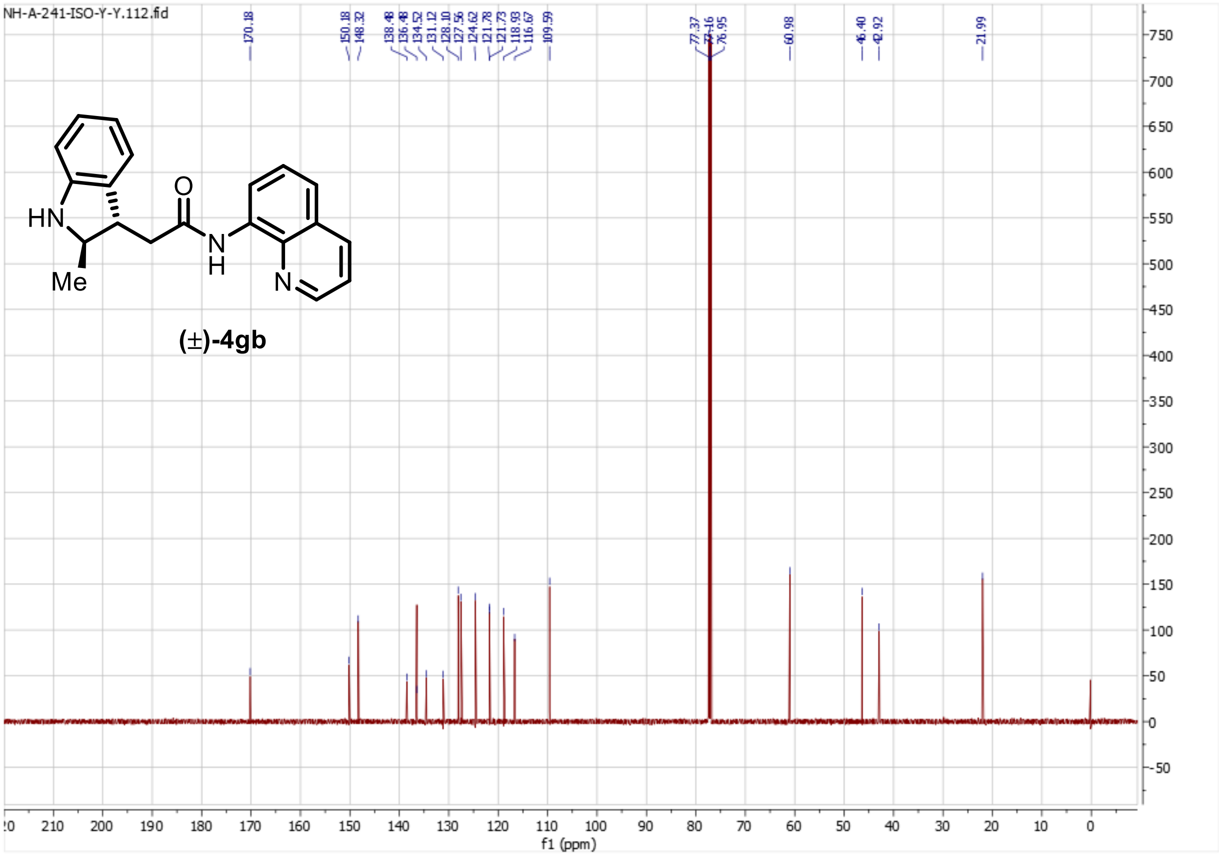
NH-A-241-ISO-Y-Y.112.fid
HN
Me
O
N
H
N
(±)-4gb
f1 (ppm)

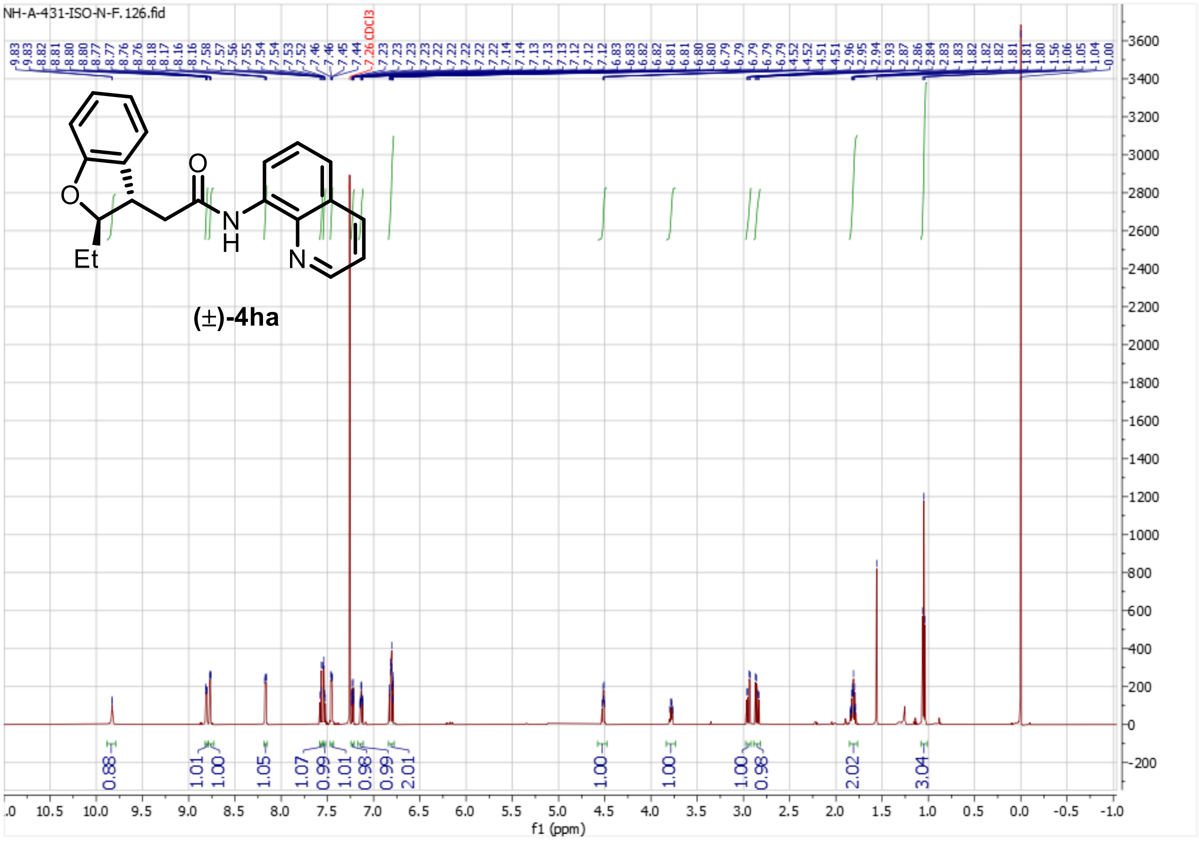
NH-A-431-ISO-N-F.126.fid
7.26 CDCl3
O
Et
O
N
H
N
(±)-4ha
f1 (ppm)

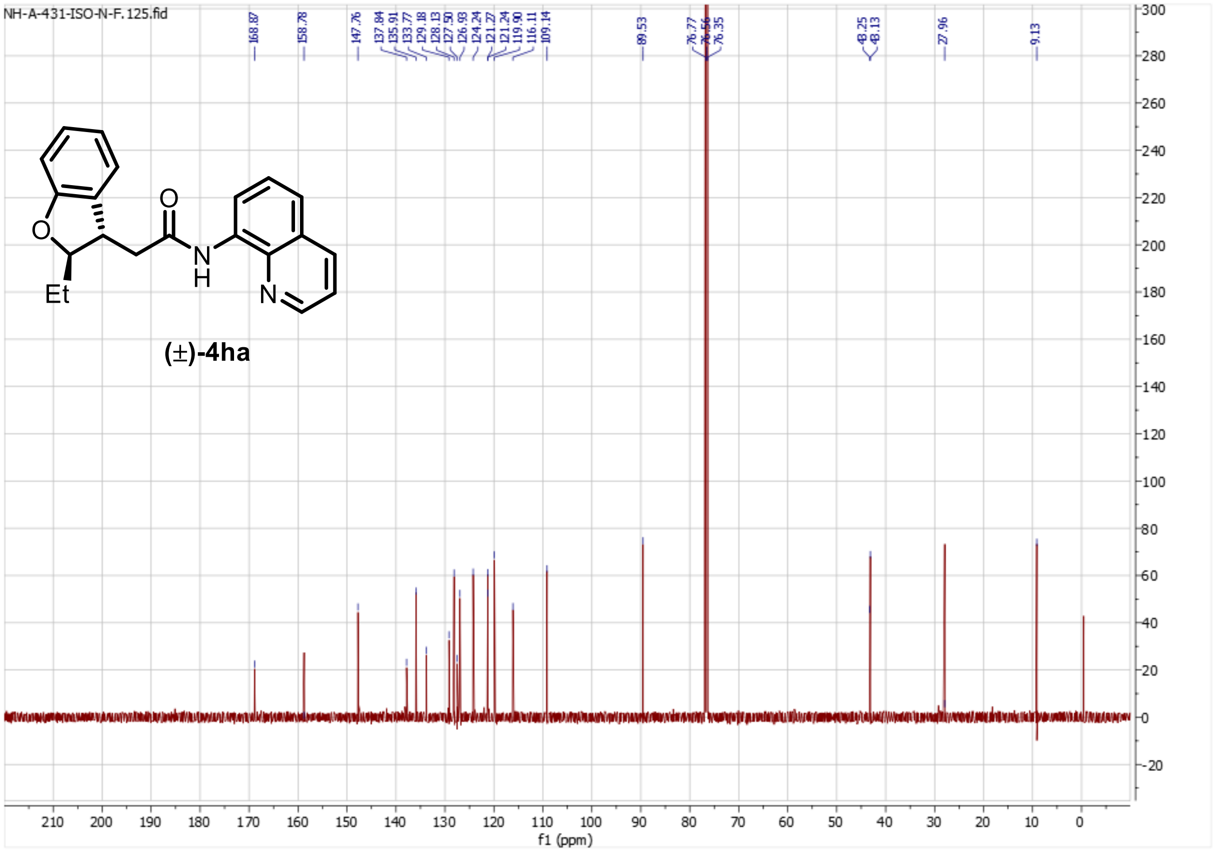
NH-A-431-ISO-N-F.125.fid
f1 (ppm)

(±)-4ha

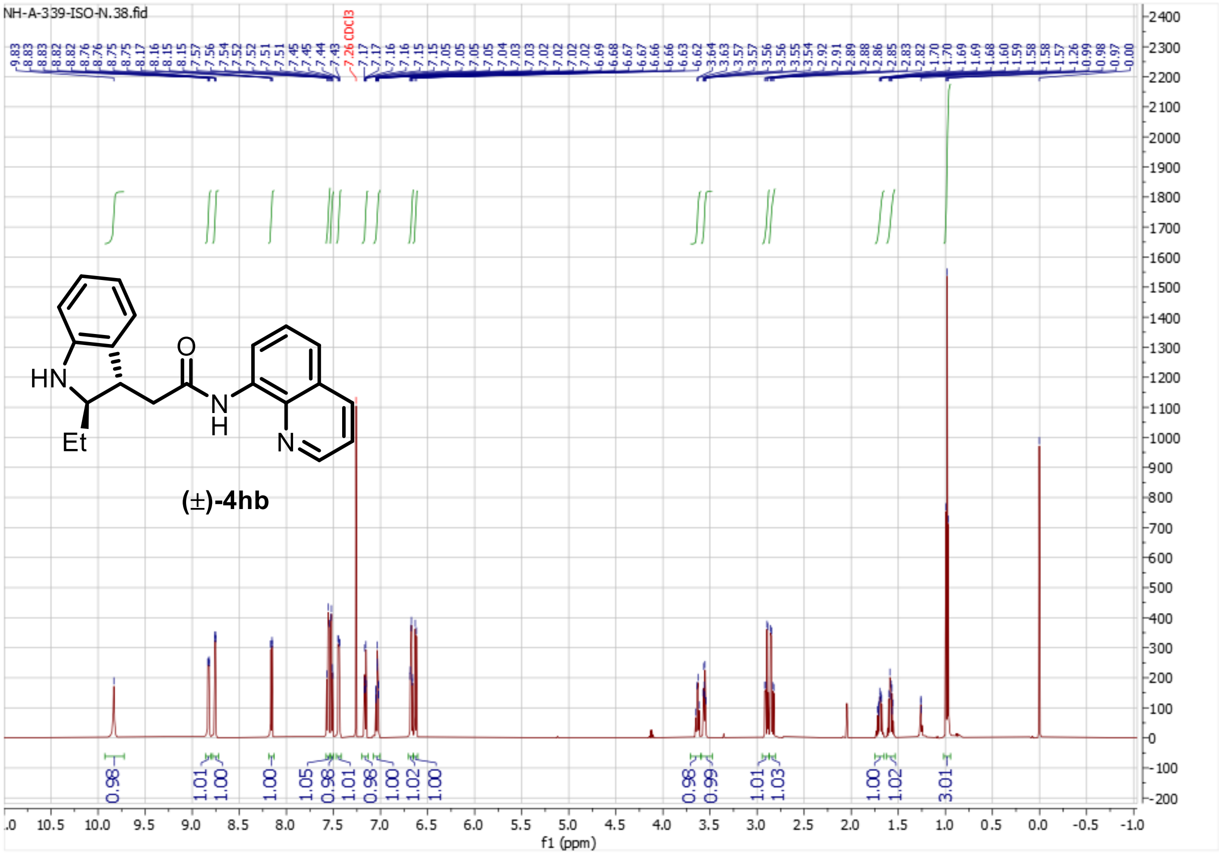
NH-A-339-ISO-N.38.fid
7.26 CDCl3
f1 (ppm)

(±)-4hb

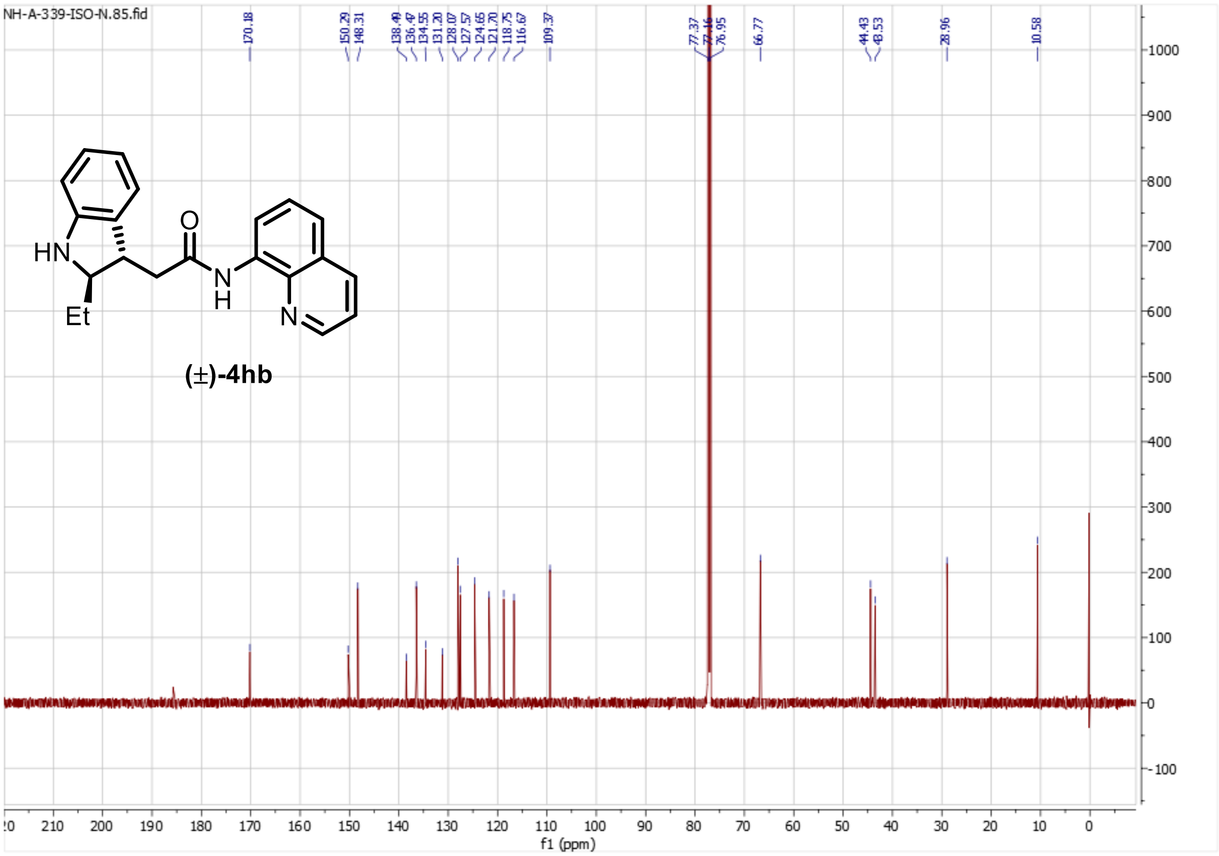
NH-A-339-ISO-N.85.fid
HN
Et
O
N
H
N
f1 (ppm)

(±)-4hb

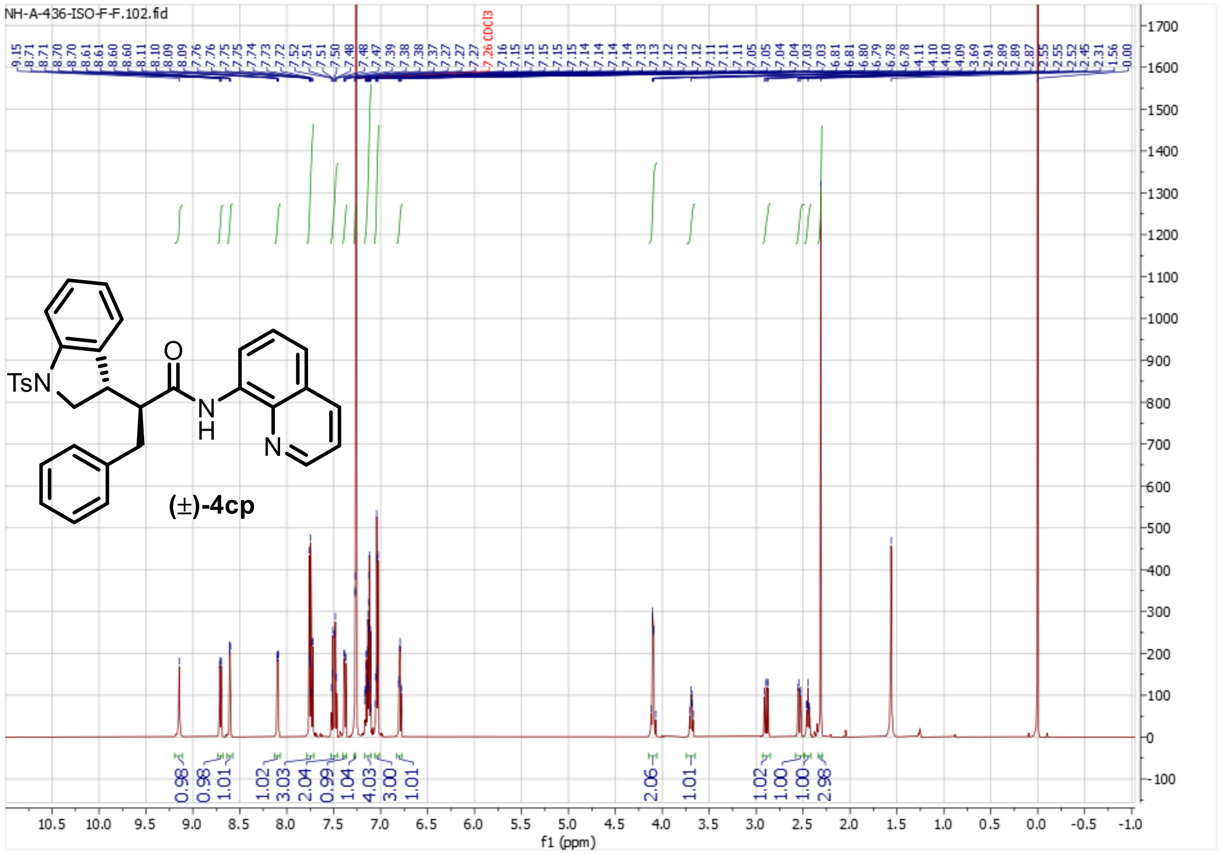
NH-A-436-ISO-F-F.102.fid
TsN
O
N
H
N
f1 (ppm)

(±)-4cp

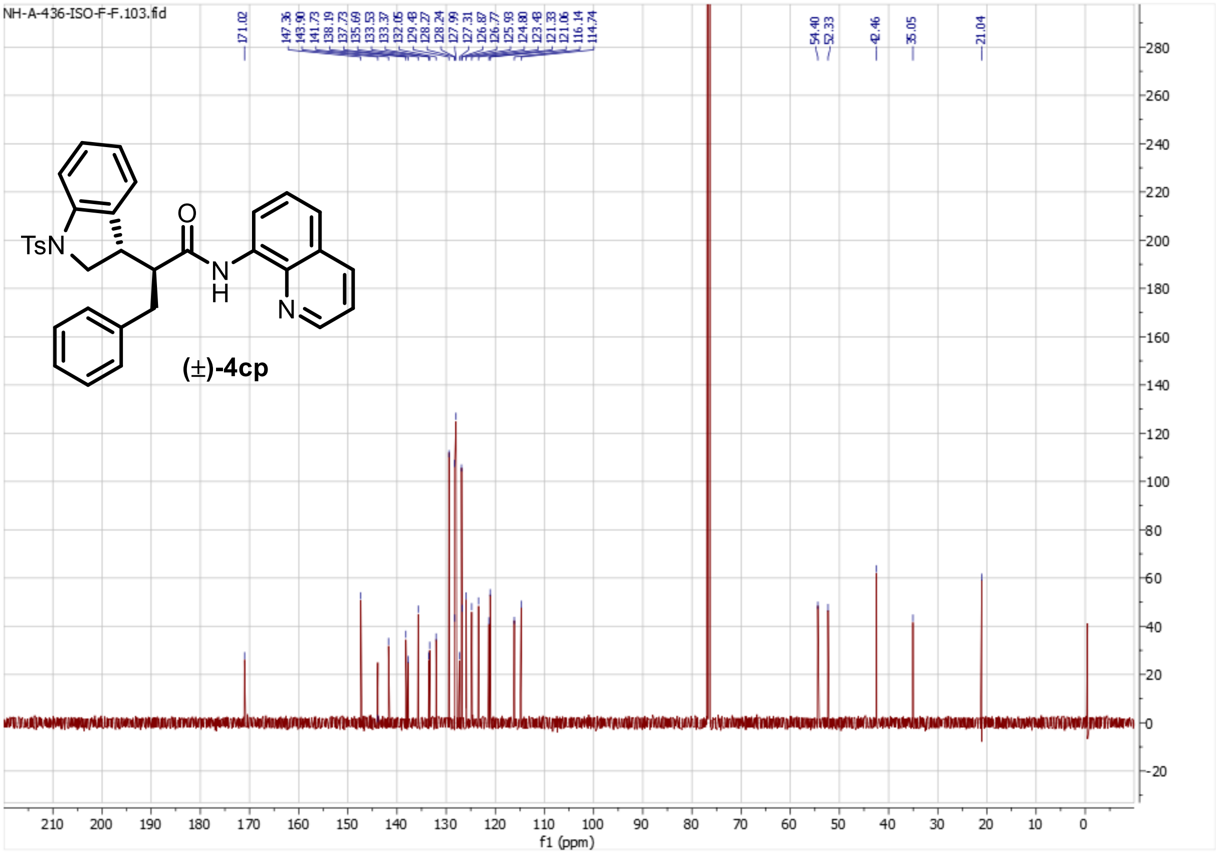
NH-A-436-ISO-F-F.103.fid
171.02
147.36 143.90 141.73 138.19 137.73 135.69 133.53 133.37 132.05 129.43 128.27 128.24 127.99 127.31 126.87 126.77 125.93 124.80 123.43 121.33 121.06 116.14 114.74
54.40 52.33
42.46
35.05
21.04
TsN
O
N
H
N
(±)-4cp
f1 (ppm)

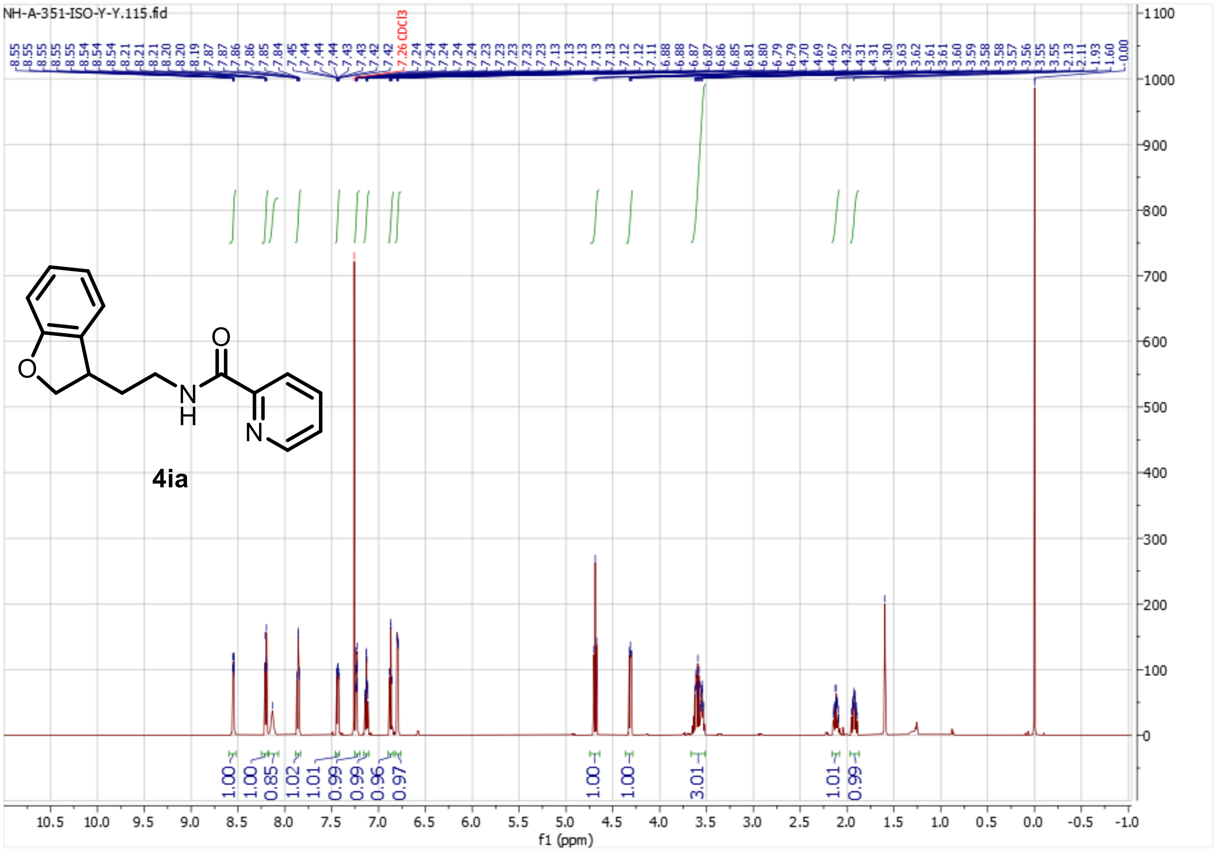
NH-A-351-ISO-Y-Y.115.fid
8.55 8.55 8.55 8.55 8.55 8.54 8.54 8.21 8.21 8.21 8.20 8.20 8.19 7.87 7.87 7.86 7.85 7.84 7.45 7.44 7.44 7.43 7.43 7.42 7.42 7.26 CDCl3 7.24 7.24 7.24 7.24 7.24 7.23 7.23 7.23 7.23 7.23 7.13 7.13 7.13 7.13 7.13 7.12 7.12 7.11 6.88 6.88 6.87 6.87 6.86 6.85 6.81 6.80 6.79 6.79 4.70 4.69 4.67 4.32 4.31 4.31 4.30 3.63 3.62 3.61 3.61 3.60 3.59 3.58 3.58 3.57 3.56 3.55 3.55 2.13 2.11 1.93 1.60 -0.00
O
O
N
H
N
4ia
1.00 1.00 0.85 1.02 1.01 0.99 0.99 0.96 0.97 1.00 1.00 3.01 1.01 0.99
f1 (ppm)

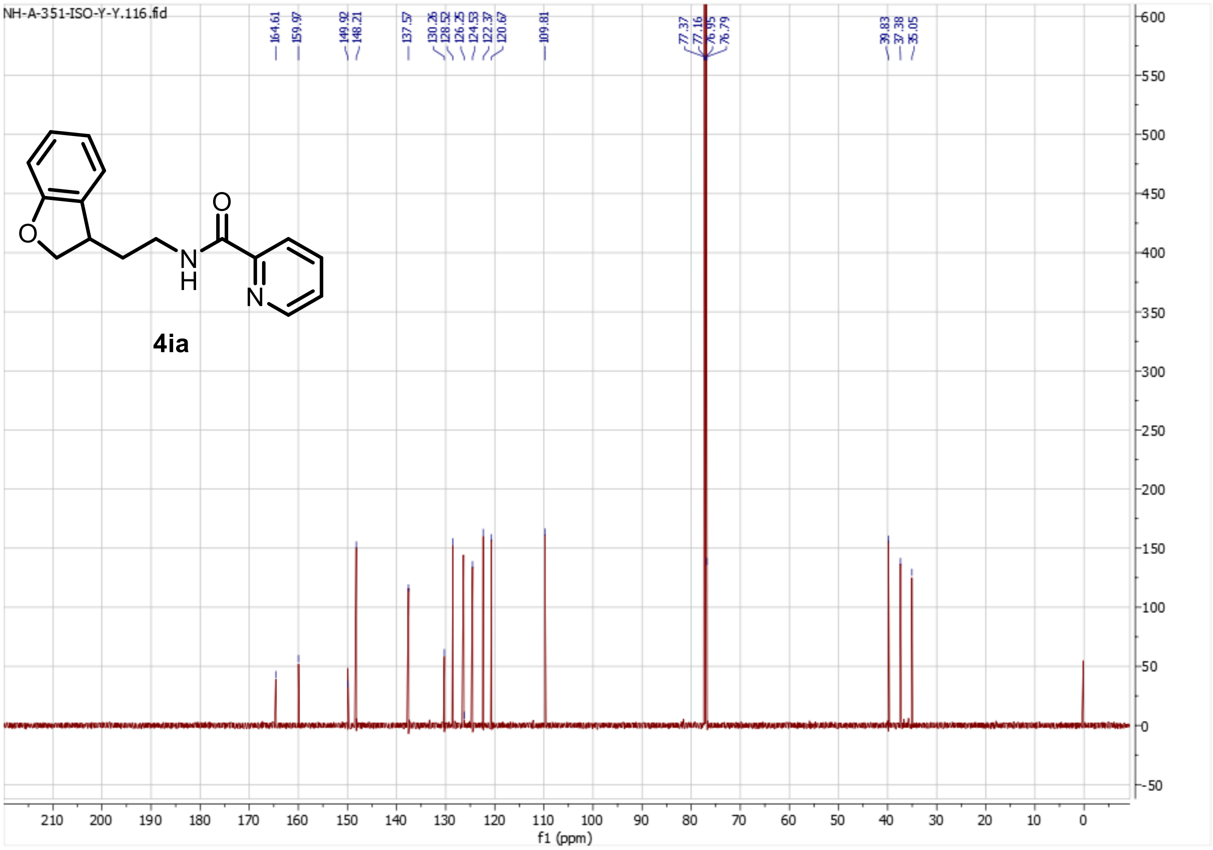
NH-A-351-ISO-Y-Y.116.fid
164.61
159.97
149.92
148.21
137.57
130.26
128.52
126.25
124.53
122.37
120.67
109.81
77.37
77.16
76.95
76.79
39.83
37.38
35.05
O
O
N
H
N
4ia
f1 (ppm)

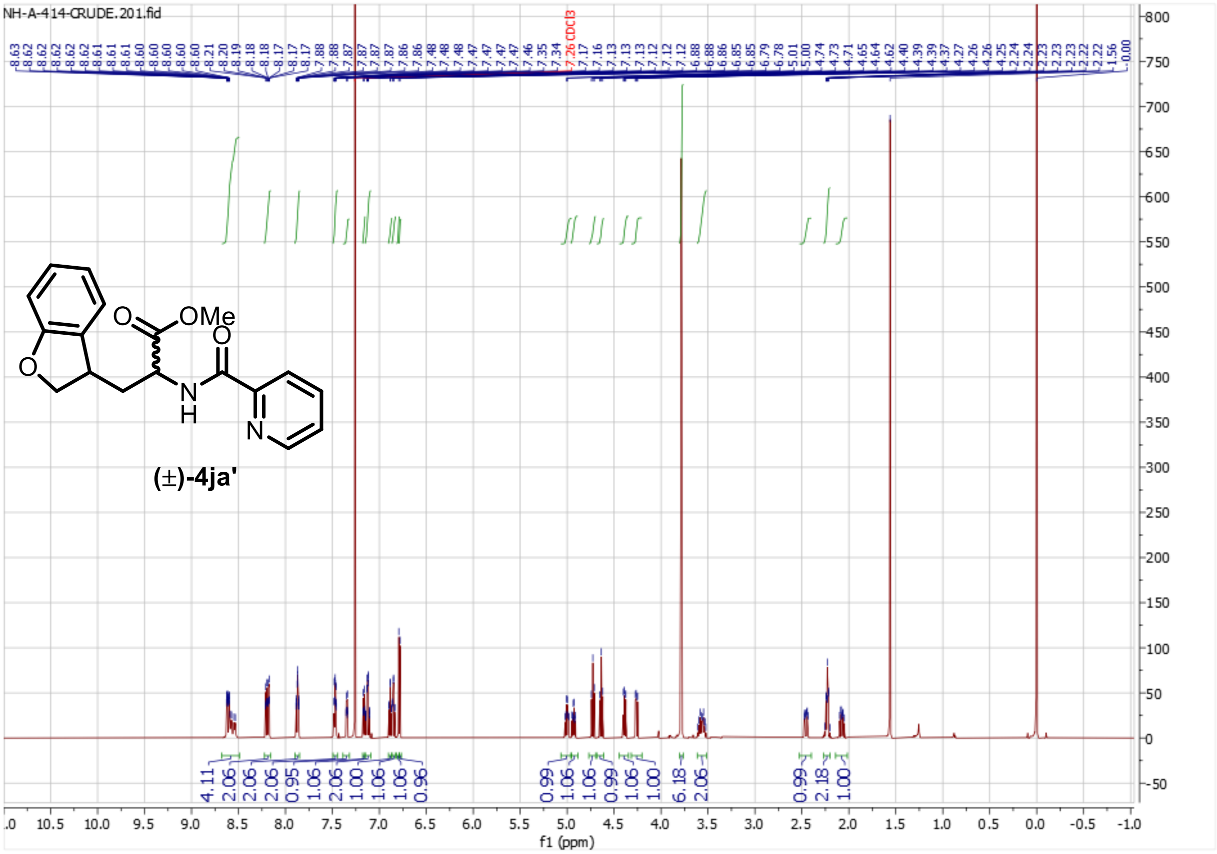
NH-A-414-CRUDE.201.fid
7.26 CDCl3
O
O
OMe
O
N
H
N
(±)-4ja'
f1 (ppm)

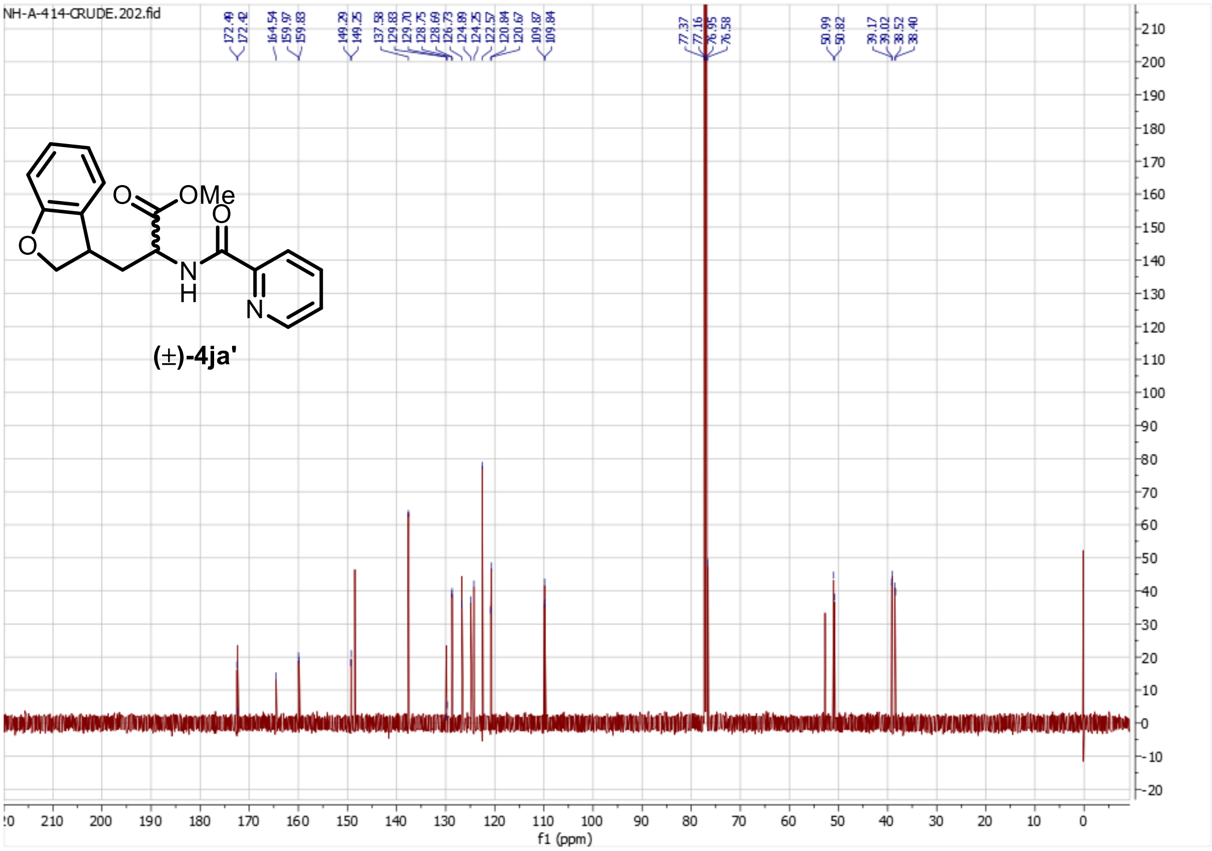
NH-A-414-CRUDE.202.fid
OMe
O
O
N
H
N
(±)-4ja'
f1 (ppm)

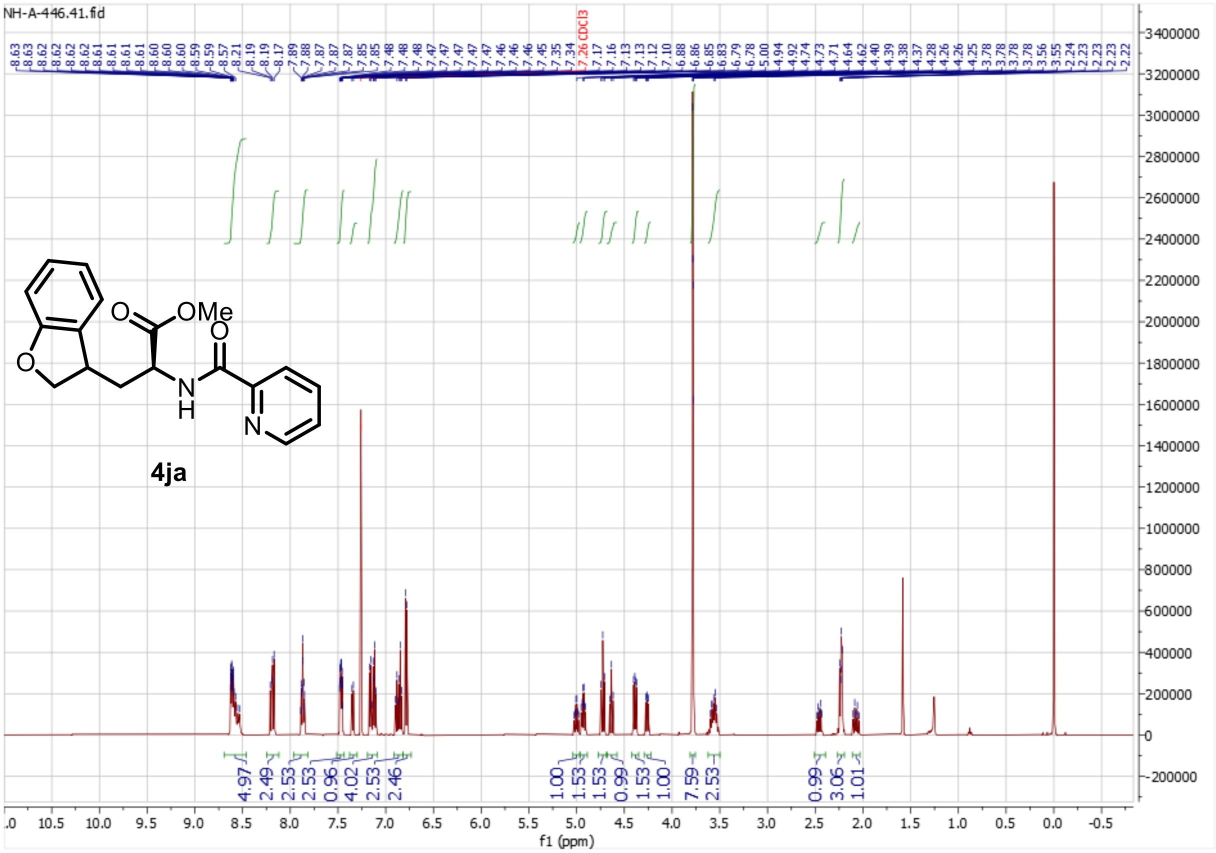
NH-A-446.41.fid
7.26 CDCl3
OMe
O
O
N
H
N
4ja
f1 (ppm)

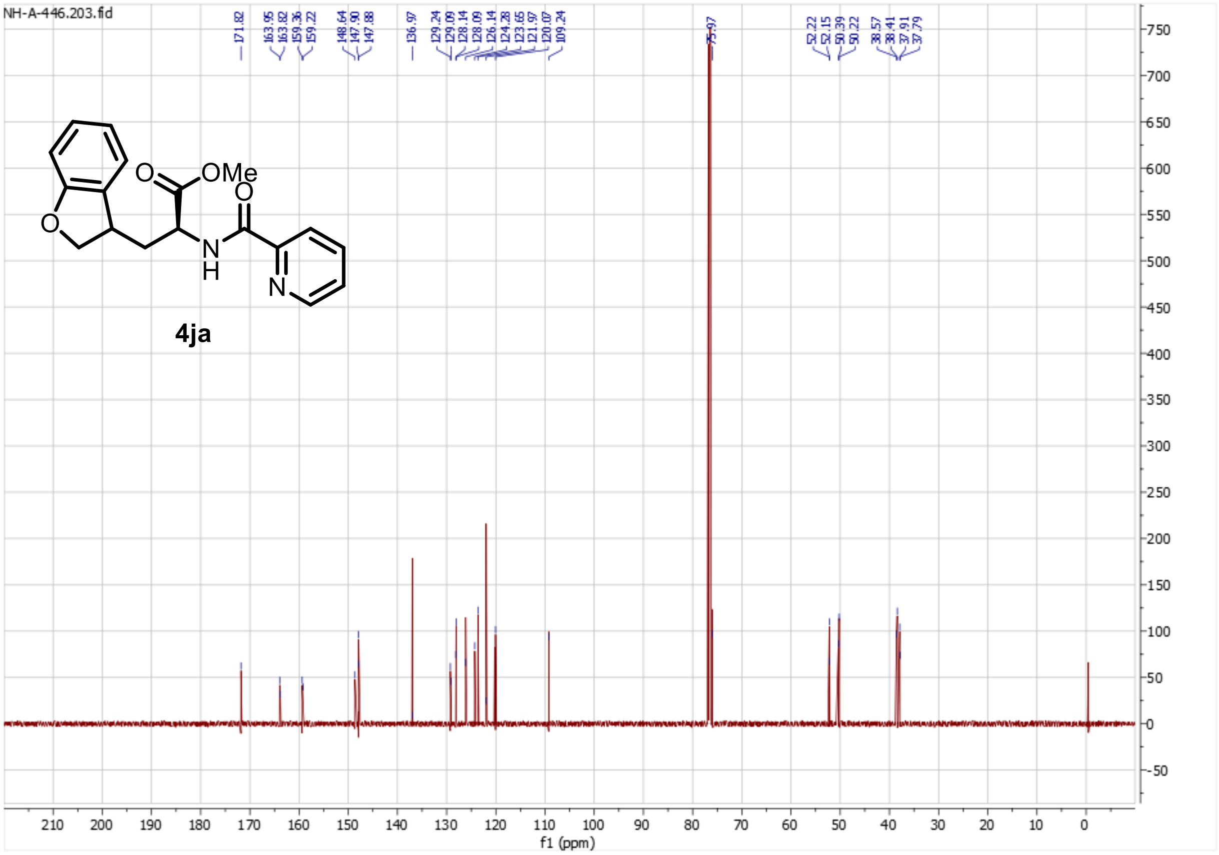
NH-A-446.203.fid
171.82
163.95
163.82
159.36
159.22
148.64
147.90
147.88
136.97
129.24
129.09
128.14
128.09
126.14
124.28
123.65
121.97
120.07
109.24
75.97
52.22
52.15
50.39
50.22
38.57
38.41
37.91
37.79
OMe
O
O
O
N
H
N
4ja
f1 (ppm)

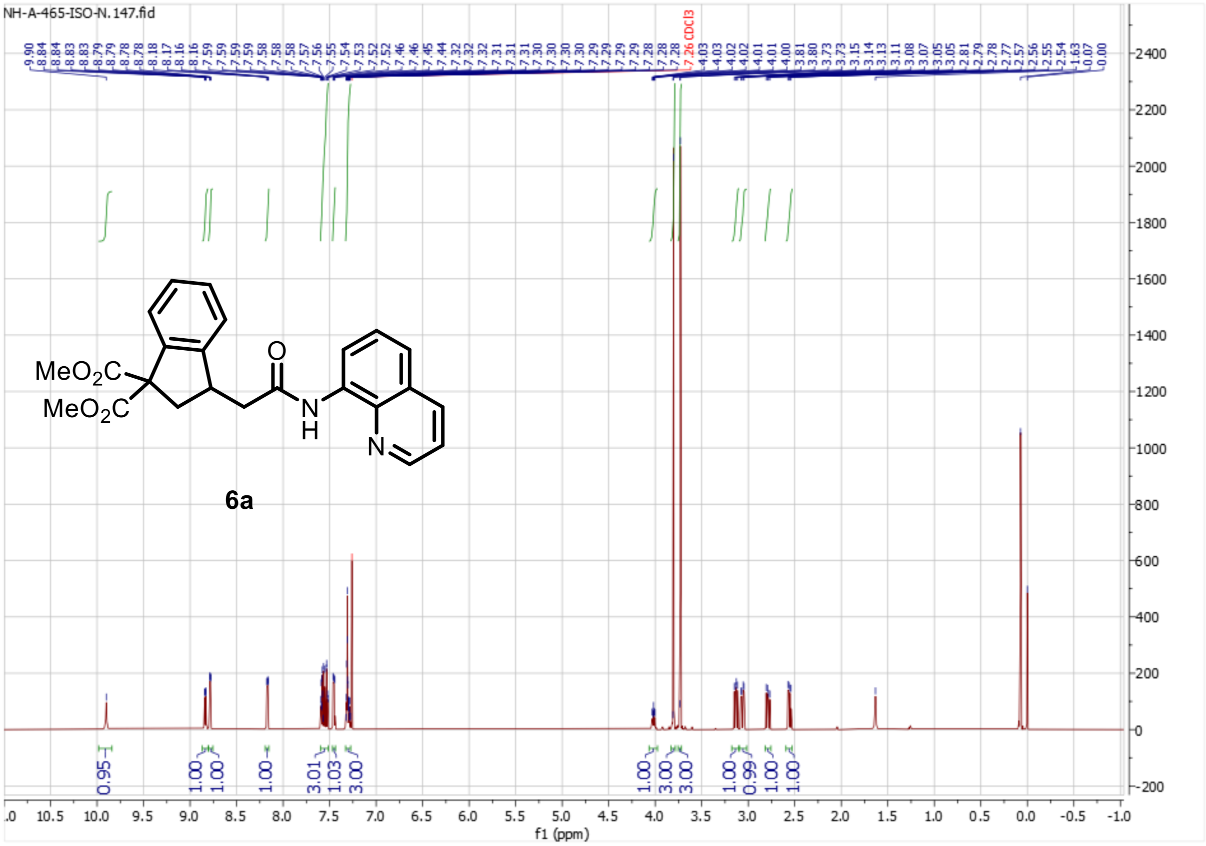
NH-A-465-ISO-N.147.fid
7.26 CDCl3
MeO2C
MeO2C
O
N
H
N
6a
0.95
1.00
1.00
1.00
3.01
1.03
3.00
1.00
3.00
3.00
1.00
0.99
1.00
1.00
f1 (ppm)

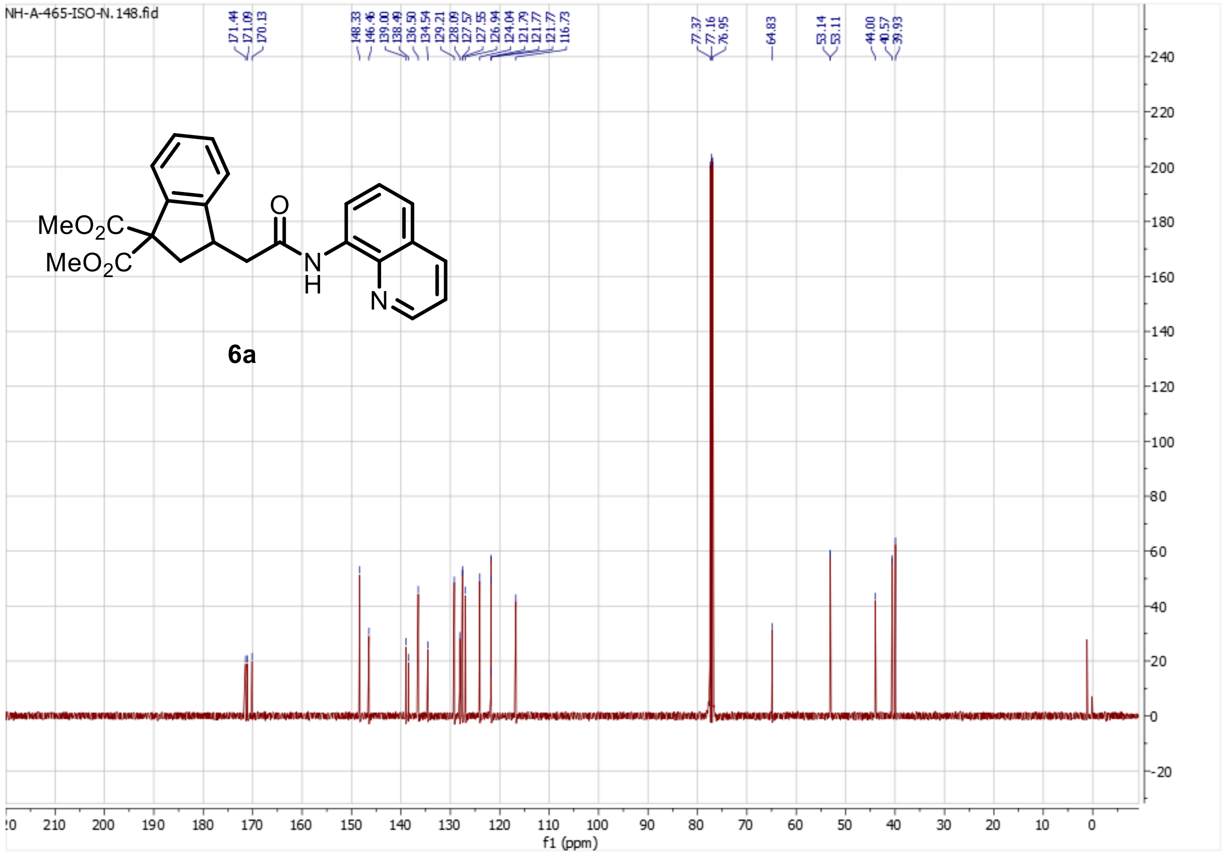
NH-A-465-ISO-N.148.fid
171.44
171.09
170.13
148.33
146.46
139.00
138.49
136.50
134.54
129.21
128.09
127.57
127.55
126.94
124.04
121.79
121.77
121.77
116.73
77.37
77.16
76.95
64.83
53.14
53.11
44.00
40.57
39.93
MeO2C
MeO2C
O
N
H
N
6a
f1 (ppm)

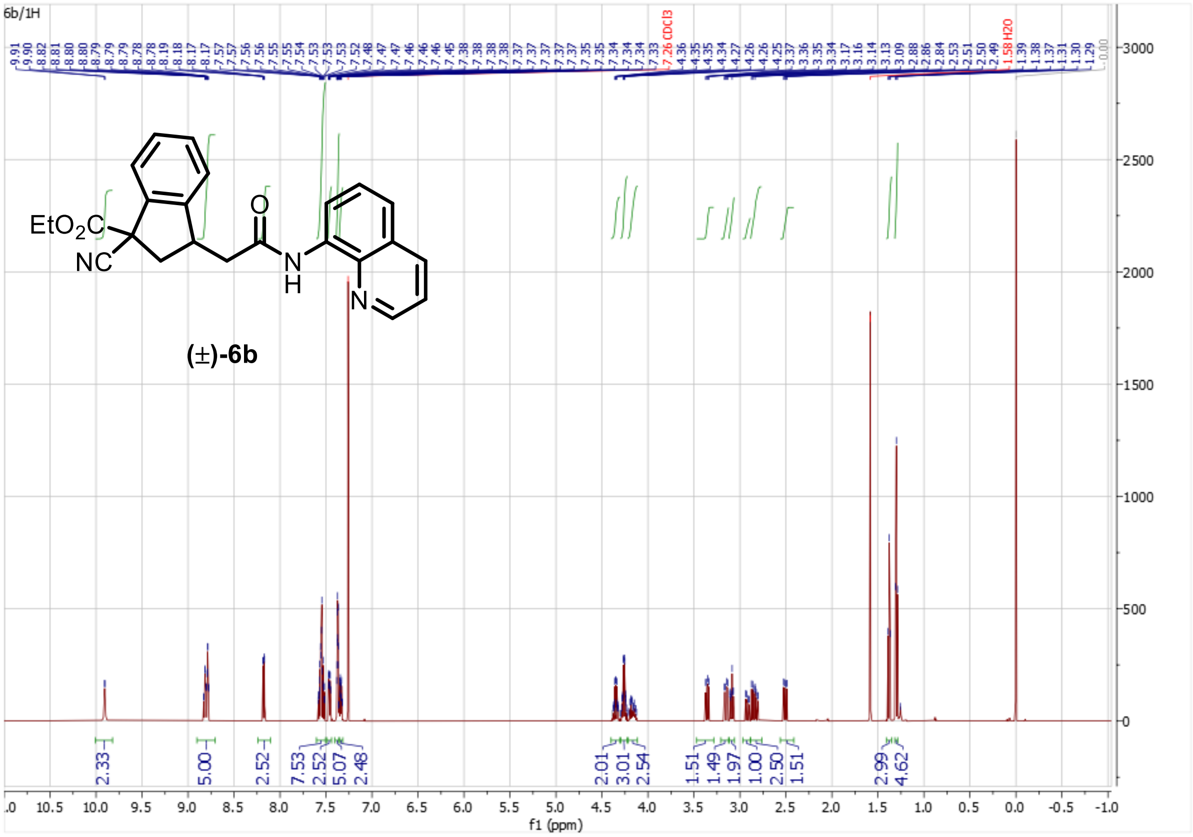
6b/1H
7.26 CDCl3
1.58 H2O
EtO2C
NC
O
N
H
N
(±)-6b
2.33
5.00
2.52
7.53
2.52
5.07
2.48
2.01
3.01
2.54
1.51
1.49
1.97
1.00
2.50
1.51
2.99
4.62
f1 (ppm)

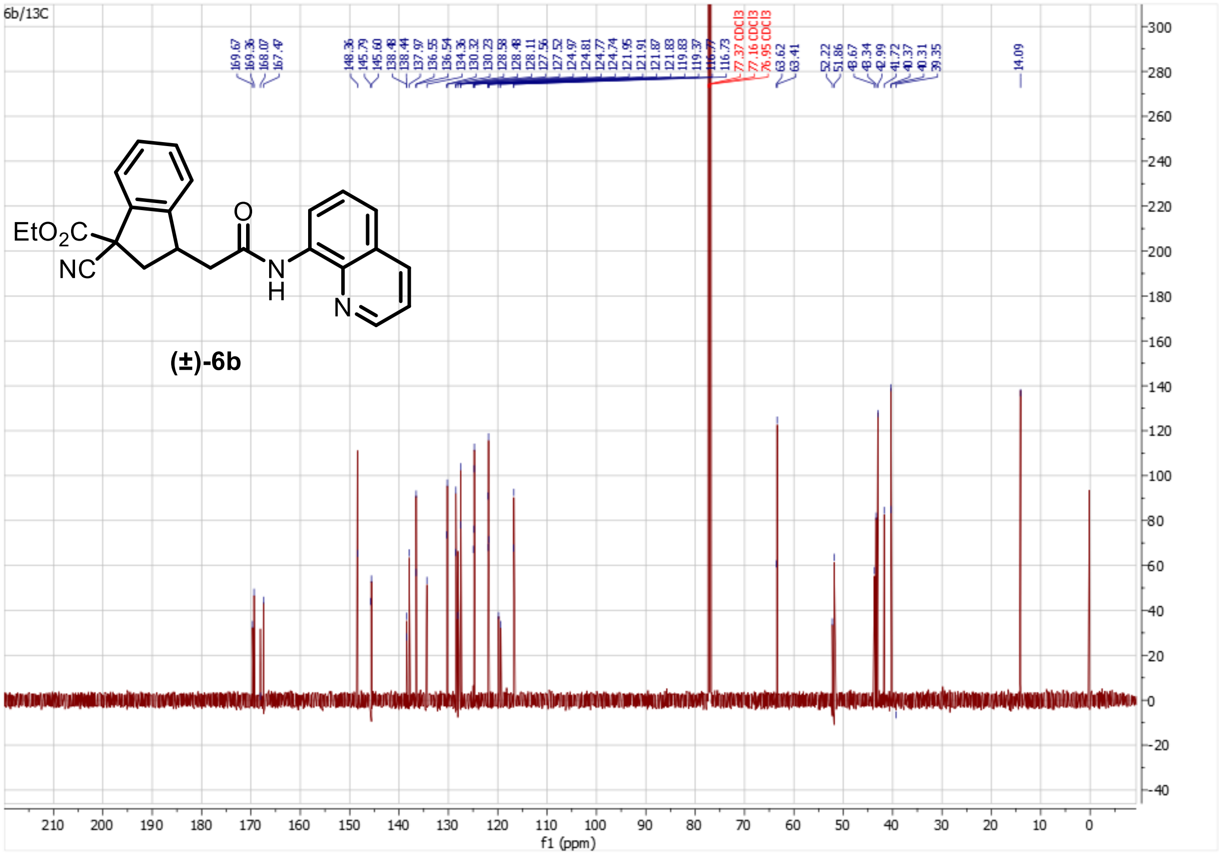

(±)-6b

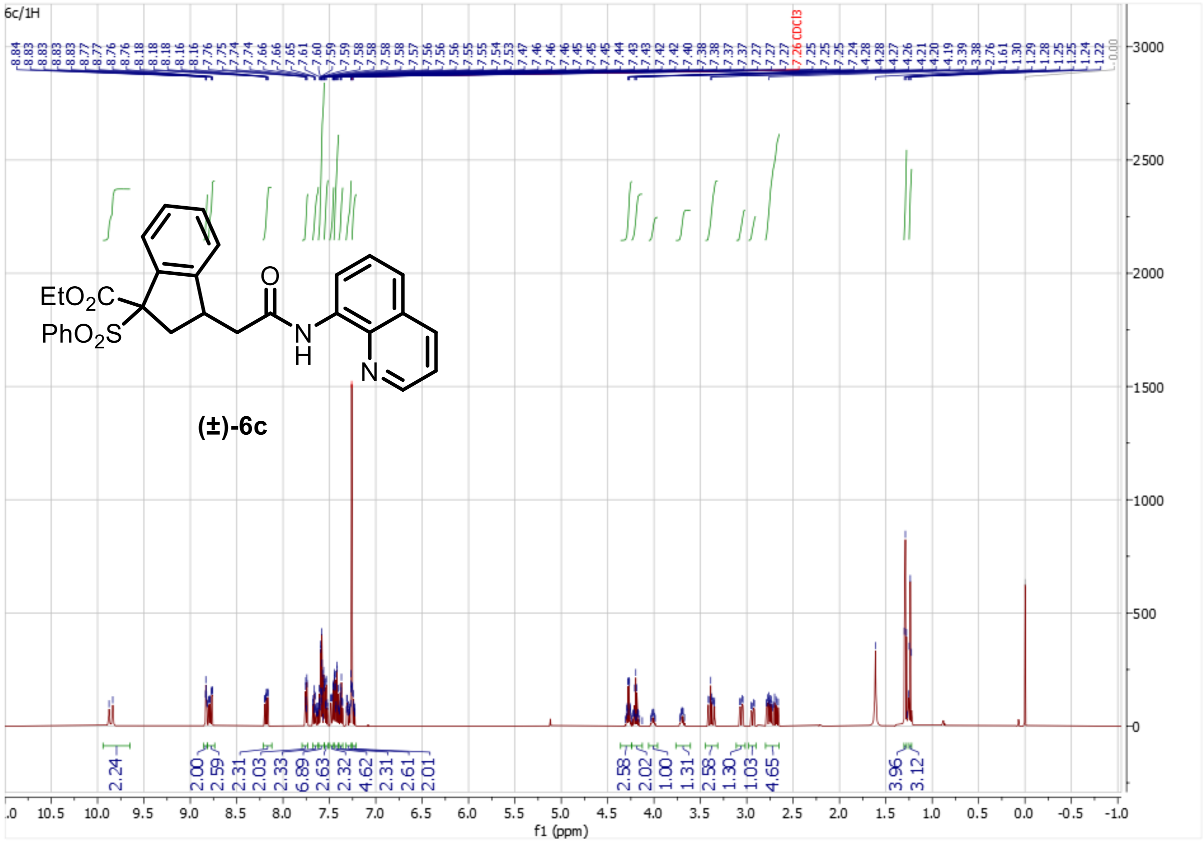

(±)-6c

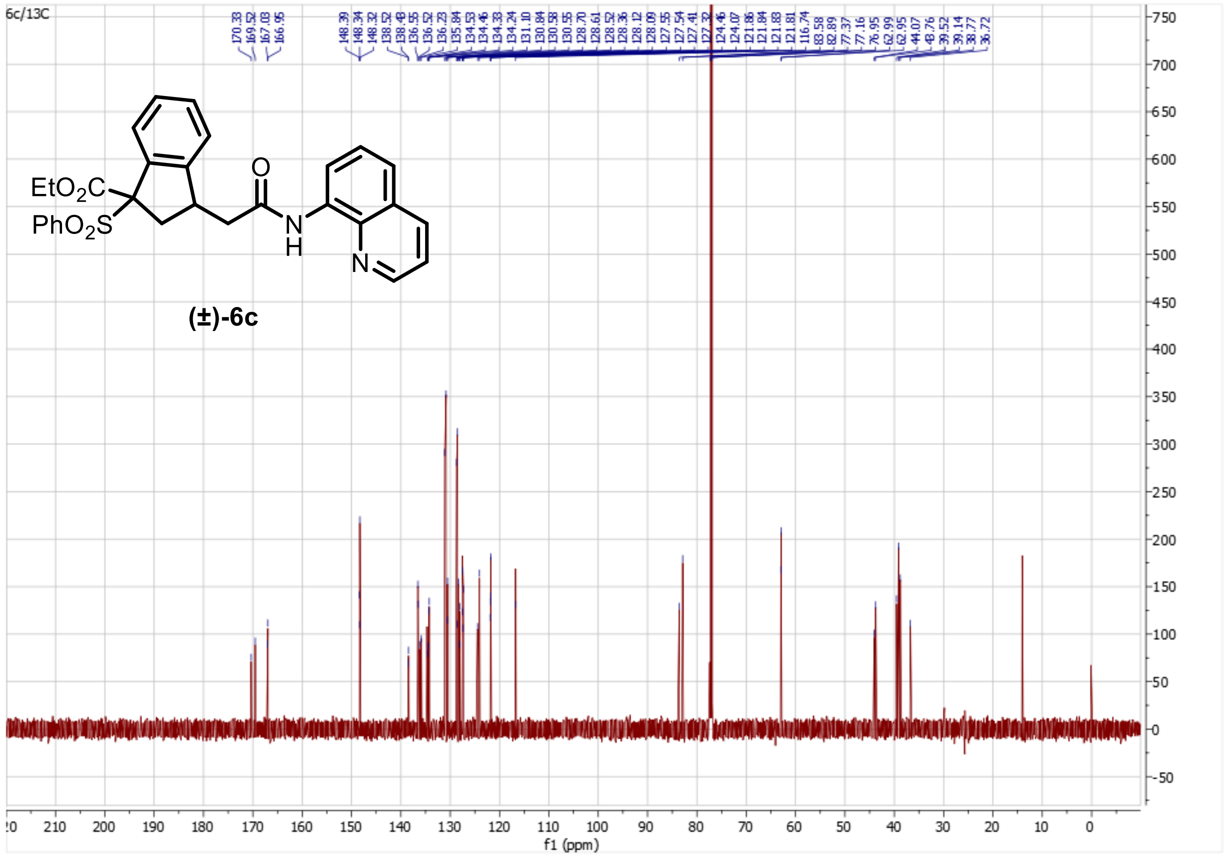
6c/13C
EtO2C
PhO2S
O
N
H
N
(±)-6c
f1 (ppm)

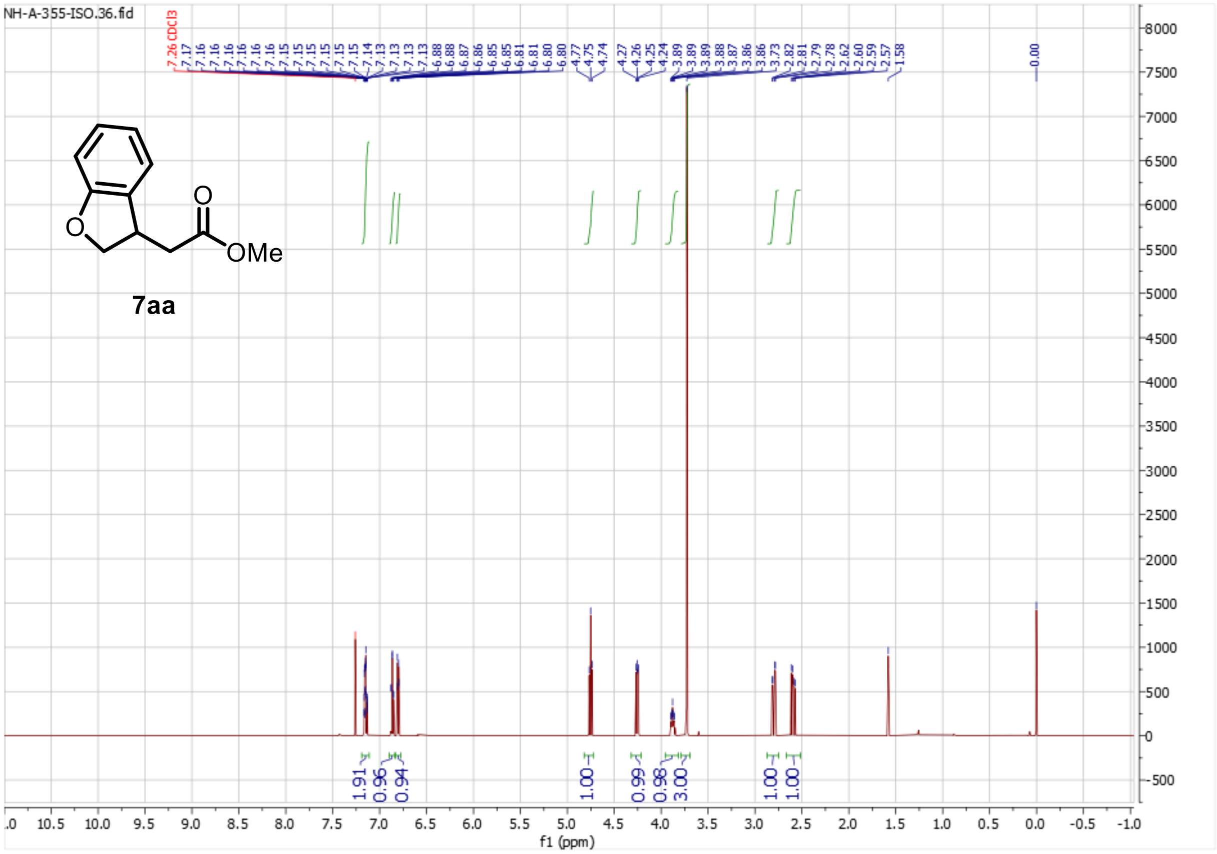
NH-A-355-ISO.36.fid
7aa
OMe
f1 (ppm)

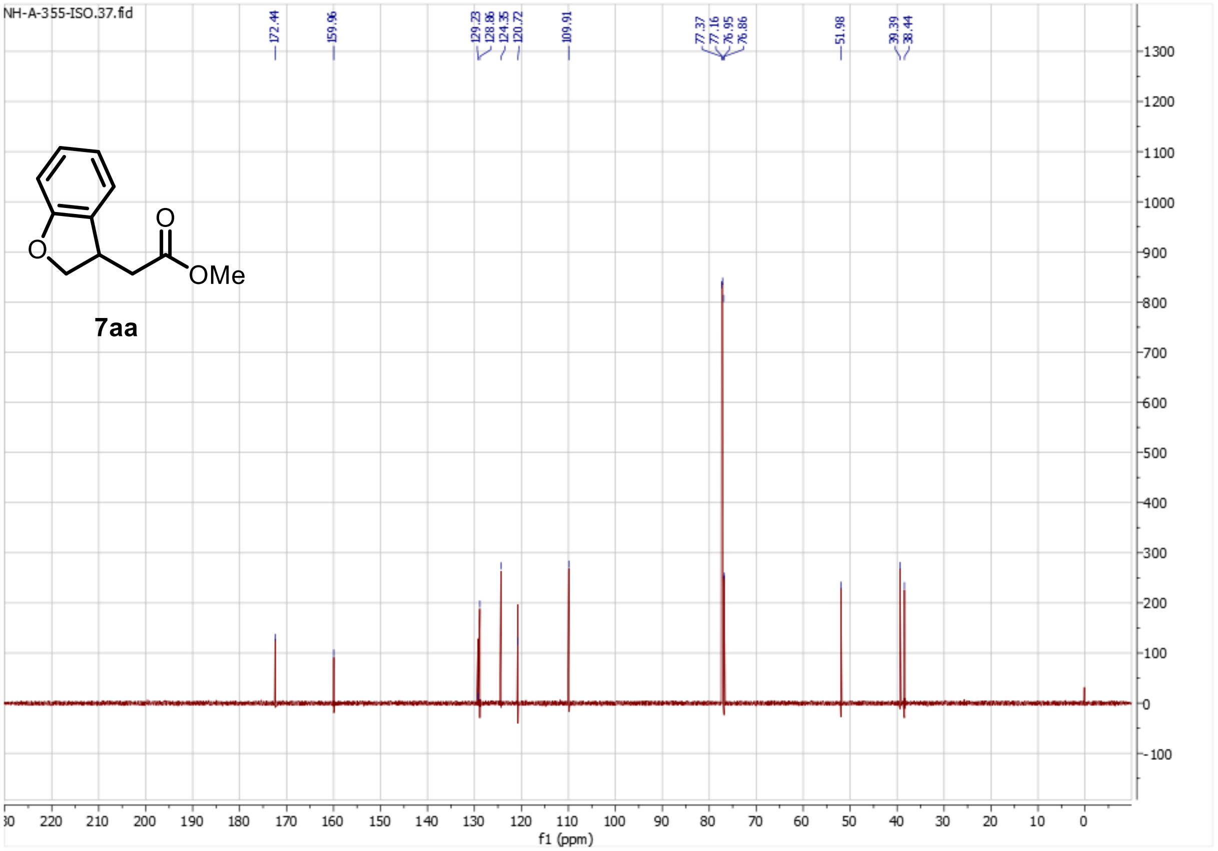
NH-A-355-ISO.37.fid
7aa
OMe
f1 (ppm)

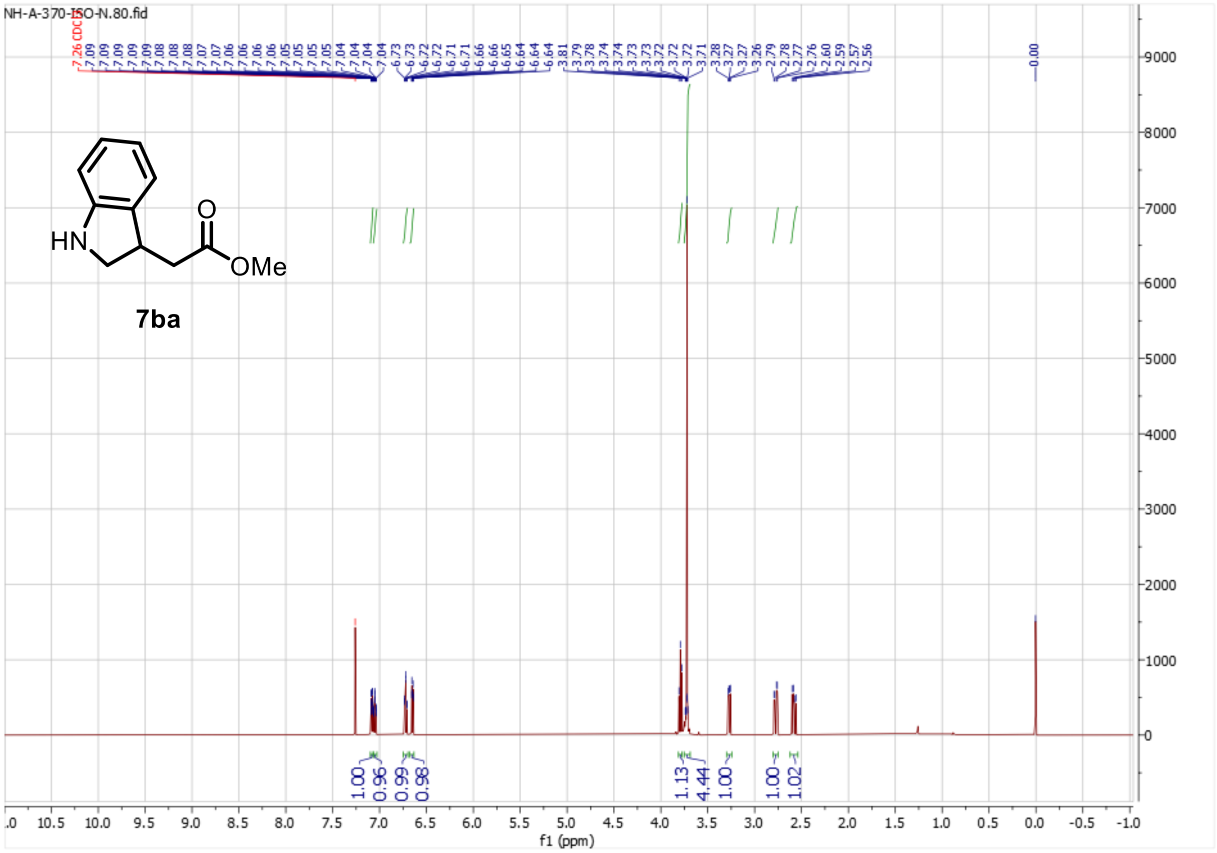
NH-A-370-ISO-N.80.fid
7ba
HN
O
OMe
1.00
0.96
0.99
0.98
1.13
4.44
1.00
1.00
1.02
f1 (ppm)

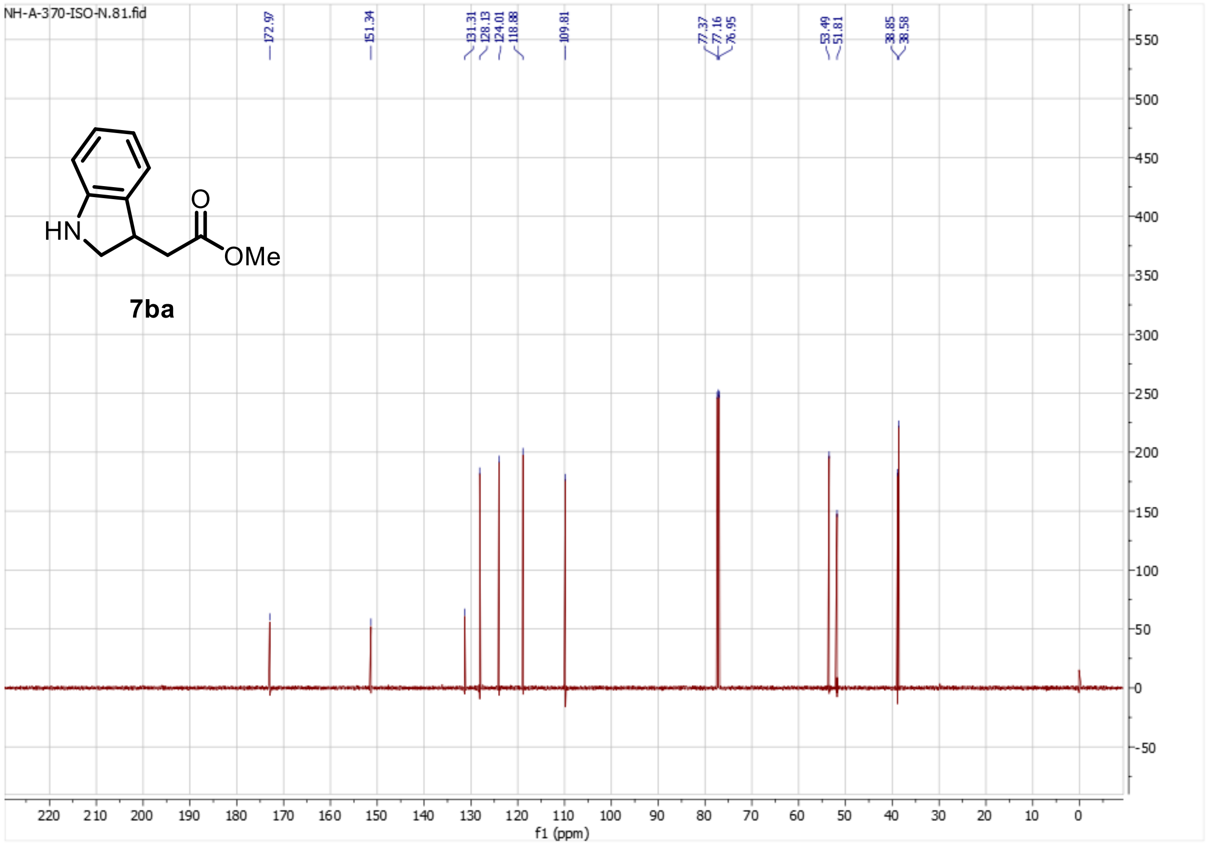
NH-A-370-ISO-N.81.fid
172.97
151.34
131.31
128.13
124.01
118.88
109.81
77.37
77.16
76.95
53.49
51.81
38.85
38.58
7ba
HN
O
OMe
f1 (ppm)

SUPPLEMENTARY REFERENCES

(1) Gurak, J. A.; Jr., Yang, K. S.; Liu, Z.; Engle, K. M. Directed, Regiocontrolled Hydroamination of Unactivated Alkenes via Protodepalladation. *J. Am. Chem. Soc.* **138**, 5805–5808 (2016).

(2) Liu, Z.; Zeng, T.; Yang, K. S.; Engle, K. M. β,γ-Vicinal Dicarbofunctionalization of Alkenyl Carbonyl Compounds via Directed Nucleopalladation. *J. Am. Chem. Soc.* **138**, 15122–15125 (2016).

(3) Liu, Z.; Ni, H.-Q.; Zeng, T.; Engle, K. M. Catalytic Carbo- and Aminoboration of Alkenyl Carbonyl Compounds via Five- and Six-Membered Palladacycles. *J. Am. Chem. Soc*. **140**, 3223–3227 (2018).

(4) Zeng, T.; Liu, Z.; Schmidt, M. A.; Eastgate, M. D.; Engle, K. M. Directed, Palladium(II)-Catalyzed Intermolecular Aminohydroxylation of Alkenes Using a Mild Oxidation System. *Org. Lett.* **20**, 3853−3857 (2018).

(5) Liu, Z.; Li, X.; Zeng, T.; Engle, K. M. Directed, Palladium(II)-Catalyzed Enantioselective *anti*-Carboboration of Alkenyl Carbonyl Compounds. *ACS Catal.* **9**, 3260–3265 (2019).

(6) O'Duill, M. L.; Matsuura, R.; Wang, Y.; Turnbull, J. L.; Gurak, J. A., Jr.; Gao, D.-W.; Lu, G.; Liu, P.; Engle, K. M. Tridentate Directing Groups Stabilize 6-Membered Palladacycles in Catalytic Alkene Hydrofunctionalization. *J. Am. Chem. Soc.* **139**, 15576– 15579 (2017).

(7) Liu, Z.; Wang, Y.; Wang, Z.; Zeng, T.; Liu, P.; Engle, K. M. Catalytic Intermolecular Carboamination of Unactivated Alkenes via Directed Aminopalladation. *J. Am. Chem. Soc.* **139**, 11261−11270 (2017).

(8) Yang, K. S.; Gurak, J. A., Jr.; Liu, Z.; Engle, K. M. Catalytic, Regioselective Hydrocarbofunctionalization of Unactivated Alkenes with Diverse C–H Nucleophiles. *J. Am. Chem. Soc.* **138**, 14705−14712 (2016).

(9) Lv, H.; Xiao, L.-J.; Zhao, D.; Zhou, Q.-L. Nickel(0)-Catalyzed Linear-Selective Hydroarylation of Unactivated Alkenes and Styrenes with Aryl Boronic Acids. *Chem. Sci.* **9**, 6839−6843 (2018).

(10) Zhu, D.; Ma, J.; Luo, K.; Fu, H.; Zhang, L.; Zhu, S. Enantioselective Intramolecular C−H Insertion of Donor and Donor/Donor Carbenes by a Nondiazo Approach. *Angew. Chem., Int. Ed.* **55**, 8452−8456 (2016).

(11) Bressy, C.; Alberico, D.; Lautens, M. A Route to Annulated Indoles via a Palladium-Catalyzed Tandem Alkylation/Direct Arylation Reaction. *J. Am. Chem. Soc.* **127**, 13148–13149 (2005).

(12) I. Nakamura, Y. Sato, S. Konta, M. Terada. Platinum-catalyzed consecutive C–N bond formation-[1,3] shift of carbamoyl and ester groups. *Tetrahedron Lett.* **50**, 2075–2077.

(13) Zhang, X.; Zeng, W.; Yang, Y.; Huang, H.; Liang, Y. Copper-Catalyzed Double C–S Bonds Formation via Different Paths: Synthesis of Benzothiazoles from N-Benzyl-2-iodoaniline and Potassium Sulfide. *Org. Lett.* **16**, 876–879 (2014).

(14) Yang, W.; Liu, Y.; Zhang, S.; Cai, Q. Copper-Catalyzed Intramolecular Desymmetric Aryl C–O Coupling for the Enantioselective Construction of Chiral Dihydrobenzofurans and Dihydrobenzopyrans. *Angew. Chem., Int. Ed*. **54**, 8805–8808 (2015).

(15) Tran V. T.; Gurak J. A.; Yang K.; Engle K. M. Activation of Diverse Carbon–Heteroatom and Carbon–Carbon Bonds via Palladium(II)-Catalysed β-X Elimination. *Nat. Chem.* **10**, 1126−1133 (2016).

(16) CCDC 1999888, 1999889, 2031197 and 2033387 contain the supplementary crystallographic data for this paper. These data can be obtained free of charge from The Cambridge Crystallographic Data Centre via www.ccdc.cam.ac.uk/data_request/cif.

(17) Gaussian 16, Revision C.01, Frisch, M. J.; Trucks, G. W.; Schlegel, H. B.; Scuseria, G. E.; Robb, M. A.; Cheeseman, J. R.; Scalmani, G.; Barone, V.; Petersson, G. A.; Nakatsuji, H.; Li, X.; Caricato, M.; Marenich, A. V.; Bloino, J.; Janesko, B. G.; Gomperts, R.; Mennucci, B.; Hratchian, H. P.; Ortiz, J. V.; Izmaylov, A. F.; Sonnenberg, J. L.; Williams-Young, D.; Ding, F.; Lipparini, F.; Egidi, F.; Goings, J.; Peng, B.; Petrone, A.; Henderson, T.; Ranasinghe, D.; Zakrzewski, V. G.; Gao, J.; Rega, N.; Zheng, G.; Liang, W.; Hada, M.; Ehara, M.; Toyota, K.; Fukuda, R.; Hasegawa, J.; Ishida, M.; Nakajima, T.; Honda, Y.; Kitao, O.; Nakai, H.; Vreven, T.; Throssell, K.; Montgomery, J. A., Jr.; Peralta, J. E.; Ogliaro, F.; Bearpark, M. J.; Heyd, J. J.; Brothers, E. N.; Kudin, K. N.; Staroverov, V. N.; Keith, T. A.; Kobayashi, R.; Normand, J.; Raghavachari, K.; Rendell, A. P.; Burant, J. C.; Iyengar, S. S.; Tomasi, J.; Cossi, M.; Millam, J. M.; Klene, M.; Adamo, C.; Cammi, R.; Ochterski, J. W.; Martin, R. L.; Morokuma, K.; Farkas, O.; Foresman, J. B.; Fox, D. J. Gaussian, Inc., Wallingford CT, 2016.
(18) (a) Lee, C.; Yang, W.; Parr, R. G. Development of the Colle-Salvetti Correlation-Energy Formula into a Functional of the Electron Density. *Phys. Rev. B* **37**, 785–789 (1988); (b) Becke, A. D. Density-Functional Thermochemistry. III. The Role of Exact Exchange." *J. Chem. Phys.* **98**, 5648–5652 (1993).
(19) Grimme, S.; Antony, S.; Ehrlich, S.; Krieg, S. "A Consistent and Accurate *Ab Initio* Parametrization of Density Functional Dispersion Correction (DFT-D) for the 94 Elements H-Pu", *J. Chem. Phys.* **132**, 154104 (2010).
(20) Marenich, A. V.; Cramer, C. J.; Truhlar, D. G. Universal Solvation Model Based on Solute Electron Density and on a Continuum Model of the Solvent Defined by the Bulk S-95 Dielectric Constant and Atomic Surface Tensions. *J. Phys. Chem. B* **113**, 6378–6396 (2009).
(21) Zhao, Y.; Truhlar, D. G. The M06 Suite of Density Functionals for Main Group Thermochemistry, Thermochemical Kinetics, Noncovalent Interactions, Excited States, and Transition Elements: Two New Functionals and Systematic Testing of Four M06-Class Functionals and 12 Other Functionals. *Theor. Chem. Acc.* **120**, 215–241 (2008).
(22) Ribeiro, R. F.; Marenich, A. V.; Cramer, C. J.; Truhlar, D. G. Use of Solution-Phase Vibrational Frequencies in Continuum Models for the Free Energy of Solvation. *J. Phys. Chem. B* 14556−14562 (2011).
(23) Mammen, M.; Shakhnovich, E. I.; Deutch, J. M.; Whitesides, G. M. Estimating the Entropic Cost of Self-Assembly of Multiparticle Hydrogen-Bonded Aggregates Based on the Cyanuric Acid·Melamine Lattice. *J. Org. Chem.* **63**, 3821–3830 (1998).
(24) Zhang, D.; Yum, E. K.; Liu, Z.; Larock, R. C. Synthesis of Indenes by the Palladium-CatalyzedCarboannulation of Internal Alkynes. *Org. Lett.* **7**, 4963 (2005).
