# Supplementary material for: Anti-selective [3+2] (Hetero)annulation of non-conjugated alkenes via directed nucleopalladation
Source: Nat Commun. 2020 Dec 22;11:6432. doi: 10.1038/s41467-020-20182-4 (PMC7755910; doi:10.1038/s41467-020-20182-4)
Supplement: Supplementary file 3 — Description of Additional Supplementary Files [file 41467_2020_20182_MOESM3_ESM.pdf]

Description of Additional Supplementary Files

File Name: Supplementary Data 1
Description: DFT calculations on the reaction of alkene **1a** and iodophenol **2aa**
